# Supplementary material for: Mitigating the identity and health threat of COVID-19: Perspectives of middle-class South Asians living in the UK
Source: J Health Psychol. 2021 Jun 22;27(9):2147–60. doi: 10.1177/13591053211027626 (PMC9353968; doi:10.1177/13591053211027626)
Supplement: sj-docx-1-hpq-10.1177_13591053211027626 – for Mitigating the identity and health threat of COVID-19: Perspectives of middle-class South Asians living in the UK [file sj-docx-1-hpq-10.1177_13591053211027626.docx]

**Covid19BAME**

Food habits and lifestyle as well. Although, as South Asians we already have a lot of good habits when we eat a lot of turmeric, cloves or all these things are really healthy. At the same time, we also have a lot of unhealthy habits. Even though we have those good habits, we don’t really implement it. Our ancient knowledge is there. We don’t implement it in our lives nowadays.

Code: Community cultural commonalities\diet and exercise\Diet

Participant08

We are living a very urban life. At the same time, the social thing is that people, there are any number of people in Asian houses, households. There are a number of people.

Code: Community cultural commonalities\Multi-generational living

Participant08

There is more social cohesion rather, maybe, less distancing. Distancing is kind of frowned at in our culture. These can be a problem, I think

Code: Community cultural commonalities\The collective nature of SA culture makes distancing more diff

Participant08

How much Vitamin D do we have in our body? Vitamin D has been known to be able to better resist, grow resistance against this virus. The thing is, if we don’t get enough sunlight, we won’t have enough Vitamin D. You can say that this is true for everyone and not just South Asians in this country. At the same time, if you have a darker skin you absorb less Vitamin D. You have to be longer outside, but not as long that you grow more tan because then you absorb less again in a situation. I think, I was discussing with a very good friend of mine and we decided that, okay, if we are going to get the sunlight from the outside it’s better to have it during the afternoon and when the sun is setting and you can have a longer period of time, soaking in the sun. This is one of the problems.

Code: Community cultural commonalities\Physical differences\Vitamin D deficiency

Participant08

There are some concerns that are appearing. I don’t buy it personally, because I haven’t seen it myself that in different countries like USA, black people are being discriminated against or brown people are being discriminated against and so they are not given enough attention in the medical health sector as the white population is. That could be another factor. I am not quite sure about this for UK or even for USA on a large scale. I really don’t think so, but there is a concern.

Code: Institutional trust\Healthcare access\NHS access is equal\Access to healthcare

Participant08

I think I will go back to what I just said, it’s the social behaviour, I think. Also certain religious views they have across different faith systems that we have there are methods of congregating

Code: Drawing differences\religious non compliance

Participant08

The white population also are religious, but then, I don’t really see people gathering in churches like that like some of the mosques. I am sure they are not gathering now. They were at some point. I think it’s a problem world I think. I have seen this issue worldwide among Muslim communities as well, Muslim Asian communities. Even Saudi Arabia, they haven’t lockdown on everything. They are not doing anything. The South Asians are.

Code: Drawing differences\religious non compliance\Islamic noncompliance

Participant08

They are openly saying that this virus is not going to affect you because you are immune to it because you are immune because of God. This is the ignorance that is there among religious people.

Code: Drawing differences\religious non compliance\Islamic noncompliance\Leave it to Allah

Participant08

There was a very funny video. I will send it to you later. It’s really funny. They said that they had an interview with a Coronavirus and the Coronavirus told them that it’s not going to affect Muslims or even if you see some of the BGP leaders doing all these things and saying, go Corona, go. Okay, they are saying it harmless. If they are congregating to do that, it’s more harmful.

Code: Drawing differences\religious non compliance\Islamic noncompliance\Leave it to Allah

Participant08

Is it more in South Asian communities and not in the other population? I doubt. It is across all—when I went out to get food, I see lots of different kinds of people of all backgrounds.

Code: Contesting "SA Community"\Social support\No difference for SA community

Participant08

That is across all backgrounds. I don’t think it is much different among South Asian or other.

Code: Contesting "SA Community"\Social support\No difference for SA community

Participant08

I personally don’t think there is any difference between South Asians or anybody to access the healthcare.

Code: Contesting "SA Community"\Social support\No difference for SA community

Participant08

If tomorrow I have a problem, I can call. I can call and get advice. I will get probably the same treatment or ask to do the same things that somebody else would be asked to do. I am staying here for a long time and I am not even a British citizen. I think I will be treated in the same way, unless I have a different experience.

Code: Contesting "SA Community"\Social support\No difference for SA community

Participant08

It’s a difficult thing to say. I think people do and they don’t. It’s very mixed. In general, people don’t believe in government. Even I don’t blindly believe any government. I try to be as objective as I can because if there is a rule that they are saying that I think in my own understanding that it’s good for me then I think it’s a good thing that the government is doing. Of course, if the government is delaying procedures or delaying certain things, obviously I will be complaining about it or not being very happy about it. I think the general perception among people is their very, there are anti government sentiments among people more than not.

Code: Institutional trust\Government trust\Lack of/mixed trust in government

Participant08

imilar, all these different populations also have similar apprehensions against the government or sometimes they support the government also. It is mixed

Code: Institutional trust\Government trust\Lack of/mixed trust in government

Participant08

I think the government what they can do is they can include some of the religious and community organisations and we can include them to disseminate these kind of information. So that they have less suspicion against the government and they can integrate with each other more, I think. If a person believes in the mosque or the temple more, okay. If somebody from that mosque tells them okay you shouldn’t do this. They will listen to it more.

Code: Community cultural commonalities\The collective nature of SA culture makes distancing more diff\Centrality of community groups in messaging

Participant08

The government can reach out to them with their leaflets or whatever the soft copy information that they have. They can say, they can tell them that you should give this information to your community if you are a member.

Code: Community cultural commonalities\The collective nature of SA culture makes distancing more diff\Centrality of community groups in messaging\messaging through religious groups/leaders

Participant08

There is one basic thing. I got some fire in my house, downstairs, where there is some community members they said, this is not South Asian really and that’s my locality related. They said that they will help out any medical needs to go out to the shop. It was very reassuring. Obviously, I don’t need that. It was reassuring that there is somebody. If tomorrow I fell ill and I can avail it.

Code: Contesting "SA Community"\Social support\support of neighbours

Participant08

I actually know a friend of mine who is South Asian, she is not very well, like she has some complications. The NHS has given her food to her doorstep. That is amazing.

Code: Institutional trust\Healthcare access\NHS access is equal\NHS support

Participant08

Another thing I have seen, this is government level, but even personal level, individual level, some South Asians are reaching out to help others who don’t have enough food. Some people don’t have the money because they don’t, they are not having any work, they lost their work or something. That is happening. That is very positive.

Code: Contesting "SA Community"\Social support\South Asian community

Participant08

We have to really think about development as a collective or at an individual level. Even like Anrad [sounds like] said the single person, the individual is the highest minority in any society. If you understand the individual, whoever it is from whatever background and if we can take it as an individual in a society then we can fix a lot of things.

Code: In-group efficacy\self-responsibility

Participant08

More in South Asian community because we come from these tropical countries and all of this are lack of sunlight over here. And a Vitamin D deficiency also causes certain of the things like depression like lower immunity and then probably those are one of the reasons.

Code: Community cultural commonalities\Physical differences\Vitamin D deficiency

Participant04

Probably that might be one of the reasons. Wherein a pandemic like this would attack a lower immune person faster than others.

Code: Community cultural commonalities\Physical differences\Vitamin D deficiency

Participant04

They also have various things. Again, here, it also depends upon their melanin pigment which is presenting in our skin. The melanin content is very high for us and that’s why we are brown, black, dark or whatever it is. The white skinned people, they do not have much of melanin and there is no requirement of much on that. They are born like through ages and they are brought up here. The requirement of 37.04 compared to us. Vitamin D deficiency, actually is across all the communities.

Code: Community cultural commonalities\Physical differences\Vitamin D deficiency

Participant04

Number two, the kind of food habits. Again, why it is so much the thing is because of heat and eat culture that junk food culture and eating all of these stuff outside has caused obesity, a lack of proper immune system. Third is—

**I:** Do you think if I can just stop you for a moment, do you think it is specific relation to the South Asian community. They have more of this kind of lifestyle?

**R:** No. South Asian community usually have the habit of cooking at home and eating.

**I:** These ones which you mention that it’s across the community or related to the South Asian?

**R:** Across the community. South Asian community see, again, as I told you one of the major reasons could be this.

Code: Community cultural commonalities\diet and exercise

Participant04

And then there are certain sections wherein unhygienic is one of the problems. I would also be dare enough to say certain things openly whereinhere are certain sets of immigrants, economical 38.53. here are certain sets of immigrants who are hoarded into, I am talking not only about South Asian, but various European or whatever it is. Let us concentrate on South Asian. They are hoarded like groups of ten people living in that. Hygienic issues. Hygiene issues. One of those guys working anywhere across would spread it.

Code: Contesting "SA Community"\Heterogeneity of the SA community: like other groups\Recent v established immigrants

Participant04

And then there are certain sections wherein unhygienic is one of the problems. I would also be dare enough to say certain things openly whereinhere are certain sets of immigrants, economical 38.53. here are certain sets of immigrants who are hoarded into, I am talking not only about South Asian, but various European or whatever it is. Let us concentrate on South Asian. They are hoarded like groups of ten people living in that. Hygienic issues. Hygiene issues. One of those guys working anywhere across would spread it.

Code: Drawing differences\Recent immigrant specific\living conditions

Participant04

I keep on seeing my family here is my wife and I. They take care as I told you. Think positive and talk positive things and talk good things. When we are together, we will have fun. That’s it. A healthy mind keeps a healthy body.

Code: Contesting "SA Community"\Social support\Family

Participant04

Everybody is maintaining—when it comes to life everybody will, what do you call, abide by the rules. You can flaunt rules. It is your life. You are responsible for your own life. So, abide by the rules. What people I have around and all we have our calls and all that stuff. Strict social distancing, everybody. They are very strict about it. They are taking care of themselves. They are taking care of the community. They are going for their short walks with their own family to the park and coming back. Only stepping outside for essential purchases or ordering online. Majority of the families are ordering onlin

Code: In-group efficacy

Participant04

ou can flaunt rules. It is your life. You are responsible for your own life. So, abide by the rules.

Code: In-group efficacy\self-responsibility

Participant04

Everybody has calmed down, actually. The hustle and bustle of running around every single day. Even the majority of them are working from home. They have calmed down a lot. I can see that in the people. We have time to at least say hello to each other or phone now. I think it was like people were so busy running around, they never had time to just say hello. I receive calls from a long lost friend and all that stuff who say hello.

Code: Contesting "SA Community"\Social support\No difference for SA community

Participant04

Healthcare has been good. All the minor complaints and all that stuff. Every system has its own flaws. There isn’t a meeting called in an ideal world. Idealism is 100% and 100% expectation is not accepted. Nothing can be perfect. There are flaws in the system. There another flaws in various things. For example, our greatest complaint of any UK citizen is about NHS. NHS are the ones who were giving up their life for others.

Code: Institutional trust\Healthcare access\NHS access is equal

Participant04

Yes. But the only thing is that certain again there are certain communities who have abused the system also. There is also a lot of abuse of the system. Obviously, people who have abused have reduced the facilities and chances for others.

Code: Institutional trust\Healthcare access\NHS access is equal

Participant04

We have a piece of cake and I want to have almost 90% of it singlehandedly and 10% should be shared amongst others. I cannot do that. I have to have my own little bit. I should be responsible enough. There is a system I cannot abuse that system. I should be responsible enough to think of my next person. Probably now, probably now people should by now people should have understood the value of human life. How to use that system properly and not abuse the system. This is most important. Even now if people are certain other people haven’t realised that, I am sorry. God only should help them. Nobody else.

Code: In-group efficacy\self-responsibility

Participant04

As I told you, like every system has its own flaws. South Asian people, okay, I cannot be a representative of all the South Asian people. I was an Indian, myself. I had no belief in immunisation. It maybe they have corrected themselves. The system is again government has its own limitations and you cannot with Boris Johnson the Prime Minister doesn’t have a magic wand in his hand. He can just wave it and turn around things. Everything should undergo a process. A single head or a single human being cannot make a decision. He has to be with the Director of Health and various other associated scientific research people, everybody and has to come to a decision. The decisions were made immediately, corrections were made and brought in social distancing was enforced and everything could be applauded. Initially, 50.50 to a certain extent. Because, by the time we and the government understood the whole thing, the spread was very high. Now, I read in the news that, even in the port of entries as of now, there are what you call quarantine. There is quarantine done now.

Code: Institutional trust\Government trust

Participant04

The government had expectations of 100,000 test to be done by the end of April and they fail. They are trying their best. Bringing in PPE equipment for NHS workers and other key workers, they weren’t able to do that. It’s a failure. They tried it. They are working on it in various ways. Another bigger thing is the testing part of it. 51.58 what he has done. What Rihas done is amazing. The local government they help the small businesses and everybody. That is amazing, actually. Immediately, immediately within seven days the response was there.

Code: Institutional trust\Government trust

Participant04

As I told you, there is a positive side. There is a negative side. We all have to look into what is more positive and how to overcome the negatives. The government is nothing but a government is not an entity, it is we. It is us, the whole government. The more the public, the more public support, the more successful is the government. When the government says lockdown and there is some result and people are sitting in the park doing barbecue and then drinking and so how is government responsible to that? It is a social responsibility is most important for a successful government. Government makes rules. Who follows it? We? If we do not follow the rules and blame the government for failure, that is unaccepted.

Code: In-group efficacy\self-responsibility

Participant04

As I told you, there is a positive side. There is a negative side. We all have to look into what is more positive and how to overcome the negatives. The government is nothing but a government is not an entity, it is we. It is us, the whole government. The more the public, the more public support, the more successful is the government. When the government says lockdown and there is some result and people are sitting in the park doing barbecue and then drinking and so how is government responsible to that? It is a social responsibility is most important for a successful government. Government makes rules. Who follows it? We? If we do not follow the rules and blame the government for failure, that is unaccepted.

Code: Institutional trust\Government trust

Participant04

You see, again, we see okay all you put into only one sector of people, sector of people I know, hundreds of South Asians across Croydon. Everybody is following strictly. They are respecting those. They are only going for essential things or a walk in the morning or in the evening or ordering online and then keeping a safe distance and keeping themselves safe and healthy. That is more than a contribution to the society and more than help to the government.

Code: In-group efficacy

Participant04

As of now, for example, the Prime Minister wrote a personal letter to each and every household in the UK. Everybody received those letters. Number two, the media are helping the government by sending across the message directly to every, it is reaching every home. And then there are social workers as well as Met Police roaming around and then even in the parks and everywhere they are present. They are taking the pain of requesting people believe not to hang around more or there in the parks are in public places. Social distancing at their superstores. All the stores wherein social distancing is strictly maintained. From the government all these rules and regulations have been passed onto various entities and these entities help follow and the general public are following it, that is very nice. What more can we ask for that. What more can we ask.

Code: In-group efficacy\Messaging recieved

Participant04

As of now, for example, the Prime Minister wrote a personal letter to each and every household in the UK. Everybody received those letters. Number two, the media are helping the government by sending across the message directly to every, it is reaching every home. And then there are social workers as well as Met Police roaming around and then even in the parks and everywhere they are present. They are taking the pain of requesting people believe not to hang around more or there in the parks are in public places. Social distancing at their superstores. All the stores wherein social distancing is strictly maintained. From the government all these rules and regulations have been passed onto various entities and these entities help follow and the general public are following it, that is very nice. What more can we ask for that. What more can we ask.

Code: Institutional trust\Government trust

Participant04

I personally, I started a business which closed in just two weeks. There restaurant was closed and all that stuff. Even then like I had to pay my staff from my own personal money and all that stuff. I received a grant from the local government, Croydon Council. I am happy and thankful to the government. They came to my help at the right time and I am really very very thankful to Croydon Council.

Code: Institutional trust\Government trust

Participant04

The way various admissions by government who help keeping the whole community safe has helped.

Code: Institutional trust\Government trust

Participant04

It’s as simple as this, whoever you are, South Asian, European or African or any Americans or anybody are the localites who live here, English. It’s only as simple as like we follow the law of the land. We are safe. Our community is safe and the government is also functioning happily for us

Code: Contesting "SA Community"\Social support\No difference for SA community

Participant04

It’s as simple as this, whoever you are, South Asian, European or African or any Americans or anybody are the localites who live here, English. It’s only as simple as like we follow the law of the land. We are safe. Our community is safe and the government is also functioning happily for us

Code: Contesting "SA Community"\Alternative definitions of community\Local, not SA community

Participant04

Whilst we support the whole system, the system cannot support us. I cannot only accept things. I also have to give back something to the society. That is called social responsibility or society responsibility. That is where the whole community or the whole country will be successful.

Code: In-group efficacy\self-responsibility

Participant04

Yes, following the rules strictly and then social distancing and keeping themselves safe and healthy. That’s it. Every home keeps itself safe and healthy. The whole of society is safe and healthy. That’s it.

Code: Contesting "SA Community"\Social support\No difference for SA community

Participant04

Well the concern is, that many of the South Asian community people have got underlying healthcare conditions- it is diabetes, it is high blood pressure, it is asthma and, uh, old age related other problems. I'm not talking about those transplant people and heart operations, people and all that. Um, South Asian community particularly, do not have the habit of regular exercising. This is one of the main topic.

Code: Community cultural commonalities\Physical differences\Underlying diseases

Participant13

Well the concern is, that many of the South Asian community people have got underlying healthcare conditions- it is diabetes, it is high blood pressure, it is asthma and, uh, old age related other problems. I'm not talking about those transplant people and heart operations, people and all that. Um, South Asian community particularly, do not have the habit of regular exercising. This is one of the main topic.

Code: Community cultural commonalities\diet and exercise

Participant13

If you look at the white people, uh, they are much, much more agile, much of sort of physical activity oriented. You see, the South Asian community do not indulge in many kinds of sports activity, outdoor activity, they are stuck on mostly, on gossip all the time, you know, then too much of political discussion, they are couch potato, watching tv, sports. Therefore they have an underlying health condition. Specially the elderly group, South Asians, Bangladeshi, Pakistani, Indians- it is all the same, because the South Asians, compared to that, the Filipinos are more outgoing that they're getting together, dancing, sports, bla, bla, and all sorts of things, but India, Pakistan, Bangladesh, Nepal, they don’t do it.

Code: Community cultural commonalities\diet and exercise

Participant13

The other thing is that, um, perhaps, they live too many people jointly, uh, that is one of the factors, where the social distancing is not observed. So, they're unknowingly transmitting these disease, that is a concern because they think they are well, but they may not be well. And they only come to note when they are affected, when it is too late because many of the community members have already been affected. There is no way, you know, there is no way of knowing it, because if you have a test today, but after five days you may test positive. So you don't know when you are going to get to that. That is the main difficulty.

Code: Community cultural commonalities\Multi-generational living

Participant13

Well, it has not been affected much in the sense my daughter can work from home, which she is doing and since myself and my wife, we are, retired already. We have no compulsion of being affected because I said that our needs are taken care by my daughter. So personally, I have not been affected.

Code: Community cultural commonalities\Multi-generational living

Participant13

Unless, unless, uh, they can take more precautions and unless they try to segregate the elderly from the younger ones, because in many South Asian families that is not happening, not that they do not have bigger living spirit, but because of their habit, uh, this is something they have to change and they have to take the precaution, or really getting more effected

Code: Community cultural commonalities\Multi-generational living

Participant13

I'm not sure about the medical care I but I don't think there is any discrimination. Uh, well, I wouldn't be able to give any comments on that because I think with all due respect to NHS, they treat everybody fairly and although there has been some who pointed out to their partiality, but I don't subscribe to that.

Code: Institutional trust\Healthcare access\NHS access is equal

Participant13

Well, as far as I know, there is no difficulty in accessing. At least I do not have personal, uh, information except that the family whom I know, although the families, one member is a GP, the other one is a sort of a research guy. Initially there was a little confusion about which hospitals to take, but ultimately, it was delayed by half a day, but then it was sorted out. But that in spite of being a GP, the wife being a GP, in that sort of a time. I'm not sure if it had been an ordinary person, whether it would have taken more time. I have no information because I have not come across any such information from any reliable source anything. I have nothing to tell you about that.

Code: Institutional trust\Healthcare access\NHS access is equal

Participant13

I don't think they should have any difficulty in following it, no, they shouldn't do it because, you see, it's equally applicable for all the communities.

Code: Contesting "SA Community"\Social support\No difference for SA community

Participant13

I don't think they should have any difficulty in following it, no, they shouldn't do it because, you see, it's equally applicable for all the communities.

Code: In-group efficacy

Participant13

I don't think they should have any difficulty in following it, no, they shouldn't do it because, you see, it's equally applicable for all the communities. So you do not know what to do and what not to do. So, you got to be self-disciplined, not because government is telling you; government may say whatever it is, but it is your own responsibility, for your own health, safety and security. You know now, what to do, and what not to do. So it has to be self-disciplined. Forget about what the government is saying, if you are not responsible enough, you behave responsibly, that’s all you need.

Code: In-group efficacy\self-responsibility

Participant13

As far as I know, um, my community, uh, we are from the part, where in India, I see everybody is just taking things seriously and behaving responsibly. I have no, I have no qualm about that. And I know everybody are responsible and behaving in very complaint ways to the rules and regulations.

Code: In-group efficacy

Participant13

Well, it is again, the general perception as I can hear. I do not know why this belief is there. That there is, a little racist approach is there. Not only the South Asian, even the African origin, the African community who have come and settled here and are second generations, I have seen in tv, even in the British Broadcasting Corporation, these allegations have come up and they were saying, in the support, that the black community has been neglected, and they're not getting that sort of facility like the white, I heard that, but I have no first-hand knowledge or information, but I have not come across any of these in South Asian community saying about this.

Code: Institutional trust\Healthcare access\NHS access is equal

Participant13

Well, it is again, the general perception as I can hear. I do not know why this belief is there. That there is, a little racist approach is there. Not only the South Asian, even the African origin, the African community who have come and settled here and are second generations, I have seen in tv, even in the British Broadcasting Corporation, these allegations have come up and they were saying, in the support, that the black community has been neglected, and they're not getting that sort of facility like the white, I heard that, but I have no first-hand knowledge or information, but I have not come across any of these in South Asian community saying about this. But, you know, this is a historical fact that the British has got a sort of a slightly racist approach, embedded in their system. Not for this coronavirus, even otherwise for, for maybe last 60, 70 years, which is perhaps to some extent that you have to accept and live with this. Why? Look at the human psychology, it is their land, where the immigrants have come. Maybe they needed to run the wheel of development because by themselves they were not sufficient. So the Asian community, the African community and the other communities came in. And they contributed to the development of this country, ok, which the British themselves, they accepted. But in that process, that is also an underlying sort of a ceiling where the white kids get more priority when it comes to a competitiveness. If things are available, then ok, but when it is a choose and take, then some sort of undeclared partiality, unspelt partiality works as an incurrent psychology and the people who are disbursing the facilities.

Code: Institutional trust\Healthcare access\NHS access is equal\In spite of British racism

Participant13

So there's not much point in complaining because in general, my experience is that I've been fairly and squarely treated. Even in India, there is another type of power gap. If you have more money and more influence, then it sounds wonderful. Everywhere, there are some underlying condition, nothing is perfect in this world. That bit of imperfection if it is there, you have to learn to live with.

Code: Institutional trust\Healthcare access\NHS access is equal\In spite of British racism

Participant13

Well, understanding is there, but the attitude is perhaps not cooperating. They understand it very well, but their ability to cooperate is not that good. I think so.

Code: In-group efficacy\Messaging recieved

Participant13

It is because of the indifferent attitude, not that day I have the wilful intention of not cooperating, but, ‘could not care’ attitude, lack of attitude. That is somewhere there.

Researcher (55:19): And that is hindering them to follow that rule here. Mm Hmm.

Participant: I have, I have seen about the mosque- I am not saying about the temples, because they are all closed- because during the Ramadan, I have been there, and I have seen, people don't give a damn about it. I'm not trying to portray a particular community. But the indifferent attitude, that is there.

Code: Drawing differences\religious non compliance\Islamic noncompliance

Participant13

I mean in all places of religion, you have to maintain social distancing. And you know, religion can wait, but your life and death, if it is at risk, why do you take risk? Your life is more important than praying. You can pray from your home, you don't have to go to your place of worship because, God or Allah, everybody are everywhere. It is not only inside the mosque or the temple. Wherever you are, he is around you. It is the lack of understanding, and then, you know, it's herd effect, if everybody is going, why not me?

Code: Drawing differences\religious non compliance

Participant13

It is not the government. It is the local leaders. It is the community leaders. The government can't just go everywhere. You have to trust the community, the local people who are responsible, they should take initiative and they should, if it is happening somewhere, they should go and take initiative and tell the folks that it is not for anybody, but for your own good. So you behave the way it is required, because if you wish to fight, you just go, get contaminated and get finished. So it is not that they don't understand it is just the community people who should tell them.

Code: Community cultural commonalities\The collective nature of SA culture makes distancing more diff\Centrality of community groups in messaging

Participant13

Honestly, I have not come across because of my restrictions, my movement restrictions, I have not come to any community or people who do not understand. I think people are generally well informed. There is not a single individual who do not know by now, what is coronavirus and how harmful it is to the mankind. So the message is already there. It is the question is of habit of implementing.

Code: In-group efficacy\Messaging recieved

Participant13

Well, the South Asian community I know, they have maintained the sort of, uh, distancing. Not only social distancing, they have confined themselves, all the elderly people. I know at least 200 to 300 people and families doing it now, except the younger folks. The elderly are all at home. Yeah. Maintaining distance. They don't go and meet the children and all that. Even their daughter and sons in-laws bring the food, they leave it at the door, and they do all the cleaning and the precaution that is necessary. They are fully aware of it, and they are following it. They are so far, so good!

Code: In-group efficacy

Participant13

You see, in my road, I have a mixed population and my next door neighbour is a young lady..not very young- but in 50 plus. She lives on her own, I have never seen her wearing mask. She goes out by her bike, at least once, and she walks at least three times to the shops. She asks whoever she thinks needs her help. She doesn't, you know, tells me she doesn't tell me anything about these, that I am worried, or I won't go on and all these. And then next to her are people with kids.

Researcher: (30:00) Tell me again, is this lady South Asian?

Participant: No, White. But my next to next door is Sri Lankan and the husband is Scottish. She hardly goes out, I have seen her. She stands on the clapping day at distance, and calls me auntie. She is born here. But she is very very careful. Even when her son's birthday fell in between- I asked her, are you comfortable if I give him any present- he is four years- and she said, auntie, leave it in your house. So I realised that she's really got scared. In our road there is another Bangladeshi lady, elder than me, and she also lost her husband a year ago; she and I used to go together, shopping. Then I don't have a nice garden, She'd got a nice one. So she used to call me almost every day, that come and spend time, have a cup of tea. She stopped doing that, she is also a bit scared. And mostly, all the other I know, they're mostly white people. Where they are from I don't I know. Local, Pakistani or I don't know. Whatever I see in the news I know, that a lot of Pakistanis were effected and when I saw the doctors. But personally, I don't know much.

Code: Contesting "SA Community"\Alternative definitions of community\Local, not SA community

Paricipant12

l the news item I can gather is that a lot of Pakistani, Bangladeshi were affected, there, as I said, the Bangladeshi lady who lives on our road, her son texts me, sometime, when any Bangladeshi died. So, it may be twice than the White people. He is born here and it must be affecting him. He is 50 plus. So every time I've seen, or anybody Bangladeshi dying, he always texts me, that auntie, I read that. So he is worried why it is affecting the Bangladeshi community so much.

Code: In-group efficacy\Messaging recieved\Informal

Paricipant12

I don't know really. Because they have more children, sometimes they're stuck up in their house, but then the one he is sending me, are quite well off one. There quite dig it. It's not, they're living in poverty or anything. No idea, it must be our immune system.

Code: Community cultural commonalities\Physical differences\Difference: immune system

Paricipant12

Because of your food intake and all these, may be different, may be lot less healthy. But then again, this is so confusing. Lot of perfectly healthy people are also dying. And I sometimes think that people in India, compare to the number of people and number of death, in my mind, it comes that because poor country people have lot more immunity, sunshine. They can take lot more than us, all these normal things comes to me for a normal person like me, I don't know entirely.

Code: Community cultural commonalities\Physical differences\Difference: immune system

Paricipant12

When I go out time to time, up to Tooting, then my Pakistani shops are full with people. The Ramadan is going on, I think they don't care. They are going inside- next to that there is a Tesco, they can see that there is a circle to stand, for social distance, blah, blah. But then when you pass those shops, even I'm going to be scared to go in, because they don't care- small shops, with lot of people inside. I don't know whether they are worried or not. They think whatever god does will happen. I also think so, time to time, looking at them- that they let that be with Allah!

Code: Drawing differences\religious non compliance\Islamic noncompliance

Paricipant12

When I go out time to time, up to Tooting, then my Pakistani shops are full with people. The Ramadan is going on, I think they don't care. They are going inside- next to that there is a Tesco, they can see that there is a circle to stand, for social distance, blah, blah. But then when you pass those shops, even I'm going to be scared to go in, because they don't care- small shops, with lot of people inside. I don't know whether they are worried or not. They think whatever god does will happen. I also think so, time to time, looking at them- that they let that be with Allah!

Code: Drawing differences\Shop noncompliance

Paricipant12

But then when you pass those shops, even I'm going to be scared to go in, because they don't care- small shops, with lot of people inside. I don't know whether they are worried or not. They think whatever god does will happen. I also think so, time to time, looking at them- that they let that be with Allah!

Code: Drawing differences\religious non compliance\Islamic noncompliance\Leave it to Allah

Paricipant12

Asian shops mostly. Near my home there is a Turkish shop, that is 24hrs open. That is it. All I see people are not bothering. Even I complained in the beginning, I'm not going in your shop, and then someone will complain about your shop. After that I noticed, people are waiting outside, but still, they are allowing..you cannot maintain the distancing like that. So either they don't care, or don't believe all these, I don't know. The girl in the till never wear a mask. Originally I went once, and after that I stopped going, when I see how many people inside.

Code: Drawing differences\Shop noncompliance

Paricipant12

Something, only thing that comes to my mind that you know, they live with lots of family members, sometimes. And suppose someone is in the key job..then they have to go isn't it. So they must be some precaution when they come back. Like my GP, a young man, he has to go back and he he's got a 5 years old girl, will be 5 in June. So when I check with his wife, that what is he doing? She said, oh, auntie, you should see when he comes back. Washes his hands.. And blah, blah, blah, many times. But now he is also fed up.

Code: Community cultural commonalities\Multi-generational living

Paricipant12

I don't know, some of them told me that, she lives very far, in Reading. She was mourning that, their doctor completely refusing to see, because she is diabetic and all the other problem. So the only 111 and then she had to wait outside the surgery. But most of them were saying that they were happy.

Code: Institutional trust\Healthcare access\NHS access is equal

Paricipant12

I, don't know really. You know, I never discussed all these, anybody except our personal, when we will see again and how are you. I personally, as I told you, it is such a new thing, nobody in the whole world knows what's going on, but that the way I think they're trying their best.

Code: Institutional trust\Government trust

Paricipant12

I think most of the people do understand the severity. I don't know. Do you know, one friend, she still cannot grasp the whole thing, because she lives with her daughter. Her daughter is a key worker. Her husband is a bus driver. The grandkid goes out to the school for key worker. So she, up to last week, no matter how much I'm telling her that there is no necessity, she's old, 80 almost, to go out, but she goes out.. She changes two buses. One day she appeared in front of my house. I couldn't say anything. I felt bad. I had to make her seat. And then another day when she came, I was walking on the footpath. So I just said, please, please, don't come in. Even my grandkids don't come, my son doesn't come, he would be very annoyed if anything happen. So, And that is the only one I have seen amongst hundreds of people I know. She goes out every day, she goes to the bank. She goes to shopping, even though she doesn't need all this.

Researcher: (50:00) So why do you think, what is the difference?

Participant: I think because she's seeing her household every day, going out.

Researcher: I see, I see.

Participant: That she thinks if everybody in the house can go out, she can can go out. Why I will be bored? I am somebody who is fanatic maybe! So, whether there are any families like that? That I don't know.

Code: In-group efficacy\Messaging recieved

Paricipant12

I think most of the people do understand the severity. I don't know. Do you know, one friend, she still cannot grasp the whole thing, because she lives with her daughter. Her daughter is a key worker. Her husband is a bus driver. The grandkid goes out to the school for key worker. So she, up to last week, no matter how much I'm telling her that there is no necessity, she's old, 80 almost, to go out, but she goes out.. She changes two buses. One day she appeared in front of my house. I couldn't say anything. I felt bad. I had to make her seat. And then another day when she came, I was walking on the footpath. So I just said, please, please, don't come in. Even my grandkids don't come, my son doesn't come, he would be very annoyed if anything happen. So, And that is the only one I have seen amongst hundreds of people I know. She goes out every day, she goes to the bank. She goes to shopping, even though she doesn't need all this.

Researcher: (50:00) So why do you think, what is the difference?

Participant: I think because she's seeing her household every day, going out.

Researcher: I see, I see.

Participant: That she thinks if everybody in the house can go out, she can can go out. Why I will be bored? I am somebody who is fanatic maybe! So, whether there are any families like that? That I don't know.

Code: In-group efficacy\Messaging recieved\Willful disregard of messaging

Paricipant12

Some people cannot do anything. It is like a family, small circle. You cannot send some people, I don't think it will go. They just think, as I said, leave it to god.

Code: Drawing differences\religious non compliance\Islamic noncompliance\Leave it to Allah

Paricipant12

I don't know if they're allowed to go to mosque or not. So they can go to the preacher- to tell them. It's like that. I know the temples are shut, but as I said, mostly, I have seen Muslim community don't really care. Because they are doing iftar shopping. Like yesterday, I went out at seven O'clock thinking that I go for a walk and the streets will be empty. As soon as I went near that vegetable shop and Pakistani mangoes and all these, it was crowded as if it was 12 o'clock.

Code: Drawing differences\religious non compliance\Islamic noncompliance

Paricipant12

I don't know if they're allowed to go to mosque or not. So they can go to the preacher- to tell them. It's like that. I know the temples are shut, but as I said, mostly, I have seen Muslim community don't really care. Because they are doing iftar shopping. Like yesterday, I went out at seven O'clock thinking that I go for a walk and the streets will be empty. As soon as I went near that vegetable shop and Pakistani mangoes and all these, it was crowded as if it was 12 o'clock.

Code: Drawing differences\Shop noncompliance

Paricipant12

Finally, what would you think has helped you and the South Asian community as a whole to cope with the crisis?

Participant: Phone! My life is depending on the phone. They all know, that I don't get up sometimes until 10, but the phones are coming lot earlier.

Researcher: That's very good that has helped you a lot to do carry on with the crisis, right? And if you look to other people around you, South Asians, do you see anything which has helped them to go through the crisis?

Participant: As I say, do you know my near neighbourhood? I only know, like that one Bangladeshi family. But they are all asking each other. Even when I'm going out, or when my son comes or drives. I also ask them, you know, my son is coming, do you need anything heavy? Rice or flour and all these. So I think people are really really helpful. I'm lucky, my both side neighbour, nearly everyday checks on me. Boat. Since my husband no more, maybe that's why. I don't know.

Code: Contesting "SA Community"\Alternative definitions of community\Local, not SA community

Paricipant12

So first of all, I must say I am from Bangladesh, but I actually do not, how can I say, I don't have any contact with the Bangladeshi community as a whole here. So I don't know about how they are responding.

Code: Contesting "SA Community"\resisting division by ethnicity\Doesn't consider self as part of a SA community

Participant11

And actually, I don't have any contact with Bangladeshi community here, so I don't know, I don't know..I know nothing about them. But I know about my in-laws, for example, some of them they're from Bangladesh, but they don't represent the community. Like Bangladeshi community or South Asian community. I actually don't know.

Code: Contesting "SA Community"\resisting division by ethnicity\Doesn't consider self as part of a SA community

Participant11

I think they're more, more, more at risk. Why they are more at risk? First of all, because their lifestyle is like, they don't take..it is very hard for them to ignore the community. Like if somebody comes, because they don't just call on them, have an arrangement to come home, they just come home, just have a visit. And they like both of them, they don't take Covid seriously to prevent it. Generally, kind of, they are a little bit ignorant about it. I have heard about it, it can happen, but they're not very much .. Basically ignorant about it, more of a communal. Okay. That's the one thing. So it's ignorance, first of all. And other thing is like some of the in the community, they just don't, how can I say, whether that is true for here or not, I'm not sure, but I think they have less opportunity or access to have the best of a health system. I don't know actually. I'm not sure about them.

Code: Community cultural commonalities\The collective nature of SA culture makes distancing more diff

Participant11

Like for the GP..I don't know the services. I shouldn't say because I don't know actually. One thing can be a problem, is like, because of the, some of the people that cannot be integrated to the society because they just live in the community, you know, for example. Yeah. So when they go to the hospital or access to the doctors, they cannot communicate properly. They don't know how to, so it could affect them that way.

Code: Institutional trust\Healthcare access

Participant11

Participant: You are talking about the South Asians?

Researcher: South Asian community as a whole.

Participant: Yeah. I have actually no idea.

Researcher: Any specific difficulties you think they may have to follow these instructions or abide by that?

Participant: I have no idea, first of all, but I have no idea, but I think there can be also of course, totally dependent on my guess. Maybe, I don't know, actually, but maybe, as they cannot see the virus, you know, you cannot see there is a virus. So if you cannot see it, it doesn't exist. So there is a little perception on that. So they are, how can I say, so they've taken it less seriously maybe. Maybe I'm not sure. Actually, maybe I don't know. It's actually not for the South Asian community, but it is for whole. Actually there are always some ignorant people. If you cannot see it, it doesn't exist. For example, they can wear a gloves, but they don't know they are wearing gloves for prevention! You wear the gloves, but if you wear the gloves and you touch with the glove, something, touch your mouth touch something, the virus is transmitted there. They don't think in that way. They think, well, I'm a wearing a gloves and I'm fine, I'm protected. So that's kind of, but it's not only the South Asian, it's like, there are always some ignorant people, uneducated in some way.

Code: Drawing differences\Noncompliance due to ignorance

Participant11

I don't think, I don't think, I don't know about the South Asians but they're part of the whole community. So I think that they're not 100% happy about it. They cannot trust hundred percent. There, there are some things that they cannot trust the government hundred percent, that it will be all right.

Researcher: Why do you think so?

Participant: Because of the financial crisis, because those who are actually thinking about the government, those who are effected by the corona, like by their doles and incomes and things like that. And I think as a whole community, they don't think that they have enough from the government. They have been supported enough from the government. I don't think that they think in that way. I don't know if that is possible. I have no idea about it also. I don't know if it is possible for the government to support everyone the way everyone wants, but so far the government has done. I think not anyone like everyone is not happy about it.

Code: Institutional trust\Government trust

Participant11

I think in the South Asian, Bangladeshi community or Pakistani community, uh if there are, for example, one person in the household, you speak of coronavirus, they don't want it to spread. They don't want them to be known by the others. So I think that is a taboo or how can I say no, not taboo, but I think this is a major issue.

Code: Drawing differences\Recent immigrant specific\Fear of stigma

Participant11

Because they think like people will see them differently or if they died out of it, the people will not come for their funerals, or people will see them differently. So because the community always think about others, right? How the people will think of how they'll see us. That is one thing. They will think like, okay, we are affected. So it doesn't matter if somebody else- so they don't want to share the information with them. So they take it more personally rather than. So that can be one issue. And that's why to go to the doctors may be, I think there can be a little bit denial about it. I'm not sure actually, I'm making everything in my mind now.

Code: Drawing differences\Recent immigrant specific\Fear of stigma

Participant11

Yeah. So they can be maybe..denial about it. Maybe if they have a symptom, they don't say like, okay, this is a coronavirus symptom I'm having. And maybe there can be denial about it because they want to hide it actually. So that can be one, one mental block. Other thing is like maybe language issues can be one. They don't take like the ignorance can be, they can have like little information about it. And thing that is normal, like normal, how can I say the cold and flu or because of the misinformation they're having from like information, they can just have tea or like gurgle and then think, oh, we are all cured. They don't have the right information. So that's why they don't go to the doctor. They don't take it seriously as it should be.

Code: Drawing differences\Recent immigrant specific\Fear of stigma

Participant11

I think the messages will be for everyone. It should be like, you have to, I'm actually very much, you have to take the right information from the doctors, not from others. And also like for example, in a temple or in, in, in a church or what is that called? The mosques or the priest and all of them. They all have to be trained to tell people you have to go to the doctors. And one doctor says, because they represent the scientific community, you have to listen to them, don't listen to anything else. Don't just drink tea or like ginger tea, just to cure or like that. Listen and rely on the doctors?

Code: Community cultural commonalities\The collective nature of SA culture makes distancing more diff\Centrality of community groups in messaging

Participant11

I think it's going to be delivered...and as I said, it can be delivered because they take very much importance of the community leaders and who are the community leaders? They are the imams at mosques, or because they're rely on them. So those leaders, community leaders, if they convey to the masses, I think they will take it seriously. That is very important. And then of course the children go to the school. This is information, but it will be valid if the parents also say, okay, yeah, the imam said, you know, okay, the priest said, the purohit said- this is the thing, we have to do. So I think the community leaders taking responsibility. Yeah.

Code: Community cultural commonalities\The collective nature of SA culture makes distancing more diff\Centrality of community groups in messaging\messaging through religious groups/leaders

Participant11

In this country, you can see what they're doing. So that's why it's not that no one is ignoring. But I have a feeling that some corners like Islam and others, they believe more in Allah. But again, rather, I am not talking from any experience I'm talking from feeling.

Researcher: Go on, you're allowed!

Participant: And that's what it is you know! If Allah is there to do such a thing, who we are, but the human beings. But in here, mosques, people are still going!

Researcher: Okay. So you think that they are still visiting the mosques?

Participant: Yeah. It's only 10% to 20% as such you know!

Researcher: You think that is affecting the people?

Participant: Not really because they are some sort of immune.

Code: Drawing differences\religious non compliance\Islamic noncompliance

Participant10

Researcher: And do you see any health conditions for which Asian community maybe more at risk?

Participant: I mean, generally my mixing up is only with Bengali. So I can't give any really reasonable experience about them as such.

Researcher: Right, right. And how do you think that South Asian community has been specifically affected by the coronavirus?

Participant: I mean, they are separate example. Bangladeshi community is so difficult to come out with results or anything, they are very much secretive about it. So you won't get the full picture!

Researcher: So they are secretive about what, for example?

Participant: Whatever happens they are secretive about it. Because this is about Allah, Allah..

Researcher: So it's the act of God or something?

Participant: Yeah. I don't have that much of knowledge about them. I know about the Gujrati, a little bit Gujrati, but they are well advance. Gujaratis are very very law abiding, this and that. I have some Gujrati friends, we talk over telephones, we always observe the bindings and regulations and rules, what is there.

Code: Contesting "SA Community"\Heterogeneity of the SA community: like other groups\Response split by home nation

Participant10

The problem in this country is that after staying so many years and after being British citizen after all these thing, and loss of our own thing, gone. So we are much more western and we are much more logical, we are much more educated. And when we came here, 50 years, 60 years ago, so that particular thing about Asian community is not that much applicable to us. I have a reservation about the Bangladeshi a little bit because they own all these sort of Bengali restaurant, Bengali chains as such. They are not still as rich like on the Punjabi, Gujrati and other. And that's why they are also not spread, their outlook has not changed that far. It changed, but not to that extent. For Gujrati, there's not much of a difference of opinion- particularly in Punjabi, Gujrati and English. In case of Punjabi, no Allah etc.! Regarding the shop and other in this country, we have to very very particular, because of the inspection and that.

Researcher: So do you think there is a role of religion in it, anywhere?

Participant( 30:00) And it's a way of life for the people. It happens for the Muslims as well. If you take the religion out of it, the business community, which do all this 'sukti-mach' (translation- small business) business, this and that. Does not matter which community they are or which religion they are from, they are all the same.

A Bangladeshi accountant or a Bangladeshi lawyer, who are as good as an English lawyer or a Bengali lawyer or a Gujrati lawyer as such but as you go down, at the bottom area, there are a lot of things. And after staying here for that long, you picked up lots and lots of habit. Though they are not staying, 10 people under the same roof and that sort of thing, those early days are gone. Financially, we have improved ourselves quite a lot. Son of a Bengali shopkeeper, they will become a doctor or a barrister, or lawyer, and that sort of thing you know. So lot of them are going up, the social ladder you can call it.

Researcher: So do you think the community was not living in the same way when you came in, in the beginning?

Participant: Improved quite a lot. The thing is that, that generation which came here, they said we came here, whatever it is, but we needed money; not only from India, but from Bangladesh as well. But after that need has gone, after 50 years, like their sons, the next generation, they don't need the money. It automatically came to them through the process of business or anything, still the percentage, which is much lower that what used to be, and the ways have improved as well.

Code: Contesting "SA Community"\Heterogeneity of the SA community: like other groups\Recent v established immigrants\Prosperity and assimilation means less difference

Participant10

Researcher: And that, do you think, have any relation with how you look at the coronavirus?

Participant: I think we do. They have improved as well. Because ten people under one roof and that sort of thing and infection, it's not there anymore. Not to that extent. They have improved quite a lot.

Code: Contesting "SA Community"\Heterogeneity of the SA community: like other groups\Recent v established immigrants\Prosperity and assimilation means less difference

Participant10

I don't think so. No way. Because the way of life is not Asian hundred percent anymore. Like religion, going to gurudwara, mandir, masjid and these sort of thing- that part of it is still there. But their daily way of life changed completely. Even if you go to a Muslim shop, like Tooting: Pooja- they are Muslim. But their behaviour, their discipline, neatness is much, much more than what it was before. I can see the improvement over 50 years. Remarkable improvement as such. Because of one generation is changed from the other. One chap has got a motor mechanic, or a repairing shop as such. One of the family members is still there because it's generation's money. Some of them are doctor, lawyer and that sort of thing, teacher. I think things have improved quite a lot.

Code: Contesting "SA Community"\Social support\No difference for SA community

Participant10

I don't think so. No way. Because the way of life is not Asian hundred percent anymore. Like religion, going to gurudwara, mandir, masjid and these sort of thing- that part of it is still there. But their daily way of life changed completely. Even if you go to a Muslim shop, like Tooting: Pooja- they are Muslim. But their behaviour, their discipline, neatness is much, much more than what it was before. I can see the improvement over 50 years. Remarkable improvement as such. Because of one generation is changed from the other. One chap has got a motor mechanic, or a repairing shop as such. One of the family members is still there because it's generation's money. Some of them are doctor, lawyer and that sort of thing, teacher. I think things have improved quite a lot.

Code: Contesting "SA Community"\Heterogeneity of the SA community: like other groups\Recent v established immigrants\Prosperity and assimilation means less difference

Participant10

It depends on the type of work they do. Like a Bengali shopkeeper- he can't do it from home. But cases of professional or some of the things can be done from home. It would depend upon the type of job we were doing.

Code: Contesting "SA Community"\Heterogeneity of the SA community: like other groups\Issue is type of work

Participant10

Because the other people like three generation or two generation- an old lady of 70 or 80, now we are not all relying on God. We now know we have to help God to help us. So that sort of feeling is there. I went to a Guajarati community, a charity as such. I saw the lady there with trouser and shoe- Guajarati ladies. Because of the winter and cold things, they see it as hygienic. And they say, no, we have to do, we have to change it. The change came gradually, but it's not exactly what it was 50 years ago.

Code: Contesting "SA Community"\Heterogeneity of the SA community: like other groups\Recent v established immigrants\Prosperity and assimilation means less difference

Participant10

now we are not all relying on God. We now know we have to help God to help us. So that sort of feeling is there. I went to a Guajarati community, a charity as such. I saw the lady there with trouser and shoe- Guajarati ladies. Because of the winter and cold things, they see it as hygienic. And they say, no, we have to do, we have to change it. The change came gradually, but it's not exactly what it was 50 years ago.

Code: In-group efficacy\self-responsibility

Participant10

No, no. It is not that 10 people living under one roof- if 5 people catches, the other 5 will catch, it is not like that.

Code: Contesting "SA Community"\Heterogeneity of the SA community: like other groups\Recent v established immigrants\Prosperity and assimilation means less difference

Participant10

Well, there are people who are always sceptical. Because, first of all, party politics comes in. And that's why it cannot be a prudent judgment. So, for somebody like conservative, whatever they do is good. But for labour, whatever they do is bad.

Code: Institutional trust\Government trust

Participant10

Researcher: So what do you think about this current step taken by the Boris government?

Participant: I think he's excellent according to me.

Researcher: And you think the South Asian community may have a similar feeling?

Participant: Of course. Our finance minister, Rishi Shunak, Shunak is Shaunak, you know, meaning the teacher of the rishis (sages). But anyway, he is a Punjabi. He is quite sharp and bright. I listened to the budget he briefed..and he is also very, very sharp.

Code: Institutional trust\Government trust

Participant10

They do now, even not hundred percent. There is a hundred percent improvement since earlier years. Only a little gap, I would say here and there. Some sort of community, they are cautious. Because you can see, an accountant or a lawyer, or a teacher, they can't be other- Indian and English mix could not be other. But in our home we can do the chapatis, we can do this; now the English are doing chapatis as well! They are not Asians or anything. They are enjoying the Asian foods as well, you know.

Code: Contesting "SA Community"\Heterogeneity of the SA community: like other groups\Recent v established immigrants\Prosperity and assimilation means less difference

Participant10

They are doing it according to me, over 80% of them are doing it. Because that thing, the type of people, 50 years ago, well whatever god do, will happen and that sort of thing people doesn't believe anymore. In my younger days, when we had to immunise ourselves, if we are unlucky that would happen to us, injection would not make any difference. But nowadays, they know, they think it makes a difference. In case of flu jab, everybody takes it, irrespective of if they are Asian, European or whatever it is, because the facility is there, they see it and take it. No one leave it to God or Allah, but they take it (jab).

Code: Contesting "SA Community"\Heterogeneity of the SA community: like other groups\Recent v established immigrants\Prosperity and assimilation means less difference

Participant10

According to me, follow what is advised, no advise is there to make any harm to you or something. And by not following you are not doing any good to anybody. Start with yourself, or any others- you are damaging others as well.

Code: In-group efficacy\self-responsibility

Participant10

I think it goes, as I say, 80% -85%, it goes to them as well. First of all, the community would have some sort of people, I say, from here, most of those of type of people are morally disappearing, so that would not happen. The thing is that people whatever South Asian people- they were not like that before. Though we still follow our religion, Hindu, Muslim or anything, but behaviour wise, we still follow the law of the land.

Code: Drawing differences\religious non compliance\law of the land v religious law

Participant10

There is always some unreasonable people. It is not necessary they are illiterate- because there are not much of illiterate people here. But somebody, character as different. But again, that percentage is very, very low

Code: In-group efficacy\Messaging recieved

Participant10

There is always some unreasonable people. It is not necessary they are illiterate- because there are not much of illiterate people here. But somebody, character as different. But again, that percentage is very, very low

Code: In-group efficacy\Messaging recieved\Willful disregard of messaging

Participant10

I think following what is laid down as a rule. Follow it religiously. You keep them above the religion, and you can follow the religion after.

Code: Drawing differences\religious non compliance\law of the land v religious law

Participant10

Yeah, for the people living here, most of the Asians, they are..they are.. they, they, they always aim to in their life for some reason, they like to be a doctor. So most of the Asians, in their careers want to become doctors and they achieved it. But even though they had done it, they are the one in the front, and they are the one, they have to suffer now. They are not given proper PPE. So poor children who studied, worked hard, and they're trying to help, but the government should help them with giving PPE and the oversight was new ones. They make the foray to go up to the front. So they have, they are forced to, in the front and get the disease. So many educated foreign people have died. So that's the loss.

Code: Community cultural commonalities\Employment\SA being "sent to the front"

Participant09

Um, that means, if you going to deal with a patient, they are sending the- that was in the news- sending the foreigner doctors to deal with that, the patients. So they are the one who are getting the contact. Even if this is correct or wrong, most of the NHS people are foreigners..Asians. So they are the one who are trying to fight the corona patients, and are the one who are affected. So that is a bit sad. Yeah.

Code: Community cultural commonalities\Employment\SA being "sent to the front"

Participant09

Mostly, Asian people like to study, they like to help, so they choose their career as doctors. Being a doctor is a difficult job. You have to be really caring for others. Because they like to do that and study, and then become doctors. And it's fine. If they give these PPE, then it's fine. Then dealing with the patients. But they are provided. So for the poor families...

Code: Community cultural commonalities\Employment\SA being "sent to the front"

Participant09

Yes. And the chances are of more affected, because they live in extended families. They live together. The parents live together, in some cultures, the children look after the parents. The children have to travel, so when the children come home, they're mixing with the parents who are elderly. So that affects the parents as well. Because that is their culture. But when you take Western culture, they are on their own. And also, they're on their own, they are also struggling nobody to help. So whether you are Western or you are Asian, it is the same problem, but here they are really always worried that they shouldn't be going near their bed. And at this pandemic, that is an advantage, for the family living together.

Code: Community cultural commonalities\Multi-generational living

Participant09

Luckily, we have good neighbours, they are helping. But if nobody were there to help, then you had it! And also with the shopping, also for first three weeks, we couldn't get any shopping. Only the neighbours bought it for us. So good. Otherwise we would have been struggling as well, but it really affected. Yeah.

Code: Contesting "SA Community"\Social support\support of neighbours

Participant09

Um, I don't think so because it fits for everybody. So everybody's going through the same with the government regulations that it doesn't affect them differently. Only thing is when they are in a group, in the same house, they are affected. The elderly are worried, the children are worried. Otherwise, same rule applies to everybody. So these days, you know, South Asian, Western or British or English or whoever is all the same. Yeah.

Code: Contesting "SA Community"\Social support\No difference for SA community

Participant09

Um, I don't think so because it fits for everybody. So everybody's going through the same with the government regulations that it doesn't affect them differently. Only thing is when they are in a group, in the same house, they are affected. The elderly are worried, the children are worried. Otherwise, same rule applies to everybody. So these days, you know, South Asian, Western or British or English or whoever is all the same. Yeah.

Code: Community cultural commonalities\Multi-generational living

Participant09

I think they all feel the same. Everybody, the struggle they go through is same, only apart from these NHS people and their families, their families would be sad because they are not getting the proper protective clothing to do their jobs. Apart from that everybody is in the same boat, everybody's worry is the same. It's not particularly any different to the South Asian people. Probably one might say, if we want to visit to our relatives or some people who live alone at home and they cannot go, so that is boring for them. But the people who don't have anybody, then it is the same, but for families who have parents, and the parents are not well or so, they can't go to visit them, to see them. So that is sad. That is the only difference between the people who live here permanently and people who have relatives abroad. Yeah.

Code: Contesting "SA Community"\Social support\No difference for SA community

Participant09

The health care is here. They didn't want anybody to go to hospital all these days. So people were worried and frightened and now the NHS says, oh, you can come to hospital, come and get this done, get that done. But I don't think people feel comfortable to go. in my case, I would rather stay with the problem, than going to the hospitals and getting more problems. That's how people would feel. For me, that's what I would think. I wouldn't go. Even if I have a full thing or anything, I would rather stay home and recover rather than going to the hospital. Because at this moment, if you go to hospital, you don't know what you are into.

Code: Institutional trust\Healthcare access\NHS access is equal

Participant09

don't think so. Because they, you know, NHS, they don't have any discrimination actually having go to the GP. They said that, I don't think so. They are suffering in that part. I wouldn't say that. Yeah. Yeah. Everybody has the same equal opportunity to access. Yeah. But people are worried. People wouldn't go. That's the thing.

Code: Institutional trust\Healthcare access\NHS access is equal

Participant09

I would say, it is all the same! The government doesn't purposely discriminate the South Asian community, everybody's affected in the same way. Only what is not right is for the people who are working with the patients. Most of them are Asians and they, they are not giving the PPE because government doesn't have it either, but they should somehow, should I get hold of it? That is so worrying. I don't think they deliberately discriminate South Asians.

Code: Institutional trust\Government trust

Participant09

I think they are aware! Nowadays there's a lot of media, all these WhatsApp and all these face time, then communicate to anybody through the network. They will find out. And everybody has an understanding of what's going on and the how much it's affecting people. Everybody will know. Because there is communication. But if you take some part, or other, like South Africa, the communication is not like as in here, then they may not be aware, but here everybody knows everything.

Code: In-group efficacy\Messaging recieved

Participant09

But as I said, I don't think so. Do they need to be told more than what they are telling? everybody has access to media. Everybody has television, everybody can speak English. Everybody can understand that they don't have difficulty specifically to put the message across. Yeah. Because of media is bad and they're trying their best everybody to understand. So yeah. I wouldn't think that they are deprived of getting any message.

Code: In-group efficacy\Messaging recieved

Participant09

I don't think, anybody got any help- apart from having a good neighbourhood people like us! If people are in strange places where they don't have anybody, then, then they will suffer a lot. So it depends on which location you are staying and how much help you are getting from your neighbours and friends, it depend on that. But personally, for us, yeah, because we have neighbours even to get the medicine or shopping. For first three weeks we couldn't get anything. The neighbours helped us; friends helped us. So that was ok. It depends on the people, where they are isolated.

Code: Contesting "SA Community"\Social support\support of neighbours

Participant09

Probably they have close families; the family members might help each other. Because with Asian community, normally they, they have a quite good bonding. They care get about their friends or families. They can look after it. They have some caring towards each other; friends, they would help each other. That's how I think they would somehow get help.

Code: Contesting "SA Community"\Social support\Family\Close family, friends

Participant09

It is a coincidence that three weeks ago, I had a blood test- maybe 1st of April. Then I spoke to my doctor. He said, there is no problem with your blood, all absolutely fine-but there is only one problem. The glucose content is high comparatively: some 6.3. He said, this is a border case, you have to be very active. And this is the case of most South Asians. The glucose content becomes high. And that is their glucose level goes high- They lack physical activity. Physical activity for South Asians are very low. They not taking part in sports or any other thing. That is a very important aspect, which is missing in our lives.

Code: Community cultural commonalities\diet and exercise\SA community is less active

Participant07

Much less! Not less, but mostly they are not very active. And that is the problem. We've got the same problems in Kolkata. If you go to Kolkata there are 100s of people standing in front of medical shop. People are buying medicines! People need some vigorous activity, like cycling, or maybe walking, or running. Lot of people are running, I go to the streets, I find those people who were not running 2 months ago, they have started running now. So vigorous exercise is very important. It gets your glucose level down.

Code: Community cultural commonalities\diet and exercise\SA community is less active

Participant07

Do you think, that, the South Asian people are more or less at risk of this virus?

Participant: More at risk.

Researcher: And that, that reason would be because?

Participant: Because we less of this physical activity, I won't comment on the food, but the physical activity, most, really.

Code: Community cultural commonalities\diet and exercise\SA community is less active

Participant07

I had spoken to my doctor. Previously I had attended a course by National Health Service. The day I retired, they called me for some seminar and there the scientist told me that the South Asian people have high glucose level, because of the physical inactivity. Normally it should be a 10 thousand steps a day, that is not a tall order. Even 6000 or 7000 steps a day would be a good exposure to start with. Or some kind of swimming or, uh, maybe cycling. I see in our road, he goes cycling every day. His name is Mr. R. It is not only shopping, he walks down to the shop. He does cycling, uphill cycling in the morning. He goes to the Lloyds park tennis ground, he plays there. He's 80 plus. And he's upright, straight. So physical activity is at the central idea

Code: Community cultural commonalities\diet and exercise\SA community is less active

Participant07

I don't know whether this, how much the south Asian community are affected, but the, the news that is coming, a lot of south Asians have passed away. All of them having underlying health conditions. I don't know what underlying conditions that had, in the South Asian community. One doctor complained about the PPE, I don’t know whether it is PPE or some other factor. But a lot of people in the south Asian community have passed away. The proportion of people in South Asian communities are quite high. So we have to be very careful.

Code: Community cultural commonalities\Physical differences\Underlying diseases

Participant07

This very difficult to say, but economic aspects I don't know, but economic aspects are important, but maybe South Asians do not the follow the rules. For instance, Ramadan is here, a lot of people are people mixing with one another. I do not know why religious requirement be so stringent. They should have been more careful, during the Ramadan period. They are mixing now, during Ramadan.

Code: Drawing differences\religious non compliance\Islamic noncompliance

Participant07

Participant: I think they are more affected than the white British.

Researcher: why do you say so?

Participant: Because I think the work they are doing at national health, particularly, they they are more exposed? I can't tell the complete thing on that, but a lot of south Asians who are have passed away. Doctors have also passed away, some 16 or 17 of them, I heard the other day. Doctors are fully knowledgeable. How they are passing away I do not know. They are not very old, but something to do with the gene, maybe genes. The way our body is constructed that can happen. We have to study what kind of DNAs we have. The Chinese. The Chinese are not affected. The Chinese are better than us.

Code: Community cultural commonalities\Employment

Participant07

South Asian communities are mainly following the rules. But mainly, some part of the South Asian community are not following the rules, like Ramadan and Islam. But generally, South Asians are following the rules. But certain community is not necessarily following the rules. Not following the rules because of religious requirement. They think that religion requirement is higher than anything else. This is a matter of debate that whether religion should come higher than other social or scientific requirement? I do not know. There is no scientific evidence that if you mix freely, this can be transferred at a faster rate.

Code: Drawing differences\religious non compliance\Islamic noncompliance

Participant07

I think there is no difficulty as such to abide by the requirements of the government, because anything, all the basic necessities, you can get from the shops, the local shops. There are some shops, like local Turkish shop, or maybe the Asian shops, they are not exactly following the same rules. But our local butcher or the local fruit shop, they are all following all the distancing rules. But the other grocery shops are not following the same rules.

Code: Contesting "SA Community"\Social support\No difference for SA community

Participant07

I think so. Yeah. There are some constraints I think everybody's faces because if you want doctor's appointment. That if you wanted a doctor appointment, you have to go to Mayday hospital. There are some constraints everybody's facing. I don’t think there is any specific technical difficulty in the South Asian community.

Code: Institutional trust\Healthcare access\NHS access is equal

Participant07

:In general everybody trusts the government, their decisions and directives.

Code: Institutional trust\Government trust

Participant07

I don't think they fully understand the health messages that are given, either by the government, or the doctors, I think they have not fully understood them.

Researcher: That transparency is not there you think, reaching the community?

Participant: Yeah. It is not reaching the community fully. They think that, no, no, this is not big. We can do whatever we like. So that maybe some, misunderstanding. People don't take it very seriously.

Code: In-group efficacy\Messaging recieved\Willful disregard of messaging

Participant07

Because this is a cultural issue, because... If you go to India, any toilet, public toilet, you got to wash your hands, but you cannot find a soap there. Toilets are not cleaned properly. Lot of toilet which are used are not cleaned properly. So the public is not much aware of the cleanliness in general. That has permeated to the society.

Code: Community cultural commonalities\Cultural difference in health education

Participant07

Because this is a cultural issue, because... If you go to India, any toilet, public toilet, you got to wash your hands, but you cannot find a soap there. Toilets are not cleaned properly. Lot of toilet which are used are not cleaned properly. So the public is not much aware of the cleanliness in general. That has permeated to the society.

Code: Drawing differences\Noncompliance due to ignorance

Participant07

For language. I think that everybody understands what is going on. some people think that oh, this is that we don't have to take seriously. but only when some families get fatalities, then only they understand, what is the consequence of this virus. I don't think full understanding has still come, and are recognised.

Code: Drawing differences\Noncompliance due to ignorance

Participant07

For language. I think that everybody understands what is going on. some people think that oh, this is that we don't have to take seriously. but only when some families get fatalities, then only they understand, what is the consequence of this virus. I don't think full understanding has still come, and are recognised.

Code: Drawing differences\Recent immigrant specific\English fluency\Language

Participant07

For language. I think that everybody understands what is going on. some people think that oh, this is that we don't have to take seriously. but only when some families get fatalities, then only they understand, what is the consequence of this virus. I don't think full understanding has still come, and are recognised.

Code: In-group efficacy\Messaging recieved\Willful disregard of messaging

Participant07

Yes, there is a communal feeling everywhere; there is a local community here. They come out every Thursday, and they are clapping for NHS. And that is where we are in the same boat, all of us, to ride the storm. We are in the community, local community, everybody is helping each other, and I can see a lot of people are getting food also from some supplier. People are getting food for those who cannot prepare food, or have other sources of food. People in general are very helpful. If we do get on top of whatever the problem is, a lot of people are asking, my neighbours are asking, do you need a help? Every time they pass by our driveway they are asking, do we need any help? Everybody ready to come and help us because we are older than the general population. I don’t have to ask, they come and ask us, are you keeping fine?

Code: Contesting "SA Community"\Social support\community help

Participant07

I do not know about the South Asian community, it might be there. But my local community here, they come from all around the world. They are always talking to each other, helping each other. That is why I like, that I find a bigger family. So if we have any problem, they can come and help us. Only our neighbour is not very helpful. But he is Chinese (laughs). If few leaves fall from our tree, our bush to his area..I tell my grandson, when they are misbehaving, I will tell that to my neighbour. They are not very friendly. Otherwise, our whole area, people are very friendly. On Thursdays, they all come out in the street and clap. You see South Asian community world over. More than 50 percent are non-English. Everybody are trying to help us.

Code: Contesting "SA Community"\Social support\community help

Participant07

No one is here to understand that this virus is going to come and go, so we have to be more physically active. Make ourselves fitter and leaner. I Think that is how we can sustain the virus. Eating meat is not very helpful. In Ramadan, during iftar, they eat a lot of meat, that is not helpful.

Code: In-group efficacy\self-responsibility\Increased diet and exercise management

Participant07

I think the most important thing is the diet. Yeah. I saw most of them suffer from diabetes, high blood pressure. Lack of exercise. I used to go I see lots of Asian 'kakas' (uncles), elderly, you know. What they do is, I mean, they go to the gym... They just sit in the jacuzzi ...being lazy, in the gym. They just sit there; they just watch other people (laughs).

Code: Community cultural commonalities\diet and exercise

Participant06

I think, lack of exercise, diet, lot of eating, many factors!

Code: Community cultural commonalities\diet and exercise

Participant06

Oh, that's right. Yeah. You know, my understanding is there shouldn't be any difference between other people. Yes. Everybody here is same.

Code: Contesting "SA Community"\Social support\No difference for SA community

Participant06

In a way that I think, me and my husband are ok with this. There is not much difference than before. But with my kids with my son, you know, he very workaholic also. So he I think it's kind of affected them, more than us. My daughter, she, she can't work from home, so, that way, it has affected her. She could not go to the class and she was just happy for a couple of weeks to have her classroom closed. But my son things to the type of person he is, he is so used to be working with people.

Code: Contesting "SA Community"\Heterogeneity of the SA community: like other groups\Issue is type of work

Participant06

I said, it’s even better because, we can talk to our son every day on Facetime, we've been saving that!

Code: Contesting "SA Community"\Social support\Family

Participant06

Better than before, when he would ring us. Before he was like, you know, when he got a chance, like, hi mom, hi dad. Now. Yeah. Spending more time on the phone, you know.. Yeah.

Code: Contesting "SA Community"\Social support\Family

Participant06

No, I cannot compare!

Code: Contesting "SA Community"\Social support\No difference for SA community

Participant06

Yeah. It says, you know, the government, so as they say, there are black family, white family..! They do not have to say that!

Code: Contesting "SA Community"\resisting division by ethnicity

Participant06

Yeah. It says, you know, the government, so as they say, there are black family, white family..! They do not have to say that!

Code: In-group efficacy\Noncompliance, same as wider population

Participant06

It says, you know, the government, so as they say, there are black family, white family..! They do not have to say that!

Code: Contesting "SA Community"\Social support\No difference for SA community

Participant06

I don't know, maybe our Asian people, because we are more like family people, we tend to be spending more time with the family, but maybe that's one way of affecting the South Asian.

Code: Community cultural commonalities\The collective nature of SA culture makes distancing more diff\SA community is more social

Participant06

But the white people, they mostly live with themselves. They are not out and open. But we are. Yeah. Yeah. Very much people, people's person. So that's me. Yeah. Maybe kind of..

Code: Community cultural commonalities\The collective nature of SA culture makes distancing more diff\SA community is more social

Participant06

Participant: Some are very ignorant, some are not following the rules. So I would say 10% are following the rules and others are not following the rules. They are ignorant, honestly.

Researcher: Why do you think this difference could be?

Participant: Lack of understanding 'who cares?' that of attitude.

Researcher: So, so you look at, there may be a difference in the attitude?

Participant: Yeah. Yeah. Definitely, it's the attitude. That this is not going to happen to me! Yeah.

Researcher: Denial? So the consciousness is more needed you think?

Participant: Definitely! Yeah, right? Yes. If you look on the street, in the wider population and everywhere!

Code: In-group efficacy\Noncompliance, same as wider population

Participant06

It shouldn't be any different between white and the Asians.

Code: Institutional trust\Healthcare access\NHS access is equal

Participant06

You know when you have to order repeat prescriptions, you can order online. I know some Asian population, and they find it difficult, as they can’t log-on, they can't be in computers, sometimes they can't find the correct options, But they can always use a form from the surgery. And, mind also there, there are some Asian people going into surgery: one of my friend is working in a surgery, and they they, don't open the door. So there's a button to ring them from outside. Um, y have to make an appointment to come in. So that way they are more ignorant, about what they should do and what they can't do.

Code: Drawing differences\Noncompliance due to ignorance

Participant06

So I think because of the language issues, you know, they cannot express what they feel, what they want to say. So they are just following the crowd.

Code: Drawing differences\Recent immigrant specific\English fluency\Language

Participant06

Researcher: So language is the main barrier you think!

Participant: Yes, language is the main barrier.

Code: Contesting "SA Community"\Social support\Family\family support in messaging

Participant06

Researcher: So language is the main barrier you think!

Participant: Yes, language is the main barrier.

Code: Drawing differences\Recent immigrant specific\English fluency\Language

Participant06

I don't know how the mandirs, the gurudwaras, the mosques... I don't know, how they relayed the messages to the, to the public. So, that works one way of relaying the message to the public, and whether they did that, I am not sure, whether they did that or not.

Code: Community cultural commonalities\The collective nature of SA culture makes distancing more diff\Centrality of community groups in messaging\messaging through religious groups/leaders

Participant06

I think the elderly generation, probably got the messages from their children, most of them live with the family, you know. Probably the children within the family are relying messages to the elderly people: what they can do and what they can't do. They are not to go to the shop and the children do the shopping. I know my, one of my cousin has a relative, she lives on her own, she is in her 80s. Her children told her she go cannot go around, she cannot go to the grocery shop- they arranged shopping to be dropped off. Yeah. So I think that, you know, children are messaging the parents. Yes.

Code: Contesting "SA Community"\Social support\Family\family support in messaging

Participant06

But then they must have, they must have information- why they were closing down. They wrote them down. I know some of the mandir, which closed down, one week before the lockdown, because they had to protect the elderly population. On Mondays and Wednesdays, there was a play group, mostly, with people which was quite regular, with young people, and they were worried about the elder generation. And I think therefore they shut down before the lockdown. They must've been informed about the lockdown- that this is happening, to make them aware.

Code: Community cultural commonalities\The collective nature of SA culture makes distancing more diff\Centrality of community groups in messaging\messaging through religious groups/leaders

Participant06

Yeah. so messaging, you know, what you see in the news. We see, everywhere, in the news, in the radio! For the elderly, I think they just watch the news on the television, and listen to the radio. You know, not many people have a mobile phone, it is mainly for the children. The newspapers? I dont know!

Code: Contesting "SA Community"\Heterogeneity of the SA community: like other groups\Generational divisions

Participant06

Yeah. so messaging, you know, what you see in the news. We see, everywhere, in the news, in the radio! For the elderly, I think they just watch the news on the television, and listen to the radio. You know, not many people have a mobile phone, it is mainly for the children. The newspapers? I dont know!

Code: Drawing differences\Generational difference in media

Participant06

So I, as an elderly generation probably, when the lock down is over, we will start visiting the temples, that is their way of dealing with it. Young people will go back to work. You know, the people in the middle...I don't know. Personally for me, I will go back to swimming and gym! That is my normal life. I would then know life has come back to the normal. I think it is more on individual person. For my husband, it is when he is able to travel. But then again, I'm not, I'm not so sure that how safe it would be, so that would be worrying for me about, about struggling.. here are lots and lots of issue. Uh, nice when this big issue is over, there will be little little things we are not thinking about.

Code: Contesting "SA Community"\Heterogeneity of the SA community: like other groups\Generational divisions

Participant06

And I usually say, don't be an Indian in this you know...[it should be fine] (translated), that type of. If you have to do something, do something, don't be silly about it. Do you know what I mean? Be responsible. Yeah.

Code: In-group efficacy\self-responsibility\Don't be an Indian in this (?)

Participant05

I think, are you talking about South Asians community in UK or globally

Code: Contesting "SA Community"\Alternative definitions of community\Home country as part of the community

Participant05

For example, many South Asian communities are obese people, compared to a general public, you know, then you've got the underlying conditions like diabetes or blood pressure or whatever. Yeah. Be, uh, uh, Oh yeah. And also things like vitamin D deficiencies.

Code: Community cultural commonalities\Physical differences\Underlying diseases

Participant05

I am not in a position to make a comment. But what I do know is that I comparatively, we suffer more and that's the data that's coming out and I can understand because we've got certain disadvantages in terms of our, immune system because of our underlying conditions, like overcoming the vit D deficiency

Code: Community cultural commonalities\Physical differences\Underlying diseases

Participant05

Maybe, our lifestyle, in terms of the food we eat, etc., but I am not sure about it. We need to accept, but I'm not sure about that

Code: Community cultural commonalities\diet and exercise\Diet

Participant05

But also I think people like me, I've been here for 51 years. So I think our food habits are much better than many people, who've just come from India, maybe five years ago. We don't, we hardly eat fried stuff or things like that.

Code: Contesting "SA Community"\Heterogeneity of the SA community: like other groups\Recent v established immigrants

Participant05

Well, I think our, our food, I am not the person, but our generation's food is much more healthier. Food is much more healthier than people who recently arrived from India, for example. Yeah. Maybe the cooking methodology is better as well. The methodology that the new methodology is cooking in less oil or steam food or whatever, you know.

Code: Contesting "SA Community"\Heterogeneity of the SA community: like other groups\Recent v established immigrants

Participant05

Well, I think our, our food, I am not the person, but our generation's food is much more healthier. Food is much more healthier than people who recently arrived from India, for example. Yeah. Maybe the cooking methodology is better as well. The methodology that the new methodology is cooking in less oil or steam food or whatever, you know.

Code: Community cultural commonalities\diet and exercise\Diet

Participant05

I think to some extent there has been dis-integration of community, partly because of closure of the most of the community, they approach the community centres, you know, I see, for example, temples or community, they have a model of having all these premises, like the Mistris, Patels, Lohanas you know, but all of those places have closed down. Yeah.

Code: Community cultural commonalities\The collective nature of SA culture makes distancing more diff\Centrality of community groups in messaging

Participant05

No, but I think those, I mean, of course I am maintaining the social distancing and that, but cases like this, closed community centres, they play a big role in scaffolding intelligent information, particularly for the elderly population, who don't have a good grasp of, uh, English, you know, who watch media, but may not digest them totally. So the leaders from their class has a role to play, but that has been cut off because the community centres are closed now.

Code: Contesting "SA Community"\Social support\Closure of community-based groups

Participant05

No, but I think those, I mean, of course I am maintaining the social distancing and that, but cases like this, closed community centres, they play a big role in scaffolding intelligent information, particularly for the elderly population, who don't have a good grasp of, uh, English, you know, who watch media, but may not digest them totally. So the leaders from their class has a role to play, but that has been cut off because the community centres are closed now.

Code: Community cultural commonalities\The collective nature of SA culture makes distancing more diff\Centrality of community groups in messaging

Participant05

No, I think we're all in the same boat. And I don't think for the Asian population as such is singled out for have been effected more in terms of profession. Yes. In terms of health, because of underlying conditions, maybe some, but not professionally. I think, that all my son, myself, my daughter we all are all work from home basis, as my neighbours are, another people who are patients, you know, we are, we are treated in the same manner.

Code: Contesting "SA Community"\Social support\No difference for SA community

Participant05

Well, I think some of it is about taking responsibility. For example, my daughter has put a block to me going out altogether. So simple things like that. You've got diabetes, so you're not allowed to go out. Anything you need, you tell me and I'll go and do the shopping.

Code: Contesting "SA Community"\Social support\social support for social distancing

Participant05

You see, we have a son who is in London, he would come to us every month or so, but it's worked out better that now we have a video call every day, before we didn't.

Code: Contesting "SA Community"\Social support\social support for social distancing

Participant05

He would call twice as a week, or three times a week, every other day. But now he's calling, uh, via video call every day. And the nice thing is that he calls in the evening when it's cooking and doing things like that. So we can see that he's actually living a normal life as opposed to a very stressed professional life. You know?

Researcher (42:00):So that's something very positive.

Code: Contesting "SA Community"\Social support\Family

Participant05

He would call twice as a week, or three times a week, every other day. But now he's calling, uh, via video call every day. And the nice thing is that he calls in the evening when it's cooking and doing things like that. So we can see that he's actually living a normal life as opposed to a very stressed professional life. You know?

Researcher (42:00):So that's something very positive.

Code: Contesting "SA Community"\Social support\social support for social distancing

Participant05

Generally? I think very well. There are, there's always a few people. We go for a walk every day because the area I live in, it's not predominantly South Asians, but there are sizable numbers. They are not sort of distancing themselves. They're not thinking that when you're, when you're sort of crossing paths, instead of moving sideways- they're not doing that kind of thing, but they are still wearing masks. They're only coming out in couples instead of 10 people at a time, except for that you know. To be fair, I think they're following, Uh, the government's guidelines fairly, fairly accurately. When I go to the shops also, which, kind of, we do normally, but the other day I had to, I had an occasion to drive my wife to the shop, and when I stopped the car, but I looked out, I was watching people going into the shop and they were actually taking elderly people. They were making the effort, which normally they wouldn't, they would just walk into the shop, making effort to check whether there were many people in the shop or not some of them wearing masks as well. And they weren't gathering and chatting or anything. They were going in the shop, doing the shopping and going back to home you know.

Code: In-group efficacy

Participant05

No, I don't think so. No

Code: Contesting "SA Community"\Social support\No difference for SA community

Participant05

I Mean, it's the same awareness. Just a personal example, nothing. Just to give you an idea. My, my brother's father in law passed away about three weeks ago. He was in a home now here's mother-in-law was in a, in a house and father-in-law's was 85, and she was about the she's about 80, but she was aware and having lived in UK for many years by watching the news, the reason what's happening and whatever. And she didn't tell even her own children to come in during this mourning period, except for her son. So what I'm saying is that even that generation, we may not realize, but they are aware of. I'm saying is that you don't,.. that generation, we may not realize, but they are aware and they're taking that on precautions.

Code: Contesting "SA Community"\Social support\No difference for SA community

Participant05

Well, I, I think that the health care access system is guided by the government in any case. So I know a lot of doctors and doctor friends, and I know from them, that they are doing what everybody else is doing. So I don't personally see, uh, any difference for the South Asian negative or positive, why will it be? For example, I have to order my medicine every month but I don’t do it now because I think there's a new rule which came out about two months ago, I do online prescription. And I'm assuming everybody else has the same level of access and I did not have a problem. They were ready, within three days, they've had it ready.

Code: Institutional trust\Healthcare access\NHS access is equal

Participant05

Things like, for example, the other day, I had a slight blood pressure issue. Notwithstanding that once a year I do my blood pressure measurement. The other day they fixed it. They said, if you have a machine at home, please check, and put it online, I haven't done it yet, but there's an online, uh, reporting system where I can check my blood pressure and give the results to them. I would think they're sending out the same information to everybody else whether they are South Asians or not, you know.

Code: Institutional trust\Healthcare access\NHS access is equal

Participant05

I think they have provisions for them, knowing that they can't. They made me have people calling them who speak the language. I have a sending visit the community nurses. I don't want, I'm just guessing nurses or, or pulling them over to the, on, in single physical bases because we know a friend who works in a surgery and she'll think that we are not seeing any patients. Everything is on telephone on video. Well, we do need to see people. We invite them, we take them to the back door. We meet them there that they've got their process in place, you know. I am sure that’s not going not be any different to anybody else. I must make a comment to this effect. And maybe it's not relevant to you in this country on the whole in England. I think we're very fortunate, but I don't think we treat people differently. I'm not saying it's a hundred percent full proof. I'm sure there is inequality, racism, whether that's a colour bar or gender racism or a relative issue, I'm sure it's there somewhere. But when it comes to government type of things, state, I think on the whole it's fairly even and equal. But the private sector may be a different story.

Code: Institutional trust\Healthcare access\NHS access is equal

Participant05

I think there's a lot of, lot of trusts. Yeah. There is a lot of trust and people believe in the government, you would get the little bit of dissent entryway, you know, but there is trust. I don't have much respect for the journalists because I think they make their income out of being vocal and sensational. So challenging. It's good to challenge the government, but challenging them on the meeting, it is not the way to do it.

Code: Institutional trust\Government trust

Participant05

I think they have. I'm not sure again, but I think because... Some authorities have actually put up things, uh, in minority languages as well. They are trying their best. Yeah.

Code: Drawing differences\Recent immigrant specific\English fluency\Translated messages

Participant05

Yeah. But again, it depends which generation you're talking about. My generation don't really need it because I, I, there are many people in my generation, who can't even read their own language. Right. So they are better informed in English.

Code: Contesting "SA Community"\Heterogeneity of the SA community: like other groups\Recent v established immigrants

Participant05

Yeah. But again, it depends which generation you're talking about. My generation don't really need it because I, I, there are many people in my generation, who can't even read their own language. Right. So they are better informed in English.

Code: Drawing differences\Recent immigrant specific\English fluency

Participant05

Well in a, in a demographic, uh, Lester, when I lived, there has been some efforts made to reach out to those people by producing material in their own languages, people who speak, you know, for example, if you ring the doctor or the hospital, not the hospital, but the doctor then generally, as far as someone who speaks your language is available. The outreach is acceptable, you know?

Code: Drawing differences\Recent immigrant specific\English fluency\Translated messages

Participant05

I think in some areas, but certainly there's been, and it's not been talked about my there's been the neighbourly support type of things, which we've been very good at. There's been areas where people have actually run out in front of you to the community in terms of food or welfare support or something like that. I don't mean money. I mean, people brought into them except for you though.

Code: Contesting "SA Community"\Social support\social support for social distancing

Participant05

Participant (42:00): When you are talking about the community, are you talking about in the UK context, are you talking about in India context?

Code: Contesting "SA Community"\Alternative definitions of community\Home country as part of the community

Participant03

Participant: Okay. In the UK context, I think there is no differentiation and it's anybody, uh, you know, it's, anybody gets treated, anybody that access to services is everybody's level playing field. But I don't see it being a different experience to. But I think I might have it different it in terms of India context. Yeah. I'll find in India lot of people who are vulnerable, just the sort of, you know, um, access to services predominantly because of infrastructure. I don't think they have the infrastructure to cope. Um, we don't have the, the, the amount of, uh, basic, you know, needs in terms of health services within the country. So I think there's a little, if I'm sure it is already there, it's just that the data's not available to the world. I dread to think what if it, if it hits at the rate it has hit Italy and UK, I think it'd be disastrous.

Code: Contesting "SA Community"\Alternative definitions of community\Home country as part of the community

Participant03

Okay. In the UK context, I think there is no differentiation and it's anybody, uh, you know, it's, anybody gets treated, anybody that access to services is everybody's level playing field. But I don't see it being a different experience to.

Code: Institutional trust\Healthcare access\NHS access is equal

Participant03

Yeah, exactly. And I think they don't, don't have the infrastructure. And at the same time, there is this, the element of social segregation, which is happening in the UK, like the grandchildren and the grandparents, not meeting. Social structures in India will not allow that. You cannot tell grandparents in India that you will not allow your grandchild in the house.

Code: Community cultural commonalities\The collective nature of SA culture makes distancing more diff

Participant03

I mean, I know one case when his mother is in the house and she had to self-isolate because his wife is a medical professional at NHS. And I was really shocked. And I said, why is she is self-isolating in the house? And he said, yeah, unfortunately she's using the kitchen in the, in the, um, in the shed. They had an extra space, that's so cruel, even at home. Okay. She has an Indian grandmother, lived in this country only for less than 10 years I guess. She can't be told to go- And you know, she's probably going through depression in that place.

Code: Community cultural commonalities\The collective nature of SA culture makes distancing more diff

Participant03

I think it would be very, it is different. I'll tell you the reasons why, because from a very early age of, early, um, sort of the social, the social structure is slightly different, um, there is, um, I mean, you don't live in a city. I think the British families do not live in joint families. They live in nuclear families, and they do see their parents; there's a lot of interaction, don’t get me wrong there, but that, that, is in a very controlled manner. Unlike ours, where, you know, in Indian community, where you know, you can door knock and your grandparents can come to your grandchildren anytime of the day in, well, you know, you don't hesitate to do that. So, you can't have those grandparents, you can't have those, you can't stay away from it. Very simple. if my mother was here, I would never tell her to go to care home. You get me. So if it is not, it is not just a normal norm for us to do. So I think that way Indian community will struggle to be able to adjust to the social distancing.

Code: Community cultural commonalities\The collective nature of SA culture makes distancing more diff

Participant03

Um, I mean we all go through the phase of fear, we all gone through that sort of element of, you know, finding out, on the grape vine, you know, you will hear, you know, if you take so and so, it will keep your immunity up, all that sort of stuff. So, you know, in times of uncertainty, you believe that, however, intellectually, when you think, you are rather knowledgeable than you are, you will still give in. My husband at went to the pharmacy at our road end, You know, he said, I heard this and I read this, what shall I do? And now he would give him a strip of Vit C tablet, and asked him to take it every day. I said, this is rubbish. This is only vit C tablet- it just keep the immunity and not help with anything else. How much did you pay for it? I paid £16 for it, because it is high level vit C.

Code: In-group efficacy\self-responsibility\Boosting immunity

Participant03

It is just fear psychosis.

Code: In-group efficacy\self-responsibility\Boosting immunity

Participant03

I think it is generally non-British people. I think it is generally related to non-British. Because the British community, predominantly is good at articulation, they articulate themselves, they know how to present themselves. They know how to...they have different faces. You meet the same man in a pub, he's very different. You meet the same man across a, a board room table, he's very different. You know, all that, the same person can suit to change his personality. But we have not been groomed in that way.

Code: Community cultural commonalities\Employment\Workplace cultural disadvantage

Participant03

I think that Asian community or the Indians per se, I finding all sorts of platforms straight to interact. It does social distancing. I mean, you know that the three of us (friends) live in a line, we've literally, you know, next door to each other. I Have not seen my neighbours in eight weeks.

Code: Contesting "SA Community"\Social support\Alternatives to normal socialising

Participant03

I think, I think, um, I think access, generally access to healthcare is yes. Obviously the system is now so blocked, whether you will actually get access to a different sort of, it is completely different ball game. I think general access, health care, as I said again, I caveat this because the people I sort of interacted sort of, you know, the young immigrant population, immigrant population, Indian population. So everybody's, um, equal rights to this country. Everybody has equal access, which is, which is fine.

Code: Institutional trust\Healthcare access\NHS access is equal

Participant03

I don't think, I think the whole of the UK, uh, sort of feels, um, South Asian community per se. I don't know much about their views in general, as I earlier said, but in general, I feel that there has been a slippage and I don't think the South Asian community will deny that. And, um, yeah, I think it's a general consensus across the country, there are slippage problems.

Code: Contesting "SA Community"\Social support\No difference for SA community

Participant03

I think the biggest, the biggest problem that, um, South Asian community per se faces is, uh, is language. And I think a lot of those that messaging or media, um, messaging was done in different languages or translated to different communities. Uh, it will help

Code: Drawing differences\Recent immigrant specific\English fluency\Language

Participant03

As I said again, I don't have any direct, direct communication with the, with the people, you know, Asians or South Asians, and sort of all. But we do projects in, we do have a lot of South Asian communities like East London and where we have to interact with people.

Code: Contesting "SA Community"\Alternative definitions of community\SA community as particular neighborhoods

Participant03

As I said again, I don't have any direct, direct communication with the, with the people, you know, Asians or South Asians, and sort of all. But we do projects in, we do have a lot of South Asian communities like East London and where we have to interact with people. And the best way I have noticed as a designer, as a, as an urban planner now, uh, one of the biggest challenges you face, it is not only with the South Asian community but any non-British community is language and being able to actually go down to them, to them rather than them having to come to you. So the system has to be taken to them. So you see what I mean?

Code: Drawing differences\Recent immigrant specific\English fluency\Language

Participant03

when we took it to the community, the feedback we got from the community was appalling. And we did probably get even five or 7% of the people come and speak to us. We went back to the drawing board and discovered we had to tear it all up because we were looking at a utopian project. Okay, which were completely irrelevant on the ground.

Participants (60:55):The reason being, we were talking to a community that didn't understand English. you got me, you're talking to interact with these persons- I think majority of people in the area were Bangladeshis, you were talking to these kinds of people, who were so scared to come out of their doors, and come to see other people. I Mean, I remember I was not even a very senior at that time. And I, uh, as a, uh, senior designer, I was asked to move on to the project simply because I could speak Hindi and they, and obviously I did not understand Bengali, but neither did I understand Urdu

Code: Drawing differences\Recent immigrant specific\English fluency\Language

Participant03

Exactly. We literally went street to street, 4000 houses. I just gave you this example to tell you how I think people do face this kind of, uh, uh, barriers. Uh, it just that you don't realize it because I understand we are different, basically English speaking migrant community.

Code: Drawing differences\Recent immigrant specific\English fluency

Participant03

One example I was giving you, the different, the different, uh, what do you call it? Uh, uh, different generations in one household. And the very fact that all these generations with the vulnerable generation within the household, how are they coping with it is, is, is very, very frankly, do we think about what we are in the country with a law saying that you're not supposed to have vulnerable people around you. That's a very big issue.

Code: Community cultural commonalities\Multi-generational living

Participant03

And the second thing, is that, of course, how, um, language, as I said, I know people who cannot pick up the phone and talk to the NHS. Why? Because it's not that we cannot do that, but the community is tongue-tied. I cannot just call 111 and ask can you talk to me in Bengali or can you talk to me in Urdu. It is not that you don’t want to do it, but the community is tongue tied.

Code: Drawing differences\Recent immigrant specific\English fluency\Language

Participant03

Same problem you face in India, same problems. Apart from that my connect with them as much, as I said I am on the planning side of things, but, uh, I'm sure that social issues are just the same because the community, the mindset of the community are same, depends on their living style.

Code: Contesting "SA Community"\Heterogeneity of the SA community: like other groups\Progressive/traditional split

Participant03

I think, as the, sort of the educated, um, forward thinking segment or the Indian community, I would put it as saying, um, we've just got to be positive, just think in the interest of everyone, just think not only in the interest of yourself, but your family, your community, the people around you, I think with everybody's struggling and everybody is going through this new norm.

Code: Contesting "SA Community"\Heterogeneity of the SA community: like other groups\Progressive/traditional split

Participant03

Participant: To start with, you know, I will just be clear on one thing, when, when I say South Asian community, my perspective of that two: one is the first generation South Asian community based in UK. Because, because, you know, we, we have not been in touch very closely, you know, community of South Asian who has been here for decades. So that's one context to what, what I'll be speaking about. And second context is because we have come here and first generation, we have not come here for decades here. We have come here more recently. Like, you know, our context of understanding also relates with how we feel the impact of that happening in those South Asian countries like India, Pakistan, Bangladesh, in terms our friends and family. There can be a joint context of, what I bring in here. When I say something, it will be two contexts together.

Code: Contesting "SA Community"\Heterogeneity of the SA community: like other groups\Recent v established immigrants

Participant02

Now, from this context, I feel, you know, people here, health wise, luckily we have, where I deal with the people here, you know, I have a lot of few people in the group who are very prone to, you know, taking care of good health. So, so we actually have, for example, created a health group for our own close community.

Researcher (4:33):When you mean people, you mean people from South Asian community?

Participant: Yeah. But I'm talking about some close family friends, or the bubble, with whom we talk about, with whom we talk and mix lot more than other people. But in that bubble, you know, there are quite a lot of people who are very much conscious about their health. For example, we also have an NHS doctor in that group. You have lot of people who, who have their own gym in the house. For example, we have, my own wife, who will basically exercise regularly.

Participant: I think for, so luckily, you know, we have those sort of people. So we have created a group where we kind of, you know, create different sort of challenge and create each other to, you know, I support each other in some form of health activity each day. Which is keeping us, you know, fit. So that's, that's one thing, you know, and secondly, I think food wise also, we if you see the South Asian community, traditionally they have, they have like eating a lot of, you know, food with spices and you know food which needs a bit more cooking and stuff like that. Now, I have seen that it happening even in my own house, you know, I'm talking about you know, before. But you know, over the last year or so, you know, things have improved in terms of how we eat the food also. During coronavirus, I could see people are eating quite healthy. So seeing that was a time to take measures, which can increase the immunity. Now, people, you know, they would take you know what could increase their immunity going forward so that they can be protected, if the coronavirus, you know, come, come in there, you know? So, am I giving you the right context?

Code: In-group efficacy\self-responsibility\Increased diet and exercise management

Participant02

Okay. More or less? I think I think age wise they are less vulnerable. At least in UK, because as I said, we have a first generation, lot more are professionals or, you know, in the middle age group. So age wise, you know, that they will have less impact I feel, and of course, you know, there are cases where things can go other way around as well, but back in India, where, again, in another context where I have my parents, my community as well, obviously for them in India, you know, they are trying to cut down you know, some lockdown measures they're taking. But in India, you know, I have, I have my own doubts if it starts, you know, increasing, you know, I feel, you know they will be, there is no similar, you know help, healthcare department like NHS, that you have here, those people, for them to survive, you know, I think it would be tricky. They are not that they're not as known to, you know, taking the right measures in terms of proper exercise, eating the right food. I think that awareness is still not as much as we have here, I feel.

Code: Community cultural commonalities\diet and exercise

Participant02

Okay. More or less? I think I think age wise they are less vulnerable. At least in UK, because as I said, we have a first generation, lot more are professionals or, you know, in the middle age group. So age wise, you know, that they will have less impact I feel, and of course, you know, there are cases where things can go other way around as well, but back in India, where, again, in another context where I have my parents, my community as well, obviously for them in India, you know, they are trying to cut down you know, some lockdown measures they're taking. But in India, you know, I have, I have my own doubts if it starts, you know, increasing, you know, I feel, you know they will be, there is no similar, you know help, healthcare department like NHS, that you have here, those people, for them to survive, you know, I think it would be tricky. They are not that they're not as known to, you know, taking the right measures in terms of proper exercise, eating the right food. I think that awareness is still not as much as we have here, I feel.

Code: Community cultural commonalities\Cultural difference in health education

Participant02

I feel, fundamentally, it is the similarity, because you know, the measures which the UK government is taking in terms of loock down, what that means for your work, what that means for your, you know, your boundaries in terms of your personal and then professional boundaries, you know. Fundamentally, I think it is the same across. But on the other hand, I feel, you know, the people about who I hear, you know, of course, you know, they, they don't have their own close community in UK, as the people who has been born in brought up here would have, would have had in terms of, you know, friends from their university, college, school, or even their, you know, family members, you know, they would have more family members around. Even though they're not meeting them, I think, you know, there’s a perspective for that. There could be a support network they might be having, which we don't have.

Code: Contesting "SA Community"\Heterogeneity of the SA community: like other groups\Recent v established immigrants

Participant02

So so we still rely on, luckily we have this good, good bubble of close friends, but I think in our network, as far as I'm aware, not many people have such a good bubble of, you know, good friends around. And some of the people might be new in UK who might have just come, two-three years back. We are here for 15 years, we know a lot more people. So I think, I think, you know, on a personal level, they could be different in terms of the support mental support you know. Physical support as well in terms of, you know, if you need to, basically, if you get coronavirus, you know, if you need some support in the form of somebody to deliver food for you or, or do certain things, I think that there could be technically some support, but I think mentally, you know, I think there will be lot more people in our, our community who might not feel like that who, who might be new in UK. Luckily again, again, again, you know, you know, in our bubble, we're close families, but if I see the entire community, you know, we are, you know, if I notice, things could be different.

Code: Contesting "SA Community"\Heterogeneity of the SA community: like other groups\Recent v established immigrants

Participant02

I feel that that helped too. I think the possibly the British people I feel has learned the art of, you know, keeping, keeping good health from childhood. It was always part of the curriculum and everything. People in the West conference on the, she, I think they have learned that over time, some people might have had that, but other people have learned that. So I feel that will have some impact depending on who I, in, what part of journey about, you know, their understanding on health, nutrition, exercise, mental health, you know, all those things. So, so that way it can be different than be defended. Yeah.

Code: Community cultural commonalities\Cultural difference in health education

Participant02

And people who are more essentially have come here, you know, might be, there might be stress actually also you know, unlike in UK where, you know, people, I used to work in a slightly different mindset in India, you know, people see work as more like a, you know, as a holy grail sort of thing, in terms of, you know, if you have work, you are safe, you have money coming in, you know, stuff like that. And if you, if you're out of work or, you know, if you're in that situation, that's quite for people from Asia and they have some limitations also, you know.

Code: Community cultural commonalities\Employment\Impact of joblessness

Participant02

That's an interesting question. Because I think generally I have the feeling that, you know, uh, people from South Asian community, they are much more sociable. uh, their context of life and they, they are, you know, uh, they're they're they want to be with people. They are in, you know, with people, they like to be people, their context of life are less individual and more social space, uh, as compared to, you know, the people in the Western world. I think it's not just India might be the case for most of Asian culture. Um, for, from those contexts we see here, I think, you know, people are not used to what UK government have asked them to do. And, uh, I, I don't know how people are taking that. When I was walking I was, uh, you know, outside in one of the local residents, you know, there were some Asians, playing Volleyball! There were like 9-10 people, which was not the UK government asked, you know? And even, even within our bubble and area around, I have seen that people are prone to asking, you know, finding ways to do social based exercise or something like that. But luckily, there are other people, who can manage those inclinations, and if we could, you know, collectively manage. But, uh, I think it's, it's not that easy for people from Asian culture to not meet collectively with other people, uh, for food or for, for talk, or for general, you know, chit-chat. Uh, in fact, we are not used to, you know, not speak with your neighbours, as we see here- where we live is a slightly different world.

Code: Community cultural commonalities\The collective nature of SA culture makes distancing more diff

Participant02

So I think, NHS wise, how you access NHS, that's universal I think. I think that that's something same with everybody. I would imagine. There is a very specific rule in terms of what to do. And, you know, in terms, in terms of isolation, in terms of when to call 111? So I guess, uh, from a, from NHS perspective, there should not be, you know, an issue health wise.

Code: Institutional trust\Healthcare access\NHS access is equal

Participant02

Um, if there's any other factor, uh, which is, outside NHS, in terms of, you know, uh, your community base, understanding of health, or something like that is there, then of course, that's where South Asian community might be missing out. If there are some gender groups, groups for community based health, learning, South Asian people might not be aware of that.

Code: Institutional trust\Healthcare access\NHS access is equal\Access to healthcare

Participant02

I think it should be okay for everyone. That's a good part of NHS, you know, that's how I feel fundamentally NHS is kind of equal and that's what coronavirus is. Coronavirus is equal for everyone, that's how it should be.

Code: Institutional trust\Healthcare access\NHS access is equal

Participant02

There are reports that recently have popped up where it says that the number of deaths in coronavirus has been a lot higher in deprived areas, versus in less deprived areas. And, uh, so, so, so I, I feel that's something we should also be, uh, you know, finding out- I don't know how, but, you know, I think the South Asian community, most of them maybe living in a balanced areas. I wouldn't say they, you know, they would be living in a very deprived areas or they would be living in very effluent areas. But I think a large community people are not part of the affluent group, for the first generation. And, and, and, and because most of the people who, came here are a bit more professionally educated, they will also not be in very deprived areas. They are more on a middle ground areas, in fact, my own area, where I live, I think it would be something around that sort of balanced area. So that's something also to be seen how being in those sort of areas also have might've impacted in terms of, you know, getting the coronavirus. You know, more than, you know, other areas. And if affluent people don't have to do some certain work and go out, you know..

Code: Contesting "SA Community"\Heterogeneity of the SA community: like other groups\Issue is affluent v deprived

Participant02

luckily, you know, many of the South Asian people here, which I said, ah, come from IT department, right. So the IT people still can work from home. Yeah. But, you know, if there are people who are, you know, non IT, and, but there's of course a certain percentage of them as well- non-IT. Uh, so, so they, they, they then go to work and, you know, uh, so, so, so the percentage of those sort of people who, uh, who are, who can't follow the measures of government in terms of, you know, staying at home and stuff like that, you know, is a bit higher here, compared to affluent areas. That the way the South Asian community, will also have an impact.

Code: Contesting "SA Community"\Heterogeneity of the SA community: like other groups\Issue is type of work

Participant02

They can have the virus more. uh, But, but it has, but it has to be seen in, in comparison to their, you know, professional profile at work. So at the same time, a lot more, as I say, Asian people are lot more from IT background who might end up, you know, working from home or staying at home, but all of them are not like that. Yeah.

Code: Contesting "SA Community"\Heterogeneity of the SA community: like other groups\Issue is type of work

Participant02

Yeah, I think, I think, uh, community, uh, do trust, uh, I mean, uh, I feel, uh, uh, with the wave you were in, you know, two, three years back with the Brexit situation where, you know, uh, parliament was shut down, everything was shut down. I think with the current stability of the government and the government taking you know, more measures in terms of updating people, watch what they are doing, bringing more transparency, Downing street, for example, you know,.uh, um, uh, I, I think those are giving people more reassurance in terms of, you know, they are taking measures for them.

Code: Institutional trust\Government trust

Participant02

I feel in my bubble at least have seen a most of the time, if not all, people have understood and followed them in terms of you know, social distancing and staying at home, you know.

Code: In-group efficacy

Participant02

I feel in my bubble at least have seen a most of the time, if not all, people have understood and followed them in terms of you know, social distancing and staying at home, you know.

Code: In-group efficacy\Messaging recieved

Participant02

Community feeling is there, you know, and that has also made them be part of an extended community in UK, which was great, particularly because, you know, there needs to be some measures to mix things up as a community.

Code: Contesting "SA Community"\Widening of community

Participant02

And then of course there will be some exceptions, but exceptions can also be in, you know, outside.

Code: In-group efficacy\Noncompliance, same as wider population

Participant02

This is, uh, this is interesting because I have seen people from some of the other culture, you know, uh, who, who are not used to English language and stuff like that, you know, they are getting impacted with the language and they have asked for certain regional based messages. But I feel, for South Asian community, the, the need of those messaging are far less because, because as I said, most of the people in the first generation have good English, they have an understanding of English and they're generally in a good profile- in terms of their, you know, awareness and knowledge and work profile and everything. So I don't think messaging wise there's much needs to be done, you know, from the South Asian community wise, I feel. But, on the other hand, I would say, um, there are some community who have, who have come here, who are not educated sort of, you know, and there are community, as I said, I have been always speaking about South Asian or first generation, but I know a lot of community, you know, here, uh, obviously I'm not in day to day, you know, in touch with them, but they are here, you know, it was 30, 40 years back in this country. They have still not learnt properly, you know, the right sort of culture, the context in terms of language and you know, of, uh, those sorts of aspects of, you know, uh, uh, ways of making them aware of what's going. So I think for those community and those areas, there are some specific areas where those people live, I think for those people, those areas, there should be some targeted messaging. And in fact, good areas, uh, have more chances of spreading, because those areas are bit more deprived side of areas.

Code: Drawing differences\Recent immigrant specific\English fluency

Participant02

This is, uh, this is interesting because I have seen people from some of the other culture, you know, uh, who, who are not used to English language and stuff like that, you know, they are getting impacted with the language and they have asked for certain regional based messages. But I feel, for South Asian community, the, the need of those messaging are far less because, because as I said, most of the people in the first generation have good English, they have an understanding of English and they're generally in a good profile- in terms of their, you know, awareness and knowledge and work profile and everything. So I don't think messaging wise there's much needs to be done, you know, from the South Asian community wise, I feel. But, on the other hand, I would say, um, there are some community who have, who have come here, who are not educated sort of, you know, and there are community, as I said, I have been always speaking about South Asian or first generation, but I know a lot of community, you know, here, uh, obviously I'm not in day to day, you know, in touch with them, but they are here, you know, it was 30, 40 years back in this country. They have still not learnt properly, you know, the right sort of culture, the context in terms of language and you know, of, uh, those sorts of aspects of, you know, uh, uh, ways of making them aware of what's going. So I think for those community and those areas, there are some specific areas where those people live, I think for those people, those areas, there should be some targeted messaging. And in fact, good areas, uh, have more chances of spreading, because those areas are bit more deprived side of areas.

Code: Drawing differences\Recent immigrant specific\English fluency\Language

Participant02

think, I think that's a very good question you asking? Um, because I, I never thought of that, but if now, if I, you know, if I now try to reflect, I strongly feel they, they, this, this is playing a huge role because, because of the way that culture in India is, people, right from childhood, they have been born and they've been developed with some contextual spirituality in some form. Each family has that context. Of course, that context differs based on where you are born, which part of India and how would they see spirituality, but the spirituality and faith, adds some context to that. I think in many contexts is imbibed in people who have come from there. So, so I feel definitely that it plays a huge role in their mental psyche, which might be playing a part for them to continue sustaining. Yeah.

Code: Community cultural commonalities\SA culture of spirituality sustains

Participant02

Some of the health concerns which I have heard or which I have seen that were our South Asian community is more prone to hypertension and diabetes, which tends to be the COVID-19 the virus seems to be affecting in a much worse way to those who suffer from these two diseases. In that sense, it’s probably affecting the South Asian community more.

Code: Community cultural commonalities\Physical differences\Underlying diseases

Participant01

one is a lot of these people are involved directly into key worker jobs. They are more exposed to the virus.

Code: Community cultural commonalities\Employment\Key workers

Participant01

Sorry about that. It’s one, like I was saying earlier, it is, in a sense, one is the health aspect. Second is, a lot of them are involved in key worker jobs which exposes them to the virus even more. The third probably is their living conditions. Not everyone coming into this country. A majority portion of these people coming and living in this country as asylum seekers and they don’t always live in a very, they don’t always have the best of the living situations. That can probably sometimes prevent them from reaching out for help when they are unwell, maybe or they might want to hide and they don’t want anyone else to know about them in fear of losing their job or they probably losing their housing or whatever, the accommodation they have. They risk their lives more just to hide themselves from others for the fear of getting caught, which probably contributes to higher numbers to some extent.

Code: Drawing differences\Recent immigrant specific\Asylum seekers don't seek help: conjecture

Participant01

It’s not only whether they can seek help or not. Sometimes they probably don’t have the proper communication skills or the language to approach others to let them know about their difficulties. Sometimes, it’s probably—they are just scared. They don’t know. They don’t have the proper information of where they can seek help or if there is any help available at all or not. Things, already they are in a very vulnerable situation. There could be one section of the people who will probably take advantage of their situation and will demand more money or they can threaten them by saying we can evict you or we can do this and we can do that. They won’t know. They won’t come forward or they won’t know where to go to seek help. They will just give whatever problem they are having, they will just give them, keep it themselves, rather than reaching out and getting the proper help when, if they are unwell or when they are unwell.

Code: Drawing differences\Recent immigrant specific\Asylum seekers don't seek help: conjecture

Participant01

I know white British in the sense, of course, it’s—If I particularly say the British white British people, of course, they are in their own country. Communication wise they are in a much better place. They don’t have the language barrier which other sections have.

Code: Drawing differences\Recent immigrant specific\English fluency\Language

Participant01

So, of course, their living condition wise. You are not an immigrant or are you not a migrant at the end of the day.

Code: Drawing differences\Recent immigrant specific\living conditions

Participant01

Yes, of course, it’s going to affect the job situation. But that is, in terms of South Asian community, particularly if I think of the South Asian community, it’s not going to be any different from any other person who is involved who is doing their job. In that sense, no, I don’t think so.

Code: Contesting "SA Community"\Social support\No difference for SA community\No difference in ability to adhere

Participant01

I would say in my own view, probably the South Asian community is a much more—they are following the rules more. Following the social distancing more than probably their counterparts in one sense. Whether it’s their general psychology or how they work. I am yet to—I don't know exactly why. In my general with what I have seen among my friend’s circle or the immediate social circle that probably the South Asian community is following the social distancing to the book compared to the other counterpart.

Code: In-group efficacy

Participant01

I would say in my own view, probably the South Asian community is a much more—they are following the rules more. Following the social distancing more than probably their counterparts in one sense. Whether it’s their general psychology or how they work. I am yet to—I don't know exactly why. In my general with what I have seen among my friend’s circle or the immediate social circle that probably the South Asian community is following the social distancing to the book compared to the other counterpart.

Code: Institutional trust\Healthcare access\NHS access is equal\Access to healthcare

Participant01

There shouldn’t be any difference in that. NHS is free for everyone. It doesn’t ask you whether you come from, where you come from. It treats you equally. You can always access NHS. It doesn’t matter which background you are from.

Code: Institutional trust\Healthcare access\NHS access is equal

Participant01

There shouldn’t be any difference in that. NHS is free for everyone. It doesn’t ask you whether you come from, where you come from. It treats you equally. You can always access NHS. It doesn’t matter which background you are from.

Code: Institutional trust\Healthcare access\NHS access is equal\Access to healthcare

Participant01

There shouldn’t be any difference in that. NHS is free for everyone. It doesn’t ask you whether you come from, where you come from. It treats you equally. You can always access NHS. It doesn’t matter which background you are from.

Code: Institutional trust\Healthcare access\NHS access is equal\Access to healthcare\Lack of trust in healthcare due to home country experience

Participant01

No, not always. If I have to go back to your previous question whether they can access the health system, yes, they can access the health system. Whether they will do it at the right moment or not that is still, that is doubtful.

Code: Institutional trust\Healthcare access\NHS access is equal\Access to healthcare\Lack of trust in healthcare due to home country experience

Participant01

If I, again it’s all my own view and my own experience that I grew up outside this country. We came from a different society where things were especially the health system works in a different way. Where so a lot of people still expect NHS to work the same way that on demand, accessing it as immediately and I am not putting it in a correct way. Let’s say the view and incompetence in terms of how they have always perceived how the healthcare system should be, because of their own upbringing outside this country.

Code: Institutional trust\Healthcare access\NHS access is equal\Access to healthcare\Lack of trust in healthcare due to home country experience

Participant01

No, sorry, yeah. In that sense, sometimes it’s difficult for the first generations it’s difficult for them to always rely on the healthcare system or how the system or to understand how the healthcare system works in this country.

Code: Institutional trust\Healthcare access\NHS access is equal\Access to healthcare\Lack of trust in healthcare due to home country experience

Participant01

The major difference that has been first of all where I grew up in India and so the healthcare system over there is, you go see the doctor or if you have any major problems you go to the hospital and you pay the fee and you get your treatment. It’s pretty much instant what is happening, what happens there. Whereas when it comes to NHS you see your doctor and you get your referral and so there is always bit of a waiting time. It doesn’t, in my possible experience, of course, when you have an emergency and it doesn’t matter if you are getting your treatment from NHS also, you will be treated as emergency and you will get the care that’s needed at that point. If your symptoms can be treated in a much better way or with a little bit of time given a proper diagnosis or proper—getting the proper consultation. You wait for that to happen when it comes to NHS. We are not really coming from the Indian background. We haven’t got that wait. That wait in time. It unnerves you more that what is happening or not understanding what is happening and why it is happening. Why you are asked to wait for your treatment and why it’s delayed to get that treatment, that particular treatment.

Code: Institutional trust\Healthcare access\NHS access is equal\Access to healthcare\Lack of trust in healthcare due to home country experience

Participant01

My understanding, to the majority of the community it should be clear as they follow the news media or the global media. They have the information in their hands. They should be aware of the Coronavirus in the same way as any other person.

Code: In-group efficacy\Messaging recieved

Participant01

To some extent it has to happen within the community. I would say different clubs or who run or religious places can inform their people that what has been happening and how they can take the precautions of what they can do when needed or different organisations they who are involved within this community they can educate their members that what can be done.

Code: Community cultural commonalities\The collective nature of SA culture makes distancing more diff\Centrality of community groups in messaging

Participant01

The other sense is, this country has been pretty good when it comes to translated into different languages. We often see the NHS messages in Hindi or Buratti or Bengali. That can be done always to communicate in those languages so that people can understand more that what has been happening.

Code: Drawing differences\Recent immigrant specific\English fluency\Translated messages

Participant01

Difficulty in the sense it’s that like when I initially said that how there is a majority part of the section who are asylum seekers. They are already wary about revealing their status to the rest of the community or other different groups or different organisations. That can actually, rather than—that can prevent them to be open to these ideas, these messages rather or reaching to understand or just reaching out for help or if they are not in touch with the organisations or what has been happening around, they won’t get the message themselves. They have to come forward as well for the organisations to work with them.

Code: Drawing differences\Recent immigrant specific\Asylum seekers don't seek help: conjecture

Participant01

I don’t think there is anything that can affect it in a different way.

Code: Contesting "SA Community"\Social support\No difference for SA community\No difference in ability to adhere

Participant01

Otherwise I meant the health, whether they are diet restrictions or diet, but we all try to live healthier. We all try to be healthier in our life. Sometimes health conditions happen. But that is not always in our own, in our control. But in another way, yes, to some extent you can say that if someone is having diabetes, which is quite, with South Asian people are quite prone to having diabetes. Their eating habits might not help always what they are eating or they are controlling it might not always help to control the diabetes which might trigger when someone is having the COVID symptoms. In another sense, if someone is having a healthy diet or following the general hygiene, it’s nothing different they have to do. It’s just washing hands and maintaining the basic hygiene. If they are following that, it shouldn’t affect them in any different way than any other community.

Code: Community cultural commonalities\diet and exercise\Diet

Participant01

In another sense, if someone is having a healthy diet or following the general hygiene, it’s nothing different they have to do. It’s just washing hands and maintaining the basic hygiene. If they are following that, it shouldn’t affect them in any different way than any other community.

Code: Contesting "SA Community"\Social support\No difference for SA community\No difference in ability to adhere

Participant01
