# Supplementary material for: Mitigating the identity and health threat of COVID-19: Perspectives of middle-class South Asians living in the UK
Source: J Health Psychol. 2021 Jun 22;27(9):2147–60. doi: 10.1177/13591053211027626 (PMC9353968; doi:10.1177/13591053211027626)
Supplement: sj-docx-10-hpq-10.1177_13591053211027626 – for Mitigating the identity and health threat of COVID-19: Perspectives of middle-class South Asians living in the UK [file sj-docx-10-hpq-10.1177_13591053211027626.docx]

**uk7-groupb-PMU-may20**

Transcribed by Sharmistha Chaudhuri

(54 minutes)

**Part 1**

Researcher: Thank you for consenting to participate in this study. So I will start the research by asking you, what do you think is happening to the world?

Participant: It is changing very fast. In last two months, my word has changed. Every week, we used to travel to my daughter's place. Now we have not gone there for the last two months. It's very different now.

Researcher: Correct. So it has affected your personal routine.

Participant: routine, considerably.

Researcher: And what other effect do you think is, uh, is happening in the world and what the world is facing now?

Participant: This is the consequence of these viruses is very deadly and if the vaccine is not found in the next one year, we are in big trouble, big trouble. For example, my elder brother, who lives alone, he cannot get out of the house. He's in Kolkata. He's 80. He can't get out of our house. We can still go for a walk. And oppose to this, I am busy, I go for walk. I go for a long walk in the park. But there are lot of people who are housebound. They cannot get out.

Researcher: Definitely. Definitely. And what comes to your mind when you think of coronavirus?

Participant: It is not a virus which is welcome. It is too dangerous. So I get all kinds of bad feelings about coronavirus, I guess.

Researcher: So what kind of bad feeling like ?

Participant: It is, it is related to, its fatality is very high. It's not seen as a normal virus, like the influenza. When we looked at it, we thought it is a secondary item..But now it is too serious.

Researcher: How, how do you think coronavirus has affected the people in general?

Participant ([02:16](https://www.temi.com/editor/t/_ymtB8nkVuF0J59FCxgHFL8SbzGLUPsWjQSPfIJD4QxjOCAxBPplpLgAYp4ilPUx5DIu_SHFcIMkV34VlOOrS0OBl5Y?loadFrom=SharedLink&ts=136.4)):People have lost their liberty. They cannot go anywhere they like. Life has become restricted and sometimes you can be depressed. You can't go out and it's a depression setting for everybody. So now I'm finding, I have to spend a lot of time in front of the TV. I was never sitting in front of the TV ever. But now last few weeks I'm sitting in front of the TV to spend time.

Researcher ([02:45](https://www.temi.com/editor/t/_ymtB8nkVuF0J59FCxgHFL8SbzGLUPsWjQSPfIJD4QxjOCAxBPplpLgAYp4ilPUx5DIu_SHFcIMkV34VlOOrS0OBl5Y?loadFrom=SharedLink&ts=165.16)):And any other things like apart from mental depression, you see the virus has given to the world?

Participant: Yeah. Mental depression is a major thing. And then you start to doubt everything. I can’t get into the bus because I think I can get some influenzas, or the virus. So even I can get into a bus our movement has been restricted considerably and that also has an effect on the body, because we have to keep ourselves active.

Researcher: Where have you learned about coronavirus? Like, um, which of the sources that you got the knowledge of this from?

Participant: Because it came in the news. I went to work regularly till 22nd or 23rd of March. I was going to work. And then suddenly we had to stop working. Then our administrator said, you'll be working from home. So they gave me a computer and said, you don't have to come yet. You'd be working from home. Since then we are working from home. It is not an ideal answer to work from home. It is not an ideal answer.

Researcher: And which other sources you rely for, for the news?

Participant: BBC news and Sky.

Researcher: Sky news. And that's the mainly the news channel. Right?

Participant: News channels, yes.

Researcher: And what about the news in that social media?

Participant: Social media, social media. Yeah. My wife is in Facebook, but I'm not into social media. Okay.

Researcher: So you do not normally access to any material from those forum?

Participant: Yeah, actually I maybe let you send me some big Facebook messages. Sometimes some messages are sent by my brother, I sometimes open it. Sometimes I don’t open it. I go to some sites in internet, other sources like independent newspapers, I google them.

Researcher: Google. Okay. Can you share, what do you know about coronavirus?

Participant: I do not know very much about coronavirus. I know that it causes breathing problem and you probably find very difficult to breath. And my doctor had given me a call and he said, you have to be very careful. He has given me a, not a warning, but somehow he told me, you have to really careful now. and get your weight down as much as possible.

Researcher: And how about like how it spreads and how it transfers between people?

Participant ([06:00](https://www.temi.com/editor/t/_ymtB8nkVuF0J59FCxgHFL8SbzGLUPsWjQSPfIJD4QxjOCAxBPplpLgAYp4ilPUx5DIu_SHFcIMkV34VlOOrS0OBl5Y?loadFrom=SharedLink&ts=360.94)):You know, it's in the media, there's a lot of theory that it would spread from two metres. Some people say, it could be 6 metres. So, uh, it is transmitted in the air by people breathing out, speaking and things like that and body fluid. Still now I could not understand how this virus gets into the body. You can wash your hands time and again, some people say it can even go through the eyes, the NHS without eye protection, they are getting the virus, probably through the eyes. It is a very dangerous virus. we never do fully understand how it propagates.

Researcher: And in your opinion, how did coronavirus appear?

Participant: It appeared in Wuhan, in China? That is where I came to know. And then when I, on my way to work, I used to meet one gentleman who used to visit China in connection with work. And he used to visit China. He was, I said, are you not in China. He said, no, I can't go there. We have been, we have been asked not to go to China. He's not going. And he's was not going, and giving mixed messages on what is happening in China.

Researcher: So in China, what happened in Wuhan you think?

Participant: In Wuhan, you have an animal market, somewhere. They have got this virus from some animals, I think. I think it has come from animals. Some kind of virus, which has been transmitted to men from it anyway.

Researcher: So, in your opinion, how did the virus appear in the UK?

Participant: That was brought into by the tourists, and some people who went skiing to Alps. Italy, Alps. They came back with the virus, that is how it started. I think that the tourists were not stopped from coming to UK, and they also transmitted the virus.

Researcher: Right, right. Now do think about this since the beginning of the pandemic, have your perception about coronavirus changed?

Participant: It has changed because of, initially I thought that it is difficult, everybody, people will not be able to get close to coronavirus, but slowly, coronavirus or COVID, how it is spread and what is it's mechanism? And it is still, lot of grey areas. Even wearing mask or not, the government cannot decide. A lot of things have not been fully understood how coronavirus spreads and we find it difficult in our life, how to go about in our life. It has made our life very difficult for all of us, not only UK, but in places like India. It has become very difficult.

Researcher: So when you said difficult, what do you mean by saying that?

Participant: Difficult means you can’t trust public transport, which is something not very welcome. you can’t mingle with people, you can’t talk to people. My friend's son, he's researching on it, mostly it's like a flu, now I am air getting the impression that it’s not like a flu.

Researcher: What do you think about how these pandemic is different from anything we had before?

Participant: Because how it is being transmitted is not very clear. How it is getting from one man to another person- how it is spread. The way it is transmitted, if it is not clear, we cannot take care of. So through contact it is coming. What kind of contact it is being transmitted is not very clear yet.

Researcher ([10:50](https://www.temi.com/editor/t/_ymtB8nkVuF0J59FCxgHFL8SbzGLUPsWjQSPfIJD4QxjOCAxBPplpLgAYp4ilPUx5DIu_SHFcIMkV34VlOOrS0OBl5Y?loadFrom=SharedLink&ts=650.09)):And how old do you think this has a different effect than any other pandemic in the world? Apart from the transmission?

Participant: Yeah. Apart from the transmission, because it is fatal, especially for older people. For older people it is very important not to get infected with the virus. We don't have a vaccine, once a vaccine comes, maybe we might be able to deal with it.

Researcher: What do you think about your government's response to the pandemic?

Participant: Government reacted very late to this pandemic. They should have, the moment China was in trouble, we should have expected that we will be there, but the government, instead of helping us, they gave back China, some 400,000 PPEs. They should not have parted with the PPEs at that time. They should have arranged to buy some PPEs for the health workers. The health workers were left exposed. Although they have started late, they are trying to control it. This should be able to control it in the next two, three weeks.

Researcher: What was the information about coronavirus that most surprised you?

Participant: Is that there, how it is being transmitted. I never thought that people who are working in NHS will be infected. That the bus drivers will be infected. The bus drivers never meet any passenger. They are inside a box. They got infected with coronavirus. I could not work out how bus drivers got infected with coronavirus.

Researcher: You got surprised by how they got infected as well.

Participant: How they got infected by the coronavirus. Old age home also got lot of infected.

Researcher: So it's age or the intensity with which it is spreading. Right. And when you discuss about coronavirus with your friends, families, what would you discuss most?

Participant: Friends and families, especially in India, that if everybody is safe or not. Luckily for my relatives, they are keeping well. But for them, it is also difficult to avoid areas where coronavirus is there. So this makes me worried. In London, UK, We don't see a lot of people with coronavirus. But I hear some people have got it or something like that. And then we discuss, one thing I discuss with my colleagues is when we can go back to work, you know?

Researcher: So it's mainly about safety, mainly about..

Participant: Going back. And it appears that we will not go back to work as, as it was understood. But now, we will be there just there for a few hours, and work from home. Maybe we can have some small space, where if we can come and have a meeting and then go back and work from home, these kinds space..people are not encouraged to go back to work.

Researcher: And do you think working from home can help?

Participant ([14:52](https://www.temi.com/editor/t/_ymtB8nkVuF0J59FCxgHFL8SbzGLUPsWjQSPfIJD4QxjOCAxBPplpLgAYp4ilPUx5DIu_SHFcIMkV34VlOOrS0OBl5Y?loadFrom=SharedLink&ts=892.88)): I do not know, working from home is not very comfortable for the work I do. I, what I do is work with someone- he or she produces something, and this is, a lot of team work is required. It is not very comfortable, sitting isolated, working out something classic. There is a co-ordination problem. Where we are working as a team, now we are all isolated.

Researcher: And, uh, how has your personal life has been effected? Though you have mentioned some, but if you can look at the effect.

Participant: Well, as I said, we used to travel to see my grandson every week. We have not been there for four weeks now, to see my grandchildren. And, uh, so we are not interacting with our friends now just to give a phone call or something like that. And that has affected our life. I was doing a lot of moving around. I have not stepped out for a while.

Researcher ([16:09](https://www.temi.com/editor/t/_ymtB8nkVuF0J59FCxgHFL8SbzGLUPsWjQSPfIJD4QxjOCAxBPplpLgAYp4ilPUx5DIu_SHFcIMkV34VlOOrS0OBl5Y?loadFrom=SharedLink&ts=969.52)): And, do you see any kind of positive effect on your life?

Participant ([16:18](https://www.temi.com/editor/t/_ymtB8nkVuF0J59FCxgHFL8SbzGLUPsWjQSPfIJD4QxjOCAxBPplpLgAYp4ilPUx5DIu_SHFcIMkV34VlOOrS0OBl5Y?loadFrom=SharedLink&ts=978.8)): Maybe more, we see our families are more, as we are a close knit family. I see more of me, but even then I can see the restrictions on everybody else. I am not that active, but my son in law, my son more active and I find that their lives are restricted, this is due to lack of activities by the situation, they are having a lot of problems in their activities. They're facing a lot of problems.

Researcher: Got it. And give me a bit about how your daily life goes during the pandemic?

Participant: During pandemic I wake up late. I used to get up at 5:30, I was on my feet now. I get up at 6:30, when I don't have to travel. It's more relaxed. I can go for a walk, still there's plenty of light. So I then go out for a walk and come back. So there is a trade off when I don't have to travel. For traveling, there is some, you lose some energy, with lot of exercise. You climb up the overbridge, you climb down the overbridge. That lost some weight. I gained some weight in the last.

Researcher: So you have gained some weight (laugh).

Participant :When you travel, you have to go up the overbridge, come down the overbridge. I have to travel to Sutton. I take a tram to West Croydon. From West Croydon I take the train. Then I get down a Sutton. I have to take the next connecting train, I get down the stairs. Then I have to go to Leatherhead. For Leatherhead, I have to run to bus stop because sometimes the bus leaves, but then I have to walk down to work.

Researcher: That is not bad. That is not being compensated by walk.

Participant: No, no, exercise missing.

Researcher: Exercise missing in your life..did you use to go to gym? or any other place?

Researcher ([19:06](https://www.temi.com/editor/t/_ymtB8nkVuF0J59FCxgHFL8SbzGLUPsWjQSPfIJD4QxjOCAxBPplpLgAYp4ilPUx5DIu_SHFcIMkV34VlOOrS0OBl5Y?loadFrom=SharedLink&ts=1146.5)): No, I used to be, but I have given up on gym. I had a personal trainer, and he used to, he used to come and he used to set my back. I had a back problem about one year back. So he corrected my posture and everything, but now he cannot because of coronavirus. He had been very helpful. I am not going to the gym, he told me what exercises to do to take care of the back. And now he is giving advice, the money I spend at gym, I am doing at home. He told me all the exercises, but he's not been able to come, or I can go to him.

Researcher ([19:48](https://www.temi.com/editor/t/_ymtB8nkVuF0J59FCxgHFL8SbzGLUPsWjQSPfIJD4QxjOCAxBPplpLgAYp4ilPUx5DIu_SHFcIMkV34VlOOrS0OBl5Y?loadFrom=SharedLink&ts=1188.68)): How about eating habit? Has it changed?

Participant: Eating habit, has become something more. I think more is normal, because every day you are eating ice-cream. Whenever I sit, I am having ice-creams.

Researcher: So you are pampering yourself!

Researcher: Yeah. But when you go to work on, so you, apart from the lunch or the tiffin you take with you, Normally there is some social get together...somebody's birthday, somebody who's got a promotion. Or somebody sitting in the pub. Those things are not here. Sometimes families have also some samosas, some cakes, those things are not there.

Researcher: And, uh, could you please tell, tell us, um, what do you think, how this pandemic is going to end.

Participant: It is a very difficult question! I do not know when I shall go back to work. I hope some kind of vaccine should come. Even in India people think that Oxford have got the answer to it. I don’t know why...they will tell me, oh, they have found something in Oxford, that vaccine will be made in India. I hope God provides an answer to this, because we are in a big trouble, because, for instance, your son or children will be going to school in the next few weeks I hope. But till then you are going back to school, everybody has to work out how things will be going there. We are in a difficult phase now, we have to live with this new virus.

Researcher: So do you, when you say re-adjusting, what do you mean by re-adjusting?

Participant: Re-adjusting means, we have lost the normality now. Two months now, and now we have to change our mentality. For instance, in the winter, we would go to India and spend time. it is a nice time. But now, things will be different. The plane fare will go up. everything costly, life will become much more costly now. And that was a big depression. World economy will go through depression. We have last seen depression in 1930. Even 2008 depression was nothing compared to that. we will go through a similar depression now. All of us will be having trouble I think, because the world economy is shattered.

Researcher: So why do you think the economy has been hit by the virus? What are the things?

Participant: Because there is no economy activity for last or seven weeks. Even our local restaurants, it really, it doesn't have any output. Even the bars are closed, so many activities that are taking place normally, we thought that they are not important. In fact, they are all economic activities, all have been stopped and they will all take a hit. Even the airlines, while 600, 700 will travel in the end of November. Now, it will be maybe daily 1500 or 1600. So everything will be costly now.

Researcher: And, um, how do you think that we are able to prevent further depression like this in future?

Participant ([23:46](https://www.temi.com/editor/t/_ymtB8nkVuF0J59FCxgHFL8SbzGLUPsWjQSPfIJD4QxjOCAxBPplpLgAYp4ilPUx5DIu_SHFcIMkV34VlOOrS0OBl5Y?loadFrom=SharedLink&ts=1426.94)): So these kind of pandemic has to be avoided. In the future we should be having some system where, when this kind of virus starts, there should be everybody, the world should be told immediately; that area should be covered up. That that area should be up and immediately some world help operation to take care of it, stopping there. It should have been stopped at Wuhan itself.

Researcher: Would you think this is possible or to, how extent?

Participant ([24:26](https://www.temi.com/editor/t/_ymtB8nkVuF0J59FCxgHFL8SbzGLUPsWjQSPfIJD4QxjOCAxBPplpLgAYp4ilPUx5DIu_SHFcIMkV34VlOOrS0OBl5Y?loadFrom=SharedLink&ts=1466.32)): Possibly. If all countries think that we are one group, one group. Because we are interconnected and everybody has to take in globally. You cannot think that this family will stay, this country will not come, whatever. Like in India, one state is thinking it is another state's problem. It is not that. It is a universal problem. The whole world has some one global issue and everybody has to work to solve it.

Researcher ([25:01](https://www.temi.com/editor/t/_ymtB8nkVuF0J59FCxgHFL8SbzGLUPsWjQSPfIJD4QxjOCAxBPplpLgAYp4ilPUx5DIu_SHFcIMkV34VlOOrS0OBl5Y?loadFrom=SharedLink&ts=1501.63)): Together. And, um, we are at the end of the section one, just the last question. Apart from that health and mental depression, what you have mentioned, um, what are the other effect you see of the virus?

Participant: Big economical, effect. Psychological effects. You start doubting where should I go, or should not go. Shall I shut down. Previously, I never doubted. I never thought about this problem. Now. I always think, shall I go, or not- there are so many places. For instance, I was doing voluntary work for the Oval cricket ground, because this is where you get this virus. There the chance of getting the virus is very high, where a big crowd is gathering. I just stopped that activity. I will give them a notice. Maybe, I..they are also going through a difficult time, I will not give my notice now, but in the next coming few months, I will give them a notice. I am not going to work in the Oval playground anymore.

Researcher: And do you think human has learned any lesson from this pandemic?

Participant: I don't think man has learned any lesson. Man thinks he is global- it is his world, he can do anything- But this is he got. Maybe God has an answer to that- you have gone too far, you should stop. Climate change is a major issue now. This is part of the climate change maybe. We should not eat anything- whatever is available in the market, we have to think twice what we are eating. our eating habits has to be more restrained. All these pandemic, we transfer from germs from animal to man, we have to learn that animal products...animal market has to be stopped. I had been told that Wuhan animal market has been closed. I hope that is the case. Even within our country there are some animal markets which has to be stopped.

Researcher: So how about the non-vegetarian food? How do you see that?

Participant: Non vegetarian food is not very helpful. This is what I find. I was not in favour of non vegeratrian food. But now that it is better to be vegetative, or eggetarian, or maybe you can take fish. But meat, all kinds of meat are not very healthy.

Researcher: Is it something new which has occurred to you after this pandemic

Participant: It has occurred to me that meat, in any form, is not healthy.

Researcher: So that's a new realisation you had?

Participant ([28:04](https://www.temi.com/editor/t/_ymtB8nkVuF0J59FCxgHFL8SbzGLUPsWjQSPfIJD4QxjOCAxBPplpLgAYp4ilPUx5DIu_SHFcIMkV34VlOOrS0OBl5Y?loadFrom=SharedLink&ts=1684.34)):Yeah. It is better to be vegetarian than non-vegetarian.

**Part II**
Researcher ([28:09](https://www.temi.com/editor/t/_ymtB8nkVuF0J59FCxgHFL8SbzGLUPsWjQSPfIJD4QxjOCAxBPplpLgAYp4ilPUx5DIu_SHFcIMkV34VlOOrS0OBl5Y?loadFrom=SharedLink&ts=1689.56)): Okay, thanks. That is the end of the part one, which is very helpful. Okay. And I will now go to the section two of the interview in which, um, I will ask you, being focused on the South Asian communities.

Participant ([28:30](https://www.temi.com/editor/t/_ymtB8nkVuF0J59FCxgHFL8SbzGLUPsWjQSPfIJD4QxjOCAxBPplpLgAYp4ilPUx5DIu_SHFcIMkV34VlOOrS0OBl5Y?loadFrom=SharedLink&ts=1710.95)):Yeah.

Researcher: Now the first question I start with, is, what do you think are the main health concern for people in the South Asian community during the pandemic? And why.

Participant: It is a coincidence that three weeks ago, I had a blood test- maybe 1st of April. Then I spoke to my doctor. He said, there is no problem with your blood, all absolutely fine-but there is only one problem. The glucose content is high comparatively: some 6.3. He said, this is a border case, you have to be very active. And this is the case of most South Asians. The glucose content becomes high. And that is their glucose level goes high- They lack physical activity. Physical activity for South Asians are very low. They not taking part in sports or any other thing. That is a very important aspect, which is missing in our lives.

Researcher: So do you think South Asians are less active sports wise?

Participant: Much less! Not less, but mostly they are not very active. And that is the problem. We've got the same problems in Kolkata. If you go to Kolkata there are 100s of people standing in front of medical shop. People are buying medicines! People need some vigorous activity, like cycling, or maybe walking, or running. Lot of people are running, I go to the streets, I find those people who were not running 2 months ago, they have started running now. So vigorous exercise is very important. It gets your glucose level down.

Researcher: Do you think, that, the South Asian people are more or less at risk of this virus?

Participant: More at risk.

Researcher: And that, that reason would be because?

Participant: Because we less of this physical activity, I won't comment on the food, but the physical activity, most, really.

Researcher: And why do you think so? Like the South Asians, maybe less active?

Researcher: I had spoken to my doctor. Previously I had attended a course by National Health Service. The day I retired, they called me for some seminar and there the scientist told me that the South Asian people have high glucose level, because of the physical inactivity. Normally it should be a 10 thousand steps a day, that is not a tall order. Even 6000 or 7000 steps a day would be a good exposure to start with. Or some kind of swimming or, uh, maybe cycling. I see in our road, he goes cycling every day. His name is Mr. R. It is not only shopping, he walks down to the shop. He does cycling, uphill cycling in the morning. He goes to the Lloyds park tennis ground, he plays there. He's 80 plus. And he's upright, straight. So physical activity is at the central idea.

Researcher ([32:34](https://www.temi.com/editor/t/_ymtB8nkVuF0J59FCxgHFL8SbzGLUPsWjQSPfIJD4QxjOCAxBPplpLgAYp4ilPUx5DIu_SHFcIMkV34VlOOrS0OBl5Y?loadFrom=SharedLink&ts=1954.59)): And looking at the south Asian community. How do you think it has been specifically affected by the coronavirus?

Participant ([32:45](https://www.temi.com/editor/t/_ymtB8nkVuF0J59FCxgHFL8SbzGLUPsWjQSPfIJD4QxjOCAxBPplpLgAYp4ilPUx5DIu_SHFcIMkV34VlOOrS0OBl5Y?loadFrom=SharedLink&ts=1965.04)): I don't know whether this, how much the south Asian community are affected, but the, the news that is coming, a lot of south Asians have passed away. All of them having underlying health conditions. I don't know what underlying conditions that had, in the South Asian community. One doctor complained about the PPE, I don’t know whether it is PPE or some other factor. But a lot of people in the south Asian community have passed away. The proportion of people in South Asian communities are quite high. So we have to be very careful.

Researcher: And how would you think, like, not only looking from the health ground, but from economic ground or education or any other aspect, do you think they are more affected than the white people? More or less?

Participant: This very difficult to say, but economic aspects I don't know, but economic aspects are important, but maybe South Asians do not the follow the rules. For instance, Ramadan is here, a lot of people are people mixing with one another. I do not know why religious requirement be so stringent. They should have been more careful, during the Ramadan period. They are mixing now, during Ramadan.

Researcher: Yes, certainly. Yes. And, um, how has your family been affected by the coronavirus? I know you mentioned your daily life and everything, but to summarize health wise, or trade wise or anything else?

Participant: Before the pandemic I used to go and work, and this is very difficult working from home. My work requires a lot of co-ordination. co-ordination is missing. Going to office and working from home, It is quite different. And I, I do a lot of cross reference. A lot of, a lot of cross reference are needed, which I cannot consult. So workwise it has affected, and my family, especially India, my son, they have got a company. So the company is in lockdown now.. So, so it is not a nice period for him also. And then my elder brother who lives in India, he lives in a house, all alone. They are immobile, they cannot get out. And then their children, my grandchildren, they can’t go to school, but their family is also affected. So this is not a situation, we would like planned to have it, right. This is how we are now, their activities are affected.

Researcher: So would you think, uh, commenting on that, that, even the South Asian community are more or less affected by than the white British?

Participant: I think they are more affected than the white British.

Researcher: why do you say so?

Participant: Because I think the work they are doing at national health, particularly, they they are more exposed? I can't tell the complete thing on that, but a lot of south Asians who are have passed away. Doctors have also passed away, some 16 or 17 of them, I heard the other day. Doctors are fully knowledgeable. How they are passing away I do not know. They are not very old, but something to do with the gene, maybe genes. The way our body is constructed that can happen. We have to study what kind of DNAs we have. The Chinese. The Chinese are not affected. The Chinese are better than us.

Researcher ([37:28](https://www.temi.com/editor/t/_ymtB8nkVuF0J59FCxgHFL8SbzGLUPsWjQSPfIJD4QxjOCAxBPplpLgAYp4ilPUx5DIu_SHFcIMkV34VlOOrS0OBl5Y?loadFrom=SharedLink&ts=2248.73)): And I'm looking at the government measures, like working from home, social distancing, restriction on travel, et cetera. How do you think the South Asian community are coping with these restrictions?

Participant: South Asian communities are mainly following the rules. But mainly, some part of the South Asian community are not following the rules, like Ramadan and Islam. But generally, South Asians are following the rules. But certain community is not necessarily following the rules. Not following the rules because of religious requirement. They think that religion requirement is higher than anything else. This is a matter of debate that whether religion should come higher than other social or scientific requirement? I do not know. There is no scientific evidence that if you mix freely, this can be transferred at a faster rate.

Researcher: Are there any specific difficulties the community is facing do abide by the directives of the government?

Participant: I think there is no difficulty as such to abide by the requirements of the government, because anything, all the basic necessities, you can get from the shops, the local shops. There are some shops, like local Turkish shop, or maybe the Asian shops, they are not exactly following the same rules. But our local butcher or the local fruit shop, they are all following all the distancing rules. But the other grocery shops are not following the same rules.

Researcher: Why you think that should be? Why do they do that?

Participant: Difficult to say, But maybe the shop layout is such, that they think if they allow only two or three, uh, uh, two or three customers, at a time, it will take time, and will lose the customers. You want to boost the sales go. They don’t stop people from coming in. But our local butcher, he has got policy. Only one man can get in, one at a time. Last time I saw him he allows only one man at a time.

Researcher: And health wise, do you think the south Asian people have the same ease? Same ease of access to the healthcare facilities as the white British?

Participant: I think so. Yeah. There are some constraints I think everybody's faces because if you want doctor's appointment. That if you wanted a doctor appointment, you have to go to Mayday hospital. There are some constraints everybody's facing. I don’t think there is any specific technical difficulty in the South Asian community. Our local GP has got a budget. So once his budget is over, he won’t entertain. If you have any illness, you go in the next financial year. Budget is over, he will not entertain. This is the constrain, the government has put on the GP here. It's not personal, something personal piece. Our GP, he comes from Karachi. But both his parents come from India. His grandparents used to come. I think his grandfather was doctor to the Nizam of Hyderabad, India. He has got a lot of connections in India. he says, I can't help you, if you want a treatment, you come to the next financial year (laughs).

Researcher: Do you think the South Asian people trust the government decisions on the directives?

Participant ([42:25](https://www.temi.com/editor/t/_ymtB8nkVuF0J59FCxgHFL8SbzGLUPsWjQSPfIJD4QxjOCAxBPplpLgAYp4ilPUx5DIu_SHFcIMkV34VlOOrS0OBl5Y?loadFrom=SharedLink&ts=2545.53)):In general everybody trusts the government, their decisions and directives.

Researcher ([42:34](https://www.temi.com/editor/t/_ymtB8nkVuF0J59FCxgHFL8SbzGLUPsWjQSPfIJD4QxjOCAxBPplpLgAYp4ilPUx5DIu_SHFcIMkV34VlOOrS0OBl5Y?loadFrom=SharedLink&ts=2554.93)): And, to what extent do you think that people in South Asian community understand the health messages surrounding these directives?

Participant: I don't think they fully understand the health messages that are given, either by the government, or the doctors, I think they have not fully understood them.

Researcher: That transparency is not there you think, reaching the community?

Participant: Yeah. It is not reaching the community fully. They think that, no, no, this is not big. We can do whatever we like. So that maybe some, misunderstanding. People don't take it very seriously.

Researcher: Why do you think that some people would not take it seriously?

Participant: Because our thinking is not based on it on those, like, that that we can avoid getting the virus, by avoiding close contact, or social gathering and things like that. That kind of understanding is still not there. They do whatever they want right there. If you go to any Asian shop, you can freely go, roam around it..I don't think that thing is there in the other shops.

Researcher: So, uh, how do you think any improvement could be done with the messages, uh, to this community to make them more aware of the situation?

Participant: Very difficult problem. Because this is a cultural issue, because... If you go to India, any toilet, public toilet, you got to wash your hands, but you cannot find a soap there. Toilets are not cleaned properly. Lot of toilet which are used are not cleaned properly. So the public is not much aware of the cleanliness in general. That has permeated to the society.

Researcher: Any other aspect you would think, like community, language? The way of living, which may that may have interfered with their understanding the messages?

Participant: For language. I think that everybody understands what is going on. some people think that oh, this is that we don't have to take seriously. but only when some families get fatalities, then only they understand, what is the consequence of this virus. I don't think full understanding has still come, and are recognised.

Researcher: And we are at near the end of our interview. I would like to ask, what do you think has helped you or the South Asian family in general to deal with this crisis?

Participant: Yes, there is a communal feeling everywhere; there is a local community here. They come out every Thursday, and they are clapping for NHS. And that is where we are in the same boat, all of us, to ride the storm. We are in the community, local community, everybody is helping each other, and I can see a lot of people are getting food also from some supplier. People are getting food for those who cannot prepare food, or have other sources of food. People in general are very helpful. If we do get on top of whatever the problem is, a lot of people are asking, my neighbours are asking, do you need a help? Every time they pass by our driveway they are asking, do we need any help? Everybody ready to come and help us because we are older than the general population. I don’t have to ask, they come and ask us, are you keeping fine?

Researcher: Do you think the same applies to the South Asian community in general?

Participant: I do not know about the South Asian community, it might be there. But my local community here, they come from all around the world. They are always talking to each other, helping each other. That is why I like, that I find a bigger family. So if we have any problem, they can come and help us. Only our neighbour is not very helpful. But he is Chinese (laughs). If few leaves fall from our tree, our bush to his area..I tell my grandson, when they are misbehaving, I will tell that to my neighbour. They are not very friendly. Otherwise, our whole area, people are very friendly. On Thursdays, they all come out in the street and clap. You see South Asian community world over. More than 50 percent are non-English. Everybody are trying to help us. So what do you think, like reflecting on the South Asian community? Is that any force you see that that can help them to sustain? Or anything different from the white British people ?

No one is here to understand that this virus is going to come and go, so we have to be more physically active. Make ourselves fitter and leaner. I Think that is how we can sustain the virus. Eating meat is not very helpful. In Ramadan, during iftar, they eat a lot of meat, that is not helpful.

Researcher: That's right. So we have more or else covered everything in the interview. Do you have anything to add?

Participant: Yes, another thing, about vegetarian diet. It is helpful, even for ecological reasons, vegetarian diets are in. Being a Bengali, two months back, I did not have the heart to cut the non-veg.

Researcher: Are you now turning to vegetarian then?

Participant: No, no. If you tell my son that we are going to a party, he will first ask whether it is vegetarian or non-vegetarian. The moment I say vegetarian, he will not go. My brother in law, he is a senior man, said we have got a dinner in the evening, can you, will you come with us? They are inviting you also. That family is from UP (Uttar Pradesh in India), he said, he won’t come because it would be a vegetarian meal.

Researcher: Is there anything else you would like to add on anything that we missed out?

Participant: No, I think we have covered most of the subject.

Researcher: (52:45) Thank you. I'm ending the recording now. Thank you so much.
