# Supplementary material for: Mitigating the identity and health threat of COVID-19: Perspectives of middle-class South Asians living in the UK
Source: J Health Psychol. 2021 Jun 22;27(9):2147–60. doi: 10.1177/13591053211027626 (PMC9353968; doi:10.1177/13591053211027626)
Supplement: sj-docx-11-hpq-10.1177_13591053211027626 – for Mitigating the identity and health threat of COVID-19: Perspectives of middle-class South Asians living in the UK [file sj-docx-11-hpq-10.1177_13591053211027626.docx]

**UK 8 – GROUP A**

**I:** Thank you for your participation again. I will start with the question, what do you think is happening to the world?

**R:** I think we are witnessing a kind of situation which usually happens once in a century. A pandemic situation. There are lots of different views on it. I personally tried to take the middle ground because I think truth can be in many different sides. We have to be sensible in kind of taking the middle ground, possibly the middle ground has a better version of truth, but it’s just my opinion. There are some conspiracy theories going on, but the problem is real. It is very much real and it is happening around us. By now, most of us know somebody or other who has got the virus or recovered from it or even died from it. It’s very real and that is my initial perception of it all.

**I:** What do you mean when you say, middle ground?

**R:** When I say, middle ground, I mean there are lots of opposing views on this. Some people saying, this is just the normal virus that has been going on for some big pharma or vaccination conspiracy company is going on. All of this is okay. I am not refuting or supporting any of them, because they then have some truth in them. We have to see things sensibly from a very biological point of view, what it is doing to me, to myself or my immediate family. It is very clear how and what your behaviour would be so sort of situation. I think that’s why a middle ground like you don’t have to be paranoid with it or **[Unclear]** 02.26. You also have to be very complacent about it.

**I:** That makes sense.

**R:** That is what I mean by middle ground.

**I:** What comes to your mind when you think of Coronavirus?

**R:** That is a very interesting question. If I say something it will come as a completely different—I don't know. I mean, one thing that I think about the Coronavirus is it has really levelled up a lot of things in the whole world. The whole world is affected. Honestly, situations are different specific to the regions and the culture and economy of the country. More or less it has affected people equally. It is like a big leveller that is the main thing I would say about Coronavirus. Another thing is, it has brought forward some of the existing problems in our society. Health-wise and lifestyle wise, economy wise, it has brought up these things. If you see some of the migrant workers in India or even essential workers in the UK suffering or at high risk or even doctors at high risk from it, it perhaps shows all the existing inadequacies in our infrastructure within the government and in the society at personal level and also a government political level.

**I:** That is a very interesting answer. Thank you for that. How do you think Coronavirus has affected the people in general?

**R:** People in the UK?

**I:** All over. Like maybe for two other countries. Doesn’t matter. It’s just your perception how the people in general.

**R:** If I had to say it has somewhat affected people a little bit differently according to people’s socioeconomic structure. At the same time, there is a lot lack of awareness also. Even though everybody is talking about it, there is a lot of a lack awareness like we see in places like Florida beaches or even the parks there in the UK or even some places in other parts of the world no matter what economic or education background they are from. They are not listening to some of the rules that you have to follow right now. These rules are not necessarily imposed to control people, for the biological reasons. The reasons are very biological and medical. Of course, there can be disagreements on how much strict one can be and one cannot be. It is also up to the individual. The collective, as a part of a collective society, I think a lot of people are not really understanding the gravity of the situation, sometimes. That is something that I can see and which kind of bothers me a little bit as well.

06.12

**I:** How does the social status come in here?

**R:** Let’s say, for example, if it’s a very poor person, let’s say, a migrant worker. We have other basic problems in the everyday life already that has been brought to the fore because of the lockdown even more. They already had those problems before. But they are more in a problematic because of the lockdown and they can’t go back to their homes and recently they were able to because of the trains and whatever, but then they were in the lockdown for a long time. Nobody was really—I am not blaming any particular government or any particular people. It’s sometimes difficult also because it’s a huge population.

**I:** Are you referring to India in this one?

**R:** I am talking about countries like India, Bangladesh, Pakistan, these kind of countries. Because the population is huge. Sometimes even the government don’t know how to reach them. At the same time, we see media reports of certain people being brought back home on aeroplane and on special convoys or whatever. I am wondering if the social status of the people really matter here. They probably do. These migrant workers, they possibly never mattered in the society, to some extent. Obviously, they matter obviously the media is talking about it, but they still have the problem. Also, I know now, again, I am saying something contrary to what I am just saying. I know somebody who is a researcher and a student like I am in China and he is Indian. He is now at a point where he wants to go back to India, but when he contacted the embassy there was no constructive or positive response. So, these are also happening.

**I:** Mobility to taking them to that place of the residents or work.

**R:** I am not saying these are not happening. It’s not reaching everyone maybe because it’s not possible to reach everyone. It is a big if, ifs and buts.

**I:** If I ask you how have you learned about Coronavirus? Or how you keep yourself updated like various news sources, which one you rely on most?

**R:** The first time I came to know about it. I might have seen it from Facebook posts by somebody. I didn’t look into it as much. The three ways that I became aware of it, more consciously than like just skimming through a news article, which I didn’t really care much about or something was when I was one day travelling to London and I took, actually no, not London. I was going somewhere nearby and took an uber. The uber driver told me that if you are going to London, be very careful of Chinese people. I was like, why Chinese people? He said it in a very crass way, but he didn’t mean it in a bad way. What he was saying was that there is a virus outbreak in China and if people are coming from China as they are like not restricted to move here. You should be careful being in London because London is a place where there is so many people out there and lots of Chinese people are out there. He didn’t say it in a very discriminatory way or anything. He was saying it in a very practical way. I was like, okay. I know you know that uber drivers, they know about what is going on. What is really going on on the ground and they really know that. That made me very careful and then when mother, she called me up and she’s like, you need to be very careful about this. This is coming. I don’t know how she knew about it. She said, this is coming. I think I know why she said that because she has endured certain situations in her life when she was younger. She herself was in the middle of an epidemic, during the war in 1971. She has that memory and she has that experience. Maybe she heard it from somewhere she got alerted immediately and she told it to me. The third source that I got alert from was that friend in China that I told you about. It’s like you need to buy mask if you haven’t already.

**I:** Do you remember when was it? What time period was it?

**R:** I think it was mid-February, something like that.

**I:** You were just at the start of the pandemic.

**R:** Yes, yes.

**I:** Do you look at any other sources, news, media, or anything to keep you updated?

**R:** After I had heard from them, I saw various sources like numerous sources like news, the mainstream news channels and some YouTube. On YouTube they have Sky News, ITV, Daily Mail, Guardian and Telegraph, all of that. I used to see them like constantly getting updates and to verify. I tried to get **[Unclear]** 12.39 each news source will say different things, sometimes. One will miss the other. A lot of pseudo-science were also there. It doesn’t matter, it’s good to be aware nonetheless.

**I:** What about WhatsApp, Facebook?

**R:** All these news sources that I am telling, they were shared on Facebook, mainly. What happens nowadays is, when you click on them, they become notifications on your phone automatically from Google. If you are looking at certain sources, they become like the notification search, automatically. There is how I keep getting all these updates everyday on my phone.

**I:** How much do you think you trust the WhatsApp sources like various news going on?

**R:** Honestly, I don’t really have that kind of thing on WhatsApp. A lot of people send me, not many do. Some of the things people share are kind of shady. They really don’t give me a very comprehensive idea of what I am looking for. I only get and some like my friend in China, he sometimes sends me updates like Chinese news portal, which is in English. Then I can read them and I can understand. He tells me himself what he is going through like first-hand account, like how, in everyday life he and what restrictions he has to follow. That gives me an idea. I think that is more authentic for me than any WhatsApp or any other source.

**I:** First-hand information. With all your research and all your reading, could you tell us what do you know about Coronavirus?

**R:** I am not, I mean, well, the first thing I can say is, whenever I hear of a virus, I go back to my own studies of Coronavirus. A virus has a protein coat and it has an RNA or a DNA. This one is an RNA and so likely to be more deadly. I know that Coronavirus itself has some variants. It spreads very quickly. It’s very potent. There are lots of different views on how it is spread in the air or between people to people. I use some common-sense as well because, for example, if you and I are sitting very close together, when we talk, droplets can get exchanged. Nobody we would not care about it, but now you have to. I think there is a general consensus in many European countries and you don’t really need to wear a mask. My personal opinion is, please wear it. I personally wear it as well. I look at those countries will have experience of dealing with the virus or like SARS or other outbreaks. For example, Hong Kong or South Korea or China and for them, wearing a mask is like strict. It’s for the good of the people. It’s not like it is some agenda they have to control people or all these kinds of things. I am not judging those who don’t wear it here. I just wear it for my own safety and for the safety of the others.

16.51

**I:** And now a difficult question. In your opinion, how did Coronavirus appear?

**R:** In these sort of things, I don’t like to take conclusions. A lot of my friends who in microbiology or pharmacy sector, they tell me that it is most possibly a normal virus that has developed over the years. Coronavirus has become kind of threat, over time. There will always be new viruses that keep coming up. Maybe it will affect us more if we move away from our natural resistance or if we don’t healthy or live healthy, these kind of outbreaks will be more often. That is one side and the other side is lots of blame going on between different nations that all this was in a laboratory and this leaked out from a laboratory. My personal opinion is if it did leak out from a laboratory, it was not intentional. It happened by mistake if it came out from a laboratory. Or it has really developed in the natural environment like how viruses develop. I really don’t have a conclusive answer to that because I am not a judge. I don’t have all the facts.

**I:** So, it could be down to the eating habit so to say. What you mentioned.

**R:** No, what I meant to say is, our lifestyle in everyday life like—we really don’t care about what will give us more immunity, in general and not about the virus, but in general, we don’t really care about it. A lot of people say, it is only affecting those who already have in England. Do we know what ailments we have or not. Do we know that if we are immune to it, we might think we are healthy and very young. A lot of young people have died with this virus. They were perfectly healthy. Maybe have some of the other problems, but obviously they didn’t know about it. I am sure a lot of people didn’t know that if like for example, me, maybe I have got high cholesterol and I don’t even know about it. What I mean to say is, there is a lack of awareness of what immunity is or you know. Viruses develop over time. Like any virus can become deadly at any point in time. How it is developed is a complex thing, obviously. I cannot say, researchers can say. Some people say that it came from the bats. It transferred from different animals in the wet markets. That is possible. I am not saying because there have been some papers that were published last year. I think I read a few of those articles. Maybe they tried to warn people, but nobody really gave it much importance because it was just another article in the **[Unclear]** 20.11 or some portal. I don’t have a definite conclusion about what made this virus appear and whether it was a conspiracy or it was a bio-weapon or not. I don’t know that. I really don’t know.

**I:** In your opinion, how did it appear in the UK?

**R:** In the UK definitely it was travel. It was unrestricted travel. I think they should have, when the outbreak was happening, they should have restricted flights from China or other potent places like Italy or Iran, but they didn’t. Even if they don’t restrict the flights, they should have carried out measures to quarantine them and test them or have those facilities available. I think nobody really cared about it. It kind of happened on its own.

**I:** Just appeared. Now something to think like from the beginning of the pandemic, has your perception of Coronavirus changed? No.

**R:** My perception really hasn’t changed much. Regarding my behaviour from the very beginning, I became very careful. When I saw nobody was worried about it and I was washing, cleaning everything. I was maintaining hygiene by myself. That still exists whenever I go out. I haven't gone out in a very long time. I only go out when I have to really buy some perishable food, otherwise I don’t go out at all. I can go out for walks and I am then okay, but even that I don’t do because then I have to think about a lot of things. I have to come back and wash everything. Your mind has to be very alert. It may sound a little bit paranoid, but it’s better to be safe than sorry. I am completely by myself and nobody is there to take care of me and I don’t know if I really get it. I don’t know what will happen to me and whether the medical facilities are as good or not. I don't know. It’s better to stay healthy.

22.43

**I:** Absolutely. Do only what is necessary. Correct. Do you think this pandemic is any different from anything we had in the past?

**R:** Yeah, definitely it is different. I think it is very different because, in a very short time a lot of people are getting affected and a lot of deaths are happening in a very short time. Because a lot of people are saying that there are so many other people dying of other diseases. Nobody is talking about it because this is supposed to be some kind of answer. They want people to panic and because some big Pharma conspiracy. I don’t really think so. You can see that it is spreading. The uncertainty of the spread and the uncertainty of who will survive and who will die is so uncertain and rapid. One of my friend’s dad, he was taken to hospital in Tarka. The next day he died. His symptoms were Covid symptoms. Obviously, he was old and probably had other complications. It was so sudden. It was so sudden. I think it is definitely different. We cannot see that it’s like the other diseases where people are dying every day. Every year there is an account of death due to some things like cancer or HIV. Sure, there are. We know about it. We are aware of it for a long time. Their method of spread is different. Cancer is genetic and you can’t do really much about it. It’s the spread. It’s the way that it’s spreading the uncertainty of how it will affect people I think. It is very different from the other diseases that we already have.

**I:** When it started to spread, did your perception change like how it is going to affect or anything around the knowledge of the virus or pretty much the same thing you have.

**R:** I don’t see much difference in me, but I saw it around me. People I know, when I told them or discussed with them about it, lots of people gave me the impression that I am being paranoid. I didn’t tell them anything. Sometimes I said, okay, we will have this conversation after two or three weeks. I think I missed something. I also got alert when I saw the graphs. There are some data modellers. They were not scientists. They were not epidemiologists. They were some data scientist. They did some incredible to warn people about how quickly it is going to spread and the numbers will shoot up. If we know even basic math, you don’t have to be a data modeller. If you know basic math, you can know that, okay, if you know that London has one patient, by two weeks it will double and we will know what will happen next. That is what I thought. I spoke with a lot of people who would have similar socioeconomic educational background. They were not really bothered. They thought what—I am not judging them. I am saying they were not bothered and they were like, it’s nothing. Don’t worry about it. I am like, yeah, I am not worrying about it. I am not panicking. I have to take the measures and I would like you to take the measures if you are my friend. I will just say that. After two weeks there, they changed, obviously.

**I:** Obviously, they are in your own league now.

**R:** I am not rubbing it on them or anything. They have to also be careful.

**I:** Reality has come in. What do you think about your government response to the pandemic?

27.20

**R:** The UK government I think it was trying to be very politically correct in many ways. It’s going to affect our freedom, blah, blah, blah. I understand their position as well. There are a lot of people I know. If they had a strict lockdown measure from the beginning a lot of people would say, oh, this is bad. Now they haven't done it, they will also be criticised now. Now we know that it was kind of wrong not to take measures. I always felt bad from the beginning, even mid-February. I always felt that they should take those measures, but they didn’t. I think they had a flop. They had an understanding. I think, I was quite surprised that the main advisor, the scientific advisor who advised them about herd immunity. It wasn’t—they were expecting people to die. You don’t know who is going to die and who is going to grow immune to it. We don’t know if you and I would be immune to it. You don’t know. It was a very stupid thing I think that they had done. I forgot his name. I really don’t remember, he’s from the London Tropical School of Hygiene and Medicine or something. Even after the weeks he was interviewed, now you know that you are wrong. I think he was having a debate with that data modeller from the US. He was Brazilian or Portuguese or an Italian, I am not sure. He was American. They had a debate on Channel 4 or something. He was saying like, you need to listen and you are ahead of Italy. You have a few week’s time, a window of time and you need to have strict lockdown. They did start the lockdown before they decided they wouldn't, but they did. It was still late. It was still very late.

**I:** Correct. What do you think the information about Coronavirus that most surprised you?

**R:** I think it didn’t really surprise me. I think the only thing that concerned me was the fact that it was here in my own place that I stay. Whenever there is an epidemic, you think it’s Ebola and it’s in Africa or always in China or somewhere far and it’s not going to affect you. Usually it doesn’t because there are certain reasons that it doesn’t spread. What else was surprising, I think, okay, there is another thing that surprised that how real it was to one of the Hollywood films like there was one Hollywood film called, Contagion and I watched it and it was so similar. There was differences. It was so similar. Even some of the debates going on, the political debates going on. It was so similar. I felt like it was a scripted thing and it’s happening now.

**I:** That is funny isn’t it. Funny in a real sarcastic manner.

**R:** Exactly. It was kind of more big fun.

**I:** When you discuss Coronavirus with other people like you are friends, family, colleague. What do you mostly discuss?

**R:** We talk about potential travel in the future. I think we now have a chat group which we never had in my entire life. Me and my cousin we used to talk to each other randomly, not as regularly. Now we have a chat group and now we have elaborate plans of how we are going to go on holiday when this is all over.

**I:** I think that is a positive way to look at it at the moment.

**R:** We do that. At the same time, I think like death, as a subject comes more often in our conversation. Like oh, if I die, this going to happen or if somebody is concerned about some situation in their locality about the virus spread. We talk about that. I think we share information more often about the awareness of the virus. Everyday new things are getting discovered and new things are getting are coming to the public information. We share it as often as we can. People constantly ask, how should we disinfect my home. Lots of people are disinfecting it in the wrong way, which is actually unhealthy. Some people say, you use baking soda with water to disinfect. If someone is, as I am saying, if you are eating it it’s not having a problem. It’s when you touch your—all these little bits of information we share for when we are talking with friends and family.

33.28

**I:** Can you share how your personal life has been affected by the virus?

**R:** This is a question I always want to answer. I am kind of proud that it hasn’t affected my personal life. I was already living in isolation. I was kind of happy that everyone else is in isolation. On another side of it is, because I live completely alone at the moment, I feel safe for myself and for others because I am not affecting somebody else because of my habits or my any problems or whatever. I am completely responsible for myself. I have a lot of time to look within, which I was obviously doing it from before, but now you have more time. I have not felt, until now, why can’t I go out. Why can’t I have not come to that yet. Maybe because I have lots of creative elements that I like thinking or I don't know, reading books or watching films, which I do. I am cooking more, which I never used to do.

**I:** I think that’s a positive take.

**R:** For me, I think most of the changes were positive. I started meditating more often, which I never used to do. For me, that is what I am going to tell you like my 90% of the change is because of this lockdown, has been positive.

**I:** That is really encouraging to hear.

**R:** I constantly give positive vibes to those who feel bad. Before, it was different, I was depressed. Before I was depressed.

**I:** Before the pandemic?

**R:** Yeah, like I had a depression problem. I was struggling. I was really struggling. This thing has completely changed it. Maybe I am a funny person.

**I:** Maybe you have the whole world with you now in a similar situation.

**R:** Maybe. This is the thing, I can see a lot of people stuck with families and they are not always happy. They are fighting. There are divorce and domestic violence on a very extreme that end. I know my friends were married and they are like constantly complaining to me, sometimes and all it was happening in my family where the others are not listening to the rules and they are doing this and tomorrow I might get the virus. I might die. All of this is happening. I am generally concerned about them as well. At the same time, I see that I am content within myself. My depression has gone.

**I:** I try it out. It’s a very encouraging thing to hear.

**R:** I really tell people that you should meditate. I have developed something interesting that I was not doing before. I noticed that I can see the sunset from my window. I look and I watch the sunset every day because every day it’s different. Every day the sky is different and I take pictures of it.

**I:** Very nice. Very encouraging. Can you tell me, don’t you have, you did touch on that. How is your daily life during this period? You said something about adjusting a bit.

**R:** The point that I would try to make is, there is no outside commitment that I have to attend to or outside commitments do not affect my timing. I can have my own time. If I want to stay awake all night and in the morning, I can do that without feeling guilty. I don’t have to go anywhere. I don’t have to, maintain any timing to go outside or anything. As a student, I already had that kind of habit before. Even then, if you had to do something outside, you had to go in a certain time. For me, apart from shopping for food or essentials, I don’t need to go out at all. It has simplified things. I can do more. People tell me that oh, I am getting bored. I am not getting bored. I am not getting enough time. I am really not getting enough time because I need more time to spend for my thesis. I am interested in so many other things that—

**I:** Hopefully, we will find time for your thesis as well. How do you think this pandemic will end?

**R:** I don’t think it will end in a very definitive way. It will stay for a while. The condition will extend for more than two years, I think. Unless some big development in the medical field comes up. I think it will go on for a while. What will happen is, people will slowly start to do their own things, but we have to change our behaviour to maintain certain rules and for ourselves and not for the government or not for anybody else, for ourselves. What I fear is, in the middle of it, there might be more outbreaks in between. When people don’t have the lockdown, they might just go out without thinking too much. That is a worry I have. Other than that, I think it is a very long-term thing. We have some traumas **[Unclear]** 40.29. I have said all the positive things, but definitely there will be some traumas like, for example, we will always think, my god, I have to wash my hands even though you don’t really have to. You will think about it. These behavioural changes. These traumas or paranoias will stay with us for very long.

**I:** That is the last question of that one, which is, how do you think we might be able to prevent any such pandemic in the future?

**R:** I think government should have, it is mainly government that must have these certain mandates, certain bills that the should think about from now on. They should have a set of action plans, economic, social, whatever. They should definitive action plans that they can implement very quickly whenever there is a future pandemic outbreak. We should be prepared. For example, I will give a very small example, when a lot of us were storing things because we were afraid that we won’t be able to go out. There is a disaster management thing that you might have to store water. If somehow, the water supply is cut off because of some deficiency, you need water to survive. We am saying government should understand that what is needed and what is essentially needed and have those things ready. For example, if a nation is attacked, the nation knows how to defend and they already have an army. Countries should now have this very serious **[Unclear]** 42.36 before them. It’s not like prevention, it’s more like immediate action plans can prevent a lot of outbreaks. A lot of countries were able to do that because they acted quickly. They knew what to do, not just quickly and they knew what to do. That is there. Another prevention would be, we have to slowly rely on organic living. We have to think. I was thinking it myself, this pandemic has given me a new kind of outlook about how I should lead my life in the future. Maybe in five years-time maybe I want to be in a place were I grow my own food, which is free from GMO, which is free from chemicals and will give me a better immunity and it will equip me to have a better healthy existence. That way we can probably prevent future outbreaks.

**I:** Thanks. This is very helpful. This is the end of part one. That section two which we will discuss, it’s focused on the South Asian community and their health issues. Similar as like we had been discussing. The focus is a little different here. The first question is, what do you think there are some health concern for people in the South Asian community during this pandemic and why?

44.24

**R:** I have seen some news reports where it’s been said that South Asians are getting more affected by it. I think I heard a bit more like, even black people are getting more affected by it as well. My understanding is, it could be social behaviour on one side. It could be social behaviour. Food habits and lifestyle as well. Although, as South Asians we already have a lot of good habits when we eat a lot of turmeric, cloves or all these things are really healthy. At the same time, we also have a lot of unhealthy habits. Even though we have those good habits, we don’t really implement it. Our ancient knowledge is there. We don’t implement it in our lives nowadays. We are living a very urban life. At the same time, the social thing is that people, there are any number of people in Asian houses, households. There are a number of people. There is more social cohesion rather, maybe, less distancing. Distancing is kind of frowned at in our culture. These can be a problem, I think. Can you remind me of the question again.

**I:** The question is, what do you think are some of the health concerns for people in the community?

**R:** There is another thing I learned, I read. I don't know how true it is scientifically. I think it is kind of true because I got it from a very reliable source. How much Vitamin D do we have in our body? Vitamin D has been known to be able to better resist, grow resistance against this virus. The thing is, if we don’t get enough sunlight, we won’t have enough Vitamin D. You can say that this is true for everyone and not just South Asians in this country. At the same time, if you have a darker skin you absorb less Vitamin D. You have to be longer outside, but not as long that you grow more tan because then you absorb less again in a situation. I think, I was discussing with a very good friend of mine and we decided that, okay, if we are going to get the sunlight from the outside it’s better to have it during the afternoon and when the sun is setting and you can have a longer period of time, soaking in the sun. This is one of the problems. I think there are some social, biological and behavioural problems that we have, which might be the reason behind our more health. There is another aspect I think people are also concerned about. I don't know how true it is. It could be neglect coming from social racism.

**I:** Can you elaborate on that?

**R:** There are some concerns that are appearing. I don’t buy it personally, because I haven’t seen it myself that in different countries like USA, black people are being discriminated against or brown people are being discriminated against and so they are not given enough attention in the medical health sector as the white population is. That could be another factor. I am not quite sure about this for UK or even for USA on a large scale. I really don’t think so, but there is a concern.

**I:** How do you think the South Asian community has specifically affected Coronavirus?

**R:** As in—

**I:** Health-wise or any other issues you think, anything like is there any particular way you think they could be more or less affected by the white people?

**R:** Yeah. I think I will go back to what I just said, it’s the social behaviour, I think. Also certain religious views they have across different faith systems that we have there are methods of congregating.

**I:** You think that it’s yes they are more at risk?

**R:** Yes, because they congregate more in those religious places. The white population also are religious, but then, I don’t really see people gathering in churches like that like some of the mosques. I am sure they are not gathering now. They were at some point. I think it’s a problem world I think. I have seen this issue worldwide among Muslim communities as well, Muslim Asian communities. Even Saudi Arabia, they haven’t lockdown on everything. They are not doing anything. The South Asians are. There are lots of religious figures like bollards. They are openly saying that this virus is not going to affect you because you are immune to it because you are immune because of God. This is the ignorance that is there among religious people. I am not saying everyone. There is a good section of people and a lot of people are not as educated. They would buy these. There was a very funny video. I will send it to you later. It’s really funny. They said that they had an interview with a Coronavirus and the Coronavirus told them that it’s not going to affect Muslims or even if you see some of the BGP leaders doing all these things and saying, go Corona, go. Okay, they are saying it harmless. If they are congregating to do that, it’s more harmful.

51.12

**I:** About the government directives, what we have like social distancing, travel ban and work from home, would you think it affects the South Asian community any differently than the white people?

**R:** It’s hard to say because individuals have different ways of reacting. In a way, yes, I would say, in a way it will affect South Asians differently in some ways because again because of our social cultural behaviour at home. If a person is trying to work from home sometimes it can be difficult for him or her with other family members around. It’s not a big problem, but it is a problem, sometimes.

**I:** How would you see these directives being experienced by the South Asians? The directives to stay home, to not gather, social distancing?

**R:** If somebody, if somebody is intelligent enough to understand that okay, this is for my own good. This is for my health, then it’s fine. A lot of people might be taking it in a different way. They are saying, we are not allowed to do it and so let’s do it more, that kind of thing; that is there. Is it more in South Asian communities and not in the other population? I doubt. It is across all—when I went out to get food, I see lots of different kinds of people of all backgrounds. We are not really listening to our directives. They are not suddenly going coughing away on the street. If they had a mask, without having something to cover their mouth. That is across all backgrounds. I don’t think it is much different among South Asian or other. There is certain social cultural behaviour patterns that might affect people differently. That is very subtle and that is very difficult to pinpoint.

**I:** To what extent do you feel people in the community feel able to access the healthcare during this crisis. Do you see any challenges. I remember you had been mentioning about some people not being able to access the facilities because of what you mention about racism just before. Do you feel that in any manner the South Asian community are being more able or less able to access the healthcare facilities?

**R:** I personally don’t think there is any difference between South Asians or anybody to access the healthcare. What I was saying was, there are concerns that once in the healthcare they might not be getting enough attention. There are many other patients as well. There might be a factor where people get discriminated, but I cannot say it because I haven’t seen it and I have not heard of it from people that I know. I will not say that, okay, this happens, but there is a concern in the media you can see some people talking about it or reporting about it in different countries, different western countries. If tomorrow I have a problem, I can call. I can call and get advice. I will get probably the same treatment or ask to do the same things that somebody else would be asked to do. I am staying here for a long time and I am not even a British citizen. I think I will be treated in the same way, unless I have a different experience.

55.37

**I:** Do you think people in the community trust government to take the right decision for them?

**R:** It’s a difficult thing to say. I think people do and they don’t. It’s very mixed. In general, people don’t believe in government. Even I don’t blindly believe any government. I try to be as objective as I can because if there is a rule that they are saying that I think in my own understanding that it’s good for me then I think it’s a good thing that the government is doing. Of course, if the government is delaying procedures or delaying certain things, obviously I will be complaining about it or not being very happy about it. I think the general perception among people is their very, there are anti government sentiments among people more than not.

**I:** Do you think it is any different than other white people?

**R:** No, it’s similar. I think it is similar. Similar, all these different populations also have similar apprehensions against the government or sometimes they support the government also. It is mixed.

**I:** To what extent do you think the South Asians, they understand what is the message given to them dealing how to deal with this crisis by the government and is there any way to improve the way the message reaches them?

57.37

**R:** For South Asians particularly?

**I:** For South Asians particularly. Is there any issues you think there are. Have any difficulty of them to access the healthcare messages.

**R:** Some people might have linguistic barriers. The information should be there in different languages like I think Bengali is very common and so is Hindi or Urdu or I don't know there are some other Arabic or whatever. Obviously, Arabic is not South Asian. That can be there. We can have those different languages. I think the government what they can do is they can include some of the religious and community organisations and we can include them to disseminate these kind of information. So that they have less suspicion against the government and they can integrate with each other more, I think. If a person believes in the mosque or the temple more, okay. If somebody from that mosque tells them okay you shouldn’t do this. They will listen to it more. Whether that means **[Unclear]** 59.10 that is a different question. It’s my perception that maybe this was a way to better communicate with them sometimes.

**I:** How would you think the government can do that, practically?

**R:** They can themselves reach out, in person, using their own infrastructure. They can reach out to these organisations, whether it’s religious or not. There are lots of community organisations, I am sure. There is so many South Asian communities. They are making charity organisations to help some of the others. I know personally, some of them who are helping people put somehow can get trapped in the lockdown and don’t have enough means to support themselves. The government can and I think they should be, they can get involved as well. It doesn’t have to be independent. I am not saying the government should control them. The government can reach out to them with their leaflets or whatever the soft copy information that they have. They can say, they can tell them that you should give this information to your community if you are a member. I think that can be done.

**I:** Finally, something to reflect, do you think anything in particular which has helped you or your community to deal with the crisis? Rituals or any other aspect, religious, spiritual community or anything you can think of which has given you the strength to deal with the crisis.

**R:** I will say the various things. There is one basic thing. I got some fire in my house, downstairs, where there is some community members they said, this is not South Asian really and that’s my locality related. They said that they will help out any medical needs to go out to the shop. It was very reassuring. Obviously, I don’t need that. It was reassuring that there is somebody. If tomorrow I fell ill and I can avail it. I actually know a friend of mine who is South Asian, she is not very well, like she has some complications. The NHS has given her food to her doorstep. That is amazing. Another thing I have seen, this is government level, but even personal level, individual level, some South Asians are reaching out to help others who don’t have enough food. Some people don’t have the money because they don’t, they are not having any work, they lost their work or something. That is happening. That is very positive. Intro community cohesion is helping. I cannot deny it. It is definitely helping. Personally, I think I am an extremely creative person though my creativity has helped me like I do singing like Indian classical singing. It is very meditative in itself. I do some meditation as well. It is, I never called it religious because it’s not. It helps your brain to function better, I would say. It definitely helps. I really recommend it to everybody. This has really given me a positive change like I am not depressed. I don’t take things negatively at all. Before too I was quite an optimistic person. But then, it is easy for you to get depressed when you are by yourself and having the stress of university and everything. I think meditation really helps me. Yoga, I think yoga was very very helpful. There is some breathing techniques that we have in yoga that is very helpful.

**I:** What we were saying? Creativity and meditation.

**R:** Creativity and meditation and yoga. I think these are very important. Every person is different. They can choose their own kind of creativity and they can choose kind of meditation techniques to do that. I do things that are from my culture and from my historical background. I connect with that more. Wherever there is some sort of Tiveton or some Indian chance or something like I do them for my meditation practice. These definitely have positive affects. And yoga definitely yoga, you know, those breathing techniques in yoga. These are long term. This is not only helping you now. It is a long-term thing. I think personally I think this is an excellent time to have, to grow a good habit. You have all the time and you can do it at a stretch for like 21 days to form a habit. You could do that.

**I:** We are at the end of our interview. Do you remember anything that we have missed or you want to add something to wrap up?

**R:** The only thing I want to say to wrap up is, I personally feel that people should internalise this whole thing and see what we are doing wrong to ourselves and to our environment, to our immediate environment. I am not saying it from a very, a spiritual thing. It’s a very general being aware of your surroundings and yourself. I think people should really think about maybe it is a chance for us globally to reboot and change our systems. The systems that are unhealthy or less equal. Right now, we are worried about those migrant workers. Were we worried about them before the pandemic? We were not.

**I:** Do you think it can change our attitudes?

**R:** It’s hard. We can only do it at an individual level first. We cannot expect that everyone else will change. If tomorrow, the pandemic is over they will go back to living their previous lives. Some of us can change and maybe that will change the society. I think government really play a very important role here. Like they are not passive and they can really have an important role here. If they can understand that this, which should not be completely greed or power oriented. We have to really think about development as a collective or at an individual level. Even like Anrad [sounds like] said the single person, the individual is the highest minority in any society. If you understand the individual, whoever it is from whatever background and if we can take it as an individual in a society then we can fix a lot of things. What I mean to say is, if you think of health and food sector, we can bring changes in that. That is the only thing and education, maybe, perhaps education, health, education and food. Only these three things can fix everything. That is it.

**I:** Thank you so much for your input.

**R:** I am really glad that I was able to say all these things.

**I:** I am happy to hear them. It has been a very good experience and I am ending the interview now.

**R:** Sure. Bye.

END OF INTERVIEW – 68 mins

Transcribed by Linda Pitt. Email: linda@laptopconfidential.com or laptop.confidential@btinternet.com - Telephone: 01964 612088
