# Supplementary material for: Mitigating the identity and health threat of COVID-19: Perspectives of middle-class South Asians living in the UK
Source: J Health Psychol. 2021 Jun 22;27(9):2147–60. doi: 10.1177/13591053211027626 (PMC9353968; doi:10.1177/13591053211027626)
Supplement: sj-docx-12-hpq-10.1177_13591053211027626 – for Mitigating the identity and health threat of COVID-19: Perspectives of middle-class South Asians living in the UK [file sj-docx-12-hpq-10.1177_13591053211027626.docx]

UK9_GroupB_USH_May20

Transcribed by: Sharmistha Chaudhuri

Researcher: Okay, so thank you, miss, for your consent again. I will start with the first question. What do you think is happening to the world?

Participant: Yeah, in the world everywhere the coronavirus is spreading and people are suffering. So we don't know when that is going to end. And then people are really frightened and you feel, even if the word is going to come to an end: everywhere people are suffering.

Researcher: So how would you say the world is coming to a halt? Like how, what things would be?

Participant: Oh, the people, because they coronavirus, they don't have a vaccination either. I that they spreading spreading pretty fastly everywhere in the world. So we don't know how that is going to end. So that's how we look at it. Maybe it spreads, spreads..no end to it!

Researcher: And what comes to your mind first when you think of coronavirus?

Participant: Yeah. You feel frightened if you don't know where the virus is!

Researcher: So do you feel frightened in that manner?

Participant: Yeah, of course. Yeah. We don't go out, we don't go out for shopping, we are frightened to touch. So it's really the frightening situation.

Participant: And how do you think that has affected the people in general?

Participant: Yeah, people in general, when you look at it, some people are really really worried and frightened. But not everyone, because the government is, um, putting lock down, but not all the people are listening to that. So I think not all of them are aware of the danger the world is in.

Researcher: Right, right. And how do you think this may have affected people's life in general? Looking at the global population.

Participant: It is really sad that the people who are living in small flats cannot get out. They're finding it difficult when, especially with the little children, so that they are really affected. And also when you think of the peoples' income, they can't go to work. So they are suffering. In some countries, people don't even have food, because they don't have money to get food. So people are really really affected.

Researcher: And um, when you think about coronavirus, how have you learned about coronavirus?

Participant (3:11): From all the noise, all that we know is, it is a virus. We don't know that it's coming from, that's it! Um, uh, who has it, who is positive, we don't know! This is really, really frightening to go out and especially if you are vulnerable, then you had it! If you are diabetic, if you are a heart patient, and if you have it, no survivors!

Researcher: Got it. And where have you learned about it, like from media, like newspaper, Facebook or any other place?

Participant: It is all in the news, they keep on telling in the news. People also spread news in WhatsApp, but they add openly saying in the news. So always watch the news.

Researcher: So which news channel you generally watch?

Participant: We watch BBC and ITV.

Researcher: BBC. How much, do you get information about this from WhatsApp or Facebook?

Participant: Yeah. I mean, I don't have a Facebook, but in WhatsApp people send things out to say, you can avoid, you can avoid getting this by eating this and that and all sort of thing. But mostly we get the information through the news.

Researcher: News and, and how much you read about messages, you get in the WhatsApp and how much you trust them?

Participant: And I used to read WhatsApp and the thing about this virus, but now I don't bother because who knows, whether they're dealing the right thing, when you get it through the WhatsApp, you can't trust. So I don't bother to read it. It sometimes say, the doctors speak. How do you know? How you trust them? So I don't, I don't know, I don't trust the WhatsApp, only go by the news.

Researcher: Got it. And, can you tell us a bit about, what do you know about coronavirus?

Participant: What I normally know is that it is spreading. Say, someone coughs, its' germs go everywhere, whoever is near, you had it. When you cough in the objects, in shopping things, it is in the shopping things; you have to clean it with disinfectants. You should not touch it, you should have to leave it, all sort of, and that's the dangerous thing. you know, whether we have cleaned everything enough!

Researcher: And, um, in your opinion, how did coronavirus appear?

Participant: Yeah, we heard this happened in China, in that one laboratory. Probably when they did some research, something leaked, they say, probably they did some experiment. This virus leaked and spread to people and then people travelled all over the world and then they bring it everywhere. So that's how it started. So I think, yes.

Researcher (6:54): So, why do you think they would be doing any research with this Vitus?

Participant: Yeah, but you don't know! There are so many theories. Some theories say, it is like a war they do. They deliberately did this thing. Well, that is what they say! Or maybe, they're trying to find some medicine or something and this happened. We don't know what is the truth?

Researcher: So when do you say they, who do you mean?

Participant (7:32) :Whoever is in the lab? The government, who is doing it? You don't know in what circumstances this happened. If they deliberately, they're are doing this for a war or deliberately or was it an accident..

Researcher: Have you heard of any other source of coronavirus?

Participant: Not really. Those from China, ..that's all we heard.

Researcher: Right, right. How do you think coronavirus appeared in the UK?

Participant: Yeah. People travelled. People who, who, meet people in China, they travelled or people who travelled abroad, maybe Italy, people with coronavirus in Italy and they spread. They got it from there and taking back and brought it here.

Researcher: Correct. And, um, since the beginning of the pandemic, have your perception about coronavirus changed?

Participant: Coronavirus increased! In the beginning, we heard that 2-3 people had, and they would be asked to isolate. And then suddenly it does become a pandemic, everybody, lots of people, they are admitted, they are dying, specifically the vulnerable, the elderly.

Researcher: In the beginning, did you think that it would have gone this way?

Participant: Yes. Because in China, people were dying. Yeah. They have locked down in China. So when they came here we were frightened to think what would happen, but like in China, it's just like that in here as well.

Researcher: So is your understanding, your perception is the same as in, like maybe at the end of February, March and as what you are thinking now?

Participant: Yeah, yeah, yeah. We were thinking..because now they are really relaxing, the lockdown, but the death threat is not going down. They, they are relaxing, because it looks like because of the economic situation, because people need money. They need to go to work, then only they can earn. So that's why are relaxing, but the frightening part is, when they relax, people will, they will mix, everybody mix together and then it get worse. That's the worrying thing.

Researcher: Right. And do you think these pandemic is different to others which happened in the past?

Participant: I don't..we never came across, but they say they had Spanish flu in the 1930s or so. . We don't know, but it did affect a lot of people they say, because we are not aware of the pandemic, we don't know!

Researcher (11:00) :So, if you like had any idea of pandemic, how same or how different this is, to you ?

Participant: No. We never had- we only seen in films. But not in reality.

Researcher: Right. So it's, it's we have never seen these before, right?

Participant: No, we never, never!

Researcher (11:27):And what would you say about your government's response to the pandemic?

Participant :Yeah, I think the government, they are not doing much because, when it started, they never took it very seriously. They let the people isolate themselves, they did not put them in quarantine. They didn't put them in for a long time. So I think they should have done it, long long ago. They should have tested, put when they found out, when they are coming from places they had been with coronavirus. They should have been isolated- government should have made them! They should have brought the lock down a bit earlier. Then they would have avoided so much deaths. And these elderly people, who are in the care homes, they are left to die, that is unfair. But I think they are more worried about the economic situation. Poor people, poor NHS people are dying! They don't have protective with PPE, so they have to face patients without protection for themselves.

Researcher: And how do you think, when you say economically affecting people, how do you think it is happening?

Participant: Because people are not going to work, so they have no income. They're not, uh, nothing is happening, everything is sealed at the moment. So as a result people don't have money to spend or eat. Some people don't even have food, they can't buy food.

Researcher: So government also can't do much, but at least they should have stopped or no, done something earlier then leaving it this bit later.

Researcher: Right. And what was the information about coronavirus that most surprised you?

Participant: Yeah, because people are just dying, when people who get it, especially when elderly people get it and that's it. They go to hospital and they hardly ever come back. Going to the hospital is frightening. Especially this part of the world. We think they have everything, but in other parts where they say, developed countries, they are managing it, but they are not doing it here.

Researcher: Got it. And, um, when you discuss about coronavirus with other people, like your friends or family, what do you mostly talk about?

Participant: All we talk about is how long we are going to be like this! Will we ever see each other, because we don't know what's going to happen.

Researcher: Is there any other things you discuss?

Participant (14:54):There's no way we can go, we cannot go on holidays anymore. unless they get a vaccination or another thing. It, it's not safe to fly. And we don't know, when the flights will start! Because so many flights, uh, they, they stopped it.

Researcher: Correct! And can you share how your personal life has been affected by the pandemic?

Participant (15:24):Yeah! We cannot go out, we cannot see our children. Children can't come to us to see us and it's worrying, but they, they, they, at the moment they are working from home. But if they are asked to go to work then we will be worried, how they are going to go to work. But for us, and you wouldn't get food and things for two to three weeks, we couldn't get hold of any. Only friends helped us. We couldn't get any delivery from online shopping. And most of it is prescription medication. They won't deliver it either. so those are all sort of inconvenience for us. Specially we are elderly, so we are worried to get out.

Researcher: Correct. So that's the food delivery was the difficult part for you?

Participant: Yeah, the prescription, medication. Basically getting medication from the Boots and the food items, because it's online. It's very difficult to get a slot.

Researcher: And, though it is difficult time now. But do you see any positive effect of this time on your life?

Participant: I don't think so. Because really, every day we, are worried. We try to think positively. Everything will come to an end, but not! But that's, at least for us, we are retired and it's okay. We can remain in the house, but people go to work. And people with children, their situation is worse.

Researcher: During this pandemic period, how your personal daily life is and is that any different from what you did earlier?

Participant: Oh, of course. We couldn't go to do any work. Any part time work, we couldn't do it. We couldn't go on holidays. We couldn't visit friends. So yeah. All stuck, everything is changed.

Researcher: And how would you describe a typical day for your life of your life?

Participant: After the lockdown, only but how do you spend the time! I spend, I spend time. I do some prayers, that way I take more prayers than what I used to do. So do the prayers, then read: that's all, then cook, cooking is not much, because you don't, you can't get much to cook! So we just do the readings, tidy, tidy and then get rid of the things that you don't need in the house and stuff, but did not have time to sort the things to throw..otherwise, just eat, pray and cook.

Researcher: Other than going out and your life in the house and the daily chores, are they any different than what you had been doing before?

Participant: Yeah. Yeah, because I used to teach, I can't do that anymore. Because you cannot go out. So, so you, you lost, you know, it's a pleasure teaching and getting ...when children are doing well, you feel happy, this and that, they are all gone! And also, that occupies us as well! getting the work ready, teaching. And now that that's gone. Don't know whether it will start again, because of the virus! unless this virus, this deadly virus is gone, we don't know how that's going to end. So now we ask ourselves, we have to change our lifestyles, reading, praying, cleaning, cooking, this is all we can do.

Researcher (19:51):Do you think this virus has taught us anything?

Participant: Not really! But what this virus has taught is, for the virus, it does not have any discrimination. Whether you are poor or rich, you had it. if you have to come to me .. in that way it is a lesson to people who thinks, oh yeah, yeah, yeah, we are this and that. That's the only thing they would have learned the lesson..but in a high price. So it is really good.

Researcher: How, who do you think this pandemic will end?

Participant: That's a bit difficult to say, because people who are in the A&E, the death rate are nearly the same. It doesn't change. They're just...using the lock down because it looks like it's because of the economic reset. So once they relax this lock down, people are going to get infected more! They only said it is the elderly, but now the children are getting affected as well. So the parents will be frightened to send the children to school. And, if it spreads to children and the adults, then it will get worse. So we don't know how that they are going to just stop this, but you don't know. It's difficult to predict.

Researcher: And how do you think, how do you think we might be able to prevent further pandemics in future?

Participant: Yeah. The lessons that we have come to learn is that if we come to know that it is in another country, then whoever comes from the other part of the world should be tested or quarantined. And then to find out if they are developing any symptoms, they should not release these people into the country. Don't just leave them to say, go, you stay home for a while. People are not going to listen to that whether they are going to stay in their house or not. So government should have, should take extra care when they find out any pandemic going anywhere else, they should take extra precaution so that people don't bring that into the country. Now it's too late with this.

Researcher: And any other thing you can think of relating to our lifestyle or something?

Participant: Okay. Not really, because it's a sudden change. People won't be people..they like to socialize, go to work, go to park. They like to do the normal things, but what they can't change is staying indoors or just go to school and come home and stay in those. That doesn't work, there's no life for people, children! So we don't know what's going to happen.

**Part II.**

Researcher (23:06): Right. So, miss, we have finished the part one of the interview. So now we would move to that part two. In part two, its’ focus is on the South Asian community. And yourself as a member of the community, and you would experience with it. So what do you think are some of the health concerns for people in the South Asian community during the pandemic and why?

Participant: You mean whoever living here?

Researcher: Generally among South Asians living here.

Participant (23:53): Yeah, for the people living here, most of the Asians, they are..they are.. they, they, they always aim to in their life for some reason, they like to be a doctor. So most of the Asians, in their careers want to become doctors and they achieved it. But even though they had done it, they are the one in the front, and they are the one, they have to suffer now. They are not given proper PPE. So poor children who studied, worked hard, and they're trying to help, but the government should help them with giving PPE and the oversight was new ones. They make the foray to go up to the front. So they have, they are forced to, in the front and get the disease. So many educated foreign people have died. So that's the loss.

Researcher: So when you see front, what are the professions, you mean?

Participant: Um, that means, if you going to deal with a patient, they are sending the- that was in the news- sending the foreigner doctors to deal with that, the patients. So they are the one who are getting the contact. Even if this is correct or wrong, most of the NHS people are foreigners..Asians. So they are the one who are trying to fight the corona patients, and are the one who are affected. So that is a bit sad. Yeah.

Researcher: That's something to think about. And do you think, uh, really that, NHS or other big organizations are choosing them on purpose?

Participant: Mostly, Asian people like to study, they like to help, so they choose their career as doctors. Being a doctor is a difficult job. You have to be really caring for others. Because they like to do that and study, and then become doctors. And it's fine. If they give these PPE, then it's fine. Then dealing with the patients. But they are provided. So for the poor families...

Researcher: Apart from the profession of medical profession, how do you see these people now, the South Asian people may have got affected?

Participant: Yes. And the chances are of more affected, because they live in extended families. They live together. The parents live together, in some cultures, the children look after the parents. The children have to travel, so when the children come home, they're mixing with the parents who are elderly. So that affects the parents as well. Because that is their culture. But when you take Western culture, they are on their own. And also, they're on their own, they are also struggling nobody to help. So whether you are Western or you are Asian, it is the same problem, but here they are really always worried that they shouldn't be going near their bed. And at this pandemic, that is an advantage, for the family living together.

Researcher: And how about like people working, work wise? I mean, I know you mentioned about the doctors or any other, other profession in specific you see that has any specific effect on the South Asian people?

Participant: South Asian people, whoever are educated, they are the one who are running the job, they are the one working, mostly in professional jobs. Whoever are city educated, are holding higher positions. If you think, if you take the parliament, itself, the finance secretary, the chancellor, home secretary, and even the other ones- the judge- I can't think of her name- she is an Asian, somebody from Goa. So they are the people in the government. What happened to people over here? So it looks like if you are here, educated, that is getting you the job. That is fine. However, if you are not educated here it looks like they are being discriminated getting a job here. But if you have the education, you are fine, if not, you had it, it is worse.

Researcher (29:41):And, um, how your own family has been affected by this coronavirus?

Participant (29:49):For us, in our case, with our children, I know they can't go out, they are working from home and also with the shopping they're struggling. With the shopping, they can't get, they have to go out for a bit of shopping and then they are worried. And we say, don't go out, but they have to go out to get shopping. And also this is our children face. Though they can work from home, these difficulties, they still have to face. And for us, I know we are retired, but we can't get out. And as I mentioned, you have to get medicine, you have to get out. You have to ask the neighbours to get the medicine for us. Luckily, we have good neighbours, they are helping. But if nobody were there to help, then you had it! And also with the shopping, also for first three weeks, we couldn't get any shopping. Only the neighbours bought it for us. So good. Otherwise we would have been struggling as well, but it really affected. Yeah.

Researcher: Got it. And the government has introduced a lot of different measures, like walk from home, social distancing restriction or traveling, etc. So how, are there any specific difficulties that you think South Asian community are facing in relation to these measures?

Participant: Um, I don't think so because it fits for everybody. So everybody's going through the same with the government regulations that it doesn't affect them differently. Only thing is when they are in a group, in the same house, they are affected. The elderly are worried, the children are worried. Otherwise, same rule applies to everybody. So these days, you know, South Asian, Western or British or English or whoever is all the same. Yeah.

Researcher: And, to what extent do you think these, um, expedience of the lock down are similar or different, as experienced by the South Asian community compared to the white British?

Participant: I think they all feel the same. Everybody, the struggle they go through is same, only apart from these NHS people and their families, their families would be sad because they are not getting the proper protective clothing to do their jobs. Apart from that everybody is in the same boat, everybody's worry is the same. It's not particularly any different to the South Asian people. Probably one might say, if we want to visit to our relatives or some people who live alone at home and they cannot go, so that is boring for them. But the people who don't have anybody, then it is the same, but for families who have parents, and the parents are not well or so, they can't go to visit them, to see them. So that is sad. That is the only difference between the people who live here permanently and people who have relatives abroad. Yeah.

Researcher: To what extent do you feel that people in the community are able to access the healthcare facilities during this crisis?

Participant: The health care is here. They didn't want anybody to go to hospital all these days. So people were worried and frightened and now the NHS says, oh, you can come to hospital, come and get this done, get that done. But I don't think people feel comfortable to go. in my case, I would rather stay with the problem, than going to the hospitals and getting more problems. That's how people would feel. For me, that's what I would think. I wouldn't go. Even if I have a full thing or anything, I would rather stay home and recover rather than going to the hospital. Because at this moment, if you go to hospital, you don't know what you are into.

Researcher (34:32):Do you think that South Asian community are in any way, has any difficulty to access the facility?

Participant: I don't think so. Because they, you know, NHS, they don't have any discrimination actually having go to the GP. They said that, I don't think so. They are suffering in that part. I wouldn't say that. Yeah. Yeah. Everybody has the same equal opportunity to access. Yeah. But people are worried. People wouldn't go. That's the thing.

Researcher: Do you think the people in the South Asian community trust the government, that they are making the right decision for them?

Participant: I would say, it is all the same! The government doesn't purposely discriminate the South Asian community, everybody's affected in the same way. Only what is not right is for the people who are working with the patients. Most of them are Asians and they, they are not giving the PPE because government doesn't have it either, but they should somehow, should I get hold of it? That is so worrying. I don't think they deliberately discriminate South Asians.

Researcher: And to what extend do you think the South Asian people, they understand the message around the health surrounding coronavirus.

Participant: I think they are aware! Nowadays there's a lot of media, all these WhatsApp and all these face time, then communicate to anybody through the network. They will find out. And everybody has an understanding of what's going on and the how much it's affecting people. Everybody will know. Because there is communication. But if you take some part, or other, like South Africa, the communication is not like as in here, then they may not be aware, but here everybody knows everything.

Researcher: So do you have any, suggestion, that how the message to the South Asian community could be improved or can reach more, all the directives.

Participant: But as I said, I don't think so. Do they need to be told more than what they are telling? everybody has access to media. Everybody has television, everybody can speak English. Everybody can understand that they don't have difficulty specifically to put the message across. Yeah. Because of media is bad and they're trying their best everybody to understand. So yeah. I wouldn't think that they are deprived of getting any message.

Researcher: Right. And, finally, what do you think has helped you and the South Asian community to deal with the crisis?

Participant: I don't think, anybody got any help- apart from having a good neighbourhood people like us! If people are in strange places where they don't have anybody, then, then they will suffer a lot. So it depends on which location you are staying and how much help you are getting from your neighbours and friends, it depend on that. But personally, for us, yeah, because we have neighbours even to get the medicine or shopping. For first three weeks we couldn't get anything. The neighbours helped us; friends helped us. So that was ok. It depends on the people, where they are isolated.

Researcher (39:07):And any other strength you see in the South Asian community, which can help with the crisis as we are going through.

Participant: I don't know what to say, but you mean how they can contribute?

Researcher: Not contribute, how they're coping with the crisis?

Participant: Probably they have close families; the family members might help each other. Because with Asian community, normally they, they have a quite good bonding. They care get about their friends or families. They can look after it. They have some caring towards each other; friends, they would help each other. That's how I think they would somehow get help.

Researcher: Right. So it was great talking to you about this again. And so thank you. And do you think anything we have not mentioned that you want to mention, or do you have any questions?

Participant: Yeah. Not really. But if they ask any ideas from me, I would say keep the lock down. But in the other hand, government cannot do that. But I think they shouldn't send the children to school either and I don't know when they're going to send the little ones first, state by state. I don't think parents will be happy to send the children to school at this stage. So they shouldn't do that.

Researcher: Thank you so much!

Researcher (40:48):Yes. Yes. Hopefully it will reach the audience someday. Okay. Thank you so much. Thank you.
