# Supplementary material for: Mitigating the identity and health threat of COVID-19: Perspectives of middle-class South Asians living in the UK
Source: J Health Psychol. 2021 Jun 22;27(9):2147–60. doi: 10.1177/13591053211027626 (PMC9353968; doi:10.1177/13591053211027626)
Supplement: sj-docx-13-hpq-10.1177_13591053211027626 – for Mitigating the identity and health threat of COVID-19: Perspectives of middle-class South Asians living in the UK [file sj-docx-13-hpq-10.1177_13591053211027626.docx]

**UK10-groupb-ana-may20**

56 minutes

Transcription by: Sharmistha Chaudhuri

**Part-1**

Researcher: Thank you for your participation in the study. I will start with the question, what do you think is happening to the world?

Participant: Glorious Uncertainty, but it is dangerous!

Researcher: Right, right. That is the uncertainty you are thinking that is happening to the world. Why do you think it is uncertain?

Participant: Because no one knows what it is, how it started and how it could be stopped.

Researcher: Right, right. And what comes to your mind when you think of coronavirus?

Participant: Some sort of nightmare. We don't know what it is, but something is coming in, which is not very good for you.

Researcher: Correct. And anything else? Any image or any visual thing you can see, about coronavirus?

Participant: Plenty, but it is very difficult to say what it is!

Researcher: Right. It's mostly the nightmarish feeling that you get.

Participant: Exactly!

Researcher: Right. And how do you think coronavirus has affected the people in general?

Participant: Everybody is affected, so it stopped everybody's daily activities, normal movement and everything.

Researcher: Right. So when you say everybody, who are they?

Participant: Like person from six year old and 106 year old.

Researcher: Right, right. Age no bar! And anything else about the category of the individual or anything you can see are affecting them ?

Participant (2:06): The thing is, people who are vulnerable, they are expected to be affected quickly.

Researcher: When you say vulnerable, what do you mean by that?

Participant: I will go by the example, somebody has got cold or cough. The old men has some of the old defect. That's what I mean.

Researcher: That’s right. So, underlying any health condition. And how, how have you learned about the coronavirus?

Participant: Well, unfortunately, all from the media. I had no knowledge about it prior to it happening.

Researcher: And from the media, so which media do you follow?

Participant: To start with, the tv channels and newspapers.

Researcher: What are the newspapers you follow? What television news you watch?

Participant: ITV and Sky news.

Researcher: And do you follow any other social media source? WhatsApp?

Participant: No.

Researcher: Okay. Okay. So you watch, and all the information you gather are based on that television news. Right?

Participant: Not on that way. I use it, my television news watching time is about an hour, taking 2-3 times, an hour and half in a day.

Researcher: Right. So you, you keep yourself updated that way. Do you go to any website?

Participant: No.

Researcher: Right. And could you tell us a bit about what do you know about coronavirus?

Participant: I didn't know anything about before that, but it appeared that it is something which must not take lightly and stay away for the possible area of infection.

Researcher: Right. Right. So which, in your opinion are the possible areas?

Participant: Places like which has been in touch with others who had it, or picking up the germ from the gatherings or shopping and that sort of things.

Researcher: So it is mainly from large gatherings. And anything else, you know, like how it affects the health?

Participant: (5:22) I mean, more fear, because of the phenomenon, very difficult to know because we always get so scared and that's why we haven't got the judgment. Our usual judgement does not always act there.

Researcher: That's a very good point. That's because you are scared, do you think?

Participant: Always, sometimes the logical mind does not make sense!

Researcher: So, does that happen to you?

Participant: I am afraid it doesn't, we are so much used to lots of varieties of information, but it could have easily happened.

Researcher: Right! So it can easily happen. Correct. And this is a very, very good point actually. And in your opinion. How did coronavirus appear?

Participant: Well, lots of things like couple of thousand, hundred years ago, when plague came in, the scientists couldn't find out the reason. So they put it on the rat. Rats can't come back and say, sorry, we didn't do it! In case of Spanish, it is already marked- as Spanish flu. Here, no one knows, somebody or some area or some sort of organization wanted to put it on bat. But according to me, bat is so little in use, even in China, I don't think that it could spread from that. And bat can't really come back and say, sorry, we didn't do it.

Researcher: Correct! So the, the source is not known in your opinion?

Participant: No, completely unknown!

Researcher :Completely unknown. And would you put any specific geographical area for its source?

Participant: No, because it happened to so many places, like I thought it Asia, South Asia population, but it has happened to the pure English areas as well.

Researcher: Yes. So that's a very interesting point of view.

Participant: You can't earmark any nation, any particular community or particular geographical area.

Researcher: Right. And how do you think it appeared in UK?

Participant: Well, UK is a place, where everybody is coming from everywhere and going from here to the other places. So if it is airborne, it could come easily and it could come to the other people connection or some sort of communication, then it could come from there as well.

Researcher (8:26):Right. And one question to think about like, since the beginning of the pandemic, have your perception about coronavirus changed.

Participant: My wife is very, very intelligent!

Researcher: I'm sure she is!

Participant: I have no way of escaping her, so long I am married, I have to listen to her. And she picks up from various sources, doctors, word of mouth, media, newspaper, and all sorts of things, you know. She is much more sociable than I am.

Researcher: But according to you, is your perception the same as it began and as it is now?

Participant: Yes. Yeah.

Researcher: So do you think the pandemic is any different from what we had in the past?

Participant: Think about 100 years ago- We have got very, very little knowledge, even two years ago, plague. Only thing kind bury the dead body and all sort of thing. And I also know, still, still in Brockley, East London, there is a vast area which is still, they are not sure whether there is germ or not, even after two years. So, what they did, they made it a park, instead of habitat, residential area.

Researcher: I see, I didn't know about that. So we were talking about whether this pandemic is any different from what happened in the past, right? So you think it's not, more or less similar. What are the main, main things or pandemic that you see when you think about pandemic, what are they?

Participant: People die. Without knowing what it is for.

Researcher: Death, isn't it? And what do you think about your government's response to the pandemic?

Participant: Well, they tried. Because government in UK is the combination of people, representative, elected from us. So their aim is try to stop it for the community. So they tried their best, you know, but they can't do anything, which is not known.

Researcher: So how much you agree or disagree or what do you think, how the policies they have taken are at the best interest of the community?

Participant: The thing is that that is all that there will be all differences of opinion. If you look from different angles, according to me that is best, and I'm quite happy with the progress and approach, which is the main thing.

Researcher: What was the information about coronavirus that most surprised you?

Participant: Well, the thing is that it happened in countries like here. But one happened 100 years ago, the ethical and one before that was 200 year ago. So it is difficult to have an idea around it.

Researcher: So did any of them surprised you, any of the information about coronavirus?

Participant (13:14): No, because it's so unknown in order to be surprised, you have to have a bit of a knowledge. We have no knowledge. Yeah. I think that is something had happened. I am not really a true believer of God in that sense, but I think it is heaven sent, in a reverse way. Heaven sent generally mean boom or something good. But this is something worse. No one is responsible for it. No one knows the reason of it.

Researcher: So it is just sent by an unknown force. Right?

Participant: Yes. In case of rat, 200 years ago, they found out something, it's got different sizes and they move around countries; town mouse, home mouse. Now it has got so many sizes and it moves so many places. And the most important thing they can't argue you back because it was in the name of that.

Researcher: Correct! And if you discuss coronavirus over phone, or talk about with your relative, to your friend, family. What do you discuss most about coronavirus?

Participant: I say, talk to my wife. Sorry! (laughs) She knows more than I do. She is beating me online. That's why they she is more knowledgeable and intelligent!

Researcher: I hundred percent agree with you. I couldn't agree more, but say, you speaking, when you are speaking to your son, what do you say these days generally?

Participant: Surprisingly, he knows more than I do. He's an accountant. And he has got knowledge from media and everywhere. And that's why he knows much more. Sometimes I say, you must not do that. You must not do it. And in my age, I took it casually. Only thing I need to be pushed by the corona to pass the border.

Researcher: So you talk about health then, like what to do, what not to do?

Participant: Hmm..Dos and don'ts.

Researcher: And how has it your own personal life has been effected by this pandemic?

Participant: Because I can go out and talk with people: my activity like rotary and all other. I have to stay at home. That is more or less. And also the people I meet and we meet socially on a weekly basis. It's not happening.

Researcher: So how does this make you feel then?

Participant: In Bengali, horibol (translation: The end).

Researcher: Do you have any English for that?

Participant: Yeah, horrible! (laughs).

Researcher (17:22): That's a good one. So, now do you see any positive side of this coronavirus in your life?

Participant: I mean, I can see one thing. People are very very cautious about it. They do whatever they can do and they have to do. No one is ignoring anything as such and hopefully it will gradually reduce. It's not atom bomb, like we do not know how much power, in hydrogen bomb, nitrogen bomb, the scientists know the power.

Researcher: Correct.

Participant: And here they don't know!

Researcher: Correct! So what changes, when you say changes, what are they like?

Participant : They are like.. make people much more cautious. Make people are much more, believe me or not, sociable. Because each one is trying to think of others problem and realizing, and that sort of thing. Mutual understanding is better, much much, growing much more.

Researcher: So has the sociability, which you said that has become more, has affected you, like people are reaching out to you or you are interacting or anything like that happened?

Participant: Not in that way because unfortunately, or fortunately I mix up with very very knowledgeable and understanding people.

Researcher: Right.

Participant: So in my society here, I haven't got any ignorant or what should I say, arguing without reason- these are the sort of thing that all visible in the world.

Researcher: Correct! So they have...how they are different from any other people?

Participant: Well, the thing is that most of the people are well educated and well conversant. They're very knowledgeable. If you look at common people in the shopping area, market or something. They are big..above the rank.

Researcher: Right. So how does that help them do cope with the crisis?

Participant: Because they knew it. Where you have to push someone, make them understand, and here they are telling you because they know the information and they can grasp it almost strictly. In that way it is easier for them.

Researcher: Correct! And could you please tell us a bit about how your daily life is like during the pandemic?

Participant (20:23) Well, the thing is, because of my type of work, it didn't affect 100%. Because I got lots of things through email, WhatsApp and computer and that sort of thing, and things could come to me through post. My job is to process it because I work from my own place and that thing hasn't changed at all. So it gives me the opportunity to give advice to my clients, through that- don't do it, don't care about Inland Revenue letter, I may also say that direct that to me, that sort of thing. So I am also, more or less, giving some sort of positive power to them.

Researcher: Correct! And could you please tell us a bit about how your daily life is like during the pandemic?

Participant: Well, the thing is, because of my type of work, it didn't affect 100%. Because I got lots of things through email, WhatsApp and computer and that sort of thing, and things could come to me through post. My job is to process it because I work from my own place and that thing hasn't changed at all. So it gives me the opportunity to give advice to my clients, through that- don't do it, don't care about Inland Revenue letter, I may also say that direct that to me, that sort of thing. So I am also, more or less, giving some sort of positive power to them.

Researcher: Do you see any change in your daily life?

Participant: Yes, because I can't talk to anyone. My telephone conversation has grown quite a lot and I'm watching much more Bengali films, Rabindra sangeet (songs written by Rabindranath Tagore) than I did before! I of course enjoy it, but not all the cinema, or the films are not good. And in case of song, I choose it, that's why I enjoy it.

Researcher: So having some quiet time, sometime for yourself.

Participant: Yeah. Also the English, the English classics. They're really, really classical. Classics like Quo Vadis, 10 commandments, Ben Hur, that are sort of things. These sort things I watched ages ago, and now I am almost revising it. You can say it is a blessing in disguise.

Researcher: How do you think this pandemic will end?

Participant: No one knows, it is endless! No, it will end- the question is, we have got very little experience to follow it up because what happened two years, 200 years ago, we have no idea. And so far our knowledge goes, it is gone automatically. The question is how long and how, that answer is not there.

Researcher: How do you think that we might be able to prevent further pandemic like that in future?

Participant: Oh, it will not happen in 100 years, you will not be there to prevent it! You will not be there.

Researcher: I see. So you see that, we hope that nothing comes in, but how can we actually prevent?

Participant: We can only talk from experience, ok?

Researcher: Right. So you think that we may blessed enough, not to have another pandemic, like such, in our lifetime!

Participant: Because you have to live for hundred more years, which I think not possible.

Researcher: That is hopeful, that is very much hopeful!

Participant: Our next generation, generation, after...It takes 5 generations..4-5 generations to recoup it, that's why, once it is over, We don't need to think of it.

Researcher: Okay.

Participant: Now religiously do, what is needed. My wife is there, to put me the line. Sometimes I am derailed.

Part II. (25:00)

Researcher: Right. So, we have ended our first part. In the second part, the focus would be on the South Asian community. Okay. And I will just start with the question, like, what do you think are some of the health concerns for people in your community during the pandemic?

Participant: In this country, you can see what they're doing. So that's why it's not that no one is ignoring. But I have a feeling that some corners like Islam and others, they believe more in Allah. But again, rather, I am not talking from any experience I'm talking from feeling.

Researcher: Go on, you're allowed!

Participant: And that's what it is you know! If Allah is there to do such a thing, who we are, but the human beings. But in here, mosques, people are still going!

Researcher: Okay. So you think that they are still visiting the mosques?

Participant: Yeah. It's only 10% to 20% as such you know!

Researcher: You think that is affecting the people?

Participant: Not really because they are some sort of immune.

Researcher: And do you see any health conditions for which Asian community maybe more at risk?

Participant: I mean, generally my mixing up is only with Bengali. So I can't give any really reasonable experience about them as such.

Researcher: Right, right. And how do you think that South Asian community has been specifically affected by the coronavirus?

Participant: I mean, they are separate example. Bangladeshi community is so difficult to come out with results or anything, they are very much secretive about it. So you won't get the full picture!

Researcher: So they are secretive about what, for example?

Participant: Whatever happens they are secretive about it. Because this is about Allah, Allah..

Researcher: So it's the act of God or something?

Participant: Yeah. I don't have that much of knowledge about them. I know about the Gujrati, a little bit Gujrati, but they are well advance. Gujaratis are very very law abiding, this and that. I have some Gujrati friends, we talk over telephones, we always observe the bindings and regulations and rules, what is there.

Researcher: Okay. So you say South Asians are generally law abiding?

Participant: Yes. Yeah.

Researcher: Right. And that can help with the crisis.

Participant: Yeah. The problem in this country is that after staying so many years and after being British citizen after all these thing, and loss of our own thing, gone. So we are much more western and we are much more logical, we are much more educated. And when we came here, 50 years, 60 years ago, so that particular thing about Asian community is not that much applicable to us. I have a reservation about the Bangladeshi a little bit because they own all these sort of Bengali restaurant, Bengali chains as such. They are not still as rich like on the Punjabi, Gujrati and other. And that's why they are also not spread, their outlook has not changed that far. It changed, but not to that extent. For Gujrati, there's not much of a difference of opinion- particularly in Punjabi, Gujrati and English. In case of Punjabi, no Allah etc.! Regarding the shop and other in this country, we have to very very particular, because of the inspection and that.

Researcher: So do you think there is a role of religion in it, anywhere?

Participant( 30:00) And it's a way of life for the people. It happens for the Muslims as well. If you take the religion out of it, the business community, which do all this 'sukti-mach' (translation- small business) business, this and that. Does not matter which community they are or which religion they are from, they are all the same.

A Bangladeshi accountant or a Bangladeshi lawyer, who are as good as an English lawyer or a Bengali lawyer or a Gujrati lawyer as such but as you go down, at the bottom area, there are a lot of things. And after staying here for that long, you picked up lots and lots of habit. Though they are not staying, 10 people under the same roof and that sort of thing, those early days are gone. Financially, we have improved ourselves quite a lot. Son of a Bengali shopkeeper, they will become a doctor or a barrister, or lawyer, and that sort of thing you know. So lot of them are going up, the social ladder you can call it.

Researcher: So do you think the community was not living in the same way when you came in, in the beginning?

Participant: Improved quite a lot. The thing is that, that generation which came here, they said we came here, whatever it is, but we needed money; not only from India, but from Bangladesh as well. But after that need has gone, after 50 years, like their sons, the next generation, they don't need the money. It automatically came to them through the process of business or anything, still the percentage, which is much lower that what used to be, and the ways have improved as well.

Researcher: And that, do you think, have any relation with how you look at the coronavirus?

Participant: I think we do. They have improved as well. Because ten people under one roof and that sort of thing and infection, it's not there anymore. Not to that extent. They have improved quite a lot.

Researcher: Do you think the Asian community are maybe more affected by the pandemic than the white, British people?

Participant: I don't think so. No way. Because the way of life is not Asian hundred percent anymore. Like religion, going to gurudwara, mandir, masjid and these sort of thing- that part of it is still there. But their daily way of life changed completely. Even if you go to a Muslim shop, like Tooting: Pooja- they are Muslim. But their behaviour, their discipline, neatness is much, much more than what it was before. I can see the improvement over 50 years. Remarkable improvement as such. Because of one generation is changed from the other. One chap has got a motor mechanic, or a repairing shop as such. One of the family members is still there because it's generation's money. Some of them are doctor, lawyer and that sort of thing, teacher. I think things have improved quite a lot.

Researcher: And the government has introduced a lot of measures, like the social distancing, working from home, et cetera. So are there any specific difficulty do you think the South Asian may have?

Participant (35:00) It depends on the type of work they do. Like a Bengali shopkeeper- he can't do it from home. But cases of professional or some of the things can be done from home. It would depend upon the type of job we were doing.

Researcher : So do you think the South Asian community in particular are in any different kind of job than the white?

Participant: Some of them like shop keeping and others, you know, restaurants. There are lots of small restaurant here in England, but they are much more, much, much more in number than South Indian and others like Gujarati. In general, if you go to a shop like Pooja, ok, lots of the things, the behaviour talking, and their other members of the family, are quite good as such, you know.

Researcher: Correct. And do you think that people in that community are able to access the healthcare facility during this crisis?

Participant: They do. Because the other people like three generation or two generation- an old lady of 70 or 80, now we are not all relying on God. We now know we have to help God to help us. So that sort of feeling is there. I went to a Guajarati community, a charity as such. I saw the lady there with trouser and shoe- Guajarati ladies. Because of the winter and cold things, they see it as hygienic. And they say, no, we have to do, we have to change it. The change came gradually, but it's not exactly what it was 50 years ago.

Researcher: And, and you think that they may have no difference to access the healthcare? Any difficulty? Do you see any difficulty or they are all same?

Participant: No, no. It is not that 10 people living under one roof- if 5 people catches, the other 5 will catch, it is not like that.

Researcher: Right. And do you think the South Asian community trust the government to make the right choice about the pandemic?

Participant: Well, there are people who are always sceptical. Because, first of all, party politics comes in. And that's why it cannot be a prudent judgment. So, for somebody like conservative, whatever they do is good. But for labour, whatever they do is bad.

Researcher: So what do you think about this current step taken by the Boris government?

Participant: I think he's excellent according to me.

Researcher: And you think the South Asian community may have a similar feeling?

Participant: Of course. Our finance minister, Rishi Shunak, Shunak is Shaunak, you know, meaning the teacher of the rishis (sages). But anyway, he is a Punjabi. He is quite sharp and bright. I listened to the budget he briefed..and he is also very, very sharp.

Researcher: Okay. So, to what extent do you think the South Asian people understand the health messages surrounding coronavirus?

Participant (40:00) They do now, even not hundred percent. There is a hundred percent improvement since earlier years. Only a little gap, I would say here and there. Some sort of community, they are cautious. Because you can see, an accountant or a lawyer, or a teacher, they can't be other- Indian and English mix could not be other. But in our home we can do the chapatis, we can do this; now the English are doing chapatis as well! They are not Asians or anything. They are enjoying the Asian foods as well, you know.

Researcher: And do you think these are people get the message what they are asked to do?

Participant: They are doing it according to me, over 80% of them are doing it. Because that thing, the type of people, 50 years ago, well whatever god do, will happen and that sort of thing people doesn't believe anymore. In my younger days, when we had to immunise ourselves, if we are unlucky that would happen to us, injection would not make any difference. But nowadays, they know, they think it makes a difference. In case of flu jab, everybody takes it, irrespective of if they are Asian, European or whatever it is, because the facility is there, they see it and take it. No one leave it to God or Allah, but they take it (jab).

Researcher: Do you think you can suggest anything how the message to the South Asian people from the health perspective?

Participant: According to me, follow what is advised, no advise is there to make any harm to you or something. And by not following you are not doing any good to anybody. Start with yourself, or any others- you are damaging others as well.

Researcher: So do you think anyway, these advise could be more reachable to the South Asian community and any way to suggest that?

Participant: I think it goes, as I say, 80% -85%, it goes to them as well. First of all, the community would have some sort of people, I say, from here, most of those of type of people are morally disappearing, so that would not happen. The thing is that people whatever South Asian people- they were not like that before. Though we still follow our religion, Hindu, Muslim or anything, but behaviour wise, we still follow the law of the land.

Researcher: And when you said that 80% of the community, so what do you think of the rest of 20? That the message is that reaching them?

Participant: There is a word TCC- they wait 'till coffin comes'. They will not retire from their view until they retire, until they die.

Researcher: So they wait until the doom day comes or the last day comes? Who would they be and why do you think they do that?

Participant: There is always some unreasonable people. It is not necessary they are illiterate- because there are not much of illiterate people here. But somebody, character as different. But again, that percentage is very, very low. And if you see the flu jab, almost hundred percent people takes it. So these are the examples indicating something- because the government gave them the facilities. The example indicating something. They took it because, because the government gave them the facilities. You can take it, here, there, anywhere, even at chemist shop, so they take it.

Researcher (45:00): Right. And this is the final question I'm asking you. What do you think has helped you and the South Asian community to deal with this crisis?

Participant: In my case, or in our case, we were well in advance even in India. So that's why, I am the third generation who came here in this country. So I had a link.

Researcher: Third generation? You are the first who came here right?

Participant: My grandfather, not my own, but my cousin grandfather, he was the first principal of the medical college, Calcutta medical college. He came here, stayed here. Then my uncle came here as well. So that's why communication with England was always there since I was born or before I was born.

Researcher: I see. Right. So do you see yourself as what generation would you say you are?

Participant: Well, the thing is that I am a funny man. When I go to India, or when I go to my hometown, I am a pure Bengali man. And I go here, there with a cycle, rickshaw (speaks in Bengali: I go to my friends' house and roam around.) But when I go to the urban area, where there is western culture like pubs, I adapt to that culture.) I can adapt myself in any aspect.

Researcher: Yes. So that, that has helped you towards the adaptability.

Participant: Yeah, because when I came here, my elder brother was here, was here, he didn't help me physically as such, but he was here. And I also know I am the 10th or 12th person who came here as such. So there was nothing new in our family.

Researcher: And you say your ability to adapt helped you?

Participant: Yes. Well, when I was twenty, I went to the plantation of the tea garden. And I stayed there for a couple of months with English people. They were managers and everything at that time.

Researcher: Do you think anything you can think about the South Asian community, which has helped them to go through the crisis?

Participant: I think following what is laid down as a rule. Follow it religiously. You keep them above the religion, and you can follow the religion after.

Researcher (55:00): Got it. And that has come to the end of the interview. Do you want to add anything?

Participant: No, I have covered everything!

Researcher: Thank you for your time.
