# Supplementary material for: Mitigating the identity and health threat of COVID-19: Perspectives of middle-class South Asians living in the UK
Source: J Health Psychol. 2021 Jun 22;27(9):2147–60. doi: 10.1177/13591053211027626 (PMC9353968; doi:10.1177/13591053211027626)
Supplement: sj-docx-14-hpq-10.1177_13591053211027626 – for Mitigating the identity and health threat of COVID-19: Perspectives of middle-class South Asians living in the UK [file sj-docx-14-hpq-10.1177_13591053211027626.docx]

**UK11_groupA_MAH_May20**

Transcribed by Sharmistha Chaudhuri

Duration: 56 minutes

Researcher: Thank you again for your participation in our study. It has been very helpful. I am going to start with the question, what do you think is happening to the world?

Participant: Few things are happening to the world. I think this is a change that we are not familiar with, so it's a huge change. I think, for most of us, the situation is very unusual and this is the largest unusual situation we are having worldwide since the second world war, I think.

Researcher: Correct! And what comes to your mind when you think of coronavirus?

Participant: In my mind, it's very strange, the first thing is positive and the other one is negative. So first thing, it's like a minor virus that can affect that much. We are kind of helpless. But we don't think about it all the time. Like for example, we didn’t think about all the technologies. We were having all the lifestyle, we are having these necessities of life, but we never thought of this situation, that a virus can affect us this much and we are helpless. Ignoring it is one thing. Another thing, you know, like, as the pandemic happens, we see some positive things. The positive thing is that now we have, we know actually know what should be our real costs? Like we come to our basics, so, and we come to know our actual needs. You understand? And we think as an unified way, not like that separately. I told you about the negative things. The positive thing is now I think, as a world, like everyone in the world, as the pandemic shapes, everyone, individuals have now some time to think about themselves. Like they can think about their basics. Now they know how it's important it is to be like, like your family, how important it is just to walk for example, and how important to just, how can I say, like, don't just rush and rush, just slow down. And to just come down to the bare minimum, you see. So that is a positive thing. I think they have the time to reflect how the life was before. That is the positive thing.

Researcher: Definitely. Definitely. And how do you think, coronavirus has affected the people in general?

Participant: (5:00) Um, that's my personal, of course my personal opinion. I think people in the beginning, they were very much panicked or we don't know anything about it. So we were, all, everyone was in a panic, they didn't know what to do. So they felt very panicked and helpless, but as the time passes by, um, then I think people just take it, um, like easily, I think. It's not absolutely normal, but they just take it easily. But in the beginning they were very panicked, people had no idea what was happening.

Researcher: So how would you think it has affected their livelihood or any other issues in the life?

Participant: So the first thing, um, essentially is the income, of course. The people..some people, I think how can I say it, like me, myself? I think I kind of actually enjoy in some way, because now I don't have to go out. So people who have job security, they enjoy this situation. Who doesn't have the job security, and that’s the majority, vast amount of people are feeling very insecure and very insecure about that.

Researcher: So, it’s good to know you are enjoying the time. So when you say enjoyment, can you elaborate what you mean?

Participant: Because we don't have that fast life. They don't have to think about going up early in the morning to catch the train or catch whatever people take or drive or whatever; go to office, or take the children to the school and come back in time and all the rush, rush...that is gone. So that part is gone. So our life is easier. We have the job security. On the other hand, there is a majority of number of people, I think more than half of the population they have this job insecurity. So they have no idea what’s going on. But just to keep in mind that I cannot, I can only speak for myself because for the last two months, I am actually at home and I don't follow up the news or I don't think about other things very much. My world is like, my world is like here in this house. So that's why I can just kind of, kind of say how people can take it.

Researcher: Absolutely, we want to hear your own voice, you know? And can you remember, how have you learned about the coronavirus?

Participant: I think we learned about it from the news and also from workplace both came at the same time. I think basically the first is from the news, newspapers and also TV news.

Researcher: And what are the media, which you follow for any update on coronavirus?

Participant: I follow BBC..the BBC mainly. So tv and the news channels.

Researcher: And what are the role of social media like Facebook, WhatsApp?

Participant: WhatsApp, there is, I think no activities in my WhatsApp about coronavirus. Once in a while I, as I said, I have a peep on Facebook, there's a lots of information and misinformation.

Researcher: Correct! Do you, do you follow them? Do you read them?

Participant: I used to, but now I stopped.

Researcher: So why do you think you have stopped?

Participant: Because I think it drives me crazy. That's why I stopped. Yeah, because in the beginning as I didn't know anything about coronavirus, so I followed up everything. What needs to done, on video or any media..social media. But then after a couple of weeks, like three weeks to four weeks, I just, I just stopped completely. No.

Researcher: Okay. And, could you tell us a bit what do you know about coronavirus?

Participant: Like scientifically or like which way, because I know both about it which I can add. So first of all scientifically I know that coronavirus, there are actually a lots of myths going on. That is not correct. Like how does is originated actually, no one knows how it originated. So its origin was from somewhere else where it started and then it just spread. And this is actually a new type of virus that can actually adapt with environments so it can evolve with the environment very, very fast and it affects human beings, homosapiens . And if it affects in very many ways the human body and it affects mainly the respiratory system, that is how people feel, like they cannot breathe, but it actually affects every part of the body when it is at the last stage. And now as the virus can evolve very fast with the environment, new symptoms are also coming up.

(10:00) That can for example, affect the blood, affect the circulations, so many things, so that is the scientific bit, but the genome if you track back because some people it's actually a hypothesis, like the virus in North America, for example, just track back. And if you track back the genome of European virus and also Asian virus, maybe it can be from different origin or maybe not, we don't know yet, but they are working on it. So we don't know scientifically, we don't know yet. We don't know what is the origin of the virus. People say from Wuhan, but or originally wherever it is from, we don't know. So that is, I know about Coronavirus, and, and it is a, how can I say, it transmits, so it's a contagious disease or virus, and it can transmit from any kind of human saliva, like for example, anything from respiratory system, for example, breathing from mouth or foam, any fluid that comes from the nose and it can actually travel. It's also airborne. So if somebody sneezes it can spread.

Researcher: I see. And when you say Wuhan, so you must have read about that somewhere. So how do you connect that information with your understanding?

Participant: Okay. Wuhan, that name comes from, because from the news we learn about coronavirus. Wuhan is the place where people have started to die from coronavirus. So that's why we knew that people are dying in Wuhan and there is a new virus called coronavirus COVID-19 and then it has started. But the thing is, the question is we learn about the death from coronavirus in Wuhan. Maybe the virus had spread already actually, or it transferred, like, and we didn't know about it. So that's why ..Wuhan comes because people, we have heard about people dying and it's because of a virus, and it is from Wuhan, that's why.

Researcher: So, so it may have already been somewhere?

Participant: Yeah, yeah. It has been already spread out and people, we didn't know because we have not tested, but a mass amount of people were in Wuhan and we learn it from the media, of course, and the news.

Researcher: Sure. And how do you think it has appeared in UK?

Participant: So of course from human transmission. That, that is the theory. Not theory, it is the hypothesis- it's not proven. But it must be by leaving homo sapiens, because otherwise the virus cannot transmit, that far we know, how the virus can transmit. So it must be from a human contact on human. So if a carrier was a human it must be from outside.

Researcher: So do you see any particular reason why you UK might be more affected by human carrier?

Participant: Okay. So it is not only in UK, but all over. But for example, Asia, if you go to, for example, India or Bangladesh or that part the death rate is not that much. So that'd be two reasons because there is no, we cannot keep track of the count because people are not tested. And also the death is not recorded the way the Western world does. So that is one part, that is like the technical part. That's why the death rate is not that much. But the other part is maybe because of because how can I say, is the genetics we have. It depends on the atmosphere, the climate and then genetics of us. So that can be different, but I'm not sure about it. It is all my guess. Like here, for example, if people are affected by coronavirus, different people are affected in different ways, even if they're like, for example, if we talk about a human race, for example, White British. If you think about White British, among them, also some people are effected more than the other, it's because of their whole, how can I say.. Um for example, somebody is prone to have diabetes because of the genetics. It's the same way, some people can be affected more by the virus because of their structure and genetics or lifestyle or whatever it is. So it’s the structure of each people. So, I think Asia, not like China, but like tropical country, they will take actually less. So one part is like, we don't keep track of the count. The system is not there. Other can be because our, genetics are different.

Researcher: (15:00) And since the beginning of the pandemic have your perception about coronavirus changed?

Participant: So from the beginning of the pandemic towards now?

Researcher: Yes, do you see any change or your understanding has remained the same?

Participant: I don't actually see any change. But I have become more reliable on scientific observations. For example, I can see that what predictions scientists made at the beginning of the pandemic, still now is almost 100% accurate. So I relied more on the scientific research and more on the scientist prediction. More on science.

Researcher: Did you believe in the same manner to the same extent in the beginning on the scientific methods?

Participant: Yeah, I, it was I was a little, yeah, because that will help prediction, right? Because of the times also they can only predict based on the data. And they said, I'm not talking about all the pseudoscience and whatever, like homeopathy..Its not like that- those are pseudoscience. But the science, the scientific research. So they said, like, it would be peak in April, May and it picked up in April-May. And they said like, it will decline like afterwards. And the scientists said, okay, these other precautions we have to make to make the slope. I mean, how can I say, to make the curve downwards. Like rates and we followed exactly their words. And even those who did not follow their, how can I say their words they were affected and those who follow their words, Germany, UK, Italy. Um,so, the results came accordingly. So, so I am, I'm more prone to like, actually, how can I say, there is another thing. So like now, we have to reflect on that, how much we should take scientific data or science. That is another thing that we have, like in this pandemic, we have another observation now. I won't hold it over world, people can be in denial about it, but they should really like some people really like, how can I say, understand that it's the scientific data, it is the cell scientists we should rely on in a crisis like this. Yes. So is there any changes? No, I think I've become more reliant on the scientific community now. And I don't think no perceptions...no, I don't think there is any perception change.

Researcher: Do you think this pandemic is any different from the other ones we had in past?

Participant: We did not have any in life time? Before our lifetime, when there was black death or like Spanish flu, we are not alive that time, not born that time. So, you know, in our life time, it is different because it's all over the world. Swine flu or mouth and foot disease that was actually located into like a specific area, continent. But coronavirus is not like that. Coronavirus is all over the world. So that's a difference.

Researcher: How would you think about the effect of the pandemic?

Participant: We're still going on until you see the effect! The financial effect, we'll see that after some time from now. Otherwise, otherwise, we don't know yet.

Researcher: (20:00) What do you think about your government's response to the pandemic?

Participant: I think the government could be like, I think they responded almost two weeks behind so they could respond two weeks before the date. But otherwise it was all right. But they, the government is kind of following some other guidelines, so they have taken little bit longer.

Researcher: And that you think that may have an effect on the spread?

Participant: I think so. Yes.

Researcher: And what was the information about coronavirus that has surprised you most?

Participant: Um, information about coronavirus that surprised me? I'm going to say I'm not much actually, because we know from the beginning how it...but I think now I'm very surprised to see, like it's not only the respiratory system it is affecting!

Researcher: So that that's something new. Right. And when you discuss about coronavirus with other people, like with your colleagues, friends, family, what do you mostly talk about?

Participant: I don't discuss..in the beginning of the pandemic, I was so panicked few weeks. I was like in a panic. So I was like spreading my panic everywhere. What is this? But now, I do not actually talk at all about it. All we do is just wash hands. If there we're not going out, take the precautions, that’s it. And I'm very strict about it. And I, if I talk to anyone, I just talk about that. It is just the safety precautions, safety precautions only now, nothing else. Like in the beginning it was like a maniac, you know, but now I'm just taking it easy. And taking all the necessary precautions. Like I'm taking and talking about it.

Researcher: How do you think that the previous discussion, what do you used to have and now you don't talk about that much?

Participant: The previous one and now ...the previous one was all negative, like panic, panic, but now it's more realistic and more positive. Okay, we can survive this and we can like, it's more realistic now. He began with the panic, panic, all the talking was about panic, like what would happen? Are we going to die. Are we going to die like that? You know?

Researcher: So has, do you think, has that panic gone now, like in you?

Participant: Yes. because now we have as I said, it went the way we predicted. So I can now kind of like rely on something we predicted because of the scientific community, everything like it went up and it will go down and it will exactly happen that, so now I know there was someone or some community, like scientific group or whatever, like they can just work together and they can just give us right information and we can follow it. And we'll be fine. I know now.

Researcher: You're trusting that the scientific community, which has helped you.

Participant: Yes. Scientific community. Yeah, mainly. And also for the government rules are there even, yeah, yeah, yeah. But about the scientific community, how this, how it has helped. I think it's the only thing that helped me.

Researcher: That's very interesting. Not, not, not many people say that way. How your personal life has been affected by the pandemic?

Participant: Personal life. I think as a family, we are more, how can I say, we give time more? I actually enjoy it, I love it! So my personal life, I have become more relaxed. I have become more like cosy at home and the family has become cosy of course! Yeah, of course. So it has become easier, I think, the life has become easier in that way. Yeah.

Researcher: That's the positive thing you've seen your family?

Participant: Yes. A negative thing is like, for example, we have to go for shopping or anything; we, because we don't go out, like once or twice, maybe for the shopping. So we have to plan from before. That is one thing. Other thing is like the children they cannot go to school. So they are like, our routine has been changed. Like our routine changed. Yeah.

Researcher: (25:00) Correct! And could you tell us a bit how, like your daily life now goes?

Participant: It's mine or my whole family?

Researcher: you can comment on your whole family, as a household.

Participant: Okay. So I, my waking routine has been changed. So the timing is different. Now, for example, I had to wake up early in the morning and go to work and come back and then go to sleep by maybe 11. Now it has become upside downs. So I sleep like four o'clock, three o'clock at night, I wake up like 11 o'clock. So me and my daughter, we are like this. For my son and my husband, I mean, their life is almost the same. They wake up early in the morning and they just work from home. And my son, he has ..he's very much disciplined. So he wakes up in the morning, he does his schoolwork and then he just stays with his computer, things like that. So, so each has been changed. We are less mobile now, that the negative. The routine has been changed.

Researcher: Why do you think your routine has been changed so drastically?

Participant: Because now in the morning I don't have to rush to actually get up. So, that is my personal habit. Yeah. No rush to get up.

Researcher: So you're taking it in a more relaxed manner. Correct. And how do you think this pandemic will end?

Participant: Actually, we do not know. It will end gradually, like following this, but we don't even know if it would come back again. Because people will forget about the social distancing. I, we do not know how, how the virus can react again. Spread more, or we shall, we have like immunity against it. We don't know. I don't know.

Researcher: That's a very tricky question. And the last question of this section is how do you think we might be able to prevent further pandemics like this?

Participant: That is a very tricky one because the virus or COVID-19 we knew nothing about it. So if there is some virus, germs. If that is unknown and what kind of unknown this will be, we don't know. So we cannot stop that. One thing I must say we have to rely on the scientific data. That is the only thing we can do, but we cannot make preparation that will be a virus like these. And then we can do these, to make vaccines. No, we cannot, because COVID-19 is an example. We knew nothing about it. So maybe there will be new type of virus. We know nothing about it. Only thing, we have to rely on scientific data. Not political, nothing political, like ..only the scientists. And the leaders of the country, the leaders of the world, the leaders of the community, everyone should rely on their data first and foremost, and they can make policy according to that. So the only way we can just make precaution about anything,

Researcher: I'm very interested to know your opinion, about the scientists. Do you, or can you trust the scientists, due to the political environment?

Participant: No. Sometime I don't.

Researcher: So how can you close that gap? Trusting them?

Participant: (30:00) Yeah, actually so far the positive thing is, for example USA is the prime example, like how stupid political leaders can be in talking about injecting disinfectants to prevent coronavirus- that was stupid. That was like, but the scientific community who are like the representative, for example the scientific advisor, he, what he was saying actually was from scientific evidence. Do you understand, the scientific community did not agree on to what Trump was saying? So they were not influenced. Some could misinterpret, what the scientific community said, but the scientific community denied and they were just saying the right thing. So that was the positive thing. So far, I think in all of the countries, also UK, the scientific community relied on their own scientific data. So they are not influenced by the political parties. The political leaders may change things, but there is a possibility of course, the scientific community are not influenced.

**Part II.**

Researcher: Yes. All right. So this is the end of section one. Now I would go to section two in which the focus would be on the South Asian community. Right. The first question would be, what do you think are some of the health concern for people in your community during this pandemic?

Participant: Okay. So first of all, I must say I am from Bangladesh, but I actually do not, how can I say, I don't have any contact with the Bangladeshi community as a whole here. So I don't know about how they are responding. We have some in-laws who are also originated from Bangladesh. I can talk about them, but they don't belong to the. They are from Bangladesh, like originally, but they don't belong to the community as a whole. Do you understand how, like, I don't know if I can explain it to you, like, we don't do things together with the community or do things I don't know about. I don't have any, any, connections.

Researcher: So, you're more integrated in that manner to the Western committee.

Participant: Yeah, yeah, yeah, to the whole community, actually. And actually, I don't have any contact with Bangladeshi community here, so I don't know, I don't know..I know nothing about them. But I know about my in-laws, for example, some of them they're from Bangladesh, but they don't represent the community. Like Bangladeshi community or South Asian community. I actually don't know.

Researcher: You can talk from your own experience, you know, and your own point of views.

Participant: Yeah. Yeah.

Researcher: You do not have to have any data to support your argument on because it is about understanding what you think. You see what I mean? So you do not have to say that I'm saying this because this happened there, but in general.

Participant: It will not, it will not be from my experience. I don't have any contact with them, but I don't know what they're doing or how they're doing, but I can only yeah..say from my point of view.

Researcher: Yes, if you have an idea of how it can affect the community as a whole.

Participant: Yeah. Okay. As far as I can.

Researcher: And do you think that South Asian community could be more or less at a risk to the COVID?

Participant: (35:00) I think they're more, more, more at risk. Why they are more at risk? First of all, because their lifestyle is like, they don't take..it is very hard for them to ignore the community. Like if somebody comes, because they don't just call on them, have an arrangement to come home, they just come home, just have a visit. And they like both of them, they don't take Covid seriously to prevent it. Generally, kind of, they are a little bit ignorant about it. I have heard about it, it can happen, but they're not very much .. Basically ignorant about it, more of a communal. Okay. That's the one thing. So it's ignorance, first of all. And other thing is like some of the in the community, they just don't, how can I say, whether that is true for here or not, I'm not sure, but I think they have less opportunity or access to have the best of a health system. I don't know actually. I'm not sure about them.

Researcher: It is perfectly okay to share your views.

Participant: Maybe I'm not sure. Maybe, maybe not. Maybe not. Because the Covid No, I don't know because the community is living in a certain area maybe, and the whole system there, I don’t know how good it is actually compared to the other part of the country.

Researcher: So why do you think that about healthcare access and in what manner?

Participant: Like for the GP..I don't know the services. I shouldn't say because I don't know actually. One thing can be a problem, is like, because of the, some of the people that cannot be integrated to the society because they just live in the community, you know, for example. Yeah. So when they go to the hospital or access to the doctors, they cannot communicate properly. They don't know how to, so it could affect them that way.

Researcher: So language issues and linguistic things.

Participant: Language issues. Yes. Things like that.

Researcher: That can be very valid.

Participant: Yeah, it could be. I actually guessed; I don't know. May be it is there- I don't know how it works.

Researcher: Though, we have discussed this, but just to reiterate, how has your family been affected by the coronavirus?

Participant: So here, my family?

Researcher: If you have extended family, in UK, you can also comment on them.

Participant: No, I can just talk about my family. Yes, definitely. I think we have become less mobile. So that is the thing like less mobile and less routine and more, more relaxed. More relaxed, less routine because you know. And physically less mobile being inside all the time. The children, they couldn't to go out at all. And because of this, they're not going to the school or anywhere, so less mobile.

Researcher: Got it. And when the government has introduced these measures, like distancing, social distancing, restrictions on travel, et cetera, do you think if the South Asian community is having or not having any specific difficulty to follow these instructions?

Participant: You are talking about the South Asians?

Researcher: South Asian community as a whole.

Participant: Yeah. I have actually no idea.

Researcher: Any specific difficulties you think they may have to follow these instructions or abide by that?

Participant: I have no idea, first of all, but I have no idea, but I think there can be also of course, totally dependent on my guess. Maybe, I don't know, actually, but maybe, as they cannot see the virus, you know, you cannot see there is a virus. So if you cannot see it, it doesn't exist. So there is a little perception on that. So they are, how can I say, so they've taken it less seriously maybe. Maybe I'm not sure. Actually, maybe I don't know. It's actually not for the South Asian community, but it is for whole. Actually there are always some ignorant people. If you cannot see it, it doesn't exist. For example, they can wear a gloves, but they don't know they are wearing gloves for prevention! You wear the gloves, but if you wear the gloves and you touch with the glove, something, touch your mouth touch something, the virus is transmitted there. They don't think in that way. They think, well, I'm a wearing a gloves and I'm fine, I'm protected. So that's kind of, but it's not only the South Asian, it's like, there are always some ignorant people, uneducated in some way.

Researcher: (40:00) So if you say ignorant, so who could be ignorant in that manner, of the COVID?

Participant: As a whole, I can say, not for the South Asian actually, as a whole, the ignorant people who don't have any, they are just that uneducated. They don't have proper education about viruses or diseases or anything on the hygiene, for example, as a whole.

Researcher: Correct. Yes. So now this government has put in so much restrictions. How do you think the South Asian community think they're doing it for their best?

Participant: I don't think, I don't think, I don't know about the South Asians but they're part of the whole community. So I think that they're not 100% happy about it. They cannot trust hundred percent. There, there are some things that they cannot trust the government hundred percent, that it will be all right.

Researcher: Why do you think so?

Participant: Because of the financial crisis, because those who are actually thinking about the government, those who are effected by the corona, like by their doles and incomes and things like that. And I think as a whole community, they don't think that they have enough from the government. They have been supported enough from the government. I don't think that they think in that way. I don't know if that is possible. I have no idea about it also. I don't know if it is possible for the government to support everyone the way everyone wants, but so far the government has done. I think not anyone like everyone is not happy about it.

Researcher: And you mentioned some issues with the community in accessing the healthcare facilities. What particular challenges can you think that they are really facing that way? You mentioned about language; what else can you think of?

Participant: Another issue- I actually kind of forgot about that, on other point about the coroner virus, I think in the South Asian, Bangladeshi community or Pakistani community, uh if there are, for example, one person in the household, you speak of coronavirus, they don't want it to spread. They don't want them to be known by the others. So I think that is a taboo or how can I say no, not taboo, but I think this is a major issue.

Researcher: So why do you think someone would try to hide?

Participant: Because they think like people will see them differently or if they died out of it, the people will not come for their funerals, or people will see them differently. So because the community always think about others, right? How the people will think of how they'll see us. That is one thing. They will think like, okay, we are affected. So it doesn't matter if somebody else- so they don't want to share the information with them. So they take it more personally rather than. So that can be one issue. And that's why to go to the doctors may be, I think there can be a little bit denial about it. I'm not sure actually, I'm making everything in my mind now.

Researcher: Please do, you are allowed!

Participant: Yeah. So they can be maybe..denial about it. Maybe if they have a symptom, they don't say like, okay, this is a coronavirus symptom I'm having. And maybe there can be denial about it because they want to hide it actually. So that can be one, one mental block. Other thing is like maybe language issues can be one. They don't take like the ignorance can be, they can have like little information about it. And thing that is normal, like normal, how can I say the cold and flu or because of the misinformation they're having from like information, they can just have tea or like gurgle and then think, oh, we are all cured. They don't have the right information. So that's why they don't go to the doctor. They don't take it seriously as it should be.

Researcher: And what do you think about the messages about the health reaching the community? How do you think anything could be done to improve them?

Researcher: (45:00) In the South Asian?

Researcher: Among the South Asian people?

Participant: I think the messages will be for everyone. It should be like, you have to, I'm actually very much, you have to take the right information from the doctors, not from others. And also like for example, in a temple or in, in, in a church or what is that called? The mosques or the priest and all of them. They all have to be trained to tell people you have to go to the doctors. And one doctor says, because they represent the scientific community, you have to listen to them, don't listen to anything else. Don't just drink tea or like ginger tea, just to cure or like that. Listen and rely on the doctors?

Researcher: How this message can be better delivered you think?

Participant: I think it's going to be delivered...and as I said, it can be delivered because they take very much importance of the community leaders and who are the community leaders? They are the imams at mosques, or because they're rely on them. So those leaders, community leaders, if they convey to the masses, I think they will take it seriously. That is very important. And then of course the children go to the school. This is information, but it will be valid if the parents also say, okay, yeah, the imam said, you know, okay, the priest said, the purohit said- this is the thing, we have to do. So I think the community leaders taking responsibility. Yeah.

Researcher: What do you think has helped yourself and your community, South Asian community to deal with this crisis? Can you think of any specific issues that has helped you to go through this crisis?

Participant: Yeah, so I spoke already about that, like, what I rely on. What is predicted and not. Yeah. That is one other thing, actually. Yeah, just talking normally with the friends, like I have my friends in Facebook, that is a real friend. I know them and my family members and I just see okay on what you're doing now today. Okay. We have done this and that like, so daily life, that we share. Yeah. And that help us actually. So just to having conversation with others.

Researcher: So, normal casual conversation.

Participant: Yeah. Casual conversation. In the beginning, it was panic conversation, of course. But now it's a casual conversation. Yeah, that's helped.

Researcher: And, like as we know that now it's Ramadan month. So how do you think for the Muslim Bangladeshi community it has any effect? Like during the festival month and for that Eid coming up, how do you see this and how do you think they are coping with this crisis?

Participant: Okay. They are coping. They find it very abnormal because Ramadan should not be like this. They should be different, different traditions of it. You know, the stars and all the things. And Eid would be different, that they find it very unusual, very unusual Ramadan. So they are taking it very unusually. This is a different type of occasion.

Researcher: Do you see any restrictions which has been put on the people due to the COVID to celebrate?

Participant: (50:00) Yes, I think so. I think they, yeah. Yeah, of course. I don't know if it is absolutely like correct what they are maintaining, but there has been huge change than usual. It has affected them a lot. Yeah. For example, like in the Easter time, there should be a whole community eating together because that is the tradition, that is part of this, but now it's gone. During Eid, it's like lots shopping or something like visiting each other. Like, you know, this is the time for jamaat, the prayers. It will not be there. So, there will be a huge difference.

Researcher: How do you think they can cope with that crisis?

Participant: They all just have to take it; it is an unusual situation. They are not happy about it of course.

Researcher: On the other hand, do you see, the celebration can affect the covid?

Participant: yeah, it can actually. For example, let me put it in this way. There is a very unclear declaration from the government, ok, people can meet each other up to 7 people. That is a very unusual announcement from the government. These can be.. this is actually mis-guidance, it can happen at any other community. Government has allowed it, yes, by law they can do it, so it entirely up to the government. If there is no clear instruction, it can happen.

Researcher: We have actually come to the end of the interview. Do you have anything to add?

Participant: I don’t know if I was to the point to answer.

Researcher: They are perfect!

Participant: First of all, I can say, we are living in a bubble now, since the coronavirus started. So inside the bubble what I can say, some part of it is just prediction, it is in my mind, but I just shared with you.

Researcher: Thank you very much.
