# Supplementary material for: Mitigating the identity and health threat of COVID-19: Perspectives of middle-class South Asians living in the UK
Source: J Health Psychol. 2021 Jun 22;27(9):2147–60. doi: 10.1177/13591053211027626 (PMC9353968; doi:10.1177/13591053211027626)
Supplement: sj-docx-15-hpq-10.1177_13591053211027626 – for Mitigating the identity and health threat of COVID-19: Perspectives of middle-class South Asians living in the UK [file sj-docx-15-hpq-10.1177_13591053211027626.docx]

**uk12-groupb-cdu-may20**

Transcribed by: Sharmistha Chaudhuri.

57 minutes

Researcher: Thank you again for participating in our study. And, as you know, that today's study, it is looking at your perception of COVID-19 as it is happening now at the moment. So my first question, I will begin with, what do you think is happening to the world?

Participant: All over the world people are very stressed, anxious, worried, scared, including me, my whole family. Because it is so infectious and serious and there is no cure.

Researcher: The anxiety, and the fear! And why are you scared?

Participant: Because, I lost, my husband, two years now. And I used to go out almost every day. My grandchildren used visit regularly. Now I am told no one should come into my house. I should not go anywhere. So that's making me more anxious, that whether I will see them again, or not, or when! People don't know the deadline, that is the problem.

Researcher: What comes to your mind when you think of coronavirus?

Participant: You see, in the beginning, nothing but I was watching nonstop TV or radio. But in the end, obviously people are phoning you from all over the world. Relatives, friends. I think I'm little calmer now. That everybody I know are still there. I did lose a friend, but she was in old age home. We never used to meet that much, but it shocked me a while. But I feel I am lot more calmer now. Another thing, the friends and neighbours thing, that friends and neighbours, I never knew some of them- they all knock my door, that I live alone. All very very kind and helpful.

Researcher: Do you see any picture, if you think of coronavirus, what picture emerges in front of your eyes?

Participant: Originally, what that they were showing us that people are not allowed to see when they're dying; they are putting their bodies in the bag. That really made me upset, that no one can see you, even when you are... Now I don't watch it that much, I only watch once or twice a day. I pray that everybody even whom I know or not ... There are lot good things happening. Like people are raising money- that old man, I follow him, it is his birthday today, 100 years old. Both good and bad happening.

Researcher: And how do you think coronavirus has affected the people in general?

Participant: More or less I think most of the people feel the way I feel, because they all are saying either in my age group or younger, that God knows when it will end, whether we will see each other to again, maintain the function or not, so same, same repetition really.

Researcher: It's mainly because you're not being able to meet people. Right?

Participant: Yeah. As if your liberty has gone completely.

Researcher: Absolutely! And how have you learned about coronavirus?

Participant: Mainly from tv I think. And WhatsApp came from all over the world. Some people saying that it is purposely done by China, some said no, no. So mainly from TV.

Researcher: Mainly from tv. Is there any news channel you watch?

Participant: Mostly I watch in the morning. I make sure that I watch about an hour, BBC 1. In the beginning, originally I used to watch, god knows, nonstop. But now after three months I don't bother that much.

Researcher: So why do you think you have stopped watching the news so much?

Participant: Because some time I think I am listening same thing. And there's nothing, nothing I can do. I do listen, whenever I go into the kitchen, and not in front of tv, and make the dinner ready, I switch on the radio. But there is also no other talk than this talk. But there are a lot of positive talk: people are coming back, our prime minister came back. Good and bad. So, as I say, most of the time I get fed up of listening. So I don't listen that much.

Researcher: (5:00)I Think that's a better way to deal with it really. And, do you, also look at the WhatsApp news, the messages via WhatsApp?

Participant: Yeah, many people are sending. Yeah whenever it comes I read it. And out of that now, lot of nice things coming. People are making music, watch party. I, only, you know, have a smartphone only 6 months. I was not interested, you know. But because now I am sitting at home, started using it more.

Researcher: So you can make use of that now! Now could you tell us a bit about what do you know about coronavirus?

Participant: Medically, all the days they're comparing, it's like flu, like, is it another one- was it SARS? Mainly people are worried because if you catch it and if you have some underlying health issues, then the death is also very painful. And originally, they used to say there are not enough ventilators. So everything what comes in TV, I only know. That you are aged, so you have to be careful. And if that can be spread through cough to the next person, so better have to cover your nose and mouth. Whatever they are saying, same thing, you know. What are the symptoms, every day they are saying new new symptoms. But I never worry for our age group because I'll be 75 soon. All I worry about is my grandkids.

Researcher: Okay. So in your opinion, how coronavirus appear? What do you think?

Participant: From some people travelling I think. Originally, whatever they are saying, if it is true, it's from China. How is it I don't know. And because they didn't take any action in the beginning, a lot of people came from all over the world.

Researcher: How do you think it happened in China?

Researcher: Oh, that I don't know. They're saying it's from the bat from the market, like what I watch in the TV or something made up in the laboratory, because they want to become super power. Who knows what is true?

Researcher: So which one do you think could be the possible one ?

Participant: That could be, I think you know, when I'm reading all these nowadays all the posts, that it could be true, that they're trying to make it. I'm not sure about anything really.

Researcher: And if you think that it could be true that they produced in a lab, why do you think they would do that?

Participant: Like they are saying, in the post, they want to build the superpower, they want to dominate. You see, I'm not so politically minded person. I only know by what I'm reading and then I accept few things and something I can know. It may be just rumour, so, I don't know. They never spoke originally anything much about that it is manmade in tv at least, but now I have heard quite a few times in LBC that they were discussing that it was man made. So I have no idea whether it is true or not.

Researcher: Right. And how do you think that this virus appeared in UK?

Participant: (10:00) From the people who were travelling, and they didn't know because their symptoms are like flu. So the people, might be even now, the problem is, half of them, they don’t know..there were not enough testing. So whatever discussion goes on, I think part of it is true! People don't know, then what can they do?

Researcher: And since the beginning of the pandemic, have your understanding of coronavirus changed?

Participant: I understand one has to be careful, one has to cover the nose. Main problem is that people sometimes don't know after seven days. Somebody, like you say, I am meeting someone if they allow and if that person has it or I have it, I don't know. I don't know. Really. I still think we have to be careful for a long, long time!

Researcher: You mentioned that initially you used to watch a lot of news, but now you don't?

Participant: I find it, same thing more or less yeah. Ok, originally they never said anything about smell and taste, they may have said, but did not get. Now staying more in the home. And then when they're giving the number of how many died I was feeling is just like, as if some mosquito or flies has been killed. Everything goes numb after a while. I think, if a plane full of people goes, they will talk more, you know, the way they're saying, Oh, today's 400, tomorrow was 300. So I don't know..the value of life. The country I come from, India, it's impossible to manage the workers back from work. I feel sad, and that is why I stopped watching it. That I cannot solve anything, rather it makes me more depressed. Again, I am saying- because I am on my own know, anything because that makes me more depressed because I'm on my own note.

Speaker 3: It is more difficult, isn't it? Yes. And do you think this pandemic is different to any other, which happened in the past?

Participant: Problem is, I never faced any in my life. I left India 54 years ago and, unfortunate or fortunate, I heard few things from mum and dad, but I never faced anything like, you know.

Researcher: So, while looking at the pandemic, which happened, maybe not in your lifetime, but previously, do you think this is any different or this may be the similar to what happened in the past?

Participant: I recently read about Ratan Tata. And then I can't remember, but I remember, he mentioned a lot happened in the world lately: world war, plague and all the other you know. And in the end we will all get back to normal. And a lot of people have obviously lost their dear ones, and there will be a lot more. And that I also hundred percent believe that all will die, But I only pray all the time to God that, you know, like our children, let them survive. We have our time. Please, please let our children- and they are suffering mentally a lot. And I was doing volunteering in a school, that way I miss it very much. I was told not to go long before it was closed.

Researcher: And what would you think about the government's response to the pandemic?

Participant: (15:00) You know, sometimes I think that nobody handled in all over the world, a situation like that, I'm not the one to criticise, I'm thinking that they are trying their best. They just don't know. They may be all in the government, but they are not super human being. I think they're trying their best.

Speaker 3: And you would agree with how they are handling the crisis with all the measures they are putting or anything they could do better. What do you think?

Participant: No, I will not criticise at all. Whenever I see the hospital situation and all of that, the program only on tv, I don't read newspaper that much, listen to LBC, and I think they're trying their best.

Researcher: And what was the information about coronavirus that most surprised you?

Participant: Meaning?

Researcher: Something new..which you didn't think that it would be, something that you saw and said wow..

Participant: I never thought anything more that they are telling you. I don’t know much!

Researcher: Is that something like you thought that, I never thought that way. So this is something new?

Participant: I, I never really thought that way because every time what they're giving you in the plate, yeah. Sometimes it surprises me when 90 plus people are coming back and the young ones gone, and they're saying the children never catch it. And then they're showing next minute, yes! Few kids died, young ones, all of these make me a little bit surprising or confusing.

Researcher: Correct! That that's, that's a good point actually, because elderly people coming back and then young children dying, which was not in the news in the beginning. Right.

Participant: Even in my friend circle, Dr. R, I don't know if you know him, they both were admitted in Covid virus, and even know he committed and he was almost in very bad condition! And he came back! He and his wife, both.

Researcher: That's a very good point...the elderly people, they are coming back and young people are dying. Yes. Yeah.

Participant: Even they were showing how to hold babies yesterday, I was saying it before also, that the baby was born premature, and he is back! Another thing that really bothered me, some of my..his name is D, and he used to work in Victoria, with the black people who died. One was spot dead. Did you see the news, that the lady, somebody spat on her?

Researcher: Yes. I saw that.

Participant: And she, she used to work with D, who is like my adopted son..I have them all over the world, lots of sons and daughters! And he texted me, 'oh, auntie, I lost a close friend!' . So, three of them died basically. And they are all black...I keep wondering, why is that!

Researcher: And when you discuss coronavirus with other people, like your friends, family, what do you discuss?

Participant: Clearly, when we going to meet each one of us? Are we going to see each other again? There is a Loknath Baba puja...in mid-July- A friend does puja, you know. So, when are we seeing each other?

Researcher: So, yeah. I'm sure you will do that soon! And, how your own personal life has been affected by this, I mean, though you have mentioned about some already?

Participant: (20:00) My personal life is terribly affected in the sense that, God help me, I used to go out every day, whether it is sunshine or rain, at least for 2 hours. I used to go around using my bus pass, all completely stopped. Because my son and daughter, they are very very strict about it, so now I can’t go around, I can’t go to their house. They only come once a week with the kid and from distance for 10 minutes. So it has affected that way. I am a very friendly person, so I used to go to friend's house, with my little bit of cooking, because they are elder than me. So all this stopped.

Researcher: So that's, that's terrible.

Participant: I have not been doing anything; just by myself!

Researcher: And can you see anything positive happening to your life from this pandemic?

Participant: Yeah. Yeah. I can do lots of chat, I have lots of WhatsApp group. Neighbours, good, good neighbours, I hardly knew their face, maybe some of them I've never seen, they made a group, they included my name and some of them really knocking almost every day. When I say, Oh, I'll go out. Or my daughter in law said, no, no, no, if you need anything, we will get them. So yeah, good things happening. And then from India, because people are sitting more, they're sending lots of music, lots of nice talks!

Researcher: So this is something which has not happened really before the pandemic, right?

Participant: No, no, no, no.

Researcher: So why do you think things has changed in your surroundings?

Participant: I think out of, out of something bad, always something good comes. As I explained first, people has more time in hand.

Researcher: So this has got something positive. How about your son? What is happening with him currently?

Participant: My son he's one of them. He doesn't talk much about anything at all. It has affected him because he is self-employed. Small business. They wanted to give more time to the kids, that is why they decided to go self-employed. But then again, he knows my mom is a warrior and no matter how much I asked him, money wise are you ok, he says you don't have to worry about it.

Researcher: Yes, definitely. And can you tell me a bit how your daily life goes during the pandemic?

Participant: My daily life? You see, I sleep very late always. Almost morning, 1 o'clock, 1:30. That is why it takes time to sleep. I get up in between, I have to go to toilet and this. And I, I keep watching tv, BBC news, as I said, from 8 0'clock. And then after an hour I make my cup of tea and toast. And then again, as I told you, this is the best time, I just sit down, answer phone calls. I can't be bothered to cook and clean and all that cooking. Cooking is really really unimportant because one person, whatever I cook there is leftover. I eat, obviously, but try not to waste. I used to take it as I said, to grandchildren, to others, but, now I can't do. It is sunny now, so I go out my house, just in front of my own house, for a walk. Just local walk for half an hour, and then I come back. I do go out from Tooting to Broadway, but not every day. As I said, my son says, oh mummy, you don't go out, don't go near other people. Just walk on your footpath.

Researcher: (25:00) How do you think this pandemic will end?

Participant: That is one thing I have no idea. I wish it ends. I think it will take a long time. It's all about the infection, one have to be careful. And one day we will know they will give us vaccine as they said, but that is also long time. They have to give it to volunteers first, isn't it?

Researcher: Do you have any idea by how long...what do you mean by long time?

Participant: A Year, at least for the time being. I don't think people will be able to go for any holiday, everybody plan to go to India in November, December. I don't think it will happen. Maybe scared, but you know.

Researcher: And how do you think we might be able to prevent further pandemics like this In future?

Participant: Like corona or any other thing?

Researcher: Anytime, anything like that in future..

Participant: I don't know because they've got time to get ready with this one, isn't it?. If anything same sort of thing comes, I think they will be better equipped to help patients, whoever suffers.

Researcher: So do you think that the world has learned any lesson from the pandemic?

Participant: I think so. I used to think that why do they have to fly so many planes? I used to ask so many 'why' questions in my mind. That when we came originally, there were not so many planes. Why every day, they have to go to India. Why can't it be alternate days? Why there can't be less flights. Look at the sky so clear! People sending pictures. Somebody from Punjab texted that they can see the Himalayas, Everest, which they could never see before. So sometimes I think more than the necessary people are doing. One household has so many cars and including our family, so why we need so much. We don't need so much.

Researcher: So this is then a new thing which has come to you, right? Like we can survive without so many things.

Participant: Not very new thing. Because our generation, when we used to sit down, we used to discuss all these all the time. That we came, we never had this, we never had micro-oven, washing machine and we survived. Now you cannot go without them for one day. We all were working, we all were healthy, you are more careful, look at us, but this can't be a problem because we all are living longer.

Researcher: So you think dependence on the technology is not good?

Participant: Not hundred percent all the time. If you didn't have them for one day, then you would say Oh, God..I don't like the feeling.

Part II.

Researcher: Right. Okay. So we have ended the first section. I will proceed with the second section in which I will ask you your understanding about the effect of coronavirus on South Asian people.

Participant: Can you explain what you mean by South Asian?

Researcher: South Asian means Indian, Bangladeshi, Sri Lankan and Pakistani. Mostly these, these people that we are talking about. Okay? So what, what do you think are some of the health concern for people in your community? Like the South Asian community during this pandemic?

Participant: You see, in my road, I have a mixed population and my next door neighbour is a young lady..not very young- but in 50 plus. She lives on her own, I have never seen her wearing mask. She goes out by her bike, at least once, and she walks at least three times to the shops. She asks whoever she thinks needs her help. She doesn't, you know, tells me she doesn't tell me anything about these, that I am worried, or I won't go on and all these. And then next to her are people with kids.

Researcher: (30:00) Tell me again, is this lady South Asian?

Participant: No, White. But my next to next door is Sri Lankan and the husband is Scottish. She hardly goes out, I have seen her. She stands on the clapping day at distance, and calls me auntie. She is born here. But she is very very careful. Even when her son's birthday fell in between- I asked her, are you comfortable if I give him any present- he is four years- and she said, auntie, leave it in your house. So I realised that she's really got scared. In our road there is another Bangladeshi lady, elder than me, and she also lost her husband a year ago; she and I used to go together, shopping. Then I don't have a nice garden, She'd got a nice one. So she used to call me almost every day, that come and spend time, have a cup of tea. She stopped doing that, she is also a bit scared. And mostly, all the other I know, they're mostly white people. Where they are from I don't I know. Local, Pakistani or I don't know. Whatever I see in the news I know, that a lot of Pakistanis were effected and when I saw the doctors. But personally, I don't know much.

Researcher: So do you, do you see any health concern that this community has in particular?

Participant: No, because they only talk to me in, in WhatsApp group if I need anything. Like a Window cleaner, you know somebody, they come from outside and do it..nothing much, they don't talk.

Researcher: Would you think that the South Asian community are affected less or more by pandemic than the White people?

Participant: l the news item I can gather is that a lot of Pakistani, Bangladeshi were affected, there, as I said, the Bangladeshi lady who lives on our road, her son texts me, sometime, when any Bangladeshi died. So, it may be twice than the White people. He is born here and it must be affecting him. He is 50 plus. So every time I've seen, or anybody Bangladeshi dying, he always texts me, that auntie, I read that. So he is worried why it is affecting the Bangladeshi community so much.

Researcher: So why do you think it is affecting the Bangladeshi community in any different form?

Participant: I don't know really. Because they have more children, sometimes they're stuck up in their house, but then the one he is sending me, are quite well off one. There quite dig it. It's not, they're living in poverty or anything. No idea, it must be our immune system.

Researcher: Immune system. And if you say poverty and all, how do you think that can contribute to the coronavirus?

Participant: Because of your food intake and all these, may be different, may be lot less healthy. But then again, this is so confusing. Lot of perfectly healthy people are also dying. And I sometimes think that people in India, compare to the number of people and number of death, in my mind, it comes that because poor country people have lot more immunity, sunshine. They can take lot more than us, all these normal things comes to me for a normal person like me, I don't know entirely.

Researcher: How would you think the South Asian community has been specifically affected by this crisis?

Participant: Okay. I don’t know. When I go out time to time, up to Tooting, then my Pakistani shops are full with people. The Ramadan is going on, I think they don't care. They are going inside- next to that there is a Tesco, they can see that there is a circle to stand, for social distance, blah, blah. But then when you pass those shops, even I'm going to be scared to go in, because they don't care- small shops, with lot of people inside. I don't know whether they are worried or not. They think whatever god does will happen. I also think so, time to time, looking at them- that they let that be with Allah!

Researcher: Right! So are only the Pakistani shops or any other Asian shops?

Participant: Asian shops mostly. Near my home there is a Turkish shop, that is 24hrs open. That is it. All I see people are not bothering. Even I complained in the beginning, I'm not going in your shop, and then someone will complain about your shop. After that I noticed, people are waiting outside, but still, they are allowing..you cannot maintain the distancing like that. So either they don't care, or don't believe all these, I don't know. The girl in the till never wear a mask. Originally I went once, and after that I stopped going, when I see how many people inside.

Researcher: And how has your family been affected? I know you mentioned some, but anything more you can add?

Participant: My close family, my sister in law, I have not seen her face. And she has got 5 grandchildren between two daughters. All of their birthday gone and I had to just wish over phone. I haven't seen her for the last two months, not even stopping dropped or anything. And as I said, my son tries and make it every week, and my daughter in law. I mourn that, I haven't seen my grandkids, I miss that hug because my little granddaughter was 13 on March. First time I am here and still wish her or meet her. And my daughter in law's dad is 85-86, poor man, also they go outside and say hello to him. And his birthday was in between, in April. So yeah, it's all mentally, physically, maybe we are going on.

Researcher: And if you look at the government measures, which has been introduced, like social distancing work from home, et cetera, how do you think the community are coping with this measures?

Participant: When I see my neighbourhood, most of them are working from home and they are in really good good jobs. They are solicitor, media. I don't know much, about seven, eight family I know. Then they are coping very well. Wherever we are outside, we talk, Thursday, mainly, the clapping day. They're all young, 30+. Like they never moan. Some of them has no job, of course, worrying. Some of them I know they are in the media. So they don't have any, earning. My GP also born here, but Bengali, but he's younger than my son. So only 40 plus. He also, his wife comes outside my house and chats with me, she calls me auntie, so everybody, you know, feeling mentally depressed.

Researcher: Do you think there are any specific difficulty in the South Asian community to follow the measures?

Participant: (40:00) Something, only thing that comes to my mind that you know, they live with lots of family members, sometimes. And suppose someone is in the key job..then they have to go isn't it. So they must be some precaution when they come back. Like my GP, a young man, he has to go back and he he's got a 5 years old girl, will be 5 in June. So when I check with his wife, that what is he doing? She said, oh, auntie, you should see when he comes back. Washes his hands.. And blah, blah, blah, many times. But now he is also fed up.

Researcher: It's so difficult. It's so difficult.

Participant: And his parents are both elderly. So it didn't stop him going. I asked him, he said, no auntie, I have to go see them, whether I am a doctor, seeing patients or not. I can't leave them alone.

Researcher: To what extent do you feel the community is being able to access the healthcare facilities?

Participant: Healthcare..I know one or two cases amongst my friends. I had a serious boil, and the pus had to be taken out. She was very, very happy. She went 2 weeks ago, to St. Georges. She said, I could not believe there are so few people. You can count in your finger. And I was treated, the pus was taken out. Another one said that she had a small stroke, very minor stroke. She stayed one night. Another one, had to go, with her irregular heartbeat, which she suffers from. And touchwood, they all came back home.

Researcher: So when you look at the South Asian people in general, everybody, do you see any particular difficulty they may have to access the NHS, the healthcare?

Participant: I don't know, some of them told me that, she lives very far, in Reading. She was mourning that, their doctor completely refusing to see, because she is diabetic and all the other problem. So the only 111 and then she had to wait outside the surgery. But most of them were saying that they were happy.

Researcher: Do you think the South Asian community, they trust that government, that they are making the right choice for them?

Participant: I, don't know really. You know, I never discussed all these, anybody except our personal, when we will see again and how are you. I personally, as I told you, it is such a new thing, nobody in the whole world knows what's going on, but that the way I think they're trying their best.

Researcher: To what extent do you think the South Asian people, they understand the health messages given out?

Participant: I think most of the people do understand the severity. I don't know. Do you know, one friend, she still cannot grasp the whole thing, because she lives with her daughter. Her daughter is a key worker. Her husband is a bus driver. The grandkid goes out to the school for key worker. So she, up to last week, no matter how much I'm telling her that there is no necessity, she's old, 80 almost, to go out, but she goes out.. She changes two buses. One day she appeared in front of my house. I couldn't say anything. I felt bad. I had to make her seat. And then another day when she came, I was walking on the footpath. So I just said, please, please, don't come in. Even my grandkids don't come, my son doesn't come, he would be very annoyed if anything happen. So, And that is the only one I have seen amongst hundreds of people I know. She goes out every day, she goes to the bank. She goes to shopping, even though she doesn't need all this.

Researcher: (50:00) So why do you think, what is the difference?

Participant: I think because she's seeing her household every day, going out.

Researcher: I see, I see.

Participant: That she thinks if everybody in the house can go out, she can can go out. Why I will be bored? I am somebody who is fanatic maybe! So, whether there are any families like that? That I don't know.

Researcher: That's a very valid point. That's a very valid point. And you remember, you mentioned about the Pakistani shops and the shops which are not maintaining the social distance. So how you think the message can reach them or what the government or anybody can do?

Participant: Some people cannot do anything. It is like a family, small circle. You cannot send some people, I don't think it will go. They just think, as I said, leave it to god.

Researcher: But even then, if the government wants to try, what are, how do you think they can reach them?

Participant: I don't know if they're allowed to go to mosque or not. So they can go to the preacher- to tell them. It's like that. I know the temples are shut, but as I said, mostly, I have seen Muslim community don't really care. Because they are doing iftar shopping. Like yesterday, I went out at seven O'clock thinking that I go for a walk and the streets will be empty. As soon as I went near that vegetable shop and Pakistani mangoes and all these, it was crowded as if it was 12 o'clock.

Researcher: So is it particularly relevant to the Muslim community or do you see that in other places?

Participant: Because, the Guajarati people I have seen at least, some Guajarati shop shop we have, they are telling the people to stay one at a time. Then I have seen another shop also, the sweet shop, don't come more than three. They are all small small shops. But since it started, I have not gone to Tooting Broadway, so I don't know. I have only gone halfway.

Researcher: Finally, what would you think has helped you and the South Asian community as a whole to cope with the crisis?

Participant: Phone! My life is depending on the phone. They all know, that I don't get up sometimes until 10, but the phones are coming lot earlier.

Researcher: That's very good that has helped you a lot to do carry on with the crisis, right? And if you look to other people around you, South Asians, do you see anything which has helped them to go through the crisis?

Participant: As I say, do you know my near neighbourhood? I only know, like that one Bangladeshi family. But they are all asking each other. Even when I'm going out, or when my son comes or drives. I also ask them, you know, my son is coming, do you need anything heavy? Rice or flour and all these. So I think people are really really helpful. I'm lucky, my both side neighbour, nearly everyday checks on me. Boat. Since my husband no more, maybe that's why. I don't know.

Researcher: So it's the community feeling?

Participant: Yeah.

Participant: Okay. So we are at the end of the interview now. So do you remember to say anything or do you want to share anything we have missed out?

Participant: Not really. I only wish like everybody else that it gets over. And again and again, I pray to God every day that keep all of us. The second generation at least.

Researcher: Thank you very much for your participation.
