# Supplementary material for: Mitigating the identity and health threat of COVID-19: Perspectives of middle-class South Asians living in the UK
Source: J Health Psychol. 2021 Jun 22;27(9):2147–60. doi: 10.1177/13591053211027626 (PMC9353968; doi:10.1177/13591053211027626)
Supplement: sj-docx-16-hpq-10.1177_13591053211027626 – for Mitigating the identity and health threat of COVID-19: Perspectives of middle-class South Asians living in the UK [file sj-docx-16-hpq-10.1177_13591053211027626.docx]

**uk-13-groupb-DSI-may20**

Transcribed by Sharmistha Chaudhuri

(60:14 minutes)

**Part 1**

Researcher: Thank you for your participation again. The first question would be what do you think is happening to the world?

Participant: As per the general information all around, it is a pandemic, and which is a typical virus, which is a global pandemic virus, that is the pandemic and it is turning our normal life upside down because of the effect of the pandemic. This is the general global perception, which I also share the same views.

Researcher: So when you say upside down, what do you mean by upside down?

Participant : Upside down means whatever has been the norm in all spheres social life, school, college, education, commerce, trade, travel, um, everything and anything that you can think of, has, has come to a certain halt. Just like a fully running engine. Certainly without any reason has just stopped turning the whole thing upside down.

Researcher: Correct. And what comes to your mind when you think of coronavirus?

Participant: Well, it is an unseen enemy, we don't have much information about this enemy, if you may call them so. And you have been attacked without, um, any notice, certainly. And you don't know how to tackle the situation we are facing, this what my personal perception in general. That you are fighting with an unseen, unknown enemy and you don't know how to battle it.

Researcher: And why do you think specifically, that it is, we do not know how to fight with it?

Participant ([02:33](https://www.temi.com/editor/t/H-TwOEKHuPxFPzNVwXj6i6VSr_mAQKhWTzUQyQBik4BVgltxTLvQcLlcQKKpHNItXufWKMCbB-RVy0SmiNW8WcAH65M?loadFrom=SharedLink&ts=153.77)): Because it is a new thing, and you don't know how dangerous it would be, you don't know what is the strategy of the enemy, you have no experience about. And, uh, the enemy also changes its, uh, uh, types and style because this particular virus, which is mutating itself and it effecting in different ways and different human beings. So you don't get any information and reliable information on how to tackle it. I know we don't have any, sort of a cure, and how to fight it with. So that is why it is, uh, a mutated form of virus which is spreading geographically, from place to place, people to people.

Researcher ([03:35](https://www.temi.com/editor/t/H-TwOEKHuPxFPzNVwXj6i6VSr_mAQKhWTzUQyQBik4BVgltxTLvQcLlcQKKpHNItXufWKMCbB-RVy0SmiNW8WcAH65M?loadFrom=SharedLink&ts=215.43)): Got it. And how do you think coronavirus has affected the people in general in the world?

Participant: Oh, it has that effect in every sphere. And, and as I said, first of all, you know, you can't refuse, because it is a human to human transmission, and it is not from an animal or plant or anywhere. And the human to human transmission is the most dangerous thing, because if it were an animal or any outside agent without being human, you could try to eliminate it. when it is you, your family members that are getting infected, and in turn, they are trying not to infect you, but you have to just be away from them. So it is the fundamental thing where it is separating the loved ones, the nearest and dearest ones. And that, that psychologically, it is very, very distressful. And it has affected, as I said, in education, in business, in daily life, uh, in every sphere of our ways of living on this planet. That just why I meant is there's no end of, uh, giving you the examples, in every sphere of life.

Researcher: Absolutely. Now, how have you learned about the coronavirus? Do you remember?

Participant :Well, they first came into the press report, the media, uh, in the main frame, which we came to know sometime in the month of December, that there's a city, Wuhan, a province in China, where it was first reported. They didn't know what it was. First it was told that it was a serious and different type of pneumonia, and there was some, video visuals which we saw in the TV that people were getting affected, dying of it, and ultimately we came to know, again through media, that it is started in a sort of their seafood market, in that part of the world and then it spread in Europe and other parts of Asia, like South Korea, Singapore, and then right up to the West coast of America. And this is all media reports.

Researcher: So which news do you follow to get the coronavirus updates?

Participant ([06:37](https://www.temi.com/editor/t/H-TwOEKHuPxFPzNVwXj6i6VSr_mAQKhWTzUQyQBik4BVgltxTLvQcLlcQKKpHNItXufWKMCbB-RVy0SmiNW8WcAH65M?loadFrom=SharedLink&ts=397.75)): Well, all of the news and views are not 100% reliable, And, uh, I, I , almost see all the major TV channels of world, and, countries whether it is Asia, and that is where I sometimes find very contradictory and conflicting information about the, uh, you know, the actions and the reactions of different nations. And it gives an idea, I think, there is no cohesive, joined approach from the wider, whole organisation, to tackle it and it is all individual approach sometimes. Um, and I, it gives you, as human kind, an impression as if we are all living in different thoughts and in different ways instead of united and unified way. That is my perception that we should have a better approach through the united nations. But unfortunately, that organisation is old, vitiated with political influences by superpowers, or less superpowers of the powerless. That is a tragedy for the human kind.

Researcher: That's a very good point actually, to have all the nations working together.

Participant: Yeah. That's sad, that, Unfortunately, what I say, a unified approach is missing.

Researcher: So how would you think it could have done better? Like, if people were working together, how would you think?

Participant ([08:37](https://www.temi.com/editor/t/H-TwOEKHuPxFPzNVwXj6i6VSr_mAQKhWTzUQyQBik4BVgltxTLvQcLlcQKKpHNItXufWKMCbB-RVy0SmiNW8WcAH65M?loadFrom=SharedLink&ts=517.01)): If this sphere of influence in the global world was that prominent, if it was not between the existing so-called super powers and a rise in superpowers. I am also sharing this particular view, I think between two main powers, starting an economic warfare is trying to prove who is better than the other. And that is dividing, uh, the, the, the mankind into two spheres, sometimes we can say as the East and the West. One side it would be China, who seems to be the main culprit for not telling the world. And it's true, initially they were trying maybe to suppress information, which should have been shared. And that is a counter allegation, that it is from the West , i.e., the United States who had this thing and the same people. You know, these are all sort of much sling from one side to other. So the question of unified approach is out of, out of the system, because there is no strong international body, as I said, they are called united nations organisation, which is a lame duck organization, and there is no leadership in the united nations. It is just like a post office, so whoever pays you more stamp duty, he gets the faster delivery of the mail. That is what is happening right now. That I pay you more, here is my story, tell my story. I paid him less, he does not tell my story sort of approach.

Researcher: So this is, uh, so true from the current approach, looking at this. Could you tell us a bit about what do you know about coronavirus?

Participant : Well, I know it is, uh, a mutating virus, which used to reportedly live in the animal kingdom, whether it is a bat or a pangoline, or whatever. Humans have destroyed their habitat and the virus wants to find a new abode and a new habitat. So what the virus discovered that proportionately it's habitual place of residence has been destroyed by this humankind and they have got numbers from let's say from 3 billions to 7 billion in last 4-5 years. So the virus which also wants to live, has to find a new place and a residence or abode.

Researcher: That is very interesting!

Participant: They're coming, they are coming not to kill you, but they are coming to live with you. But their style of living is so virulent and so sort of aggressive that the human kind is not accustomed to it. And they are succumbing to their way of behaviours. Those who can withstand their aggressiveness, are not dying, which the virus does not want. He wants the humans to live, and to live within the humans. So, the mutual understanding and the balance of existence has not been reached yet because the humans are not prepared and this is new to them. They don't know how to accept and without getting perished and to allow it to live with them. So that is my understanding of the virus. It will moderate it's aggressiveness, it really take time, maybe one year, or two years, three years or four years, that I don't know, but the virus is going to stay. And this virus will live in humankind and both the virus and the humankind has to strike the balance of life, live and let live. The previous virus that has come, whether SARS or MARS, normal influenza, Ebola, AIDS, whatever in past. That is my understanding of the virus.

Researcher: Um, well, that's very informative actually. And, though you mentioned about China, about the origin of the virus. How do you think that they really appeared in the planet in the first place?

Participant ([14:03](https://www.temi.com/editor/t/H-TwOEKHuPxFPzNVwXj6i6VSr_mAQKhWTzUQyQBik4BVgltxTLvQcLlcQKKpHNItXufWKMCbB-RVy0SmiNW8WcAH65M?loadFrom=SharedLink&ts=843.36)): As I said, that the virus lived in the animal kingdom. When you destroy them and bring it to your table in your market place in your kitchen, the virus comes with you. They have not travelled at their own sweet will , you brought them in. And they also find that they have been decimated systematically over the decades, otherwise they were are happy there and in their normal habitat. Another example is, when Sundarbans is being exploited by the humans too much. Why the tigers would come and kill the human population leaving their habitat? Because you are encroaching into their normal natural field. You are destroying their food chain. Because you are destroying the food chain, you have to survive or before they allow themselves to be dead. So that is why they are attacking, it is that huge animal chain that you can see. That virus is a microscopically small. All humans live with millions of viruses and bacteria, because without viruses and bacteria we cannot survive. Right. Every human has, if we have, um, trillions of cells, we have millions and millions of virus and bacteria. And we have no problem with that. This is a new virus. We don't know how it behaves; the virus don’t know how to live. We go killing the habitat. So that is why these new sort of problem are going on. Once, as I said, it is done, it may take two, three years, which medically they say, to get infected. You develop your antibody. Once you have developed it, it will not kill you. But it will live in you. You may not know it is living within you.

Researcher: And how do you think, Coronavirus appeared in UK ?

Participant: Coronavirus appearing in UK is very easy. All of those people who went outside, they came back. There was no screening. Apart from those British, UK guys who went outside, went to different part of the world, they came back. They did not know they were infected, and there were no screening procedure. If you remember, even at the end of March, there were hardly any screening at the airport. And nowadays, international travelling is so frequent, people coming from all over the globe, to the city of London, Manchester and where not! From the English Channel and everywhere. That is how the virus came in- through the people.

Researcher: Absolutely. And, since the beginning of the pandemic, right, Like February, March, have your perception of the virus changed?

Participant ([17:32](https://www.temi.com/editor/t/H-TwOEKHuPxFPzNVwXj6i6VSr_mAQKhWTzUQyQBik4BVgltxTLvQcLlcQKKpHNItXufWKMCbB-RVy0SmiNW8WcAH65M?loadFrom=SharedLink&ts=1052.68)): Well, you did not know any means of knowing it, it is only through these guys who are- they tell you via, through the different channels, that it has mutated. In China it was one strain and in USA, it is different. In Europe it is a different strain. I have not heard much of all in India or other parts of South-Asia, but what is happening that this virus is affecting different people in a different way. Sometimes it was said that it is only the lungs, now, it is saying, it is kidney, it is liver, it is your meningitis, or passing into your brain, and so it is affecting all your vital organs. So, whether it has mutated or not, and it is all about the scientists and the research guys who has to control, uh, after examining the individual, or communicating the data or the overall data. That is very important. So the general perception, is yes, it has mutated at least 7-8 times.

Researcher: And personally speaking, like, what your attitude was to the virus in the beginning and what is your attitude now: Do you see any difference or it is the same?

Participant: See, initially when it came and it happened, there was more panic because all sorts of funny stories were going around. Now scientifically, you have to approach, first of all, whether it is man-made or not. Because you know, there had been a lot of, uh, unfortunate, uh, social media, reports, uh, that it was cultured, you know, biological warfare virus, developed by the people, who want to ride to the top of the world. Because many confrontation, you know, has become outdated, with every nations, having the nuclear capability. So, if there is a world war, it won't be fought militarily, it has to be either through bio- warfare or cyber warfare. So the style of world war, with the change of time has changed. So a lot of social media had been telling that it is a biologically developed. So, those who have developed must find out the cure. But maybe this is totally untrue, maybe it is really from the animal kingdom which came into the man, and now they want to live with the human kind. So again, the questions are because all sorts of, I mean, media propaganda is going on, and as I said again, between the East and the West. They are saying you have done this and you say, no, you have done these sorts of things. So if you ask me about my initial idea, it was an unknown panic. And as you know, after four months, we have come to the idea of living with it. Now you have to find a way how you live with it, and find the cure, like other kinds of influenza, pneumonia etc., by vaccine. So the race for the vaccine is going on, and let us hope that some country comes out with a dependable, reliable action.

Researcher ([22:01](https://www.temi.com/editor/t/H-TwOEKHuPxFPzNVwXj6i6VSr_mAQKhWTzUQyQBik4BVgltxTLvQcLlcQKKpHNItXufWKMCbB-RVy0SmiNW8WcAH65M?loadFrom=SharedLink&ts=1321.38)): Got it. So that is so true. Isn't it. And, do you think these pandemic is any different from any other we had in the past?

Participant :Well, this is in our all generation where we are seeing the first pandemic. The other things like SARS, MARS, Ebola, AIDS, it had not taken a helpless global situation. They had been mostly localized. Or, they had, uh, uh, created a problem for a limited period of time and in a limited geographical area. It is all the global, and from four corners of the world. And, sometimes without, even without human, contactless people that been affected. But now, it says, it does not travel in the air. But there has been instances where some lady who has never contacted anybody, for past three months, she has been affected. These are the story is that we see. We still don't know the modus operandi of this virus 100%. We know generally, that when you sneeze, when you cough, when you loudly talk, it goes 6 feet or 10 feet and airborne, and you know, the globules from the moisture of your mouth, et cetera, the droplets. Whether it is capable of floating in the air for longer period of time, that has not been established.

Researcher: Absolutely. And what do you think about your government's response to the pandemic?

Participant: It could have been better. I won't say it is measurable, though people are trying to say that, but, you see, they were very late and a sort of really laid back in taking action, because, uh, when it appeared, the things are getting out of control in December, January. The government in United Kingdom should have immediately acted upon it with haste and hurry and precaution. Unfortunately, they were not sure which way to roam. There's a lot of indecision, and because, and this is the first time they have come out of the European Union. The United Kingdom was observing, though not now as part of European Union, what Germany is doing, what France is doing, what Sweden is doing, what Italy is doing. And they were very late in taking preventive action. Preventive action means, distancing, screening, isolating. Even now, can you believe it, that we are talking about from June we will really start testing, but where in China, in South Korea, they have done that three months before. So, you can see, there's no point in blaming because if this government, the officials talk more but rather than taking action, too much talking, with too many cooks in the kitchen. This is one of the unfortunate thing; for every damn thing, you have got a big department, big collectors, big file, and numerous number of people. There are hundreds and hundreds of directors and co-directors, secretary, under-secretaries, and managers. You are top heavy with structures and there are a lot of confusion, but the action has been delayed in general. I must say, they would have tackled it in the month of January, February, without waiting for the March first week. They have delayed this for about two months.

I'm, by origin from India, not trying to put faith in the Indian administration. I say, people called third world developing country, look, one country with 1.3 billion people. Okay. They don't have the testing facilities, but whatever they have done, they have done more than 120,000 testing, which I am told. They have fallen for the things that the recovery rate is bad, and daily affected is only about 3000, like I said. Ok, it has not been reported, like I said, people would say, oh, Indians, they do not report. Take it 10,000, 3 times more in a country of 1.3 billion people. Instead of 3000-4000, I take it as 10000. It is nothing. Where in UK, with 240 million, 260 million people lost 50,000 dead, almost. It is 36, and if you take all the unreported and bla bla, it will be 45. I am sure when the final figure comes, it will be 50000. So, what is this?

Researcher: Got it. Yeah, it's worrying. Yes, certainly. What was the information about coronavirus that surprised you most?

Participant ([28:03](https://www.temi.com/editor/t/H-TwOEKHuPxFPzNVwXj6i6VSr_mAQKhWTzUQyQBik4BVgltxTLvQcLlcQKKpHNItXufWKMCbB-RVy0SmiNW8WcAH65M?loadFrom=SharedLink&ts=1683.06)): No, coronavirus report surprises- you see, as I said, in a generation of ours, after the Spanish flu, which is part of the history, we have only learnt in history- the impact of coronavirus, because it is human to human and it is sort of a Spanish flu, which was not given more than seven days. Okay. You get infected. And within 7 days you are dead. But this particular virus is very clever. First of all, it does not tell you that it has entered your cells. And then it takes an incubation period of 7 to 14 days and then extracts to show its ugly face. And then it takes up to 28 days before you have your turn. The other dangerous thing is, the symptoms are like standard flu, with which we are accustomed for so many years and we have learned to live with it; now whether it is flu or COVID-19, there is no way of knowing it, before 7-10 days. By that time, the damage has been done. And it has affected the population, and that is what is most panicking. That somebody silently coming within you, living within you, without any sort of symptom, and pretending it is nothing but a standard flu, and then overnight it changes colour and then you'll know that you are at the point of no return. There's no coming back. You are more of less confirmed to be dead.

Researcher: Absolutely. Absolutely.

Participant: That is what is the panic.

Researcher: And when you discuss coronavirus with other people like your friends, relatives, what do you mostly talk about?

Participant: We don't talk about the virus, honestly speaking, we don’t know nothing it. Even now what the big scientists coming into the TV channels blabbing and saying something, that they have got a few data, but the authentic, reliable, verifiable information is not there. The only thing I say that try to save yourself by social distancing, particularly to the elderly. Don't go out and do your physical exercise as much as possible. In fact, even if you go to park for a walk, be absolutely careful, and take all the precaution that is possible, But, it is granted that if you have gone out, the virus had gone in some part of your external body. Make sure it has not gone inside you.

Researcher: That is what is, absolutely. And if I can ask how your personal life has been affected by the pandemic?

Participant ([31:23](https://www.temi.com/editor/t/H-TwOEKHuPxFPzNVwXj6i6VSr_mAQKhWTzUQyQBik4BVgltxTLvQcLlcQKKpHNItXufWKMCbB-RVy0SmiNW8WcAH65M?loadFrom=SharedLink&ts=1883.7)): Well, honestly speaking, I am a retired person. I miss the freedom of going out whenever I feel like, but otherwise, at my age, uh, I can't complain because, uh, I have, help in my daily life living in terms of weekly shopping and all these things have been done by my daughter, fortunately, she is with us. So I have nothing much to complain about- the only thing is that psychological pressure or uncertainty- I mean, not very delightful, that we don’t know where we stand right. The degree of uncertainty that you may be infected unknowingly tomorrow, at this age, that is something which is psychologically affecting now. Physically, I do not have anything to complain about.

Researcher: Do you see any positive effect of the coronavirus on your life?

Participant ([32:48](https://www.temi.com/editor/t/H-TwOEKHuPxFPzNVwXj6i6VSr_mAQKhWTzUQyQBik4BVgltxTLvQcLlcQKKpHNItXufWKMCbB-RVy0SmiNW8WcAH65M?loadFrom=SharedLink&ts=1968.85)): Oh, yes. yes. On my life, on my life, introspection is something which I used to do. But it will be more effective for the younger generation, because our time is really limited. Even if I do introspect, I have nothing much to contribute to this society and the community, because it is only limited period for which I am there. So if the younger generation, introspect, and if the take lesson that on this planet, you are one of the species, like millions of others, that you are dispensable: you are not indispensable. And mother earth or nature will be much better without the human species. If all men and women die today, they mother nature will flourish, the animal kingdom will flourish. Rivers, lakes, sea and the ocean, everything will flourish. It is the human species which has systematically destroyed for its own benefit and for its so called development and progress. Now if the new generation who will be coming over the next 30, 40, 50 years, if they realise we have to take care of the planet on which we live, because we can't go on exploiting this planet and the mother earth for electricity generation, whether it is mining, millions and trillions of tons of carbons and automobiles. I mean, stop the development. Stop less using the modern gadgets, of the high speed cars, more cars on the street because the planet cannot bear anymore. The best ways is to learn, and it is a forceful three months. Let the world decide that for three weeks, at an agreed time, we will close everything. And we will allow the lungs of the mother Earth to flourish.

Researcher: That's a very good proposal.

Participant: You know, we have only given two months that the whole airplanes, train, traffic, cruise, ship, everything, factories are closed. If we can survive three months, why can’t we do it for 3 weeks, globally? Because one time will not do anything. Few generations will take at least three to four weeks and you see how the plants, birds, animals are enjoying?

Researcher: So do you really think that humans will take a lesson from the pandemic and can do better?

Participant ([36:23](https://www.temi.com/editor/t/H-TwOEKHuPxFPzNVwXj6i6VSr_mAQKhWTzUQyQBik4BVgltxTLvQcLlcQKKpHNItXufWKMCbB-RVy0SmiNW8WcAH65M?loadFrom=SharedLink&ts=2183.12)): You know, the people who work for profit, they will ignore it. They would say it is my human rights. I am the Supreme species. I dictate the world. You are powerless. You can have an atomic warfare, you can have a neutron bomb, they should realise how powerless they are. Not naming the president, the generals, the field marshals of any country, you have nothing, you are less powerful than the virus. And humans are really the virus for mother earth. If this one virus goes away, the world will be flourishing, the planet would look much brighter from the outer space, they will say, 'uhh', this is the planet, mother earth, which is glowing blue.

Researcher: Yeah, that's a very good perspective. I have not thought of that way that humans are dispensable in that way! So coming to the last question of the first part, um, how do you think this pandemic can end?

Participant ([38:08](https://www.temi.com/editor/t/H-TwOEKHuPxFPzNVwXj6i6VSr_mAQKhWTzUQyQBik4BVgltxTLvQcLlcQKKpHNItXufWKMCbB-RVy0SmiNW8WcAH65M?loadFrom=SharedLink&ts=2288.6)): Well, first of all, the humans will have to have their mental, commercial and market oriented pandemic to be arrested. You see, you cannot be market driven, you cannot be business driven, you cannot be profit driven. You have to give and take. Each multinational companies, who earns billions and trillions, they should take a pledge, that we will plant 1 million trees, every year. If you plant trees, the animal kingdom will flourish, the world climate will change, your health, your general expenses for healthcare will be reduced, and you will not have the modern diseases like Ebola, SARS, AIDS, cancer, these will all reduce. You know, 100 years back, these sort of complications were not there. How much money the countries just spend for fighting the, you know, the obesity disease, how much, how much mental cases are there all over the, particularly the western world, billions and billions of dollars spent for the mental care of these people. All of these will go. So, you have to tell these multinational companies, millionaires and billionaires, who are producing the wealth from the mother earth, then give it back! Some of it. That is the lesson which human should learn. If they don't learn, you have to promulgate, come together, do some brainstorming with the people who have vision, not the idiot political leaders and whose vision is only limited to how many millions I make tomorrow. How many flights of plane I bought, how much business I am doing with the Middle East oil rich countries, or how much I am doing with Africa, how much I am just exploiting the African continent, where the people are dying. And I am taking the wealth out of it. Unfortunately, you are not being able to implement it because you don’t have the modus operandi and the apparatus.

Part- II

Researcher: Correct. So we have ended our first part and it was very interesting to hear your views. In the second part, which I will start now, will be focused on the South Asian community. Okay. The first question I ask is what do you think are some of the health concern for people in the South Asian community during the pandemic?

Participant ([41:09](https://www.temi.com/editor/t/H-TwOEKHuPxFPzNVwXj6i6VSr_mAQKhWTzUQyQBik4BVgltxTLvQcLlcQKKpHNItXufWKMCbB-RVy0SmiNW8WcAH65M?loadFrom=SharedLink&ts=2469.96)): Well the concern is, that many of the South Asian community people have got underlying healthcare conditions- it is diabetes, it is high blood pressure, it is asthma and, uh, old age related other problems. I'm not talking about those transplant people and heart operations, people and all that. Um, South Asian community particularly, do not have the habit of regular exercising. This is one of the main topic.

If you look at the white people, uh, they are much, much more agile, much of sort of physical activity oriented. You see, the South Asian community do not indulge in many kinds of sports activity, outdoor activity, they are stuck on mostly, on gossip all the time, you know, then too much of political discussion, they are couch potato, watching tv, sports. Therefore they have an underlying health condition. Specially the elderly group, South Asians, Bangladeshi, Pakistani, Indians- it is all the same, because the South Asians, compared to that, the Filipinos are more outgoing that they're getting together, dancing, sports, bla, bla, and all sorts of things, but India, Pakistan, Bangladesh, Nepal, they don’t do it.

Researcher: Correct. Correct. And how do you think your, the South Asian community has specifically been affected by the coronavirus?

Participant (44:30): Well, they have been affected because of the, uh, the underlying conditions and, uh, many have not taken the right precaution. The other thing is that, um, perhaps, they live too many people jointly, uh, that is one of the factors, where the social distancing is not observed. So, they're unknowingly transmitting these disease, that is a concern because they think they are well, but they may not be well. And they only come to note when they are affected, when it is too late because many of the community members have already been affected. There is no way, you know, there is no way of knowing it, because if you have a test today, but after five days you may test positive. So you don't know when you are going to get to that. That is the main difficulty.

Researcher ([45:35](https://www.temi.com/editor/t/a0N2XiEi2Btv0Lpp0U7r76YFIO0kKxIFduIVTerG9vkPd-bbTOVsTAvC12ITM3ixYKjm6CJngVmfPViPey5RkI52MFQ?loadFrom=SharedLink&ts=275.67)): And if I can ask how your own family has been affected by the coronavirus?

Participant: Well, it has not been affected much in the sense my daughter can work from home, which she is doing and since myself and my wife, we are, retired already. We have no compulsion of being affected because I said that our needs are taken care by my daughter. So personally, I have not been affected.

Researcher: Good to know that. Good to hear that. And, do you think, the South Asian community may be more affected by the coronavirus than the white people?

Participant: They will be, will be. Unless, unless, uh, they can take more precautions and unless they try to segregate the elderly from the younger ones, because in many South Asian families that is not happening, not that they do not have bigger living spirit, but because of their habit, uh, this is something they have to change and they have to take the precaution, or really getting more effected. I'm not sure about the medical care I but I don't think there is any discrimination. Uh, well, I wouldn't be able to give any comments on that because I think with all due respect to NHS, they treat everybody fairly and although there has been some who pointed out to their partiality, but I don't subscribe to that.

Researcher: Hmm. So do you see any particular difficulty in the South Asian community to access NHS?

Participant ([47:41](https://www.temi.com/editor/t/a0N2XiEi2Btv0Lpp0U7r76YFIO0kKxIFduIVTerG9vkPd-bbTOVsTAvC12ITM3ixYKjm6CJngVmfPViPey5RkI52MFQ?loadFrom=SharedLink&ts=401.32)): Well, as far as I know, there is no difficulty in accessing. At least I do not have personal, uh, information except that the family whom I know, although the families, one member is a GP, the other one is a sort of a research guy. Initially there was a little confusion about which hospitals to take, but ultimately, it was delayed by half a day, but then it was sorted out. But that in spite of being a GP, the wife being a GP, in that sort of a time. I'm not sure if it had been an ordinary person, whether it would have taken more time. I have no information because I have not come across any such information from any reliable source anything. I have nothing to tell you about that.

Researcher: Hmm. And, the government's measures like social distancing, working from home, etc., do you think there are any specific difficulties that the South Asian community face to follow them?

Participant ([49:06](https://www.temi.com/editor/t/a0N2XiEi2Btv0Lpp0U7r76YFIO0kKxIFduIVTerG9vkPd-bbTOVsTAvC12ITM3ixYKjm6CJngVmfPViPey5RkI52MFQ?loadFrom=SharedLink&ts=486.19)): I don't think they should have any difficulty in following it, no, they shouldn't do it because, you see, it's equally applicable for all the communities. So you do not know what to do and what not to do. So, you got to be self-disciplined, not because government is telling you; government may say whatever it is, but it is your own responsibility, for your own health, safety and security. You know now, what to do, and what not to do. So it has to be self-disciplined. Forget about what the government is saying, if you are not responsible enough, you behave responsibly, that’s all you need.

Researcher: Hmm. And do you think that has been the same, sipped in the community in a similar fashion?

Participant: As far as I know, um, my community, uh, we are from the part, where in India, I see everybody is just taking things seriously and behaving responsibly. I have no, I have no qualm about that. And I know everybody are responsible and behaving in very complaint ways to the rules and regulations.

Researcher: Right. And how much do you think the South Asian people, they trust the government that they are making right choice for them?

Participant: Well, it is again, the general perception as I can hear. I do not know why this belief is there. That there is, a little racist approach is there. Not only the South Asian, even the African origin, the African community who have come and settled here and are second generations, I have seen in tv, even in the British Broadcasting Corporation, these allegations have come up and they were saying, in the support, that the black community has been neglected, and they're not getting that sort of facility like the white, I heard that, but I have no first-hand knowledge or information, but I have not come across any of these in South Asian community saying about this. But, you know, this is a historical fact that the British has got a sort of a slightly racist approach, embedded in their system. Not for this coronavirus, even otherwise for, for maybe last 60, 70 years, which is perhaps to some extent that you have to accept and live with this. Why? Look at the human psychology, it is their land, where the immigrants have come. Maybe they needed to run the wheel of development because by themselves they were not sufficient. So the Asian community, the African community and the other communities came in. And they contributed to the development of this country, ok, which the British themselves, they accepted. But in that process, that is also an underlying sort of a ceiling where the white kids get more priority when it comes to a competitiveness. If things are available, then ok, but when it is a choose and take, then some sort of undeclared partiality, unspelt partiality works as an incurrent psychology and the people who are disbursing the facilities.

Researcher: So now this is to some extent human nature, which you can't much complain about!

Participant: So there's not much point in complaining because in general, my experience is that I've been fairly and squarely treated. Even in India, there is another type of power gap. If you have more money and more influence, then it sounds wonderful. Everywhere, there are some underlying condition, nothing is perfect in this world. That bit of imperfection if it is there, you have to learn to live with.

Researcher: So that's very, very interesting really, and your point on that. What would you think to what extent that people, South Asian people, they understand the health messages, which are given by the government?

Participant: Well, understanding is there, but the attitude is perhaps not cooperating. They understand it very well, but their ability to cooperate is not that good. I think so.

Researcher: Hmm. So why do you think, that can be any different than the white people?

Participant: It is because of the indifferent attitude, not that day I have the wilful intention of not cooperating, but, ‘could not care’ attitude, lack of attitude. That is somewhere there.

Researcher ([55:19](https://www.temi.com/editor/t/a0N2XiEi2Btv0Lpp0U7r76YFIO0kKxIFduIVTerG9vkPd-bbTOVsTAvC12ITM3ixYKjm6CJngVmfPViPey5RkI52MFQ?loadFrom=SharedLink&ts=859.89)): And that is hindering them to follow that rule here. Mm Hmm.

Participant: I have, I have seen about the mosque- I am not saying about the temples, because they are all closed- because during the Ramadan, I have been there, and I have seen, people don't give a damn about it. I'm not trying to portray a particular community. But the indifferent attitude, that is there.

Researcher ([55:57](https://www.temi.com/editor/t/a0N2XiEi2Btv0Lpp0U7r76YFIO0kKxIFduIVTerG9vkPd-bbTOVsTAvC12ITM3ixYKjm6CJngVmfPViPey5RkI52MFQ?loadFrom=SharedLink&ts=897.19)): So would you say not only the people visiting mosque, but also other people have the same attitude?

Participant: I mean in all places of religion, you have to maintain social distancing. And you know, religion can wait, but your life and death, if it is at risk, why do you take risk? Your life is more important than praying. You can pray from your home, you don't have to go to your place of worship because, God or Allah, everybody are everywhere. It is not only inside the mosque or the temple. Wherever you are, he is around you. It is the lack of understanding, and then, you know, it's herd effect, if everybody is going, why not me?

Researcher: Got it. And how do you think then the community messaging could be done to approach or to make them really understand and how can government do this?

Participant: It is not the government. It is the local leaders. It is the community leaders. The government can't just go everywhere. You have to trust the community, the local people who are responsible, they should take initiative and they should, if it is happening somewhere, they should go and take initiative and tell the folks that it is not for anybody, but for your own good. So you behave the way it is required, because if you wish to fight, you just go, get contaminated and get finished. So it is not that they don't understand it is just the community people who should tell them.

Researcher ([57:54](https://www.temi.com/editor/t/a0N2XiEi2Btv0Lpp0U7r76YFIO0kKxIFduIVTerG9vkPd-bbTOVsTAvC12ITM3ixYKjm6CJngVmfPViPey5RkI52MFQ?loadFrom=SharedLink&ts=1014.07)): Do you see any other issues, which is hindering them to understand like barriers, anything else there are in the community, so they don't understand?

Participant: I have not. Honestly, I have not come across because of my restrictions, my movement restrictions, I have not come to any community or people who do not understand. I think people are generally well informed. There is not a single individual who do not know by now, what is coronavirus and how harmful it is to the mankind. So the message is already there. It is the question is of habit of implementing.

Researcher ([58:46](https://www.temi.com/editor/t/a0N2XiEi2Btv0Lpp0U7r76YFIO0kKxIFduIVTerG9vkPd-bbTOVsTAvC12ITM3ixYKjm6CJngVmfPViPey5RkI52MFQ?loadFrom=SharedLink&ts=1066.36)):Got it, got it. And finally, what do you think has helped yourself and also the South Asian community to deal with the coronavirus crisis at the moment?

Participant: Well, the South Asian community I know, they have maintained the sort of, uh, distancing. Not only social distancing, they have confined themselves, all the elderly people. I know at least 200 to 300 people and families doing it now, except the younger folks. The elderly are all at home. Yeah. Maintaining distance. They don't go and meet the children and all that. Even their daughter and sons in-laws bring the food, they leave it at the door, and they do all the cleaning and the precaution that is necessary. They are fully aware of it, and they are following it. They are so far, so good!

Researcher: So just following the rules that has helped you. Right. Right. So that is the end of the interview. And do you want to add anything or do you think that is important?

Participant: No, no, no. I think I have, shared a lot. I have, I have volunteered much more.

Participant ([60:14](https://www.temi.com/editor/t/a0N2XiEi2Btv0Lpp0U7r76YFIO0kKxIFduIVTerG9vkPd-bbTOVsTAvC12ITM3ixYKjm6CJngVmfPViPey5RkI52MFQ?loadFrom=SharedLink&ts=1154.991)): All right. Thank you so much!
