# Supplementary material for: Mitigating the identity and health threat of COVID-19: Perspectives of middle-class South Asians living in the UK
Source: J Health Psychol. 2021 Jun 22;27(9):2147–60. doi: 10.1177/13591053211027626 (PMC9353968; doi:10.1177/13591053211027626)
Supplement: sj-docx-2-hpq-10.1177_13591053211027626 – for Mitigating the identity and health threat of COVID-19: Perspectives of middle-class South Asians living in the UK [file sj-docx-2-hpq-10.1177_13591053211027626.docx]

Please find attached the (1) raw transcribed data for this study, (2) the coded data output from the MAXQDA2020 analysis in the form of (3) an overview of codes and (4) an overview of coded segments, and (5) the data structure table with example codes.

Raw data (1) was uploaded to MAXQDA2020 for initial coding (4) and pruning into a hierarchy (3) based on an inductive analysis. From this analysis, the data structure table (5) was created by the first researcher and discussed with the research team in advance of preparing the manuscript draft.

Notes in the analysis refer to participant numbers that were later changed to names as follows:

| **Participant number** | **Pseudonym** |
| --- | --- |
| 1 | Asha |
| 2 | Mohan |
| 3 | Jyoti |
| 4 | Ashok |
| 5 | Pradeep |
| 6 | Anita |
| 7 | Dilip |
| 8 | Mohamad |
| 9 | Latha |
| 10 | Sudir |
| 11 | Mamta |
| 12 | Rekha |
| 13 | Raj |
