# Supplementary material for: Mitigating the identity and health threat of COVID-19: Perspectives of middle-class South Asians living in the UK
Source: J Health Psychol. 2021 Jun 22;27(9):2147–60. doi: 10.1177/13591053211027626 (PMC9353968; doi:10.1177/13591053211027626)
Supplement: sj-docx-4-hpq-10.1177_13591053211027626 – for Mitigating the identity and health threat of COVID-19: Perspectives of middle-class South Asians living in the UK [file sj-docx-4-hpq-10.1177_13591053211027626.docx]

**UK 1 GROUP A - AMA PT. 1 and 2 – TUSHNA VANDREVALA – KINGSTON**

**I:** I would like to have your understanding on what is happening in the current around this Coronavirus crisis. Are will start with the first question on, what do you think is happening to the world?

**R:** First, it’s a health concern. A health issue. The safety of a person’s life and secondly I think it’s at first there is a huge economical impact, which will effect our lives as and when we go back to the normal life again.

**I:** When we are going back to that. What comes to your mind when first you think of Coronavirus?

**R:** I didn’t hear you. How the virus is reacting and how it’s effecting each person.

**I:** That is absolutely, yes.

**R:** Not knowing how my body is going to react to it. Just looking at someone else’s case. Also, it’s how quickly it spreads and much precaution we need to take to prevent ourselves from getting sick.

**I:** Can you tell me a bit about what you know about Coronavirus like maybe from the media and maybe from scientific journals, newspaper, anything else?

02.00

**R:** Of course, it’s a viral infection, which is almost like flu. It’s a much more severe type of flu. It’s mostly spread through droplets from cough and while you were talking it might spread through your droplets or body fluids or if you touch something, if you cough on your hand and touch something then you might be spreading the germs on the surface. The effects of it, it the symptoms the general symptoms what you get like the flu like symptoms like coughs, colds and effecting our lungs much more in a severe way. And then, of course, which resulted in some cases which has resulted in serious lung issues and death. This is my understanding of it.

**I:** Anything deeper with respect to like the biological—any scientific understanding you have with this spreading to the lungs? Do you have any further understanding of the biological like how it spreads and how the infection spreads? What happens in the body?

**R:** It’s breaking up. Can you say that again.

**I:** I am asking you that do you have any more scientific understanding of this Coronavirus? How it spreads like the sail-wise bodily and how it reacts, impacts? Can you hear me? Can you hear me?

PART 2

**I:** In your opinion, how did Coronavirus appear? It’s a very tricky question. How do you think it appeared?

**R:** I will go into different speculations of course. There are a lot of speculations going on. Some are saying, this is naturally offering virus, which micro-organisms they always change. Some would say it’s naturally awkward. Some would say, maybe it’s man-made. At this point, I think it’s our priorities should be not thinking of how it came. If it’s man-made at some point, if it proves that it is man-made then it will help to find a cure for this. At this point, it’s more important I think to concentrate on how we can deal or deal with the virus in terms of how we can help the patients with medications or vaccines and what we could do and how we can go back to our normal life as quickly as possible.

01.32

**I:** Again, how do you think it happened in the UK?

**R:** My guess would be it’s through human carrier. UK is, of course, London specifically it’s a hub for tourists. There is a constant flow of foreigners coming to the country. Not only foreigners, but people travelling on a regular basis to other parts of the world. It can be brought by any one person or maybe multiple persons. The way the virus works and it can be spread from one person to several others in a very small amount of time.

**I:** Do you think in your opinion that the UK is more or less vulnerable to this virus? What would you think? How you would place UK in the picture that how this can effect UK?

**R:** Scale wise, I would say it would effect the same way it is effecting any other European countries, any other countries, not in the European countries, but in the other countries.

**I:** You see no difference.

**R:** I would think it will not effect in any different ways. It’s going to be the same for everyone. Everybody will have to go through the similar difficulties in the future.

**I:** Since the beginning of the pandemic, have you any perception about Coronavirus changed?

**R:** No, not much.

**I:** This is the same which you had like since the beginning?

**R:** Yes.

**I:** That is quite interesting because it’s like the knowledge which you are having and putting on. In that manner, looking at the pandemic, do you think that this pandemic is different from any other we had in the past? No in the UK, but anywhere in the world?

**R:** We never thought that it was going to turn into a pandemic. It was a serious disease at that point and of course, people were affecting, it’s spreading like other epidemics, let’s say. Initially, yes, we never thought that it’s going to turn into a global incident, where most countries will go into lockdown. Yes, in that sense, yes, my view on or my knowledge about Coronavirus has changed from the very first point when I heard about it. How is it different from other pandemics, yes, in recent years, we never had a pandemic and in the last ten years, last twenty, ten years we have changed, the society has changed.

**I:** What do you mean by saying that society has changed?

05.54

**R:** Society has changed in the sense, we travel more than what we used to do ten years ago. It has, the world has become smaller. In that sense, it probably triggered or added to this pandemic that it spreads so quickly to different countries, because people are travelling from one place to another. Like I said, it’s, that has changed immensely in the last ten years, ten, fifteen years, probably.

**I:** What about how do people live apart from the travel? Do you think there is any social change which can have affected this pandemic?

**R:** In terms of social change, not really, I don’t think so. Social change in the sense—how we are behaving during this pandemic, that’s probably quite a bit of a difference from the last pandemic this world has seen, because we have the access of internet and we are saying how we are getting our food supply on a regular basis and so all these things definitely didn’t happen during the last pandemic. In that terms, society has changed, of course. In other ways, in terms of habit in different societies and like we said, the travelling.

**I:** So like people’s attitude, behaviour, do you think it has changed or are the same like when you are comparing between like ten years or something which could have affected the spread of this pandemic, as such.

**R:** When it came to the spread, I don’t think it has.

**I:** That makes sense, yes. How about like, what do you think of your government that is the UK government’s response to the pandemic? How much do you agree with how they have dealt with it or can you suggest a better way or how do you think they have dealt with this crisis?

**R:** One thing probably they could have started the lockdown a week before or put more check on the borders, so that we could have probably reduced the spread from an earlier stage. And also, a more clear guidance to common people, probably that was lacking in the beginning. That what you could do and what you can’t do and to convince people to follow those. They probably could have done it in a different way.

**I:** They could have done better in that sense.

**R:** Done better. Some specific instructions that [tape breaking up] like when the lockdown started, we were told that we can’t go out. We can only go out once in a day. Before we were just asked to stay at home. But it should have happened from the beginning itself that specific instruction that no, you have to stay at home. That could have been implemented much earlier.

**I:** Can you see any good points to how they have dealt with the crisis?

**R:** At this point, yeah, yeah—do you want to finish the question?

**I:** No carry on. Carry on.

**R:** Okay. Yes, at this point, how they are handling it I think NHS not getting the support NHS is getting and the support they are providing to the general public that **[Unclear]** 11.02. I know they have started some other, some other sections which was earlier closed. In the last month, probably there was some departments that were closed completely and now they are restarting those through different medium, through video calls of through telephone calls, which is another good thing. As of now how they are handling it, I am pretty happy with it.

11.35

**I:** Video calls and conference with like other trades or the other functions.

**R:** No, no. NHS I am talking about. NHS, the other health departments let’s say not **[Unclear]** 11.52.

**I:** How have you learned about Coronavirus? From where?

**R:** Through media.

**I:** What would you say like which media?

**R:** General news? Newspaper or online news? Online updates?

**I:** How about the role of social media?

**R:** Yes it has been, how in the role of social media in the sense of **[Unclear]** 12.51 the knowledge about it.

**I:** How you have been—sorry, it’s breaking up. I am asking again, okay. It’s like how have you learned like your knowledge from various social media has affected you?

**R:** To be honest, I have been trying to avoid the social media in that sense. Just to avoid unnecessary panic. I have been mostly relying on the news I am getting from the proper news channels, rather than rely on the social media about what was happening around or different information.

**I:** Why do you say that it can create panic?

**R:** It’s when it comes to social media, there is an overload of information or in general terms if I search on Google, my experience has been there is always an overload of information and it’s very difficult to filter from there which information is correct and which one is incorrect. That is why I have been trying to avoid the social media information regarding the pandemic.

**I:** Do you watch television news?

**R:** Yes.

**I:** Do you think that helps you to your knowledge?

**R:** Information on the news channels itself or just general online media you might say. I have been getting information from there, rather than just going to searching all by myself.

15.10

**I:** Is there any particular website that you use to have a regular update?

**R:** I use my Apple. Apple News. Apple app, yeah. It’s mostly BBC, Telegraph, Guardian. Regular news.

**I:** Like people that use a certain website to get the numbers like how many people have been affected by the day. Do you reach to any such information website?

**R:** Yes, I did and so it’s a **[Unclear]** 16.03 which I have been checking. I was checking that every now and again.

**I:** Is there any information about Coronavirus that has most surprised you?

**R:** I think it’s the whole incident. It’s turning into, Coronavirus itself turning into a pandemic has surprised me.

**I:** Why do you think it may have surprised you?

**R:** Because it never happened in my lifetime. Most of the time we see it on Sci-Fi movies that this might happen. But in truth **[Unclear]** 17.00. This reminds us that those Sci-Fi’s are based on some truth. We have to take those things into account.

**I:** Happening in your life.

**R:** Yes.

**I:** Now coming to the part like, when you discuss Coronavirus with other people, how does it go? Like with anybody you are discussing it with, what do you feel?

**R:** What do I feel when I talk to people? How do I feel? In what respect?

**I:** Like you are discussing that with a friend, with a member of your family. When you are discussing that with them, any particular aspect do you discuss or it relates to what particular ideas you want to share? What do you do? Like you are discussing that with a family member. With a daily update from a friend. What would you discuss and how the conversation would go about?

**R:** It’s mostly the discussions are based on how to take precautions. What we can do to protect ourselves. And then, the affects of this pandemic or effects of the complete lockdown and how it’s going to affect our lives in the future, probably. And how it’s affecting our current life. Surviving on a daily basis.

**I:** Can you share a bit about how—

**R:** Sorry?

**I:** Can you hear me now?

**R:** Yeah, yeah.

**I:** Can you share how your personal life has been since this pandemic?

**R:** My personal life has been—

**I:** Your daily life.

**R:** I have, my daily schedule has changed drastically, of course. We have a very busy household and I have two little children, two little kids. Their school and their activities and then my own work, my husband’s work. Everything has stalled in a sense. Everything has changed and we have to readjust ourselves living under one roof. Living and working under one roof and not going to the office and not going outside. Assigning individual cases to finish our jobs. The kid’s routine, of course, that has changed completely now the home schooling and everything that has changed. Of course, their schedule has stayed the same. In another sense, as of now in the last two or three weeks we have been staying home. We are not going out anywhere. At the same time, I feel good about it at times, because yes, life has slowed down a little bit. It gives you the chance to probably reconnect with each other and just think about life in a different perspective altogether.

**I:** That is good and bad side.

**R:** It makes me feel—yes, it has its good and bad side, so, yes.

**I:** What is it you are learning about your daily life? Any new perspective you have gained since the pandemic?

**R:** My daily learning.

**I:** Say again?

**R:** Yes. It’s to slow down at times.

**I:** You have more time now?

**R:** I have more time. But that is what I am realising that I don’t give myself the time I need to give. It has made me, it has forcefully made me realise that I need to slow my life. We are always rushing. We are always running. It has slowed down a little bit.

**I:** Do you have any idea on how this pandemic is going to end?

**R:** No. Unfortunately, no idea. I would love to know.

**I:** Like when? How? Why you think you cannot tell?

**R:** Around this virus that we are still learning how it works. And also it’s not finding a definite cure or how we can support who are affected. It’s not knowing that and that makes it very difficult to say that when it’s going to end. At the end of the day, it’s a micro-organism and so we can’t really control it. We have to learn about it and we will have to find a proper barrier to protect ourselves from it whether it’s a vaccination or whether it’s medication or whether, in some other way, some other unknown way. It’s learning that and we haven’t really finished that learning yet.

**I:** Absolutely. That is the last question which I have for part one. How do you think we can prevent further pandemics like that in future?

24.20

**R:** I think it’s not completely—it’s quite unfair to say or it could be quite foolish to say, remains to say that there won’t be another pandemic in the future. Again, this is, if it’s a natural occurrence then it can happen in the future, at any point of time. But yes, how we can, what we can do probably will take or when all this ends and when we go back to life maybe do necessary changes as a society or as a country coming from maybe the government can take some steps learning from this experience that what they can do in future to support people in a more effective way. Maybe give more support to our health system. And yes, yeah, it’s just learning from it and prepare ourselves in for any future occurrence.

**I:** Right. So, now, thanks for your contribution. Now I am going to ask you some more specific questions about the current crisis. How it relates to your community? What do you think there are some of the health concerns for people in your community do you think the pandemic?

**R:** Some of the health concerns which I have heard or which I have seen that were our South Asian community is more prone to hypertension and diabetes, which tends to be the COVID-19 the virus seems to be affecting in a much worse way to those who suffer from these two diseases. In that sense, it’s probably affecting the South Asian community more.

**I:** Correct. Do you think that people in your community are more or less at risk in the pandemic? You mentioned that about the diabetes and the basic health and hypertension, etc. Anything you can think of like why do these people can be more at risk and more or less at risk?

**R:** There can be two other reasons, one is a lot of these people are involved directly into key worker jobs. They are more exposed to the virus. The second this is probably the living conditions. Can you pause it for a second.

**I:** Yes, carry on, please.

**R:** Sorry about that. It’s one, like I was saying earlier, it is, in a sense, one is the health aspect. Second is, a lot of them are involved in key worker jobs which exposes them to the virus even more. The third probably is their living conditions. Not everyone coming into this country. A majority portion of these people coming and living in this country as asylum seekers and they don’t always live in a very, they don’t always have the best of the living situations. That can probably sometimes prevent them from reaching out for help when they are unwell, maybe or they might want to hide and they don’t want anyone else to know about them in fear of losing their job or they probably losing their housing or whatever, the accommodation they have. They risk their lives more just to hide themselves from others for the fear of getting caught, which probably contributes to higher numbers to some extent.

**I:** I think that is a very very—core of the thing which is going on. They are not coming up and they are not getting help for such conditions.

30.21

**R:** It’s not only whether they can seek help or not. Sometimes they probably don’t have the proper communication skills or the language to approach others to let them know about their difficulties. Sometimes, it’s probably—they are just scared. They don’t know. They don’t have the proper information of where they can seek help or if there is any help available at all or not. Things, already they are in a very vulnerable situation. There could be one section of the people who will probably take advantage of their situation and will demand more money or they can threaten them by saying we can evict you or we can do this and we can do that. They won’t know. They won’t come forward or they won’t know where to go to seek help. They will just give whatever problem they are having, they will just give them, keep it themselves, rather than reaching out and getting the proper help when, if they are unwell or when they are unwell.

**I:** How do you think and why do you think this can be different from other white people? Their behaviour towards this can be different from any other white British people?

**R:** I know white British in the sense, of course, it’s—If I particularly say the British white British people, of course, they are in their own country. Communication wise they are in a much better place. They don’t have the language barrier which other sections have. So, of course, their living condition wise. You are not an immigrant or are you not a migrant at the end of the day.

**I:** Absolutely. Not being an immigrant.

**R:** Yeah, yeah.

**I:** Do you think your family might have been affected likewise on the health conditions or anything you mentioned by the Coronavirus? Has it offered to you that it might be, your family maybe more vulnerable to it?

**R:** Yes, that’s always there in the back of my mind that my husband has hypertension, high blood pressure and so if it comes to me, I worry about that, whether he is more vulnerable than others. It’s the control of course. That doesn’t completely keep us in the clear. Hopefully, it is not going to happen to us, any of us.

**I:** The government has now introduced a lot of different measures as you know to contain the virus, for example, like social distancing and working from home restrictions of travel and leaving home. Is there a specific difficulty which you painted up South Asian community may face to abide by these measures or to face these measures?

**R:** In my person opinion, no it shouldn’t be. It shouldn’t be any different from others, which of the background you come from it shouldn’t.

**I:** Travel wise or what job you have.

**R:** Yes, of course, it’s going to affect the job situation. But that is, in terms of South Asian community, particularly if I think of the South Asian community, it’s not going to be any different from any other person who is involved who is doing their job. In that sense, no, I don’t think so.

35.09

**I:** That is the same, yes. To what extent do you think is it similar to and those experienced by the white people. Is that coming back to the same question again? Reflecting like is that social distancing, is it the same as South Asian than white people as they see that? The experience social distancing or travelling from home, working from home.

**R:** I would say in my own view, probably the South Asian community is a much more—they are following the rules more. Following the social distancing more than probably their counterparts in one sense. Whether it’s their general psychology or how they work. I am yet to—I don't know exactly why. In my general with what I have seen among my friend’s circle or the immediate social circle that probably the South Asian community is following the social distancing to the book compared to the other counterpart.

**I:** What are the challenges do you think by the South Asian community to access the healthcare system. Can you think of any specific things that why they can or cannot access the healthcare?

**R:** No, I don’t think there is any difference.

**I:** There shouldn’t be any difference in that.

**R:** There shouldn’t be any difference in that. NHS is free for everyone. It doesn’t ask you whether you come from, where you come from. It treats you equally. You can always access NHS. It doesn’t matter which background you are from.

**I:** Do you think people in the community trust the government to make the right choice about the pandemic?

**R:** No, not always. If I have to go back to your previous question whether they can access the health system, yes, they can access the health system. Whether they will do it at the right moment or not that is still, that is doubtful.

**I:** What do you say that they may have any issue with trusting the government?

**R:** If I, again it’s all my own view and my own experience that I grew up outside this country. We came from a different society where things were especially the health system works in a different way. Where so a lot of people still expect NHS to work the same way that on demand, accessing it as immediately and I am not putting it in a correct way. Let’s say the view and incompetence in terms of how they have always perceived how the healthcare system should be, because of their own upbringing outside this country.

**I:** Carry on.

**R:** No, sorry, yeah. In that sense, sometimes it’s difficult for the first generations it’s difficult for them to always rely on the healthcare system or how the system or to understand how the healthcare system works in this country.

40.03

**I:** Do you think that made a difference between that healthcare system which you had before coming into this country and the NHS working here? What are the major differences you can see?

**R:** The major difference that has been first of all where I grew up in India and so the healthcare system over there is, you go see the doctor or if you have any major problems you go to the hospital and you pay the fee and you get your treatment. It’s pretty much instant what is happening, what happens there. Whereas when it comes to NHS you see your doctor and you get your referral and so there is always bit of a waiting time. It doesn’t, in my possible experience, of course, when you have an emergency and it doesn’t matter if you are getting your treatment from NHS also, you will be treated as emergency and you will get the care that’s needed at that point. If your symptoms can be treated in a much better way or with a little bit of time given a proper diagnosis or proper—getting the proper consultation. You wait for that to happen when it comes to NHS. We are not really coming from the Indian background. We haven’t got that wait. That wait in time. It unnerves you more that what is happening or not understanding what is happening and why it is happening. Why you are asked to wait for your treatment and why it’s delayed to get that treatment, that particular treatment.

**I:** One further question, to what extent do you think that people in your community understand the health messaging surrounding Coronavirus, which is very important to understand.

**R:** My understanding, to the majority of the community it should be clear as they follow the news media or the global media. They have the information in their hands. They should be aware of the Coronavirus in the same way as any other person.

**I:** Any other person that they should be.

**R:** Yeah or any other community that should be.

**I:** What do you think could be done to improve these messages like to make them more aware or how the message is going through so that they do really adapt to the orders, social distancing. Actually abiding the hygiene and health. What do you think and how do you think messages can be—

**R:** To some extent it has to happen within the community. I would say different clubs or who run or religious places can inform their people that what has been happening and how they can take the precautions of what they can do when needed or different organisations they who are involved within this community they can educate their members that what can be done. The other sense is, this country has been pretty good when it comes to translated into different languages. We often see the NHS messages in Hindi or Buratti or Bengali. That can be done always to communicate in those languages so that people can understand more that what has been happening.

45.10

**I:** Do you have any difficulty in sending these messages to them or reaching these people by the government and by the bodies wherever they are. They are also trusting them to follow. Can you think of any difficulty which the government will have?

**R:** Difficulty in the sense it’s that like when I initially said that how there is a majority part of the section who are asylum seekers. They are already wary about revealing their status to the rest of the community or other different groups or different organisations. That can actually, rather than—that can prevent them to be open to these ideas, these messages rather or reaching to understand or just reaching out for help or if they are not in touch with the organisations or what has been happening around, they won’t get the message themselves. They have to come forward as well for the organisations to work with them.

**I:** Finally, what do you think has helped you and your community to deal with the crisis? Any specific thing?

**R:** Like in the sense of—

**I:** Perhaps it’s just a plant like believing in something in the community sense or really in a particular way of living, health, diet and so anything which has help our South Asian community to deal with the crisis?

**R:** I don’t think there is anything that can affect it in a different way.

**I:** You see them as the British people and the South Asian in the area.

**R:** Otherwise I meant the health, whether they are diet restrictions or diet, but we all try to live healthier. We all try to be healthier in our life. Sometimes health conditions happen. But that is not always in our own, in our control. But in another way, yes, to some extent you can say that if someone is having diabetes, which is quite, with South Asian people are quite prone to having diabetes. Their eating habits might not help always what they are eating or they are controlling it might not always help to control the diabetes which might trigger when someone is having the COVID symptoms. In another sense, if someone is having a healthy diet or following the general hygiene, it’s nothing different they have to do. It’s just washing hands and maintaining the basic hygiene. If they are following that, it shouldn’t affect them in any different way than any other community.

**I:** We are at the end of our interview. If you have any question you can ask or if you want to mention anything which you think we should have covered or if, in your mind, you want to discuss anything.

**R:** I think we have pretty much covered what I had in mind. I am okay with it, yeah.

**I:** Alright. Thank you so much. It has been lovely to talk to you.

END OF INTERVIEW – 53 mins

Transcribed by Linda Pitt. Email: linda@laptopconfidential.com or laptop.confidential@btinternet.com - Telephone: 01964 612088
