# Supplementary material for: Mitigating the identity and health threat of COVID-19: Perspectives of middle-class South Asians living in the UK
Source: J Health Psychol. 2021 Jun 22;27(9):2147–60. doi: 10.1177/13591053211027626 (PMC9353968; doi:10.1177/13591053211027626)
Supplement: sj-docx-5-hpq-10.1177_13591053211027626 – for Mitigating the identity and health threat of COVID-19: Perspectives of middle-class South Asians living in the UK [file sj-docx-5-hpq-10.1177_13591053211027626.docx]

uk2-groupa-akh-may20

Transcribed by Sharmistha Chaudhuri

Duration: 1 hr 12mins 30 seconds.

Researcher: Thank you again for participating in our study. And, as you know, that today's study, it is looking at your perception of COVID-19 as it is happening now at the moment. So my first question, I will begin with, what do you think is happening to the world?

Participant: Yeah, I think I mean, technically of course, you know, that there's this virus, which is COVID-19, you know, which originated from wildlife in China, in Wuhan, and then it transferred to the to the human beings, from then on, you know, it is transferred across globally. Because it's such a kind of, you know, it's such a disease which can basically transfer from one human being to another. So that's how it is contagious basically. Yes. So that's, that's technical technically, you know how this is happening. So I mean obviously this then has impact in terms of, you know, economic impact, there is a social impact. So individual impact, you know, those are sort of impact we could see, and one of such thing is lock down, you could see in UK. Yeah, yeah.

Researcher: Right. what comes to your mind first, when you think of coronavirus?

Participant: Nature, nature you know, you know, make up our life, you know, and has put us up in this world.

Researcher: That's very, very unique thought! A lot of things, like many things that people are thinking in that manner. So do you think it's only nature or any other factors maybe associated?

Participant: So, so basically, I mean, it's nature, I feel nature actually embed all the factors together because what I always believe is that we are all interconnected. So there's nothing in this, you know, in this universe, which are not connected. And, and to me, you know, when I think of coronavirus, I think those interconnected actions, which the world is taking in different areas in terms of how they kind of proceeding with their way of life and how, how this interconnection is leading to this coronavirus. Coronavirus is now, but there were another viruses before. So inter-connectedness of, of not just human beings, but you know, different components of nature, that, that's what I see.

Researcher: And then how do you think coronavirus has affected the people in general?

Participant: I think I think there are different you know, for example, looking into this, I think people are having, you know, both side of the, you know, context to this virus. So, you know, in one context, of course, you know, that they are in, you know, in, in difficult situation, if they are in a, for some people whose, you know, close relatives, if they are dying they will have the biggest impact. In fact, they are dying in the hospital because the way that is, they can't be there before, you know, it couldn't even there in the funeral side. So there are those contexts which is happening, which are kind of bit, bit harsh. And then the general context to harshness also where, you know, people who are used to that social aspects of going out and, you know, meeting people and conducting their affairs in a more kind of an open way, if you like, it has to subside.

Participant (4:23):So those, like, those are one context, but I think on the other context is also making people realize about, about the value of, of, you know, of, of being basically, you know, in their own words, what that means, you know, the value of gratitude in terms of work, what they have, the, the, the value of, you know, having friends around when they realise that value now in different ways, you know. Lot of innovation is happening because of that, like connecting, connecting with people around and I have heard, that people are becoming more compassion, you know, compassionate than before. So, so, so those are the positives. I think, I think this is playing in both side of peoples' psyche, uh, both positively and you know, developmental wise. Overall, it will play a positive impact going forward, that's my belief.

Researcher: Can you tell, how did you come to know about coronavirus?

Participant: So I think I have, I have been personally, I generally follow news and I'll generally very much used to watching BBC and NDTV and yeah, exactly. Yeah. As a media analyst, I use to understand what's happening. So from that, you know, the whole story, China and all, you know, and then, and from Italy you know, it came to UK, and last few weeks, of course, those Downing Street briefing, and they were kind of, I really watched that as well in terms of being aware of what's happening, but that's now basically, but yeah.

Researcher: So that, that's that basic channel, that news broadcast it to the channels. How about other sources like the social media?

Participant: So the media, you know, I you know, obviously I hear about what you know people are saying, what they're feeling about the virus, both positively and, you know, the, the thing which we discussed, you know, of contagious. I could see more, of you know, people's thoughts, what's coming to their heart about coronavirus in the social media. But in terms of, my reliance over the information, I rely on very selective media and BBC, NHS, all of these to me are that selective media.

Researcher: Right. So for that knowledge, you do not rely on the social media much. It's the news channels what you depend on.

Participant: I read a few them, and then I make my own sense of that. More to do with, you know understanding personal aspects of people around and also, you know, kind of taking the whole context in the picture helps.

Researcher: And from all the knowledge you have gathered, can you tell us what do you know about coronavirus?

Participant (7:35):So, so so coronavirus, basically, you know, again, saying technically, then it is, the wild animals, they basically have, just like us, you know, they have different forms of bacteria and virus, you know, those are inside the body. Now if you, to me, if you, if you are going in their territory, by distracting the biodiversity, distracting, you know, their habitat, and also damaging everything happening, then you are allowing those, you know, those wild animals to come in contact with human beings. And, and this virus, basically that, you know, has the potential to transfer from those wild animals to human beings. So that's how I think it is the source of coronavirus. And then with the nature of the virus is basically said, that, you know, it spreads very quickly. If you are in close proximity with, you know, some people who had the the virus, it can spread very, very quickly from, you know, from your airdrops. If somebody has it, you can very quickly catch the virus and that's why, and therefore you know, social distancing measures, stay at home measures came in.

Researcher: Do you think that any, any other information you have on how biologically it affects the people that, how, what happens within the body?

Participant (9:18):Yeah, really interesting. I had a context to that, and then, you know, I recently had, had a very good chat with one of my doctor friends, who is a pathologist in US. Very productive discussion, and you know, what came out from that is, you know, just like, you know, our body has DNA. The DNA cells. Virus is nothing but a small component DNA, which can make its own living, but when it goes to human beings, what it does is, instead of, you know, human beings, you know, to be able to function with the normal immunity, this virus has the potential to, you know, just done overturn that immunity against human beings. And in the case of coronavirus, you know, it's affecting more like lungs, you know. The lungs, it is taking fluid out of the lungs, you know, so that your breathing can happen normally. And these virus, uh brings the, you know, those fluids back to the lungs and then, you know, starts impacting your way of breathing. And that's why, you know, people suffer. In another interesting context, we discussed, which was the fact that, you know, in the same model of interconnectedness, which I was mentioning, one thing we discussed about was, how, when we think about, you know, microwave, radio wave, all those, you know, physics and quantum, quantum physics, and all those things, how those physics, you know, relate with chemistry and how those relation suffices. There is a strong connection in, on the basis of these three, you know, the critical subject of science and how those three are, you know, generally, you know, they're kind of connected.

Researcher: So, in your opinion, I know, like I have understood, you said that the coronavirus appeared first in the earth or like from the wild animals in that context, do you want to add anything to that in your opinion, like how coronavirus appeared in the planet? I know you mentioned wild animals, you mentioned humans interfering with their habitat.

Participant: Yeah. So the interference is the main thing, which includes the eating habit, In China, there are some people who, you know, has the habit of eating wild animals, as part of delicacy. And those are difficult concerns. So apart from you know, changing the biodiversity and making those wild animals closer to human beings, which is one factor, again, out of all of those factor, you are now making that part of your diet and they are in context, you know, you are trying to bring those animals very close to you. It is very difficult environment for those animals. They are prone to basically, you know, release those viruses inside them because they are kind of, you know, in, in quite in a difficult, stressful situation for those in those environments. And then those viruses, then, you know, comes in sort of, so sort of, the fluids from those animals, any human beings in contact with those fluids has the chances of them getting to human beings. You actually brought them to human proximity, which then started spreading very, very exponentially, which is what happened.

Researcher :And in your opinion, how did it come to UK?

Participant: So, so I, I feel, you know for UK basically, you know, UK is considered as one of the developed country, and I feel, I personally feel one of the, you know, aspects of being developed is, you know, being more interconnected in the social, business, political world. And one of the factor of interconnectedness is, you know, you will have lots, lot more people in your, you know, in your country will be traveling, because, you know, you know lot of interconnection happens through travel. There are also a lot of people who will come to your country because you are going to connected places. So this is the context, where you know, it is the people who travel in those affected countries then came and brought those virus back. And, and this is how I think, you know, started spreading.

Researcher (14:20):Correct. And since the beginning of the pandemic, have your perception about coronavirus changed? Or it has remained the same? I mean, it doesn't matter. What do you do think?

Participant: Yeah. It's, it's quite interesting. Yeah, I think I, I think fundamentally it has not changed yeah. In terms of, in terms of how I think the understanding of what the virus is, how it came, in the beginning, but I think what probably has helped, is, you know, relating this virus in a bit more spiritual context, (laughs) which I'm talking about. So I think, you know, trying to digest what this means for human beings and what this means for actions going forward. I think those fundamentals have developed, you know, with, with, with my own thoughts and, you know, and, and seeing a lot of thoughts, and the thoughts in social media. Obviously, you know, you can have both positive and, you know, some sort of, you know, negative thoughts which can come there. You know, if you're picking up, there are a lot of good positive, you know, aspects on social media, which you could pick up. And then it also has, you know, in your, in your thinking, as long as you're picking up the night and stuff, because social media is full with both kinds of stories.

Researcher: So what do you mean when you say this spiritual context, and how has it changed, like, say, for example, take back, like take yourself back two months before when it was declared just at the beginning of the pandemic. How your spiritual understanding or the context has contributed to the understanding of coronavirus or its effect of?

Participant: Yeah. I think, I think personally I have been inclined towards, a bit more, you know, you know, those existential questions for some time past couple of years. Even before that, those questions around, you know, what it means for us to be here, you know, what is creation, Why we are here and stuff like that. I think where coronavirus has added value in giving me the context, giving me kind of a solid example to basically, you know, to relate those, those, you know, theories to more practical aspects in terms of what's happening now. So I think coronavirus have re-enforced some of those messages that I have been kind of reading or learning from people. I think in those it has changed.

Researcher: Have it helped, for your understanding the world, the situation, has it made any positive contribution?

Participant: Definitely. It has definitely helped in, in changing my context. And also I think if, if your own context changes, it also helps you to have the context of people around. So I think in terms of how I relate myself with my family, with my friends, I think it has an impact on that as well. In that way it has taken a toll.

Researcher: Do you think that these particular pandemic is any different from anything else we had earlier?

Participant (17:58):I think for fundamentally, you know, there has been, you know, the pandemic has not happened for, for, you know, for centuries. I think the last pandemic which happened, to my understanding it had been in 1400, millions of people died. What happened later with Ebola, SARS, MARS were not exactly pandemic. It was more like epidemic or how we call it. So I think, I think from that perspective, this has been huge. And, and I am aware that these things that were happening more in developing countries like Africa and you know, those areas and, and, and they've been able to manage and subside that, you know, quicker than, than now. And in that way the vastness of situation varied- this has been enormous which has to happened now. I think at least for our generation, and possibly few generations to come, this will be the biggest event to have happened. Yeah, because, because this is the biggest pandemic we have seen!

Researcher: Absolutely! At least in our living world. Right. And what do you think about your government's response to the pandemic? How well, or how better they could have handled the situation?

Participant: I, I mean obviously it is difficult to say, but for me personally, I feel that, you know they started they possibly didn't start well, they could have had, you know because they knew what's happening in China and in all other countries, and all these were growing. And there was a time when, you know, in UK we had, you know, a number of people less than 10. So I think they would have been a bit more proactive in, you know, taking this decisions around, you know, those quarantine measures in airport and, you know, people who are coming, you know, and screening them and putting the light measures with testing, like in PPE, in testing, PPE will come later of course. I think it's more to do with testing and, you know, this ramping up testing at that time, having sort of right tracking apps, something which South Korea and, and, you know, some other countries, Singapore, or even, even Germany, you know, in Europe they managed to do well. I think could have, had done a lot better.

But I think going from that period onward once they passed that period, that's pretty good. I would say they have been quite, quite on it. You know, after the initial phase, I think, you know which was, I think what I forgot actually what name they used for that phase. They had this three phase when it's subside and then there's a new phase. The initial phase is where there is problem, but in the later part of that phase, also in the middle phase, where are in, which is okay, that phase was containment. Yeah.

Researcher: Perfect. And how about the government messages, which you hear like the daily briefing and all. How much do you think that has been helpful and communicated to the citizens?

Participant: Okay. I think, I would say it's a very, a very good method, actually, what they're doing. Communicating, so in BBC, which is, I think one of the biggest asset UK has, not many countries has paid license, which is useful, you know, for a channel like BBC. A very big asset UK has, you know, this time, for those daily briefings and also updating people on different measures. So I feel that that's a very good measure they have taken. And, and that, on top of that, you know, in those briefings, they bring multitude of different people, in the cabinet ranking, in different areas of the department. You know, some, sometimes they have brought education. Sometimes there are military people, some defence experts from medical and health sciences, and showing us proper graphs in terms of where it's happening, bringing more transparency into the process. So, so I think it had been very helpful.

Researcher: And what was the information about coronavirus that has surprised you? Anything you can think of?

Participant (23:14):I think one of, one of the thing which people have talked about even now, they are talking that you, you take two metres of distance. But no, but there are a lot of media reports, which says that, you know, the virus can go up to six meters. And it's, for me to believe that, you know, government and those officials, they're not aware of these measures and, and, you know so, so what if it surprise me is, you know, that those measures, you know, how, how you basically, how the, WHO, also knew the six metre thing, but still the WHO went on for quite long suggesting not to wear a mask. There could be a technical reason that, okay, they wanted the masks more for the frontline staffs like NHS, But I know that there had been gaps in terms of taking, you know, more proactive measures in, you know, making masks or even asking you, and, you know, households to create their own masks, but not suggesting to have the masks, and there has been more evidence within that, I think is, quite surprising to me.

Researcher (24:50):So you think that if people would have worn mask, it would have been more preventive in that sense.

Participant: So so I think masks, obviously it also says how to use the masks, yeah. Your masks, how you use it, it is only effective with certain ways. I agree with, sort of, you know instructions which came. But I think if people had, have had the mask, I think if there are people who are already you know, with COVID-19, mask for those people, it could have reduced. That is one factor and the second factor, I think wearing masks could have both the impacts. It could have had the impact of people, you know, making, taking things a bit more lightly, but it would also have had the positive impact on psyche that way. You know, you must wear mask, and this is serious.

Researcher: You are taking it more seriously. So now the question, when you discuss coronavirus with other people like with friends, families, what do you discuss most?

Participant: I think the top, the family you know, friends and family, I have basically, we discuss largely things around what we are doing, to remain fit and healthy, how could we develop our immunity at this stage before, you know, you know, before the virus hits our own house at some point maybe! You know, a little bit of those immunity. And also I think generally, you know, just, you know, getting yourself fit mentally, also, you know, helping each other and, you know, just, just being there, talking and just being engaged. Yeah.

Researcher: And how your personal life has been affected by the pandemic? It's a question everybody's asking right?

Participant: Yeah. So personally, you know, I I'm basically business consultant. So work wise, you know I was with one client for most like 14 months and I was looking forward to, you know, getting on with my client, getting on with some more good work and, and was a bit wrong timing because I left that client end of March. And that, by that time, you know, things didn't pick up in UK. So I was, I was not sure that if something like that would happen, I was not for that kind of expecting, you know, and then suddenly things kicks in and then, you know, that there are not many projects coming in. And so, you know, then the company has decided, you know, so they decided, okay, let's, let's put, you know, because it's government measure, furlough, right. You know which can compensate the company really to carry on.

Participant (27:58):So, so I think for me it's more to do with, you know, not, not being behind some good projects, which I think I could have been in this coronavirus time. There are a lot of good projects, which could have enhanced my experience with being the NHS projects, you know, and dealing with corona virus funded projects at site. I personally feel that I'm missing out on being in a good role, given my.., because I could have played an important role in, you know, helping some companies dealing with this coronavirus situation. But given that in our company has to take that call and there is some rule book, the company has to follow in furlough. You can't put people in furlough and still get people, you know, asking for work: there are some parameters you need to manage. So even the parameters, even though I could have possibly got back to work, I can't because I need to follow some measures. So basically now I have started thinking, okay, if not that, then what else? I've started engaging with some training.

Participant: And also seeing if I can do some voluntary work maybe, but I think that that's something you may have. It's good that I could have done some, you know, some good work, but I feel that it's not me, it's, you know, a lot of people and, and, you know, you have to take things from perspective that's work-wise. Yeah. And then, and then on the personal level, I think is giving me a more time, to be honest, to reflect and write. So it's giving me a bit more time to, you know, read more stuff from my books, internet and all, reflect more on my own life, you know, and getting a bit more focused that way. Me, doing bit more physical activity. I think physical activity has grown than before. Being aware, and eating the right food. I think, you know, I am more conscious of what I eat, I was giving time to my, family life, like in terms of my kid and my wife, I think obviously because we are all together, you have to give you time, you don't have any option anyway! (laughs).

Researcher: So though you have already mentioned, but just to reiterate, what are the positive effects of this pandemic in your personal life? Do you see anything?

Participant: Yeah, I definitely see, as I said, I need to start with a question of making, giving me more clarity on the fundamental of our existence as human beings. I think it's the biggest clarity, you know, you get, and those things, when you see what's happening, people are dying and you know, animals coming back to the streets and you know, wild life coming back to streets. We have a family member, who lives in Rishikesh in India, where you know, river Ganga flows. He says, he has not seen river Ganga as clean as this at any time in his life! Now, flamingos, you know, almost three times flamingos are coming in Mumbai, another city in India, you can see similar stories elsewhere. So all of these are input to my brain to bring that context, which I was talking about, what should be giving more clarity in terms of the life ahead. This is quite positive. And then on top of that, I think it's giving me time to, you know, focus on my health, my physical aspects, uh ng exercising, diet food..I am also doing different activities, which I have not done before, so I create a lot of new exercise, you know, template, with which I am working with my daughter now. Which I have not done before. And it was quite exciting to do those things. So those simple aspects of life, you know, which we didn't know before, so we are doing that now.

Researcher: And can you tell..though you have mentioned some, that, what is your daily life during this pandemic is like?

Participant (32:40):No, that's interesting question (laughs).So so I, I think I, I get up not early. I get up late. I think I get up like about 7, 7: 30 in the morning. I would have, I would love to get up at five or six that that's another thing, which is part of my plan. But at the moment I get up like, you know, seven, seven 30. I think I take like seven, eight hours of the sleep taking. So I sleep usually at 11, 11:30 in the night. And then I have, I have, I start my, day, you know, it is a weekday or weekend..maybe I speak about weekday. On weekday, basically I have, I have created a kind of a, you know, mini instruction for me in my white whiteboard in my office. And one of the best instruction is, you know, no social media, no phone between this time, (laughs) giving me focus, that this time is for my, you know, my activity.

Researcher: Are you maintaining that? (laughs)

Participant (33:58):So I was not able to follow before, and that's why I created this. And now after writing down on the board, I'm able to follow it because it's just right next to my eye, written in red marker, and it includes basically two main things. One is, you know, are you mindful? That keeps me reminding whenever my thoughts go in the area which are not productive, bringing that back. And then, you know, no social media, no phone, you know, between certain times I'm not restricting myself, I'm just restricting myself at certain times, when I want to do something else. And because my spiritual inclination grew at this time, I also included no spirituality topic (laughs).

Participant: Yeah. So that's something I'm doing. Say in the morning, I'm basically doing a bit of training. So at this time I tried to do a training, so it's not just training. I, so what I, what, at this time I am focused on my training. I am focused on anything to do with my career/professional development, any articles related to that, knowing what's going on with colleagues and stuff like that. So just for me to not get, you know, distracted, okay. This is my time of my, you know, my work, let's put it that way, that way. And then, and then in the morning I take a very light breakfast, which is more like fruits, and smoothie and stuff like that. I have decided not to take caffeine more than two times a day.

Participant: I tried to take green tea to start with and only take caffeine in the afternoon. And then I get back to my work. And then in the afternoon, in the lunch time, then, you know, I take a break and that includes my social media break as well. My lunch and everything. Then I get back to, you know, again, get back to this work stuff. And then after 5:30, I've allowed myself or, you know, having physical activity. I do one hour at least you know, I do that with my family.

Researcher: Do you think, how do you think this is any different from any time before when you had been working from home?

Participant: So that's a very interesting question. Yeah. So I think when I was working from home before firstly, no, I didn't have the whole family in the house, most of the times. So now, now one of the problems is (laughs) you know, if my daughter stays in the house and her school is going on and she's home learning, you know, she, you know, half of time, she will need a bit of support in, in, you know, in, in out assignments or what's going on. So, how you then carry one your own focus and still, you know, being able to support her, you know, that's, that's one way. Secondly, you have three people in the house, so how are you basically, you know, you know, your main focus. But there'll be distractions in some other ways. They'll be expectations. And I think, there are positives as well. There is a lot more, you know, than before, I see now, I think, because, there are certain activities my wife is doing, her health and all activities. They also kind of motivates me. So, you know to discipline my food and, you know, with my timings and health, both sides actually.

Researcher: Okay. So we are near to the end of the part one of the interview. Before that, how do you think, this pandemic will end?

Participant: I think I think this pandemic will end..will not entirely end. And in the sense that, you know, for the next 12-18 months, there is a possibility of vaccines coming in by then. And I think it will you know, it will gradually, you know things when it starts subsiding, not suddenly, with those testing measures, checking apps, you know, all those things. Also, social distancing measures, in slightly more innovative way. Each sector has to follow it in their own smart way of maintaining social distancing but still carrying on their business. There'll be a balance between health and health measures with economic measures. And that, that will lead to a very gradual process of subsiding you know, with some sort of some we're vaccine and all coming into 12-18 months’ time. But I think I personally feel even after 18 months it will not completely go away. I think with the nature of the virus, I feel it's, it's going to stay in the world. But we, we, we need to see whether we can carry on without wearing face mask or not. That's something to be seen.

Researcher (39:35):Yes, indeed. And what do you think would be able to prevent us from these future pandemic like this?

Participant: I think there, there will be you, you need number one, you need a lot more collaboration globally because it's not a story of one country or one part of when, you know, ethnicity, it's a country, the is the story of the world. But the way the socioeconomic, political dimension has changed recently with more national regions coming up, coming up, you know, in different countries I think that's not going to help, You need to bring in a lot of fundamental collaborative measures when it comes to solve such a global sort of problems. That is one thing; secondly, I think a lot more investment has to go in and, and you can see the example, you know, at this time when you needed more investment for the virus, there is a president, you know, somewhere there, we spent putting down the investment. For the future pandemic, actually investment has to increase.

So if you are G-20 nations, who are, who has more enormous power, you know, put in more as compared to other, but everybody has to put in, you know. And thirdly, I think a lot more awareness has to come in people's behaviour in terms of how they relate their life and how they relate that life with interconnected with the nature. So those are three aspects, I think, so more collaboration, globally, huge investment in the right way, and then more awareness of human behaviour and its connectedness with the nature.

**Part II**

Researcher (41:35):

Thanks. So this is the end of part one of the interview, which we discussed about how you see corona virus, which has been very helpful. You have given a rich reflection on many, many kinds, like, especially with the spirituality, which have been helpful. Now in part 2, which is the section two of the interview is focused particularly on your ethnic community, which is South Asian. This is focused on your community and how the understanding of coronavirus has affected the South Asian people. So I will start with asking you, what do you think are some of the health concern for the people in your community during the pandemic and why?

Participant: To start with, you know, I will just be clear on one thing, when, when I say South Asian community, my perspective of that two: one is the first generation South Asian community based in UK. Because, because, you know, we, we have not been in touch very closely, you know, community of South Asian who has been here for decades. So that's one context to what, what I'll be speaking about. And second context is because we have come here and first generation, we have not come here for decades here. We have come here more recently. Like, you know, our context of understanding also relates with how we feel the impact of that happening in those South Asian countries like India, Pakistan, Bangladesh, in terms our friends and family. There can be a joint context of, what I bring in here. When I say something, it will be two contexts together.

Now, from this context, I feel, you know, people here, health wise, luckily we have, where I deal with the people here, you know, I have a lot of few people in the group who are very prone to, you know, taking care of good health. So, so we actually have, for example, created a health group for our own close community.

Researcher (4:33):When you mean people, you mean people from South Asian community?

Participant: Yeah. But I'm talking about some close family friends, or the bubble, with whom we talk about, with whom we talk and mix lot more than other people. But in that bubble, you know, there are quite a lot of people who are very much conscious about their health. For example, we also have an NHS doctor in that group. You have lot of people who, who have their own gym in the house. For example, we have, my own wife, who will basically exercise regularly.

Participant: I think for, so luckily, you know, we have those sort of people. So we have created a group where we kind of, you know, create different sort of challenge and create each other to, you know, I support each other in some form of health activity each day. Which is keeping us, you know, fit. So that's, that's one thing, you know, and secondly, I think food wise also, we if you see the South Asian community, traditionally they have, they have like eating a lot of, you know, food with spices and you know food which needs a bit more cooking and stuff like that. Now, I have seen that it happening even in my own house, you know, I'm talking about you know, before. But you know, over the last year or so, you know, things have improved in terms of how we eat the food also. During coronavirus, I could see people are eating quite healthy. So seeing that was a time to take measures, which can increase the immunity. Now, people, you know, they would take you know what could increase their immunity going forward so that they can be protected, if the coronavirus, you know, come, come in there, you know? So, am I giving you the right context?

Researcher: Yes, yes! There is nothing right or wrong. It's just tapping into your own understanding. So anything is, anything can be important. So now how about do you think your people in the community are more or less vulnerable to the pandemic?

Participant: Okay. More or less? I think I think age wise they are less vulnerable. At least in UK, because as I said, we have a first generation, lot more are professionals or, you know, in the middle age group. So age wise, you know, that they will have less impact I feel, and of course, you know, there are cases where things can go other way around as well, but back in India, where, again, in another context where I have my parents, my community as well, obviously for them in India, you know, they are trying to cut down you know, some lockdown measures they're taking. But in India, you know, I have, I have my own doubts if it starts, you know, increasing, you know, I feel, you know they will be, there is no similar, you know help, healthcare department like NHS, that you have here, those people, for them to survive, you know, I think it would be tricky. They are not that they're not as known to, you know, taking the right measures in terms of proper exercise, eating the right food. I think that awareness is still not as much as we have here, I feel.

Participant: Correct. So now how do you think the South Asian community living in UK has been affected by the coronavirus? Is it any different from the British people living here.

Participant: I feel, fundamentally, it is the similarity, because you know, the measures which the UK government is taking in terms of loock down, what that means for your work, what that means for your, you know, your boundaries in terms of your personal and then professional boundaries, you know. Fundamentally, I think it is the same across. But on the other hand, I feel, you know, the people about who I hear, you know, of course, you know, they, they don't have their own close community in UK, as the people who has been born in brought up here would have, would have had in terms of, you know, friends from their university, college, school, or even their, you know, family members, you know, they would have more family members around. Even though they're not meeting them, I think, you know, there’s a perspective for that. There could be a support network they might be having, which we don't have.

Participant (51:34):So so we still rely on, luckily we have this good, good bubble of close friends, but I think in our network, as far as I'm aware, not many people have such a good bubble of, you know, good friends around. And some of the people might be new in UK who might have just come, two-three years back. We are here for 15 years, we know a lot more people. So I think, I think, you know, on a personal level, they could be different in terms of the support mental support you know. Physical support as well in terms of, you know, if you need to, basically, if you get coronavirus, you know, if you need some support in the form of somebody to deliver food for you or, or do certain things, I think that there could be technically some support, but I think mentally, you know, I think there will be lot more people in our, our community who might not feel like that who, who might be new in UK. Luckily again, again, again, you know, you know, in our bubble, we're close families, but if I see the entire community, you know, we are, you know, if I notice, things could be different.

Participant: How about health wise or their attitude to health, maybe any, any you, can you see any difference which can affect them more or less than white British people?

Researcher: I feel that that helped too. I think the possibly the British people I feel has learned the art of, you know, keeping, keeping good health from childhood. It was always part of the curriculum and everything. People in the West conference on the, she, I think they have learned that over time, some people might have had that, but other people have learned that. So I feel that will have some impact depending on who I, in, what part of journey about, you know, their understanding on health, nutrition, exercise, mental health, you know, all those things. So, so that way it can be different than be defended. Yeah.

Participant: Right. What ways do you think anything, any difference you see

Researcher: Yeah. In this vein, that's what I'm understanding that are, you know, how could they tell if you had those sort of understanding and also the [inaudible] understanding in your day to day life? You know my B has been my B in their development to part for that. As I sit in my bubble, of course, people are not going to let you, but I could see more people that aren't, you know, who might not be there yet. And people who are more essentially have come here, you know, might be, there might be stress actually also you know, unlike in UK where, you know, people, I used to work in a slightly different mindset in India, you know, people see work as more like a, you know, as a holy grail sort of thing, in terms of, you know, if you have work, you are safe, you have money coming in, you know, stuff like that. And if you, if you're out of work or, you know, if you're in that situation, that's quite for people from Asia and they have some limitations also, you know. The people in South Asian community, they have come here…and most of them are only from very specific sectors- that people would be from IT sector, because we know other sector people would be quite less, you know, to be honest. So, uh, their difficulty in managing, you know, uh, their aspects of the work could be very different how coronavirus has impacted IT versus other sectors could, could, could be, you know, could be an aspect to think about.

Researcher ([56:10](https://www.temi.com/editor/t/ZoNsBCT_YaCcnwtbMRdJJVzN44icvm3e7dpJnci1QBMocu9H8IC78TGqJl2ZzHoIHZLLBzeUm7WMdaJddflfUKkydoY?loadFrom=SharedLink&ts=850.53)): Definitely, different things can be coming up! So, um, how do you think, like the government has introduced so many measures, right? Like social distancing, working from home, restriction on travel, leaving home, et cetera. So are there any specific difficulties you can think the South Asian community faces in relation to abiding these measures?

Participant: Yeah. That's an interesting question. Because I think generally I have the feeling that, you know, uh, people from South Asian community, they are much more sociable. uh, their context of life and they, they are, you know, uh, they're they're they want to be with people. They are in, you know, with people, they like to be people, their context of life are less individual and more social space, uh, as compared to, you know, the people in the Western world. I think it's not just India might be the case for most of Asian culture. Um, for, from those contexts we see here, I think, you know, people are not used to what UK government have asked them to do. And, uh, I, I don't know how people are taking that. When I was walking I was, uh, you know, outside in one of the local residents, you know, there were some Asians, playing Volleyball! There were like 9-10 people, which was not the UK government asked, you know? And even, even within our bubble and area around, I have seen that people are prone to asking, you know, finding ways to do social based exercise or something like that. But luckily, there are other people, who can manage those inclinations, and if we could, you know, collectively manage. But, uh, I think it's, it's not that easy for people from Asian culture to not meet collectively with other people, uh, for food or for, for talk, or for general, you know, chit-chat. Uh, in fact, we are not used to, you know, not speak with your neighbours, as we see here- where we live is a slightly different world.

Researcher: Interesting point. Now, to what extent do you feel health wise, like accessing the healthcare facilities during this crisis, what are the challenges faced by the South Asian community? Do you see any?

Participant: So I think, NHS wise, how you access NHS, that's universal I think. I think that that's something same with everybody. I would imagine. There is a very specific rule in terms of what to do. And, you know, in terms, in terms of isolation, in terms of when to call 111? So I guess, uh, from a, from NHS perspective, there should not be, you know, an issue health wise. Um, if there's any other factor, uh, which is, outside NHS, in terms of, you know, uh, your community base, understanding of health, or something like that is there, then of course, that's where South Asian community might be missing out. If there are some gender groups, groups for community based health, learning, South Asian people might not be aware of that.

Researcher: So otherwise you don't think that they may have any extra challenge or, uh, to access the healthcare?

Participant: I think it should be okay for everyone. That's a good part of NHS, you know, that's how I feel fundamentally NHS is kind of equal and that's what coronavirus is. Coronavirus is equal for everyone, that's how it should be.

Researcher: That's, that's good to know. And do you think people in your community trust the government to make the right choice about the pandemic?

Participant: Yeah. Before I go to this question, I just want to address the last question, once more. There are reports that recently have popped up where it says that the number of deaths in coronavirus has been a lot higher in deprived areas, versus in less deprived areas. And, uh, so, so, so I, I feel that's something we should also be, uh, you know, finding out- I don't know how, but, you know, I think the South Asian community, most of them maybe living in a balanced areas. I wouldn't say they, you know, they would be living in a very deprived areas or they would be living in very effluent areas. But I think a large community people are not part of the affluent group, for the first generation. And, and, and, and because most of the people who, came here are a bit more professionally educated, they will also not be in very deprived areas. They are more on a middle ground areas, in fact, my own area, where I live, I think it would be something around that sort of balanced area. So that's something also to be seen how being in those sort of areas also have might've impacted in terms of, you know, getting the coronavirus. You know, more than, you know, other areas. And if affluent people don't have to do some certain work and go out, you know..

Researcher: What do you mean by certain works?

Participant: So, so I mean, uh, like, you know, luckily, you know, many of the South Asian people here, which I said, ah, come from IT department, right. So the IT people still can work from home. Yeah. But, you know, if there are people who are, you know, non IT, and, but there's of course a certain percentage of them as well- non-IT. Uh, so, so they, they, they then go to work and, you know, uh, so, so, so the percentage of those sort of people who, uh, who are, who can't follow the measures of government in terms of, you know, staying at home and stuff like that, you know, is a bit higher here, compared to affluent areas. That the way the South Asian community, will also have an impact.

Researcher: So they can have the virus more in that manner?

Participant: They can have the virus more. uh, But, but it has, but it has to be seen in, in comparison to their, you know, professional profile at work. So at the same time, a lot more, as I say, Asian people are lot more from IT background who might end up, you know, working from home or staying at home, but all of them are not like that. Yeah.

Researcher: And, and do you think people in your community trust the government to make the right choice about the pandemic?

Participant: Yeah, I think, I think, uh, community, uh, do trust, uh, I mean, uh, I feel, uh, uh, with the wave you were in, you know, two, three years back with the Brexit situation where, you know, uh, parliament was shut down, everything was shut down. I think with the current stability of the government and the government taking you know, more measures in terms of updating people, watch what they are doing, bringing more transparency, Downing street, for example, you know,.uh, um, uh, I, I think those are giving people more reassurance in terms of, you know, they are taking measures for them.

Researcher: Correct. And finally, um, how do, what extent do you think people in the community understand the health messages surrounding coronavirus?

Participant: Yeah, That's an interesting question. I feel in my bubble at least have seen a most of the time, if not all, people have understood and followed them in terms of you know, social distancing and staying at home, you know. NHS has seen people for example, going out, you know, on Thursday evenings for those NHS claps as community as well. Community feeling is there, you know, and that has also made them be part of an extended community in UK, which was great, particularly because, you know, there needs to be some measures to mix things up as a community. So I feel that the general understanding is there at least, at least in some of the people have come across, you know, the understanding is there. Um, yeah. And then of course there will be some exceptions, but exceptions can also be in, you know, outside.

Researcher: Yeah. So can you think of any ways how the messages can be improved, how they have reached the community?

Participant: This is, uh, this is interesting because I have seen people from some of the other culture, you know, uh, who, who are not used to English language and stuff like that, you know, they are getting impacted with the language and they have asked for certain regional based messages. But I feel, for South Asian community, the, the need of those messaging are far less because, because as I said, most of the people in the first generation have good English, they have an understanding of English and they're generally in a good profile- in terms of their, you know, awareness and knowledge and work profile and everything. So I don't think messaging wise there's much needs to be done, you know, from the South Asian community wise, I feel. But, on the other hand, I would say, um, there are some community who have, who have come here, who are not educated sort of, you know, and there are community, as I said, I have been always speaking about South Asian or first generation, but I know a lot of community, you know, here, uh, obviously I'm not in day to day, you know, in touch with them, but they are here, you know, it was 30, 40 years back in this country. They have still not learnt properly, you know, the right sort of culture, the context in terms of language and you know, of, uh, those sorts of aspects of, you know, uh, uh, ways of making them aware of what's going. So I think for those community and those areas, there are some specific areas where those people live, I think for those people, those areas, there should be some targeted messaging. And in fact, good areas, uh, have more chances of spreading, because those areas are bit more deprived side of areas.

Researcher: Got it. And we have our final question, but it's, what do you think has happened, um, to help you and your community to deal with the crisis? Any particular aspect you see how South Asian community has been able to deal with this crisis?

Participant: So I think coming out of the crisis is a collective thing which is beyond the community, I think, I think as I say, it's a global problem. So, so, you know, we can all collectively only come out of the crisis, but, but in the question, how the community can manage this time. Then, then I would say, you know, it's, it's about being more connected with each other so that you can help each other in terms of, you know, availing to certain services. For example, we are connected to different WhatsApp group that has helped us to know where you can order the food, uh, like some, you know, good vegetables and fruits at a right time, you know. So those small measures, you know, is going to have, uh, in, in get getting the right support. It could be mental support also, you know, where you might be in a difficult situation, and might want somebody to speak with, uh, I think, I think, you know, just, just being part of different community, work or social media, that's where I think social media plays an important role, and at the same time it's also equally important, for the community to understand that they are generally, you know, uh, come from the context of social aspect. So this is also the time to reflect. This is also that time to go within themselves and, and see that space and, and get out, get out of this, you know, situation, uh, you know, mentally. So as I say, I just said in one of the messages to one of my friends that, you know, uh, which was my own speech, and that was my own line, which was, I think, I think my own lines sometimes (laughs). That line was no, uh, 'sufferings, uh, sufferings, in this world will never end, but sufferings of your mind can end and should end.'

Researcher (70:30):Very, very, deep thoughts!. Yes. I'll try to come out of my middle sufferings, I think! So, not coming to that. Do you think your community has any particular, like aspects like faith, religion, or spirituality, as you have mentioned has any role on helping them to challenge their crisis?

Participant: I think, I think that's a very good question you asking? Um, because I, I never thought of that, but if now, if I, you know, if I now try to reflect, I strongly feel they, they, this, this is playing a huge role because, because of the way that culture in India is, people, right from childhood, they have been born and they've been developed with some contextual spirituality in some form. Each family has that context. Of course, that context differs based on where you are born, which part of India and how would they see spirituality, but the spirituality and faith, adds some context to that. I think in many contexts is imbibed in people who have come from there. So, so I feel definitely that it plays a huge role in their mental psyche, which might be playing a part for them to continue sustaining. Yeah.

Researcher: Yes. Thanks. So it was great to talk to you again and thank you very much. Uh, do you have anything, any questions, or do you want to add anything because we are coming to the end of the interview. Anything you think that you can add or you thought of?

Participant: No, I think it was a great session. A lot of good, those two sections are good questions there. Uh, made me reflect and think, uh, some of the things I might have not thought, just like the last question, you know, which you have just put. So it is a great work, and hopefully, many of such interviews will give some fundamental, you know, answers to some of the questions we are looking for. So all the best to you and you for that. And thank you for giving me this opportunity.

Researcher: You are more than welcome! It is my pleasure. Thank you.

Participant (72:30):You welcome. It's my pleasure. Thank you.
