# Supplementary material for: Mitigating the identity and health threat of COVID-19: Perspectives of middle-class South Asians living in the UK
Source: J Health Psychol. 2021 Jun 22;27(9):2147–60. doi: 10.1177/13591053211027626 (PMC9353968; doi:10.1177/13591053211027626)
Supplement: sj-docx-6-hpq-10.1177_13591053211027626 – for Mitigating the identity and health threat of COVID-19: Perspectives of middle-class South Asians living in the UK [file sj-docx-6-hpq-10.1177_13591053211027626.docx]

**UK3-groupA-NDL-may20**

69 minutes

Transcription by: Sharmistha Chaudhuri

**Part-1**

Researcher: Thanks you for participating in my study. So I will start with the first question, which is in the part one of the study, as I explained, that what do you think is happening to the world?

Participant: Transformation in one word, if I meant put it that way.

Researcher: Absolutely! Brilliant.

Participant: I think there's a lot of transformation, which comes from, there is a lot realization along with it. I mean, I could go on and on forever, but if I got to summarize what I think is happening, lifestyle changes, changes in attitude, changes in behaviour, changes in, uh, probably, you know, all of this is going to culminate in a very, very different lifestyle, which I see was heading to us. So there is, there was transformation and surely I think that is going to result, I don't know in what, but, um, we could feel it all around us. And, um, I mean, you know, this is in every sphere of life. Uh, I mean, I, as a, as a sort of designer and planner, I look at it very closely from, in the property sector, but, uh, it's, it's having an impact basically on human, human lifestyle. And that is very critical, I think, and the way we think, the way we behave and the way we live, our lives, day to day is going to, is going to change.

Researcher: So do you see as any, any like positive, negative effect or what, how do you sum up?

Participant ([01:58](https://www.temi.com/editor/t/xceXUqAXIvWqkMncqb4W9XJjY9k894aAOl-lGSqsGkld5G7qzHix9MxnTWR-6PZ5MW2FpWIMWfoydgL3cPkohpeXBzE?loadFrom=SharedLink&ts=118.43)):I think it's both, it's both. I think, uh, temporarily, because of the situation and the circumstances, there is a lot of negativity around, but I see the positiveness of it primarily as a family. I think I've got some good perspectives. As a professional perspective as well as a family person. I think as a, as a professional, I'd say there was a lot of change, uh, professionally deep in us, especially as I said, that I come from the property world, it's going to change the way we deliver our housing, the way we deliver our cities, the way we deliver our towns is going to be so different. From what we talked about earlier, the focus, the focus is as I see it, away from our dependency say, I will give you one example: everybody's now starting to talk about a moving focus from the dependency on public transport.

Researcher: Right.

Participant: We were trying to get in as many trains and as many buses. Increasing public transport. Everybody was focusing on how many of you are going to get into town? How many people got jobs, employment, da, da, da.. and it was concentration, concentration, concentration. And there was one set pattern that everybody has been living with for years and years and years. Now, everybody's thinking about it. We've been working at home for the last six weeks, and we are still functioning efficiently. We are having some teething problems-that's because the system has to cope with it. In terms of change in mentality, or people, ok, we've taken the office into our home, and I have been delivering and I have been working. Primarily, my sector hasn't stopped. In fact, it's picked up big, because people now have got more time, to be able to work on things they weren't in a position to do well, years and years. And they're thinking afresh. They are thinking of new towns, for instance, which we call off grid, of really high sustainability, generating their own energy, for instance, less dependency on social infrastructure, less dependency on transport. Are you going to be, can every child be going to be able to cycle to school, or walk to school?

Participant ([04:33](https://www.temi.com/editor/t/xceXUqAXIvWqkMncqb4W9XJjY9k894aAOl-lGSqsGkld5G7qzHix9MxnTWR-6PZ5MW2FpWIMWfoydgL3cPkohpeXBzE?loadFrom=SharedLink&ts=273.52)):For example, in London. you can't even think of, although you talk about sustainability. So this is from a professional perspective. And I think the very fact that we see world over the amount of reduction in carbon, just because of the less trips for instance, people are making. Take my own husband- You know, my, my, my family. He had to fly for a meeting to Ivory Coast. Why do you have to fly for a for meeting to Ivory Coast? The mentality was, you need to fly for meetings, as vice presidents, you need to fly to a meeting. You need to roll it out that red carpet for the president, you have to give in. That's not sustainable. Over the last year, I think he's done about, I think an average of two trips a month. about 48 trips a year! What are you talking about?

Participant ([05:36](https://www.temi.com/editor/t/xceXUqAXIvWqkMncqb4W9XJjY9k894aAOl-lGSqsGkld5G7qzHix9MxnTWR-6PZ5MW2FpWIMWfoydgL3cPkohpeXBzE?loadFrom=SharedLink&ts=336.4)): So, I think, I think the whole world, you know the carbon, uh, you know, the, the, the, the issue of carbon is going to be, the global warming is anyways a big issue, but how we as human beings changed out behaviour has a direct impact on the environment, it is phenomenal. We are living in a more sustainable life, daily environment. Definitely. So it affects the professional work, sort of personal lifestyle changes. It Impacts. It impacts us as a family. We're having to do spend a lot of time at home. It's, it's great. It's positive. Um, I get to see my son more, which I wouldn't, as a working mother, not being home all the time. However, obviously there is a downside, is that having to home-school, we are the teachers for him. So, you know, it's got, it's got the good, and the bad as I say, but so far it's working out, but I think in the long term, people have to think, because if an eight year old can pick up and pick up a video, understand what it means to do fractions, decimals, and understand, it is fantastic. Whoever thought, an eight year old, having looking at a video and do fractions, or the decimals, but he's doing it. The other day, he shocked me! Very simple thing- let me give you an example. And he was sharing his screen in the cloud with his classmates. And I said to him, I said, where did you learn? where did you learn how to share a screen and to do whiteboard markings? He said, I see you marking at all times- I see you sharing with your colleagues!

Participant ([07:23](https://www.temi.com/editor/t/xceXUqAXIvWqkMncqb4W9XJjY9k894aAOl-lGSqsGkld5G7qzHix9MxnTWR-6PZ5MW2FpWIMWfoydgL3cPkohpeXBzE?loadFrom=SharedLink&ts=443.07)): You share it with your team on Monday mornings! Yeah, it's probably watching me- But the point is, there is so much of sharing, sharing that he's picking up on it. He probably didn't know what his father and mother did. I mean, he knew that his father worked in Africa. But probably didn't know what I actually did for my life. The very fact that we all three, working together, flat out, Monday to Friday, there is cross learning, there is lot of sharing of knowledge. I think that at lunchtime, the other day, he came up with the idea of saying, let me talk about what projects we work. I told myself, what project you are doing? He said, you think I don’t have a project? But I think the only problem is at our family level, obviously your social status, has literally reduced to zero. You don't see anybody, anybody on the street. So you're, I mean, I personally benefit a lot, a lot on being in a room with professionals, was really, again, you know, mixing my professional life and personal life. But I love to go to work. Not because not because I want to share my household responsibilities, but because there's a lot of knowledge sharing and knowledge gain I get from being in that office there just, just for six or seven hours of the day. And I miss out on that. I'm just going with my colleagues for a drink of coffee in the afternoon, but that's all the downsides. Yeah. But I think been looking at it so far so far, very positive angle. I should say, I should say it's helping us, that is all I can say. I do have a new perspective. And I think, I think maybe is that positivity that is helping us to just sort of keep it going. And that pays. Maybe there will be a crash someday, but we think that they're getting on each other's nerves. Which we are totally on!

I can't keep myself out of each other's notes on a Friday afternoon when you're telling each other.. Don't look at each other's faces. I have a study room. I have a dining table, so it is lovely. So what do you mean? The thing is, we are going to have crossovers. I don't think anybody can say that they, they're not facing any difficulty. People talking to different parts of the world. People talking in different languages - three languages at work time- and all those sorts of things up with negative. And if I think this is there, we, as designers and as architects are starting to think about lifestyles, being the, sort of the seed of how we design going forward, simply because, you know, if you're going to say, Oh, well, I'm going to spend 50% or 60% of my working professional life or life in the house moving forward. And I don't have to be in the office all day. Do I need bigger spaces? What happens to people who live in zone one and cannot afford a bigger space. You see? So now it is that kind of debate that is going on in our heads and in all the professionals, what is this changing life style? How are we going to cope with it?

In a way our life style are teaching us. What is going to come out of? It is probably not a solution instantly. You shouldn't even get tested over the next five, 10 years, but it is going to be a very changing scenario.

Researcher ([11:31](https://www.temi.com/editor/t/xceXUqAXIvWqkMncqb4W9XJjY9k894aAOl-lGSqsGkld5G7qzHix9MxnTWR-6PZ5MW2FpWIMWfoydgL3cPkohpeXBzE?loadFrom=SharedLink&ts=691.78)): So now if I ask you that, what comes to your mind when you think of coronavirus? Just like that.

Participant ([11:42](https://www.temi.com/editor/t/xceXUqAXIvWqkMncqb4W9XJjY9k894aAOl-lGSqsGkld5G7qzHix9MxnTWR-6PZ5MW2FpWIMWfoydgL3cPkohpeXBzE?loadFrom=SharedLink&ts=702.41)): To be honest with you, I'm amazed by how, what we took for granted in terms of saying, because we always talk about how, um, fit and healthy life was. We took life for granted. It was a smaller issue, which we, you know, we probably all got viruses in our life, at some point in time. We've taken all sorts of, um, inoculations to combat, uh, you know, yellow fever and BCG and all that, but they don't realize the impact of any of that. I forgot. I lived in Africa, I'd have to take yellow fever. I never realized, you know, I live in a regional, where yellow fever would happen. And then you see this one little thing in the world that has created complete chaos. Oh, goodness. You know, that is coronavirus. That is one virus which killed millions of people? I don't know how many I've needed to talk..about 80 workstations trying to crack this whole sort of thing, business. You know it has turned the world upside down. And for me it is a huge level of uncertainty in terms of health, in terms of, um, you know, what we would normally take for granted in terms of our sort of, um, um, fitness or in terms of saying, Oh, I'm fairly fine. Nothing is going to affect me. Well, you know, for me, coronavirus is, unknown.

Researcher: Unknown item that we don't know yet.

Participant: It is unknown. Because I don't know. I don't know what it is. perhaps until I get it, I will not know what it is!

Researcher: I mean, God bless you that you don't, but yes, but that's what it is. How do you think coronavirus has affected the people in general? I know you have talked about sustainability. You talked about the family, profession; but anything which you have missed out and you would want to add.

Participant ([13:49](https://www.temi.com/editor/t/xceXUqAXIvWqkMncqb4W9XJjY9k894aAOl-lGSqsGkld5G7qzHix9MxnTWR-6PZ5MW2FpWIMWfoydgL3cPkohpeXBzE?loadFrom=SharedLink&ts=829.3)): I think I knew that as I said, I'm going back to what I just…I'm just repeating myself, but I think it's changing the world. It is changing the world. I mean, I can give you, I can just talk about my 73 year old mother who comes and the other day I said, you know, she hasn't gone out of the house, but I said, I said, you know, I'm too fed up. And she says, yes, but what can I do? What do you do? She said, I watch Netflix. I found my way.

Researcher: That's a new thing for her!

Participant ([14:23](https://www.temi.com/editor/t/xceXUqAXIvWqkMncqb4W9XJjY9k894aAOl-lGSqsGkld5G7qzHix9MxnTWR-6PZ5MW2FpWIMWfoydgL3cPkohpeXBzE?loadFrom=SharedLink&ts=863.831)):Exactly, and I thought, well, okay. But actually the point is my, why I gave you this example is I think it has, world over, pushing people into doing things. They would normally not do. They would normally not venture and normally don't think of it. Do I need to do that, I don't, I don't. People value life better, people would not take things for granted. That is what I think is how the world is changing and how it is going to proceed.

Researcher: And how did you think you have learned about coronavirus? What are the sources?

Participant: All over the media, all over the, you know, we pick up any, any, any, any television channel. Television channels are full of it. The virus cannot just be stopped from the television. I have stopped watching the television. Literally, when you put on the television..I used to only watch it for school news. I mean, after the prime minister had to go for the quarantine, so I got so fed up when I watched Dominic Raab for some time. And I said, you know, what I didn't want to watch is what changed the economic policies over the world and how they, one thing economically or financially. And that's all I want to listen to anything because probably it takes away. It was a, of a difficulty, but I think the media, media, everywhere, I mean, I have a default setting on my laptop and I'm running where it goes into BBC news. And I get to change that because I don't want to listen. I think it is the new norm. It is, it is the factual information and get it. It's good to be connected, but to the point. I mean, hats off to the media, they're really keeping their face. They distresses me. So I just stopped watching it. But to it, it is a base source.

Researcher: Which source do you think is reliable?

Participant ([16:45](https://www.temi.com/editor/t/xceXUqAXIvWqkMncqb4W9XJjY9k894aAOl-lGSqsGkld5G7qzHix9MxnTWR-6PZ5MW2FpWIMWfoydgL3cPkohpeXBzE?loadFrom=SharedLink&ts=1005.391)): I watch BBC very much, And I watch the BBC news. Yes. And I'm also, I do read a lot of, uh, newspapers, as a habit. And so that's my first, literally when my alarm rings, I have a habit of, you know, reading the Economic and Financial Times. So for me tv channels, um, newspapers, and media. And if you look at Indian media but sometimes the Indian media can be concocted. For me, it depends on how my mindset is because I don't get too drawn into them. I would look at NDTV, probably just NDTV, and not more than that. So I that's what it is.

Researcher: And what about the news, which are rolling into the WhatsApp? Facebook?

Participant: To be honest, I think that, that came, a couple of initially ,that was like, you know, all of that, but, uh, WhatsApp, Facebook. But I don't have a, I wasn't , personally, I don’t have a tendency of reading any forwards. So for me, for me, I would rather log in into BBC and listen to the news rather than yeah. Then linking them to these forwards. I think that is kind of not first-hand information.

Researcher: So drawing on what you read every day and you had been looking at the news, what is your current knowledge on coronavirus? In terms of, technically speaking or how it is spreading or anything like as technical knowledge?

Participant: Yeah, I think in terms of technical knowledge, it is obviously it's, it's, it's a novel flu symptom that you, you had. It started with normal flu symptoms, but there are obviously a lot of, um, of, uh, information coming on now saying that, you know, probably that it is not even a flu symptom, it could be something like a headache and all that stuff. So it normally spreads up and then hits your lungs at some point, which goes into respiratory, you know, actual respiratory problems. And obviously, technically you are told that you got to keep your distances from people where people could be carriers and the more contact you reduce with the outside world, the less, you know, the less chances of coming into direct contact with any of the services or people who might be carriers of that. So to me, for me, it is, um, this is all in a nutshell what coronavirus is.

Researcher: And biologically speaking, like people are talking about so many things like how the cells modify and genetic things?

Participant ([19:58](https://www.temi.com/editor/t/xceXUqAXIvWqkMncqb4W9XJjY9k894aAOl-lGSqsGkld5G7qzHix9MxnTWR-6PZ5MW2FpWIMWfoydgL3cPkohpeXBzE?loadFrom=SharedLink&ts=1198.14)):I just think, I don't, I dunno. I dunno. I don't, I haven't gone much into that to be very honest, but I just think if you're fragile anyways and you are vulnerable, you would have a tendency of picking it up more, um, pickup that's probably, you know, it hits your immunity basically. If you have a low immunity and you're in that category of a fragileness, you gotta be, you got to be carefully of yourself as well as you have to be careful, this is not an offence to anybody, per se, but you know, but you got to be careful when you are out.

Researcher: Now coming to the next question. How did you think coronavirus appear in the world, drawing from your own knowledge?

Participant: It was funny because, uh, yeah, I think right until the end of February, beginning of March, I personally didn't realize the severity of it. In fact, when things, when this, when the first case was from, it was detected, we were in Australia at that time and we were flying back from Australia. The whole world at the beginning of January was worried about the air strikes that were going on in Iran and all that sort of stuff. And you know, more of January, the whole focus was somewhere else, on America and Iran and all that sort of stuff. So I don't think when this was happening in the background with probably China was already trying to, it was spreading. It was only because of family in Asia and South Asia and personally a cousin of mine, who's a doctor in Singapore. She was the one who said to me, ah, you know, it's very far away and we are ready for that, we think we are..I mean people were about that time, already started to talk about closing the borders, closing connections, but I don't think you're up to take, to take it very seriously, I didn’t take it seriously. It was only until the beginning of March when India started to get locked, ok, that's felt like about security-I thought how come a country like India is taking action and what's wrong with us? You know, what becomes to you? And, but, well, we were going to work. My son was going to school, everything normal. It was two weeks after that, we were locked out. So, you know, honestly it didn't hit me until that happened. That was the beginning of March. So we don't, we knew something was brewing in the background. It kind of doesn't go in your face and you don't realize it.

Researcher ([22:45](https://www.temi.com/editor/t/xceXUqAXIvWqkMncqb4W9XJjY9k894aAOl-lGSqsGkld5G7qzHix9MxnTWR-6PZ5MW2FpWIMWfoydgL3cPkohpeXBzE?loadFrom=SharedLink&ts=1365.34)): And so do you think that your understanding of this pandemic has changed since it has begun?

Participant: Yes, of course. I mean, I think, um, I brought ,my sort of, going back to media, the more you read, the more advancement in medical science is, the more advancement in biomedical, and the amount that is coming through now, the press of course, I mean, you do see that people- there's a lot of, and there's a lot of progress towards finding a solution. And I think in the amount of progress that has been done in the last probably three months have been achieved, it been achieved till this date, the amount of how quickly this thing has grown, sort multiplied all over the world. It would never had happened, if, uh, if, uh, it was not at the speed of the spread. Medical science has coped and responded phenomenally to whatever I see now. Ok, I'm not, uh, uh, sort of, I haven't read much about the depths of, uh, you know, where the shortfalls are, the deficits and how they sort of coping with it. But I just think on the face of it, reading headline figures, um, it'd be without doing and feeling the industry, dealing with the, you know, the, the front end of it, dealing with the back end of it. They're going to look for a solution to the problem. They are trying to find an explanation for the problem, vaccination of the problem. It does, it does sort of, um, it has taken a kind of, uh, a leap which, which is good.

Researcher: So your perception has also, which I make sense of it has changed, right? Like initially you also didn't think it would be that much serious. It is now in a scale that you would not have expected?

Participant: Yeah, honestly. Yeah. End of February. My husband has just returned. Two weeks in February and he returned at the end of February, and then I think about four or five days, she was told to fly out again, to Mauritania and a friend said to him, uh, such thing, don't go, you're stupid if you go, uh, you know, the airports are locked out. He said, I am going for a meeting, fortunately, it is not a meeting, but, my, my life will depend on it. Right. And then he went. Honestly, he went. He called me from the airport and says, the airport is a graveyard. And, um, yeah, so that was the end of February. So he flew literally, until the first week of March, so he came back on 4th of March and he said, you know, I've seen the outside world. It's really bad. So stop doing what you're doing and stay home and stop, stop, uh, going to school. And I said, what rubbish are you talking? I am not the kind, you know one, who will give up to it, and then I went to work until the 19th of March. 19th of March 20, was my last day. So I worked until the 19th of March. Okay. And that was two and a half weeks after that. India locked down from the 9th of March.

Researcher: So you were in a refusal mode!

Participant: Yes, I denied it. You see, it was completely living in denial. And there were things spreading in the background, I knew that everything was happening. I knew that people are panicking but me being me, I tried to.. I am not the one who leave it so early, unless somebody tells me you're stupid. My mother was shouting at me. The school was open. I stopped working.. I mean, I stopped going to work on a Thursday. I didn't stop going to school on a Friday.

Researcher: What do you think, from your sense - how did this virus appeared in the UK?

Participant: I believe in a global world. The world is so small now. Now, the people traveling all over across the continent, and you can be from one end of the world to the other in 48, 48 hours. So it's human, it's human, a transmission. So we never closed our, our borders or our, our airspace, till what- till end of March, beginning, in the of March. So I think that was the biggest, the biggest culprit was that. People were just coming in and out.

Researcher: And, do you think these pandemic is anything different from any previous pandemic we had?

Participant: Not in my life time, but I can’t imagine in my life that I have seen. I haven't seen, but I've read, what I read. Um, uh, I think it's going to be worse than what they talk about. The, you know, sort of the dark ages of the early 19 hundreds, where people went through massive recessions and the word was going through this, um, massive, uh, dark times. But, um, I don't think there was enough in a lot of those situations and circumstances. It was also an economic downturn. This Is obviously resulting in economic downturn, the economic downturn will create out of it, unlike, 2008, whereas economic downturn created more problem. So it could be .. It could be, um, isn't a medical impact. And because, uh, it is, it all is solely dependent on an invention or it's going to be very hard. And I think it is what was maybe going to land up in, because it's just the bang and we can't control it. So you just want a deck.

Researcher ([29:21](https://www.temi.com/editor/t/xceXUqAXIvWqkMncqb4W9XJjY9k894aAOl-lGSqsGkld5G7qzHix9MxnTWR-6PZ5MW2FpWIMWfoydgL3cPkohpeXBzE?loadFrom=SharedLink&ts=1761.15)): And what do you think that your government has dealt, how they have dealt with this pandemic?

Participant: The government, firstly, I'd say the government reacted late, we should have closed and stopped a human contact. That we have stopped this sort of, uh, you know, uh, people going out and, uh, locked down. I mean, we could have done it atleast two and a half weeks before we actually get so that would have reduced number of lots of flights, um, definitely. I think they definitely reacted late because, um, the approach was a bit wrong. I don't think they were completely correct in terms of this herd mentality and approach. And, um, I'm sure. I mean, if you look back, everybody would have said they really say that, uh, had we closed, we would have saved more lives. The question. But I don't know that there's anything right or wrong at this point. The countries who close down earlier, countries were affected earlier and reopened and having a resurgence. So is that going to happen to us? You know what I mean? So, I don't know. I really don't know. You don't end up in the facts and figures, it all depends on how the countries see the various points. But I think we should have closed earlier.

Researcher: And what was the information about coronavirus that is most surprising to you?

Participant: Just the spread of it. I mean, the very fact that I see virus all over the world, but you can contain it. And you know, there are vaccinations. But nobody ever, never, ever thought about it, even take for instance, yellow fever or Ebola. Okay. Africa is probably one of the less fortunate continents in the world that they keep getting this. But there has been ways that it's been controlled really quickly. So you can turn to Africa, you can live in Africa and you can be inoculated and you get away with it. But I just think it's that unknown and nobody has been able to crack it. It’s amazing. Yeah.

Researcher ([32:01](https://www.temi.com/editor/t/xceXUqAXIvWqkMncqb4W9XJjY9k894aAOl-lGSqsGkld5G7qzHix9MxnTWR-6PZ5MW2FpWIMWfoydgL3cPkohpeXBzE?loadFrom=SharedLink&ts=1921.96)): When you discuss about coronavirus with some members of your family, your friends, what do you mainly discuss?

Participant: To be honest, we have overcome the fear phase, there is, there was a phase of fear, as I call it. And now everybody's starting to think that we have digested that pill. Okay. Can we be talking about this new lifestyle. Can we get on with this? What is it that we can make, we can do to keep this one element of sustainable living up? Is it exercising? Is it, you know, getting ourselves into sharing more in terms of health and wellbeing, how are really going to keep ourselves motivated? I think now if I see my family back home in India, or whether it's my friends get, everybody's trying to motivate each other..that's that's very positive for me. Yeah. And I think that is very good because the first couple of weeks was only that fear psychosis.

So I think now people are passing to another phase. How do you deal with it? What can we get out of it? This is, you know, is that going out and riding a bike for 20 minutes of the day brings happiness? You see what I mean? So it's really been within the norm. We've probably only taught us going on a bike or one of us going on the bike, finding ways to do a full of them.

Researcher: Though you have already discussed something about your personal life, how it has been affected. Anything else you want to add that how your personal life has been affected by this virus?

Participant: I think my personal life, in fact, been pretty much good. There was a time when I used to find it very hard, as a, as a very ambitious mother to balance my professional and personal life. And I think now I can balance that much, better, much better, although it has taken a toll in other ways, it does look, you know, having to do a lot of more work on the home front having to put all sorts of stresses on the actual domestic work. But if I keep that aside, I think other than that, I spend more time at home. As I said, spend more time with my son. Um, I can still do my work. Okay. It's probably not a hundred percent efficiency, but it is 99% efficiency.

Researcher ([34:41](https://www.temi.com/editor/t/xceXUqAXIvWqkMncqb4W9XJjY9k894aAOl-lGSqsGkld5G7qzHix9MxnTWR-6PZ5MW2FpWIMWfoydgL3cPkohpeXBzE?loadFrom=SharedLink&ts=2081.18)): So, a pattern has emerged?

Participant: Yes, a pattern has emerged. I think we all are really adjusted to that pattern. Everybody's coming out of it with the new adjustments.

Researcher ([34:54](https://www.temi.com/editor/t/xceXUqAXIvWqkMncqb4W9XJjY9k894aAOl-lGSqsGkld5G7qzHix9MxnTWR-6PZ5MW2FpWIMWfoydgL3cPkohpeXBzE?loadFrom=SharedLink&ts=2094.41)): How would you describe the typical day of your life now?

Participant ([34:59](https://www.temi.com/editor/t/xceXUqAXIvWqkMncqb4W9XJjY9k894aAOl-lGSqsGkld5G7qzHix9MxnTWR-6PZ5MW2FpWIMWfoydgL3cPkohpeXBzE?loadFrom=SharedLink&ts=2099)): A Typical day? ok. If I put in my husband's words, 'I'm not like her. She gets ready in the morning. She, she even does her hair, her hair, and she wears her office clothes and the shoes for the office, probably not her shoes on. And I sit in my bed with my phone next to me. She starts before me. She starts at 8:30, I start at 9. I start at 8:50, just because I am checking my emails and getting up speed'.

My first call, literally with the team at 9 and I shut out, I don't get up of my seat, till 12:30. Yeah. So 9 to 1230 and then 12:30, earlier I used to have half an hour break, but now I'm at home and I'm having to, you know, sort of work out the lunch and all sort of stuff. So I have one hour break. Some time, when I don’t have enough time, get all sorts of overspills, you know, I have to check his (son) work, all the spirits I have to check his work and make sure he has uploaded his work, that’s fine. But take it that is my one hour lunch break. 1:30 I am back again, flat out till 5. And you will not believe it three times a week, I have got a club, we do yoga, we do pilate. So I do yoga on Mondays and Thursdays. I do pilates when I can. I get up, quickly do something, go back into the yoga class, finish that, get up. If I'm not doing that, my son and I are on the bikes. So we take our bike, cycle right down to the Gulf course and we come back by six 30. And then it's okay, then that I have to do my housework. And then you have backup reading, reading the books and off to bed.

Researcher: You know, I really envy that kind of live, being so much disciplined.

Participant ([37:20](https://www.temi.com/editor/t/xceXUqAXIvWqkMncqb4W9XJjY9k894aAOl-lGSqsGkld5G7qzHix9MxnTWR-6PZ5MW2FpWIMWfoydgL3cPkohpeXBzE?loadFrom=SharedLink&ts=2240.28)): Yeah. That's what I said. This is my husband's world. He, he's just as productive. Hmm. Okay. All right. But he has a very different lifestyle. For him, he works at dawn, 4am in the morning. By the time I get up in the morning, I see the light on- he had been working. So he has a very different life style. But he has a set pattern.

Researcher ([37:46](https://www.temi.com/editor/t/xceXUqAXIvWqkMncqb4W9XJjY9k894aAOl-lGSqsGkld5G7qzHix9MxnTWR-6PZ5MW2FpWIMWfoydgL3cPkohpeXBzE?loadFrom=SharedLink&ts=2266.11)): But you are more focused and disciplined.

Participant: Yeah. And for me, if I have in my mind, seven hours of work, then technically I have given my best.

Researcher ([37:55](https://www.temi.com/editor/t/xceXUqAXIvWqkMncqb4W9XJjY9k894aAOl-lGSqsGkld5G7qzHix9MxnTWR-6PZ5MW2FpWIMWfoydgL3cPkohpeXBzE?loadFrom=SharedLink&ts=2275.351)): Yeah. It's a new, pattern of life. And having said that, how do you think that this pandemic will end?

Participant :It will end when they have discovered a vaccination. and that we are all going to be inoculated. I do feel going back to work. Um, I do feel the world...We live in to look past the economic downturn if we continue to make progress slowly. And obviously, um, so I think people would come back. Things would come back, but there would be lot of restrictions. The downturn will also have a have a different impact again. Today, I saw, they're talking about issuing a number of passports, uh, facilities, obviously, how are they going to maintain social distancing is a big issue. So I think that somehow it would have to come back, but it will come back for only those who can cope with the system. It will be even not be a pressure driven comeback. And it will obviously only come back to near normal. I think another 18 months, that’s a long time. At the very least I'm telling you, I mean, I know the price of the property, property industry has come back. This week in Olympia, which is one of the biggest housing providers in the country has started afresh. But at the end of the week, we had the managing director talking about the stringent measures they have to put in place just to get their sites unlocked. Who is going to work, I said. You don't want to go to work. That's not the case world over. In UK, you can say that because there's an element of social security in the average person on the street, but that's not the element across the world.

Researcher: How do you think that we can, uh, prevent this kind of pandemic in future? That's the last question in this section.

Participant ([40:22](https://www.temi.com/editor/t/xceXUqAXIvWqkMncqb4W9XJjY9k894aAOl-lGSqsGkld5G7qzHix9MxnTWR-6PZ5MW2FpWIMWfoydgL3cPkohpeXBzE?loadFrom=SharedLink&ts=2422.481)): A very hard, I think really hard. Medical science is, um, as I said, I am not a medical professional and, um, I think BioMedica, uh, the people, you know, dealing in this, uh, in the industry, the, I am sure they do understand and know, or all of this. But, I don't know. I don't know. I don't know how we went to ever cope with another virus and may be this is productive-. Uh, a lot of things that people never thought about earlier. People were not prepared for this. So whether there's going to be a vaccine that is going to be cope with any virus in the world, I don’t know.

**Part-II**

Researcher ([41:22](https://www.temi.com/editor/t/xceXUqAXIvWqkMncqb4W9XJjY9k894aAOl-lGSqsGkld5G7qzHix9MxnTWR-6PZ5MW2FpWIMWfoydgL3cPkohpeXBzE?loadFrom=SharedLink&ts=2482.9)):So that brings up to the end of the part one. Okay. So now I go to The second part will focus on the South Asian community. And your understanding of the effect of this virus on this community, uh, I will start by asking you, um, what do you think are the, some of the health concern for people in your community during this pandemic and why?

Participant ([42:00](https://www.temi.com/editor/t/xceXUqAXIvWqkMncqb4W9XJjY9k894aAOl-lGSqsGkld5G7qzHix9MxnTWR-6PZ5MW2FpWIMWfoydgL3cPkohpeXBzE?loadFrom=SharedLink&ts=2520.57)): When you are talking about the community, are you talking about in the UK context, are you talking about in India context?

Researcher: Anything, which you think about your community?

Participant: Okay. I just think that in the UK context..

Researcher:

Yeah. You can talk about UK first and then bring in, um, India.

Participant: Okay. In the UK context, I think there is no differentiation and it's anybody, uh, you know, it's, anybody gets treated, anybody that access to services is everybody's level playing field. But I don't see it being a different experience to. But I think I might have it different it in terms of India context. Yeah. I'll find in India lot of people who are vulnerable, just the sort of, you know, um, access to services predominantly because of infrastructure. I don't think they have the infrastructure to cope. Um, we don't have the, the, the amount of, uh, basic, you know, needs in terms of health services within the country. So I think there's a little, if I'm sure it is already there, it's just that the data's not available to the world. I dread to think what if it, if it hits at the rate it has hit Italy and UK, I think it'd be disastrous.

Researcher: How they would cope with that.

Participant ([43:35](https://www.temi.com/editor/t/xceXUqAXIvWqkMncqb4W9XJjY9k894aAOl-lGSqsGkld5G7qzHix9MxnTWR-6PZ5MW2FpWIMWfoydgL3cPkohpeXBzE?loadFrom=SharedLink&ts=2615.99)): Yeah, exactly. And I think they don't, don't have the infrastructure. And at the same time, there is this, the element of social segregation, which is happening in the UK, like the grandchildren and the grandparents, not meeting. Social structures in India will not allow that. You cannot tell grandparents in India that you will not allow your grandchild in the house.

Researcher ([43:59](https://www.temi.com/editor/t/xceXUqAXIvWqkMncqb4W9XJjY9k894aAOl-lGSqsGkld5G7qzHix9MxnTWR-6PZ5MW2FpWIMWfoydgL3cPkohpeXBzE?loadFrom=SharedLink&ts=2639.55)): So how do you think that, like, people living in UK or South Asian people are the more or less vulnerable to the virus?

Participant: I don't think, I think, I just think the, I, to be honest, the, the kind of people, you know, the kind of people that I, uh, associated , are everybody my age. So I really can't tell about how it is affecting maybe the, or, you know, sort of, it's hard for me to say that, uh, I mean, Indians in the UK, are not different from Indians in India. They would have to go through this same regimented approach where they, for instance, if you're in a care home, you are not seeing your family two months; would they be able to cope with that? I think it's going to be hard. So even if you're in India or in the community, your community is very tight knit.

Researcher: So, how do you think that, like people living in UK or South Asian people, are they more or less vulnerable to the virus?

Participant: I don't think, I think, I just think the, I, to be honest, the, the kind of people, you know, the kind of people that I, uh, associated my age. So I am really content about how it is affecting maybe the, or, you know, sort of, it's hard for me to say that..uh, I mean, Indians in the UK, I don't differentiate from Indians in India. They would have to go through the same regimented approach where say, for instance, if you're going to be home for two months and you don't want, I mean, I think it's going to be hard. So even if you go in India, community is very tight knit. Everybody has to see everybody and activity has to be ongoing and continuous.. social rejection is broken for the community. So I don't know much, but I'm sure. I mean, I know one case when his mother is in the house and she has to isolate this because his wife is a medical professional at the end of shift. And I was really shocked. And I said, why is she sort of isolating in the house? And he said, yeah, unfortunately she's using the kitchen in the, in the, um, in the shed. They had an extra space, but that's so cruel, even at home. Okay? Indian grandmother living in this country…She can't be durable. And you know, she's probably going through depression that place.

Researcher: So, how do you think that, like people living in UK or South Asian people, are they more or less vulnerable to the virus?

Participant: They would have to go through the same regimented approach where say, for instance, if you're going to get home for two months and didn't go for that, you don't want, I mean, I think it's going to be hard. So even if you go in India, probably illegally, either community is very tight knit. Everybody has to see everybody and everybody has to relate to everybody be ongoing and continuous social interaction- you see, that is broken for the community. That is not helpful. I don't know anyone, any examples, of what I said, because my interaction is with the same age group, you know? Yeah. So I don't know much, but I'm sure. I mean, I know one case when his mother is in the house and she had to self-isolate because his wife is a medical professional at NHS. And I was really shocked. And I said, why is she is self-isolating in the house? And he said, yeah, unfortunately she's using the kitchen in the, in the, um, in the shed. They had an extra space, that's so cruel, even at home. Okay. She has an Indian grandmother, lived in this country only for less than 10 years I guess. She can't be told to go- And you know, she's probably going through depression in that place.

Researcher ([47:10](https://www.temi.com/editor/t/fdp3bHB4pdahyE1UuAoNvUtGNC34576cyMGfahakmI_U7_uLJp9FCthf95v7BMbaqW49J5zJFni0_tcTWtjo-rytPF8?loadFrom=SharedLink&ts=130.88)):So do you think it would have been different from a white British family?

Participant: I think it would be very, it is different. I'll tell you the reasons why, because from a very early age of, early, um, sort of the social, the social structure is slightly different, um, there is, um, I mean, you don't live in a city. I think the British families do not live in joint families. They live in nuclear families, and they do see their parents; there's a lot of interaction, don’t get me wrong there, but that, that, is in a very controlled manner. Unlike ours, where, you know, in Indian community, where you know, you can door knock and your grandparents can come to your grandchildren anytime of the day in, well, you know, you don't hesitate to do that. So, you can't have those grandparents, you can't have those, you can't stay away from it. Very simple. if my mother was here, I would never tell her to go to care home. You get me. So if it is not, it is not just a normal norm for us to do. So I think that way Indian community will struggle to be able to adjust to the social distancing.

Researcher: So it can affect negatively to the health?

Participant ([48:38](https://www.temi.com/editor/t/fdp3bHB4pdahyE1UuAoNvUtGNC34576cyMGfahakmI_U7_uLJp9FCthf95v7BMbaqW49J5zJFni0_tcTWtjo-rytPF8?loadFrom=SharedLink&ts=218.87)): Yes, of course, psychologically. You know, overnight I have to turn to my mother. You know, that’s what I said, you know that’s only one family. She is isolated in the house. That is awful, its hard!

Researcher: I know! Now coming to that, uh, do you see your family being affected by this coronavirus, virus like, um, in terms of, uh, your daily health practice or Healthwise?

Participant: Yes, of course. I think ..that's what I think. Um, I mean we all go through the phase of fear, we all gone through that sort of element of, you know, finding out, on the grape vine, you know, you will hear, you know, if you take so and so, it will keep your immunity up, all that sort of stuff. So, you know, in times of uncertainty, you believe that, however, intellectually, when you think, you are rather knowledgeable than you are, you will still give in. My husband at went to the pharmacy at our road end, You know, he said, I heard this and I read this, what shall I do? And now he would give him a strip of Vit C tablet, and asked him to take it every day. I said, this is rubbish. This is only vit C tablet- it just keep the immunity and not help with anything else. How much did you pay for it? I paid £16 for it, because it is high level vit C.

Researcher: So just taking measures to boost immunity and to keep fit.

Participant: It is just fear psychosis.

Researcher: Now coming to the government introduction of the safety features like, uh, the social distancing restriction on travel, work from home, et cetera, et cetera. So are there any specific difficulty, do you think the South Asian community will face to abide by these measures?

Participant: I think, personally, to be honest with you, I, I feel confident that I can pick up the phone or I can sit in front of a video conference and speak to people. For those people who have not faced the camera that much, people I have in the team meetings, who cannot speak up for themselves. The work usually would have spoken up for them because of the work environment, people knew what they were doing, they didn't have to talk much. There are all sorts of, uh, you know, uh, sort of complex that people have in speaking and sort of, which does not help them, in the office environment, you don't bother about these things. But right now, the very fact that you're seeing the camera, you have to talk, sensibility, you know what I mean?

They are being constantly watched, you're constantly watched. You're constantly monitored. Okay. They are not used to that. People are good at their work, and it works for us, but now there's a lot of speaking for us. If you don't talk about it, or don’t show- it is hard for us! So easy to us!

Researcher: Is it something related to the South Asian people?

Participant: I think it is generally non-British people. I think it is generally related to non-British. Because the British community, predominantly is good at articulation, they articulate themselves, they know how to present themselves. They know how to...they have different faces. You meet the same man in a pub, he's very different. You meet the same man across a, a board room table, he's very different. You know, all that, the same person can suit to change his personality. But we have not been groomed in that way.

Researcher (52:41): That's very interesting. I hadn't thought of that way actually.

Participant: Yeah, to be honest with you, I'll be very honest with you. This is, I'm just giving you snippets from what my husband and I go through. In massive board meetings, ok, this is about 15 days ago. So he had to sit in a suit from nine o'clock in the morning. I think he finished about four, five. He was in here in this room. He worked in a suit the whole day. He was totally exhausted. He would wear a suit before, he would wear it every day at work. But no, the fact that the camera was on for the whole day. So, see if it was not that it was that you, have now to focus, when you talk, when you speak, when you come across and earlier you were a part of the whole, okay. You were in a room, but somebody never focused on you, it’s like.. like looking through the lenses of the camera.

Participant ([53:58](https://www.temi.com/editor/t/fdp3bHB4pdahyE1UuAoNvUtGNC34576cyMGfahakmI_U7_uLJp9FCthf95v7BMbaqW49J5zJFni0_tcTWtjo-rytPF8?loadFrom=SharedLink&ts=538.14)): It's very different. We never realised, oh yeah, that's true. Correct. I have a long shot. Honestly. I'm telling you this, this is a very stupid, probably I shouldn't be saying this one. You know, six to eight weeks I have not bothered, I haven't gone beyond putting basic makeup. This weekend, I thought, oh my goodness, I have to take out all my make-up, because on this Wednesday, I have to watch a workshop where I've got 20 consultants on there. I'm running it, I'm running a workshop with all of these people and these people has to take me seriously. Otherwise 1 million pounds of fees can go down the drain. I have to be presentable, and I've got to make it feel as important as a meeting as myself. You see what I mean? So, you don’t realise those. It is some new realisation, which was part of the life and it's gone. And now you have to put that practice back on. And what about like, it's, it's, it's a very good reflection on the working from home. How about like the other restrictions, like social distancing, for example.

Researcher: How do you think that South Asian community are coping with that? Or do you think they are coping with that any differently than the British people?

Participant: Um, I don't know about the British people as much, but I have a lot of, a lot of Eastern European friends who, in a way are very much like us. I think that Asian community or the Indians per se, I finding all sorts of platforms straight to interact. It does social distancing. I mean, you know that the three of us (friends) live in a line, we've literally, you know, next door to each other. I Have not seen my neighbours in eight weeks.

Researcher: That's commendable.

Participant (46:00): Yeah. Since middle of March. Yes. I literally haven't seen them. probably, actually I say hello to them is when they're working in their house and we passed on my side door. Just wave my hand when I say, I, I know I can speak to them from my back garden, but I actually I have not physically met them. you see? So I think a lot of ways we are sticking to a social rules. And, but we are finding it rather hard. And there's a lot of talking going on, on that you would meet on all sorts of platforms, which is media platforms, which is food sharing. Whether you like it, or you don't like it it's a different story. But at the same time, it's like the, the, the opportunities that will come there and talk, we are your friends, you speak to your friends, you blabber, you say whatever rubbish you normally want to get out of your system. You know what I mean? It's happening. It's happening all around. That is good.

Researcher: So, and now I'm talking about the health care, right? So do you think your community feel they can access the healthcare in the same manner as white people?

Participant: Yes. I think, I think, um, I think access, generally access to healthcare is yes. Obviously the system is now so blocked, whether you will actually get access to a different sort of, it is completely different ball game. I think general access, health care, as I said again, I caveat this because the people I sort of interacted sort of, you know, the young immigrant population, immigrant population, Indian population. So everybody's, um, equal rights to this country. Everybody has equal access, which is, which is fine.

Researcher: Yeah. And in some sense, do you think the South Asian community trust the government, UK government in the way, they are taking decision about the coronavirus crisis?

Participant: I don't think, I think the whole of the UK, uh, sort of feels, um, South Asian community per se. I don't know much about their views in general, as I earlier said, but in general, I feel that there has been a slippage and I don't think the South Asian community will deny that. And, um, yeah, I think it's a general consensus across the country, there are slippage problems.

Researcher: You necessarily do not see any difference of views between South Asians or the British. Right. And to, what extent do you think that people in the South Asian community understand the health messages surrounding the crisis and what could have been done to improve in delivering those messages?

Participants ([59:18](https://www.temi.com/editor/t/fdp3bHB4pdahyE1UuAoNvUtGNC34576cyMGfahakmI_U7_uLJp9FCthf95v7BMbaqW49J5zJFni0_tcTWtjo-rytPF8?loadFrom=SharedLink&ts=858.45)):I think the biggest, the biggest problem that, um, South Asian community per se faces is, uh, is language. And I think a lot of those that messaging or media, um, messaging was done in different languages or translated to different communities. Uh, it will help. As I said again, I don't have any direct, direct communication with the, with the people, you know, Asians or South Asians, and sort of all. But we do projects in, we do have a lot of South Asian communities like East London and where we have to interact with people. And the best way I have noticed as a designer, as a, as an urban planner now, uh, one of the biggest challenges you face, it is not only with the South Asian community but any non-British community is language and being able to actually go down to them, to them rather than them having to come to you. So the system has to be taken to them. So you see what I mean?

Researcher: How do you propose to do that?

Participant: Like just translating, I can talk a bit about the planning system. Uh, I mean, I can give you an example, very simple example, but you have to do it. We found it so effective. We have project running in Birmingham in a place called Lozells. Lozells is 90, say 90% of it non-British, of which 60% South Asian. What do we have to do, just put things in perspective, we have to do consultation on every project that we do. So we have to do community consultation. So literally, the project has to go to the people other way. If it is a new town, then it, it obviously there's no existing community, but if it was a region project, then you're looking at an existing community. let's take a look, if we had to buy an internal project in different forms. So the first level we do that at different stages of the project. The first level, when we took it to the community, the feedback we got from the community was appalling. And we did probably get even five or 7% of the people come and speak to us. We went back to the drawing board and discovered we had to tear it all up because we were looking at a utopian project. Okay, which were completely irrelevant on the ground.

Participants ([60:55](https://www.temi.com/editor/t/fdp3bHB4pdahyE1UuAoNvUtGNC34576cyMGfahakmI_U7_uLJp9FCthf95v7BMbaqW49J5zJFni0_tcTWtjo-rytPF8?loadFrom=SharedLink&ts=1015.12)):The reason being, we were talking to a community that didn't understand English. you got me, you're talking to interact with these persons- I think majority of people in the area were Bangladeshis, you were talking to these kinds of people, who were so scared to come out of their doors, and come to see other people. I Mean, I remember I was not even a very senior at that time. And I, uh, as a, uh, senior designer, I was asked to move on to the project simply because I could speak Hindi and they, and obviously I did not understand Bengali, but neither did I understand Urdu.

Researcher: So, so that's how like you build the confidence in them and in the communication.

Participant: Exactly. We literally went street to street, 4000 houses. I just gave you this example to tell you how I think people do face this kind of, uh, uh, barriers. Uh, it just that you don't realize it because I understand we are different, basically English speaking migrant community.

Researcher: So do you think that these, these community, as, as you mentioned maybe going through any crisis at the moment?

Participant: Of course, I'm sure they are. One example I was giving you, the different, the different, uh, what do you call it? Uh, uh, different generations in one household. And the very fact that all these generations with the vulnerable generation within the household, how are they coping with it is, is, is very, very frankly, do we think about what we are in the country with a law saying that you're not supposed to have vulnerable people around you. That's a very big issue. And the second thing, is that, of course, how, um, language, as I said, I know people who cannot pick up the phone and talk to the NHS. Why? Because it's not that we cannot do that, but the community is tongue-tied. I cannot just call 111 and ask can you talk to me in Bengali or can you talk to me in Urdu. It is not that you don’t want to do it, but the community is tongue tied.

Researcher: And what about any, any attitude, attitude issues, or any behavioural issues, which may hinder their understanding of the crisis. Do you think you can think of anything?

Participants (64:00):I think, knowledge. As I said, you know, lack of lack of information, lack of, uh, actually, um, not being told, given the correct information. Same problem you face in India, same problems. Apart from that my connect with them as much, as I said I am on the planning side of things, but, uh, I'm sure that social issues are just the same because the community, the mindset of the community are same, depends on their living style.

Researcher: Okay. Now we are to our final question. Okay. So finally, I'd like to ask you, what do you think has helped you and your community to deal with the crisis.

Participants ([65:45](https://www.temi.com/editor/t/fdp3bHB4pdahyE1UuAoNvUtGNC34576cyMGfahakmI_U7_uLJp9FCthf95v7BMbaqW49J5zJFni0_tcTWtjo-rytPF8?loadFrom=SharedLink&ts=1245.41)): What has helped? I mean, what has helped me? It just, it just my own personal, I think it's just determination and positivity, to try to come out of it. To do as much as we can within our own environment, within our own powers, within our own setup. I think that it is the new norm and you're going to find ways and means to look at things that will help us to come out of it rather than sit and sleep. I think, as the, sort of the educated, um, forward thinking segment or the Indian community, I would put it as saying, um, we've just got to be positive, just think in the interest of everyone, just think not only in the interest of yourself, but your family, your community, the people around you, I think with everybody's struggling and everybody is going through this new norm. it's not only us.

Researcher: Great. What about the general population of the South Asian people? Any like, for example, any specific aspects, do you see, like a speech, religion, um, community?

Participant: Must be, must be, uh, sort of, you know, um, depending on a lot of, uh, external, um, external factors that help them to cope with their, um, anxiety. Sort of cope with the depression, the distress, that what a, it could be religion as you already pointed out. What if it could be community interaction at different levels and different ways, sorry. In some levels, in different ways. What if it also be that in the household you have sort of you know- I don't know whether, I mean, for instance, one of the things we've found, I mean, it's not nearly, but my husband has found is he never enjoyed cooking as much as he does now. So here, for instance, he has got the recipe book and he cooks for two hours and it is a de-stressing. So I think there are these subtleties that people are picking up and sorting, which, which is probably going to temporarily get them out, and in the long term, you know, revolutionize a lot of things in the world.

Researcher: Right. So we have finally ended our interview. Is there, do you think anything we have missed, or do you want to add to this entire discussion we had?

Participants ([68:49](https://www.temi.com/editor/t/fdp3bHB4pdahyE1UuAoNvUtGNC34576cyMGfahakmI_U7_uLJp9FCthf95v7BMbaqW49J5zJFni0_tcTWtjo-rytPF8?loadFrom=SharedLink&ts=1429.16)):I think I've spoken too much.

Researcher: You have no idea, that you had been a brilliant speaker, so I am quite loving it. Yes. Thank you for your participation in the interview.
