# Supplementary material for: Mitigating the identity and health threat of COVID-19: Perspectives of middle-class South Asians living in the UK
Source: J Health Psychol. 2021 Jun 22;27(9):2147–60. doi: 10.1177/13591053211027626 (PMC9353968; doi:10.1177/13591053211027626)
Supplement: sj-docx-7-hpq-10.1177_13591053211027626 – for Mitigating the identity and health threat of COVID-19: Perspectives of middle-class South Asians living in the UK [file sj-docx-7-hpq-10.1177_13591053211027626.docx]

**UK 4 GROUP A - APR MAY 20 – TUSHNA VANDREVALA – KINGSTON**

**Transcription note: A very strong Indian accent on this one. Some words could not be deciphered.**

**I:** Thank you for participating in our study. I will start with the question about your understanding of what do you think is happening to the world now?

**R:** It was initially as understood by the whole world is from China. And then, the data of the repetition wasn’t even out by China immediately. People travelled from China to various parts of the world and probably it is spread across various places, that is number one. Number two, the majority of the countries initially did not take this very seriously, because it was considered to be just flu from symptoms which can come and go and never ever anyone thought that this would be serious. A threatening pandemic which would spread so fast across the world. So, number three, once people started understand the governments started understanding the deadliness of the pandemic. The general public did not take it seriously or neglected government’s instructions. Number four, isolation and social distancing was suggested, but initially people did not bother about it and then went on with the regular life and work. I do not blame the general public, because everybody is on their own situation in life and they have to continue living. People are working people went on working and **[Unclear]** 02.25. Everybody was into their own mojo. Ultimately, when people started realising the real spread of the virus then the social distancing and all started. By then, the bubble broke and the bubble burst and then the spread was like too big by then.

**I:** Correct.

**R:** The whole world realised that the virus really spread across a number of cases increased on a day to day basis. If you look into the number deaths in Spain, Italy.

**I:** I can’t hear you. Your audio has stopped.

**R:** It spread now. Everyone has realised that once the social distancing has started and strict lockdown measures are followed and it’s under control to a certain extent now. In some cases, new cases have come down the number of deaths have gone down, especially in the UK.

**I:** Correct. What comes to your mind when you think of Coronavirus?

**R:** Hygiene, that’s it.

**I:** Hygiene.

**R:** Once you come from outside, you remove your slippers or your shoes outside the house and enter the house only after washing your feet, your face, your hands and everything. We are getting back to basics. Today we have this habit of even I used to do that as of now, I start again. I come from outside and started to walk into the living room, kitchen, everywhere with my shoes off.

**I:** Correct.

**R:** Now, it has given us a real understanding of hygiene. You have to follow the basics.

**I:** That is a very very important and I am impressed because this doesn’t come to me naturally when I think of Coronavirus and to many people. Thank you for that. How do you think Coronavirus has affected the people in the world in general?

**R:** I think it is a humbling experience as of now to every human being. This is my own perception.

**I:** Absolutely.

**R:** Anybody can contest it. Even you can contest it around me on this. I feel this is a very humbling experience. It’s not what you earn, where you live or what cars you drive and how much money you have put aside or whatever it is. Ultimately, life is the most important thing and human beings are the most important entity on the face of the earth. It also has shown various things across. Now, I’ve can see little new birds, new sparrows for the first time on our lawn. Seriously. Everything is so pristine and so clean. It is a shame that we have to wear a mask and go outside and not enjoy the purity of the air outside. Usually, we have lawn down and the seeds they have become clean. The air space is clean and the whole atmosphere is so very clean and pristine. Nature and to human—overusing our given resources. It is a not just overusing. We are abusing the nature.

**I:** Correct. That is coming back to us now.

**R:** Nature also has its own, what you call? The **[Unclear]** 07.17 nature also lost its patience.

**I:** How do you think it has affected the people like their livelihood and their general daily life. How these people have been affected by it.

**R:** It’s the sad part of the whole pandemic. Apart from whoever it is. People have, millions of people lost their jobs. Millions of people are **[Unclear]** 07.47. Millions of businesses have gone down. Loads of businesses where we don’t back up and have closed down. Sustenance is a problem. If you look into for example **[Unclear]** 08.03. I opened a restaurant, okay this is my personal experience. I opened a restaurant. It opened on March 16 and closed on March 21.

**I:** That is very bad timing, isn’t it.

**R:** Thousands went into the whole set up. A lot of expectations and a lot of excitement and everything and this happens immediately close down. We just opened on Thursday on 30^th^ of April only **[Unclear]** 08.44. One was from March 21 too. April 30. They were closed.

**I:** You hope to open soon after this pandemic passes?

**R:** It all depends upon on the government rules and regulations. We are open now only from Thursday to Sunday for takeaways and deliveries. I am working, I am working on a very thin staff. That is two kitchen staff and that’s it. Three people in the restaurant and then nobody coming in and hanging around and for the third time the **[Unclear]** 09.33 we just keep it across the table there, outside and then people will just collect and then leave.

**I:** You are one of the worst to heat and all the people, right?

**R:** Yeah, yeah, true.

09.49

**I:** When coming back to the knowledge of Coronavirus, how have you learned about Coronavirus? Which other media sources you rely on?

**R:** Today, social media is very powerful, apart from your regular media, TV as well as newspapers. Social media is very powerful and so the news or whatever it is are being shared amongst friends, people and groups and all that stuff. That is number one. But also there is another harm of social media we are in and unwanted and unnecessary are irrelevant. Information has been spread. Looking into all this and getting back to the question and it is through social media, newspapers and then regular media, TV. That is where I got my information research a big come back. Understand this thing and then we follow strictly on the rules and regulations, staying indoors, social distancing. Wearing masks and going up in regards when we work in the restaurant everybody wears a mask and then we also have our gloves on and then we no shaking hands and no hugging and kissing and nothing. It’s just, I think the whole Indian way is the best way today.

**I:** What these various sources, which one do you think reliable like how much information do you really see from the social platforms, like—etc.

**R:** There is loads of good information which is shared across along with some what you call fast information.

**I:** Correct. Based on the knowledge which you have got, what is your knowledge of the virus, which you have?

**R:** My understanding of Coronavirus. Okay. It’s very little understanding and not much scientific information I have. Sorry.

**I:** We are scared to cough these days.

**R:** You can commit a murder outside, but if you cough like ten metres and people look at you as a criminal.

**I:** Absolutely.

**R:** Coronavirus, I understand little bits and pieces of this is a virus which has spread through droplets. Also another is that life of Coronavirus is around five to seven hours when kept outside and it doesn’t live long. Then when you touch surfaces or any things like even when you buy groceries it is better you keep them away for some time open air for around five hours and then take them and wash and then put in. If you travel outside because of these droplets back to various surfaces. When we touch the surfaces it is better you come back and wash properly. There is a proper proper hand wash and face wash which can be done before touching or entering that house.

**I:** That is the general, the basic idea that we all have now. If I can ask you like in your opinion, how did Coronavirus appear?

**R:** I think where mentioned earlier. Like probably it is from one of the labs in China, Wuhan. That is where a starting point of all of that pandemic that—

**I:** Lab means like you say man made?

**R:** Yes, very much. It’s a laboratory where a lot of viruses were tested and this is again media information, which I have read across. I don’t know. There are various ways. And also like they do say it is through bats.

**I:** Also through bats. Why do you think the people who do research with these in a lab or why did China, what was the objective do you think?

**R:** There are various theories on this and again this is from various—I have acquired this knowledge. Biological warfare is one thing, probably. The spread of this thing. China has, in a way, one economic warfare across the world. The dominance of European and US markets, especially US markets. China wanted that economic high handedness. Probably it is an economic war which uses the rates to buy China across this thing. There are also some theories and some this thing we are in and certain of the countries we are in and there is a few of their **[Unclear]** 16.21 and they would sustain and they were sold out cheap and Chinese brought it over. They are establishing our economic, strong handedness across the world.

**I:** From China like originating in China, how do you think it appeared in the UK?

**R:** People have travelled across from China to various places. For example, there is one called COVID-1 who went to South Korea and then one person was able to spread it. One person went to Mass on a Sunday and into a social gathering pub or club and then visited friends and travelled across and that’s it. It is as simple as that. We didn’t know about it. When we usually talk like this. We cover our house with our hand and then cough without gloves. Probably, you might have touched some surface and he or she would have touched some surfaces and shook some hands with some people. That is more than enough.

**I:** Definitely.

**R:** People travelled across left, right and centre. The spread was unknown. There was somebody who was carrying it. He kept on. He or she kept on spreading it. Nobody knew it. One person gets it and he gets out and then again shakes hands and then touches some surfaces and bought some shopping and it is spread.

**I:** Absolutely.

**R:** The UK especially, with all due respect to what has been done todate. There was no quarantine out the port of entry. That was the major problem. Even until last week, there was no quarantine at the port of entry. People themselves have written on social media as well as observed this and mentioned this on social media and various media that they were like thoroughly checked the temperature was checked and everything was done when they travelled in Dubai. Heathrow it was a free walk out. They just walked out of the airport, no quarantine there. That might have been one of the reasons where in the ports of entry would have carried a few potential Corona carriers.

**I:** Definitely. If I can ask like since the beginning of the pandemic, which is maybe at the beginning like January, February or around that time. Have you a perception about why this has changed?

19.43

**R:** Yes, 100%. As I told you, initially we were only thinking of something called a flu symptom or a **[Unclear]** 19.56. By January, even China did not let out its numbers. By the end of January or somewhere in February they started talking about Wuhan disaster and all that stuff. They kept it a secret.

**I:** That had definitely got your perception about change. Do you think this pandemic is any different from any other we had in the past?

**R:** There was this Spanish Flu. The Spanish are blamed for that. However, it was **[Unclear]** 20.41 spread from Spain. That Spanish Flu was one of the biggest pandemics. Plague was one of the biggest pandemics. All these have happened and people have seen though there. That was much much higher when compared to now. So, as of now, once the medical systems and then knowledge of the people has grown. Those days, when there was plague and Spanish Flu, social media and these media weren’t very powerful. So, the spreading of the news was slow. And by that time people realised they were dead, during those times. As of now, this is, though it is a very serious pandemic and knowledge of the people is more as of now. In that sense, due to the social media and everything, people came to know about it and started taking precautions very soon, very early. So, this was contained in the early stages.

**I:** You think the world has seen heavier pandemic, the more—

**R:** A plague and then Spanish Flu and all that stuff.

**I:** More serious than what is happening now.

**R:** Yes.

**I:** That makes sense, really. What do you think about your government’s response to the pandemic and how the UK government has responded?

**R:** Initially, it was a theory of the realisation which went wrong. The theory of immunisation absolutely went wrong. We fell flat on our face, seriously with that. Thanks to the government and all the economical sitting they realised very fast and then immediately took measures off lockdown, social distancing and thanks to our finance minister who came out with loads of packages to help the people across and how the local governments are helping small businesses and various other businesses to such things in this time.

**I:** So, they have definitely done their bit, you would say?

**R:** Very much. Very much. Thanks to the NHS they have put their lives at stake and worked so very hard during these times.

**I:** Do you think personally, you have got any help from the government?

**R:** Yes.

**I:** You have.

**R:** My local government Croydon Council has helped our business and I am very thankful for them. They came to our rescue because though I eat or not eat, I have to pay my staff. I have to pay their salary. My head chef as well as, though we are a small entity. But, a small entity has its own problems. We have our own problems. We have our own overheads. My landlord said, we will not give a rent holiday. I am paying the rent. I told him, I cannot pay in bulk. I can pay month on month. Month on month I am paying my rent.

**I:** Correct.

**R:** To the landlord.

**I:** Correct.

**R:** So, all this and my overheads. My rent was to just were on CCTV we are on this thing and everything were on. I have to pay those. I have overheads.

**I:** Some help from the government to sustain yourself.

**R:** Yes. We got a good local government. That is good.

**I:** That is very good news. And so, now, what was the information about Coronavirus which has surprised you most?

25.24

**R:** Shaking hands and—

**I:** That is a very good thing.

**R:** The shocking information is right. It is a underlying condition which is not, which sprouts up all of this again. What do you call has a very severe death rate. That is one of the things which is very frightening.

**I:** With the infection going on and with the death rate there is you cannot see a correlation just in your naked eye. So many people can have the infection. You don’t know. You can’t tell.

**R:** Earlier, social distancing and isolation and all are helping now.

**I:** Correct.

**R:** Being safe.

**I:** Yes, you have to be safe. When you discuss about Coronavirus with your family and with your friends, what do you mostly talk about?

**R:** Number one, most important, I don’t spread or discuss news.

**I:** That is a very good idea. Thank you.

**R:** You are terrified—people will be, probably a certain amount of the people would die of eagerness and tension rather than Coronavirus.

**I:** Correct.

**R:** Let us talk about positive things like how to keep safe. Wash your hands. Maintain social distancing. Better not step out only step out if required. These are the things I discuss with my friends and family are our priorities. I don’t talk about like today 800 deaths and 900 deaths, no. I don’t talk about that. I talk about like how we can survive and live happily.

**I:** I think that is the thing we need for the world at the moment. There is too much negativity around. Too much negativity going around.

**R:** I think there are very few things that attracts a lot.

**I:** Absolutely. Though you have discussed that like how your personal life has been affected by the pandemic. Do you want to add anything else in here? Looking at your personal life.

**R:** Personal life, probably where my wife and I, we have more time together.

**I:** That is really positive.

**R:** Positive way of looking at the whole thing. There were a lot of pending things which were supposed to be discussed and we had time to discuss those things. Number two, like I also have time, I also found time to reflect on myself to a certain extent. I was running at least I was able to think peacefully on a positive note. And on the other side like I caught up with all the missing series and Netflix.

**I:** You are binge watching.

**R:** Binging. Seriously, I binged for around two weeks.

**I:** You need that at some time in your life. The world is forcing on you to have some break.

**R:** As I told you, like nature is putting us on our feet now. Nature is putting us on our feet. We are understanding, we are able to understand our ground. We are able to understand how to respect human beings. We are able to understand to respect human life. Respect nature.

**I:** Definitely. Can you share how your daily life goes in the pandemic?

**R:** Initially, it was a regular routine. I cannot sleep after half past six come what may. One of the things were like initially there is like we were cooking various dishes and all that stuff and eating. Then later on, that is it. Life has almost come to a standstill. My friends especially is zero action. My wife was working from home. My wife was working from home and so she used to log in at nine o'clock and work until seven o'clock.

**I:** Eating and enjoying life in some manner.

**R:** In some manner. There was this depression of what you call the business closed and all that stuff. And then enjoying my whisky in the evenings.

**I:** Good that you can still have that.

**R:** I have been seeing videos.

31.19

**I:** How do you think this pandemic will end?

**R:** Normally, it will end on a very positive note wherein the people will probably, I keep my fingers crossed still on this. People haven’t been humbled by end of this pandemic and I am surprised.

**I:** Would you really think so like people will change?

**R:** People will change. For example, this pandemic has given another thing wherein. I stand on my terrace and smoke. Earlier everybody used to run around. Now, we say good morning and good afternoon, good evening. We all have a sense of community. Community bonding. Thursday evenings when everyone comes out onto their balconies and terraces and start clapping, at least people are wishing each other before they are going.

**I:** That is really—

32.25

**R:** I see those old days are the country life are wishing each other. Smiling at each other. Thank god, he’s not smiling—

**I:** Correct. How long do you think that could carry on actually, good or bad?

**R:** Probably we should get used to this now. Probably, as I told you, like we will start respecting human beings. We agreed that they are human beings as human beings.

**I:** That is very much good indeed. Correct. The last question on the first part we have come to the first part end of that. How do you think we might prevent pandemics like this in future? You can’t hear me? I was asking about how do you think we might be able to prevent pandemics in future?

**R:** I think it’s about control. One human being cannot control anything. Every human being being responsible would prevent anything in future. It is not one—as John F Kennedy said, if you want to keep the nation clean, keep your home clean.

**I:** Correct. And wash your hands.

**R:** That is it. Every citizen on the face of the earth if their place is clean, the whole community is clean, the nation is clean and the world is clean.

**I:** That is correct.

**R:** That is what.

**I:** We have conducted the first part, which was basically with your understanding of the world and what the virus is doing to the world. Now, I will come to the second part, in which the focus is on the South Asian community. When we discussed if you can reflect on the experiences of the community as we answer the parts.

34.50

**R:** Before you start recording.

**I:** Let me stop the recording. I will start by asking what do you think are some of the health concerns for people in your community during this pandemic and why?

**R:** One of the major things across the communities here. More in South Asian community because we come from these tropical countries and all of this are lack of sunlight over here. And a Vitamin D deficiency also causes certain of the things like depression like lower immunity and then probably those are one of the reasons.

**I:** Correct.

**R:** Probably that might be one of the reasons. Wherein a pandemic like this would attack a lower immune person faster than others.

**I:** That is a very valid point which I haven’t thought of that way that a sudden change on the weather. What do you think to the people who are born here? Do you think it can affect them in the same manner? They have not seen the change in the weather.

**R:** They also have various things. Again, here, it also depends upon their melanin pigment which is presenting in our skin. The melanin content is very high for us and that’s why we are brown, black, dark or whatever it is. The white skinned people, they do not have much of melanin and there is no requirement of much on that. They are born like through ages and they are brought up here. The requirement of various **[Unclear]** 37.04 compared to us. Vitamin D deficiency, actually is across all the communities. Number two, the kind of food habits. Again, why it is so much the thing is because of heat and eat culture that junk food culture and eating all of these stuff outside has caused obesity, a lack of proper immune system. Third is—

**I:** Do you think if I can just stop you for a moment, do you think it is specific relation to the South Asian community. They have more of this kind of lifestyle?

**R:** No. South Asian community usually have the habit of cooking at home and eating.

**I:** These ones which you mention that it’s across the community or related to the South Asian?

**R:** Across the community. South Asian community see, again, as I told you one of the major reasons could be this. And then there are certain sections wherein unhygienic is one of the problems. I would also be dare enough to say certain things openly wherein. There are certain sets of immigrants, economical **[Unclear]** 38.53. There are certain sets of immigrants who are hoarded into, I am talking not only about South Asian, but various European or whatever it is. Let us concentrate on South Asian. They are hoarded like groups of ten people living in that. Hygienic issues. Hygiene issues. One of those guys working anywhere across would spread it.

**I:** Correct. So that is specific with—

**R:** These are all the things.

**I:** Correct. Do you think the people in the South Asian community are more or less at risk to the pandemic which you obviously mentioned this appalling living condition which can be a risk to their health. Any other issues you can think of?

**R:** Not many. I don’t think many as of now. The communities I am associated with across. I know their lifestyles and everything. It is hygiene. Proper cooked food. Eating outside and eating at proper places rather than going for frozen and fried food. These are all the things. They take care of themselves. A 100% of all the people I know good quality of living.

**I:** If I can ask you to reflect on your family like how it has affected you in your lifestyle or in your health-wise. Your thoughts, your attitude or your behaviour.

**R:** I keep on seeing my family here is my wife and I. They take care as I told you. Think positive and talk positive things and talk good things. When we are together, we will have fun. That’s it. A healthy mind keeps a healthy body.

**I:** Absolutely. Absolutely.

**R:** That is the way it is. Our eating habits are very much traditional and conservative I can say. We love to cook food and eat at home. Me being a chef and my wife does her own thing. She is also a good cook like she cooks at home like we enjoy eating at home. Even that, you will see, when we go out, used to go out earlier and all eat out in various restaurants and everything. We were very selective. Not go for any of those things. We are careful about our eating habits and also we are careful of our thoughts and of our living and all that stuff and so that a balance is maintained. No negative thoughts entering our minds. It’s all talking about good things. We find we are closed today and tomorrow we will open and everything close where life is back to normal. We will have good life. That’s it.

**I:** That is very encouraging. If I now reflect on the government strategies which they have put in like working from home and social distancing and the hygiene, living at home. What do you think these South Asian communities are able to abide by that? Do they have any particular difficulty?

42.56

**R:** Everybody is maintaining—when it comes to life everybody will, what do you call, abide by the rules. You can flaunt rules. It is your life. You are responsible for your own life. So, abide by the rules. What people I have around and all we have our calls and all that stuff. Strict social distancing, everybody. They are very strict about it. They are taking care of themselves. They are taking care of the community. They are going for their short walks with their own family to the park and coming back. Only stepping outside for essential purchases or ordering online. Majority of the families are ordering online. They are even not stepping out. Their understanding and their knowledge about the current crisis is very high.

**I:** Correct. You see that similar pattern of a white British following these rules as compared to [sound goes].

**R:** Can you hear me now?

**I:** I can. I don’t know what is happening. Maybe the signal or something.

**R:** I don't know. It is reconnecting.

**I:** Correct. Looking at the people following the rules, comparing between the white British and the South Asian, do you see any difference or the difficulty they may have in terms of their attitude, behaviour, their approach or do you think they are similar.

**R:** Everybody has calmed down, actually. The hustle and bustle of running around every single day. Even the majority of them are working from home. They have calmed down a lot. I can see that in the people. We have time to at least say hello to each other or phone now. I think it was like people were so busy running around, they never had time to just say hello. I receive calls from a long lost friend and all that stuff who say hello.

**I:** That is connect community.

**R:** That connect community. People have calmed down a lot. They no more bother about okay, let’s do our work and let’s get our family together. Let’s keep safe. Stay healthy.

**I:** To what extent you feel people in the South Asian community are able to access the healthcare facilities in this country.

**R:** Healthcare has been good. All the minor complaints and all that stuff. Every system has its own flaws. There isn’t a meeting called in an ideal world. Idealism is 100% and 100% expectation is not accepted. Nothing can be perfect. There are flaws in the system. There another flaws in various things. For example, our greatest complaint of any UK citizen is about NHS. NHS are the ones who were giving up their life for others.

**I:** You think that South Asian are the same able to access the facilities as with the white people?

**R:** Yes. But the only thing is that certain again there are certain communities who have abused the system also. There is also a lot of abuse of the system. Obviously, people who have abused have reduced the facilities and chances for others.

**I:** When you say other people—

**R:** We have a piece of cake and I want to have almost 90% of it singlehandedly and 10% should be shared amongst others. I cannot do that. I have to have my own little bit. I should be responsible enough. There is a system I cannot abuse that system. I should be responsible enough to think of my next person. Probably now, probably now people should by now people should have understood the value of human life. How to use that system properly and not abuse the system. This is most important. Even now if people are certain other people haven’t realised that, I am sorry. God only should help them. Nobody else.

**I:** If you don’t mind you can skip the question or answer. When you say, other people, what exactly do you mean?

**R:** Pardon?

**I:** When you say other people are abusing the system. Who do you refer to or who do you mean?

48.30

**R:** I do not want to refer to anybody who abuses the system is abusing the system. That’s it. It maybe x, y, z, whoever it is. It would create a controversy or it would be controversial to mention, particular somebody. People who are abusing the system how many of the whole society; that is what I could say.

**I:** That is also helpful like you see the system being not put the use as it should be. Do you think the South Asian people they trust the government that they have made the right choice about the pandemic?

**R:** As I told you, like every system has its own flaws. South Asian people, okay, I cannot be a representative of all the South Asian people. I was an Indian, myself. I had no belief in immunisation. It maybe they have corrected themselves. The system is again government has its own limitations and you cannot with Boris Johnson the Prime Minister doesn’t have a magic wand in his hand. He can just wave it and turn around things. Everything should undergo a process. A single head or a single human being cannot make a decision. He has to be with the Director of Health and various other associated scientific research people, everybody and has to come to a decision. The decisions were made immediately, corrections were made and brought in social distancing was enforced and everything could be applauded. Initially, **[Unclear]** 50.50 to a certain extent. Because, by the time we and the government understood the whole thing, the spread was very high. Now, I read in the news that, even in the port of entries as of now, there are what you call quarantine. There is quarantine done now.

**I:** That is good news, yes.

**R:** The government had expectations of 100,000 test to be done by the end of April and they fail. They are trying their best. Bringing in PPE equipment for NHS workers and other key workers, they weren’t able to do that. It’s a failure. They tried it. They are working on it in various ways. Another bigger thing is the testing part of it. **[Unclear]** 51.58 what he has done. What Richie has done is amazing. The local government they help the small businesses and everybody. That is amazing, actually. Immediately, immediately within seven days the response was there.

**I:** That is good to hear.

**R:** As I told you, there is a positive side. There is a negative side. We all have to look into what is more positive and how to overcome the negatives. The government is nothing but a government is not an entity, it is we. It is us, the whole government. The more the public, the more public support, the more successful is the government. When the government says lockdown and there is some result and people are sitting in the park doing barbecue and then drinking and so how is government responsible to that? It is a social responsibility is most important for a successful government. Government makes rules. Who follows it? We? If we do not follow the rules and blame the government for failure, that is unaccepted.

**I:** So, how much do you think the South Asian people are actually following the rules?

**R:** You see, again, we see okay all you put into only one sector of people, sector of people I know, hundreds of South Asians across Croydon. Everybody is following strictly. They are respecting those. They are only going for essential things or a walk in the morning or in the evening or ordering online and then keeping a safe distance and keeping themselves safe and healthy. That is more than a contribution to the society and more than help to the government.

**I:** Now, surrounding the messages because the government is providing messages to the general public to follow, right? How far do you think these messages are reaching the South Asian community across and also how these messages could be done better to reach across the community.

**R:** As of now, for example, the Prime Minister wrote a personal letter to each and every household in the UK. Everybody received those letters. Number two, the media are helping the government by sending across the message directly to every, it is reaching every home. And then there are social workers as well as Met Police roaming around and then even in the parks and everywhere they are present. They are taking the pain of requesting people believe not to hang around more or there in the parks are in public places. Social distancing at their superstores. All the stores wherein social distancing is strictly maintained. From the government all these rules and regulations have been passed onto various entities and these entities help follow and the general public are following it, that is very nice. What more can we ask for that. What more can we ask.

**I:** Actually, we are at the end of the interview now. I will ask you the final question that, what do you think has helped you and your community to deal with the crisis? Anything in particular?

**R:** I personally, I started a business which closed in just two weeks. There restaurant was closed and all that stuff. Even then like I had to pay my staff from my own personal money and all that stuff. I received a grant from the local government, Croydon Council. I am happy and thankful to the government. They came to my help at the right time and I am really very very thankful to Croydon Council. That is number one and number two. Now, we have restarted our only takeaways and deliveries business from Thursday onwards, last Thursday takeaway. I keep my lights on and then keep the doors secure. Lights on and then the whole board is on. The sign is on outside and all that stuff. Proper social distancing and deliveries and takeaways are happening. That is good. That is one part of it. The way various admissions by government who help keeping the whole community safe has helped. It’s as simple as this, whoever you are, South Asian, European or African or any Americans or anybody are the localites who live here, English. It’s only as simple as like we follow the law of the land. We are safe. Our community is safe and the government is also functioning happily for us. Whilst we support the whole system, the system cannot support us. I cannot only accept things. I also have to give back something to the society. That is called social responsibility or society responsibility. That is where the whole community or the whole country will be successful.

**I:** Do you see any particular aspect of your community? Is there any aspect of the community which has sustained the crisis with the reference to the South Asian people?

**R:** Yes, following the rules strictly and then social distancing and keeping themselves safe and healthy. That’s it. Every home keeps itself safe and healthy. The whole of society is safe and healthy. That’s it.

**I:** It was great talking to you again. We are at the end of our interview. Do you want to add anything to wrap or you have, you think you have missed anything? You want to comment?

**R:** No, nothing. I think we have discussed absolutely every nook and corner of this. And probably I think like I have replied satisfactorily. Put my opinions properly across.

**I:** Absolutely. There is no right or wrong answer. It is just your perception.

**R:** It is my perception. I own every word what I say.

**I:** That is good to hear. I am like pausing my recording now. Thank you very much again.

**R:** Thank you very much.

END OF INTERVIEW – 60 mins

Transcribed by Linda Pitt. Email: linda@laptopconfidential.com or laptop.confidential@btinternet.com - Telephone: 01964 612088
