# Supplementary material for: Mitigating the identity and health threat of COVID-19: Perspectives of middle-class South Asians living in the UK
Source: J Health Psychol. 2021 Jun 22;27(9):2147–60. doi: 10.1177/13591053211027626 (PMC9353968; doi:10.1177/13591053211027626)
Supplement: sj-docx-8-hpq-10.1177_13591053211027626 – for Mitigating the identity and health threat of COVID-19: Perspectives of middle-class South Asians living in the UK [file sj-docx-8-hpq-10.1177_13591053211027626.docx]

**UK5-GroupB-MMI-May20**

Transcribed by Sharmistha Chaudhuri

(55 minutes)

**Part 1**

Researcher: Thank you again for participating in our study.

Participant: Yeah.

Researcher: Let me start with the question. Like, what do you think is happening to the world now?

Participant: Um, I think it's, in a state of flux, lot of confusion, a lot of, um, damage economically, psychologically, confusion, lot of uncertainties, everywhere, in economy, employment, profession, everything, you know, to some extent, I think one has come to a metaphorical world, it's come to a standstill.

Researcher: That's uh, everything is uncertain stand still. And what would you see? What comes to your mind when you think of coronavirus?

Participant: Um, uh, maybe not the appropriate answer, but first China. You know when you ask the make rapid, rapid round questions, it's China. And then the dangers, the implication of the way the virus probably is spreading and your probability of catching it.

Researcher: So why China? I mean, it's very apparent, but, but I would like to hear from you.

Participant: Because I think that, I follow, this is very personal, I follow a lot of news on that, and that caused me to believe that there could be some Chinese involvement in political front. I mean, it's not, not just a virus.

Researcher: Got it. So you think the Chinese authority has something to do with this?

Participant: Yeah, in some way or another, you know.

Researcher: Correct. And, how do you think this virus has affected the people in general? The life of the world?

Participant ([02:18](https://www.temi.com/editor/t/KcgZ_2A4vCMklGY9M1NBRv3G9oOVdiURt90xz6t2qCjPFBO_lmn0ap1j0zkUcnYwrL5z7jiw4LyWn8JliEppkS2ad_E?loadFrom=SharedLink&ts=138.77)): Well, certainly it's primarily frightened them. Uh, it has frightened them and it has certainly made people confused and lost confidence as well. I believe so, you know. People are scared, people are confused because they don't know what to do. There are basic problems, like, um, day to day expenses. What are they going to get it from? Salary, continuation of employment, particularly in the hospitality industry and the retail industry.

Researcher ([03:02](https://www.temi.com/editor/t/KcgZ_2A4vCMklGY9M1NBRv3G9oOVdiURt90xz6t2qCjPFBO_lmn0ap1j0zkUcnYwrL5z7jiw4LyWn8JliEppkS2ad_E?loadFrom=SharedLink&ts=182.13)):So do you see any positive side as well?

Participant ([03:11](https://www.temi.com/editor/t/KcgZ_2A4vCMklGY9M1NBRv3G9oOVdiURt90xz6t2qCjPFBO_lmn0ap1j0zkUcnYwrL5z7jiw4LyWn8JliEppkS2ad_E?loadFrom=SharedLink&ts=191.64)): Uh, consequences of the virus? Um, I think, yeah, I think it's made us, I think it's made us realize the core values of life, you know. The emotional, um, being inside, maybe appreciate our value, our family and friends, value of time, you know. Misused, in my opinion, the value of times, the value time as such, being together. But I think we've also realized that there are a lots of things in life, which we should be doing, or we should not be doing. By doing or not doing, the world doesn't stop. By not having a KFC, you are not going to die. The last six weeks, we certainly haven't had the KFC, I am not talking about myself, as I do not have it anyway. But because of that our quality of life has not changed as such. In fact, it's better, it is eating much better food and less food as such, we're not overeating, you know.

Researcher: Definitely. And how have you learned about coronavirus? Like, do you remember what are the media sources you rely on or where do you check for updates?

Participant: Well, primarily, the first source of information was, uh, the news and then, uh, continued update was from the television media, the print, not in print, but the online news media. Lots of, uh, information started coming through WhatsApp groups and WhatsApp friends like that. after a while, I actually started ignoring that information, primarily because I found out that some of it was not accurate or fake as well.

Researcher: So in, in the initial phase you did have some news from the WhatsApp, but later on you didn't. Is that correct?

Participant: Yeah. I mean, I still get it, but I don't look at it. I'll just skip it, you know, because I'm still in the groups because we got the messages.

Researcher: Hmm. So do you take any regular updates? Um, on a news daily?

Participant: A lot from, I don't watch TV much. I used to, but not for this coronavirus, because I think it's full of negative news. There's a lot of good things going on, which is not being highlighted. But I do one thing on a daily basis. I go on the BBC online news site and I primarily just google coronavirus in your area, but it's not because of my area, but I don't actually look at my area. I have a very nice, uh, maybe not accurate, but for me it's good enough information, really nice presentation of, uh, the daily progress in a graphical format, info graphs you know. I look at those drafts and I follow those graphs.

Researcher: Right. And from all the information you get around, like from media, et cetera, what can you tell us about coronavirus? Like technically?

Participant ([06:42](https://www.temi.com/editor/t/KcgZ_2A4vCMklGY9M1NBRv3G9oOVdiURt90xz6t2qCjPFBO_lmn0ap1j0zkUcnYwrL5z7jiw4LyWn8JliEppkS2ad_E?loadFrom=SharedLink&ts=402.82)):Well, I know that, um, there's a lot to tell, but what comes to mind is that, um, it's, uh, but the impact of coronavirus is global. Uh, but impact is a loss of in the sense, but we've never seen like this in living history, talking about maybe about a hundred years. You know, I think they're, they're probably likely, saying in a different way, uh, this, but impact of this virus is greater than the great depression you know. The impact, global, uh, I think also the reality is that we are not going to come out of it tomorrow, no antidotes or vaccines or anything like that. But working on the effects or the proportions will continue for maybe many months afterwards. But I think it destroyed, uh, being a professional. I think it's destroyed a lot of economic opportunities, uh, bringing back to the same to many many years, you know.

Researcher ([08:08](https://www.temi.com/editor/t/KcgZ_2A4vCMklGY9M1NBRv3G9oOVdiURt90xz6t2qCjPFBO_lmn0ap1j0zkUcnYwrL5z7jiw4LyWn8JliEppkS2ad_E?loadFrom=SharedLink&ts=488.05)):And how do you think this coronavirus, biologically spreads or effects the human body?

Participant: What, from, from what I've read on the internet and on the tv, and we're excluding WhatsApp, we're not talking that, but of intelligent sources or informed sources. I know that it's not, uh, a bacteria, this is what I know, yeah. It's not a bacteria, a flu bacteria; it is an enzyme, it's not a living thing. I know it's not, uh, uh, transmitted, uh, in the normal sense as it is used to, you know, but it spreads through touch.

Researcher: Yes. And, um, what can you say in your opinion, how did the coronavirus appear in the planet? How did it happen?

Participant: I, I think that, there are two reasons that I'm looking at, one, it is a live specimen, that it started in the market due to hygiene issues, in China. I think it is from a bat to a fish to a bird...and I can't remember the cycle, but something like that. yeah. That was the given explanation. To some extent, I also think, and I, we were talking about this yesterday with my wife, probably, maybe China has a plan which may have gone wrong, politically, to a sort of setback. This is political world, world power struggle type of things. After this trade war with China needed to a lesson type of thing. So I think there are two things. One day could be the natural cycle of the infection moving through from the bat, with the fish to the bird. And I think that there is a political, maybe not.

Researcher: So when do you say China, what's wrong, what do you want to say?

Participant: Well, I think I was reading again for educate myself a little, this, this, the first theory of it going from the back of the fish to the birds. China has some economical problems and these exotic, exotic delicacies, you know, from the bat to pangolin, but they didn't maintain the controls, you know, in terms of hygiene on that. Maybe they have got that wrong because doing all that, we will have an implication on their employment and the economic, you know, mass employment. They overlooked that, to keep people employed. They sort of just said ok, carry on. Um, and I think in terms of actual virus, maybe they didn't... and I don't know this, this is news information. Yeah. They didn't come clean early enough. Of course they said they did, but no I'm in being, you know, it is a big blame game going on. And the person who is most ignorant in this world with this thing, It's not the politicians or the bureaucrats. It's actually the mass public they don't know what's going on because they get confusing messages. So maybe they didn't disclose it early enough.

Researcher: Yes. That can certainly be one of the reasons. And how do you think in it appeared in UK? It's very obvious answer, but what is your perception?

Participant: I think mostly through travel isn't it? People mobility.

Researcher: Hmm. So why, why do you think UK has been impacted to that level?

Participant ([12:42](https://www.temi.com/editor/t/KcgZ_2A4vCMklGY9M1NBRv3G9oOVdiURt90xz6t2qCjPFBO_lmn0ap1j0zkUcnYwrL5z7jiw4LyWn8JliEppkS2ad_E?loadFrom=SharedLink&ts=762.42)): To the level, I mean, of course it's greater than many other countries, partly maybe the country, the government was hoping for something to happen. Maybe they were waiting for some information or triggers or whatever, which didn't happen. So maybe they, I don't think they delayed the action deliberately. They probably got delayed because they were hoping for something to happen. And maybe it didn't happen and the cases rose. But I think also there's an issue about in the beginning, maybe not now, there was an issue about the seriousness of social takings. You're not taking the social distancing, a stipulation serious, people weren't committed to it. You know, I remember that I think on a Friday or someday, I cannot say exactly Boris Johnson came onto the briefing and said, this is what we want people to do. Then few days later, I think they realized that the people are not abiding by.

Do you remember this piece about people going to parks and people going to the seaside or the farm or something? Yeah. Then he, on a Sunday night or Monday night, I think 23rd of March, I'm not sure. He came on TV and said, ok, this is it. You have to stay in. So maybe there wasn't enough, uh, uh, commitment from the public at the moment, but the government stepped in. So that the, may have delayed the lockdown. So Boris Johnson was probably hoping, and we would take on what he is saying, and would take it seriously, but they didn't. But of course now, now it's different.

Researcher: So when you say government was wanting something to happen, what do you think that would have happened or what they thought would have happened?

Participant: Well, maybe they didn't anticipate that the spread would be so, so severe, you know? Yes. Yeah. They, maybe they didn't anticipate that, it's in China. now there were cases in say Italy and Spain. If I remember correctly, the Italian cases, in the north, were mostly confined to the north, you know, kind of. There were some people going to China then coming back, there was a group of people, you know, something, this was a huge, bigger trigger than normal.

Researcher: People traveling from China. Yes.

Participant: Yeah. Yeah. There was some students trips, few student groups. Yeah.

Researcher: And now something to reflect on like, since the beginning of the pandemic, have your perception of the corona virus changed?

Participant: So the extent that I think it's created more fear in the beginning, I used to think and argue the fact, you know, the common again, this is the information being fed to us by, more people die from flu, and this can't be that serious , kind of. Now I think, yeah, definitely. I am actually scared not to the extent that I'm shaking every day, but every now and then you have this thought, what if you're one of the victims. Definitely. Yeah.

Researcher: In the beginning, you thought that it may not be that serious.

Participant ([16:26](https://www.temi.com/editor/t/KcgZ_2A4vCMklGY9M1NBRv3G9oOVdiURt90xz6t2qCjPFBO_lmn0ap1j0zkUcnYwrL5z7jiw4LyWn8JliEppkS2ad_E?loadFrom=SharedLink&ts=986.77)): Yeah. In, in terms of my, uh, view on it. But in terms of our, uh, our proportions for the guidelines, we were fine. We were following. But to the best of our ability. Now I think like many people I haven't been out of the house of course, for other than a walk, but for meetings or, or shopping or anything for 5 weeks now.

Researcher ([16:53](https://www.temi.com/editor/t/KcgZ_2A4vCMklGY9M1NBRv3G9oOVdiURt90xz6t2qCjPFBO_lmn0ap1j0zkUcnYwrL5z7jiw4LyWn8JliEppkS2ad_E?loadFrom=SharedLink&ts=1013.92)): So what made you have this later feeling of fear, that you got now?

Participant ([17:00](https://www.temi.com/editor/t/KcgZ_2A4vCMklGY9M1NBRv3G9oOVdiURt90xz6t2qCjPFBO_lmn0ap1j0zkUcnYwrL5z7jiw4LyWn8JliEppkS2ad_E?loadFrom=SharedLink&ts=1020.28)): Well, I think it's more closer to home now. Isn't it, Midlands. Although it's two regions together, East and West, it's a sizeable area, but, uh, uh, it's the second, second largest area, which is, uh, effected by coronavirus. So that's what I'm I'm I have one the underlined condition, I am diabetic, as I said, what it does, it makes you be a little bit more aware and vigilante.

Researcher: And do you think this pandemic is any different from those which happened in the past?

Participant: No, not really, because I remember following Ebola as well, you know, the only difference is we were not so worried about Ebola, because it wasn't on the doorstep. It was confined mostly in Africa, you know. And I realised it, and I'm sure those people must have gone through the same thing there, but it wasn't on our doorstep. It was just a news item, but this is on our door steps. So I'm not sure it's not much different, but the only difference is that we are experiencing it.

Researcher: Yes. So your perception, like you would have only read it in news report about Ebola.

Participant: Previously. Yeah. From what I remember, um, this is the first pandemic that I remember. I don't remember any of it. You know, there may have been, but I don't physically remember it.

Researcher: Well, we, we wouldn't because it's generations back.

Participant: In the sixties or seventies, they may have been, maybe not pandemics, but a smaller version, not serious enough. Um, but I, I do not remember anything. This is only two I remember, I remember E-coli, I remember Ebola in Africa and that was recent, maybe about five, 10 years ago. And this pandemic.

Researcher: So this is something really novel for you to experience, right. And, how would you say though, you have mentioned something already, but uh, how would you think UK government has responded to this crisis?

Participant: Uh, I think it's, not easy. It's like managing a house. It's also has to do with budgets and abilities, you know? So, I mean, I have a different view on this. Because generally people think they haven't managed very well, but they think, you know, there's no person on earth unless probably there's some authoritarian people, you know, who would want to manage their own, uh, uh, position, particularly in a party. I think they mean it, and they have managed to the best of their ability, they can, whatever they thought was the correct decision. Now people will have different views because, you know, it's not easy. For example, the chancellor said set aside the helmets, 35 million or 350 million or something. I can't remember. Well, that's not a small amount, you know, and you have to think where that money is going to come from. So they have big decisions to make and where to spend that. Then also the fact that all this is quite sudden, they weren't ready for this, you know. None of the governments were ready. off the record for me, maybe the Chinese were, but nobody else was. Uh, so I, I personally think they're handling it in the best of their ability. I'm not saying that it's the best way to handle it, but I think they are handling it in the best way they can. And what I really appreciate, because this is me. I appreciate the fact, not that I watched it, but I appreciate the fact that they're making efforts to do a daily update. I think it is important to keep people informed, or at least will make the effort to keep them informed.

Researcher ([21:32](https://www.temi.com/editor/t/KcgZ_2A4vCMklGY9M1NBRv3G9oOVdiURt90xz6t2qCjPFBO_lmn0ap1j0zkUcnYwrL5z7jiw4LyWn8JliEppkS2ad_E?loadFrom=SharedLink&ts=1292.73)):Now coming back to that, Corona, is there any information about it which surprises you?

Participant ([21:44](https://www.temi.com/editor/t/KcgZ_2A4vCMklGY9M1NBRv3G9oOVdiURt90xz6t2qCjPFBO_lmn0ap1j0zkUcnYwrL5z7jiw4LyWn8JliEppkS2ad_E?loadFrom=SharedLink&ts=1304.82)):Uh, in what perspective?

Researcher : I mean, means like you would not have expected to happen with the virus.

Participant: The number of deaths, you know. The number of people that have been infected, I wouldn't have expected that many, for example, the numbers are huge. Yeah.

Researcher: Or are there also any information about coronavirus you would not expect?

Participant: No, not really, because I don't even know the subject. I'm not a medical doctor, so I wouldn't know about it, but the information I have is what I've been treated by the professionals.

Researcher: Correct. And that's the number of deaths which has really taken you by surprise.

Participant: Yeah.

Researcher: What would you say, like when you discuss, about the virus with other people, with your friends, family, neighbours, what would you mainly discuss about?

Participant: Well, generally about how it's spreading or how effective, how much it's effecting people and dangers, et cetera. But the thing I mostly emphasize on is follow the rules. You don't follow the government's guidelines, do what you need to do to keep safe, you know, don't try to be too clever. And I usually say, don't be an Indian in this you know...[it should be fine] (translated), that type of. If you have to do something, do something, don't be silly about it. Do you know what I mean? Be responsible. Yeah.

Researcher: So take responsibility about you.

Participant: On your individual level. You don't have to go out and become mother Theresa for anybody. You just look up to yourself. If everybody does that, then everything will be fine. It is what it is. We have to get out of it. That's it. There's no point arguing about it. There's no point blaming China, which we should, but still, no point. We really need to look forward and move forward because I think there are bigger implications to worry about not just the virus and its effects, you know. The number of people who are going to be out of employment, et cetera, et cetera.

Researcher: And, um, can you tell us how your personal life has been affected by this virus?

Participant: Quite substantially. I am under house arrest, yeah? Of course the safety of myself, nobody else's. Yeah. The amount of things that we can do is restricted, but the biggest thing I realized is that we do a lot of things, which we did, which are not necessarily things. We don't do things which are necessary. So in my case, I, I've started gradually. I've always, valuing time and much more. What I do with the time.

Researcher ([24:55](https://www.temi.com/editor/t/KcgZ_2A4vCMklGY9M1NBRv3G9oOVdiURt90xz6t2qCjPFBO_lmn0ap1j0zkUcnYwrL5z7jiw4LyWn8JliEppkS2ad_E?loadFrom=SharedLink&ts=1495.72)):Can you give us some examples?

Participant ([24:59](https://www.temi.com/editor/t/KcgZ_2A4vCMklGY9M1NBRv3G9oOVdiURt90xz6t2qCjPFBO_lmn0ap1j0zkUcnYwrL5z7jiw4LyWn8JliEppkS2ad_E?loadFrom=SharedLink&ts=1499.74)): Um, I can…right. What I've done is, we'll see if it is, but I'll give you two examples. One, I started using time available. Now you may think this is recreation but it's not, which I was putting it off. So there were certain things I wanted to learn. So I've actually started completing programs in Udemy, the online courses you know. So you learn, uh, what's the, uh, expanding my skills or increasing my skill.

Researcher: So have you been able to finish some ?

Participant ([25:37](https://www.temi.com/editor/t/KcgZ_2A4vCMklGY9M1NBRv3G9oOVdiURt90xz6t2qCjPFBO_lmn0ap1j0zkUcnYwrL5z7jiw4LyWn8JliEppkS2ad_E?loadFrom=SharedLink&ts=1537.75)): Yeah. Professional courses, learning, learning more basically.

Researcher :So using that time to learn, to do fill up the time. Yes. That's really good.

Participant: Learning to make all that academic stuff, you know.

Researcher: So that you would say as a positive effect in your life.

Participant: Yeah, definitely. And the second thing is, uh, for years, and this may not be relevant, but I'm just because it's relevant, to the extent you realize it. I have been trying to get into the habit of, uh, doing some sort of a meditation practice for years and I haven't been able to. Now last month or so, I've actually been doing meditation for one hour every night, yeah. So that, to me, how it changed my life, you know.

Researcher: Lot of like you would say, peace?

Participant: Yeah, yeah. It's making me do things which I value. It will not be valuable to somebody else, but it is for me.

Researcher: Okay. So being a little personal here, um, can you share your..

Participant: I can't tell you for my other children in the home, my other friend's girlfriend, I'm sorry (laughs).I can't share that here because we're recording.

Researcher: That we can take offline (laughs). Um, few sentences on how is your everyday daily life now?

Participant: Okay. So I don't wake up as early as I used to. So I wake up a little later is, I mean, in general terms, I'm not going to mention hour by hour, yeah?

Researcher: No, no, just overall.

Participant: Yeah. So, but then I go to sleep early. Usually I went to sleep at about one o'clock. Now it is 2, 2:30, et cetera. Remember I've added an hour in my activity for the meditation. Yeah. So, um, I, I do things in a less rushed way, you know. Yes. Because I have time. Yeah. Yeah. I would travel. Travel has gotten down quite substantially.

Researcher ([28:09](https://www.temi.com/editor/t/KcgZ_2A4vCMklGY9M1NBRv3G9oOVdiURt90xz6t2qCjPFBO_lmn0ap1j0zkUcnYwrL5z7jiw4LyWn8JliEppkS2ad_E?loadFrom=SharedLink&ts=1689.71)): And any other like, uh, at ending to your health, to the house chores.

Participant ([28:15](https://www.temi.com/editor/t/KcgZ_2A4vCMklGY9M1NBRv3G9oOVdiURt90xz6t2qCjPFBO_lmn0ap1j0zkUcnYwrL5z7jiw4LyWn8JliEppkS2ad_E?loadFrom=SharedLink&ts=1695.89)):That's fine. I mean that doesn't change, that's routine and that's carrying on, you know, but in terms of the rat race type of thing, it's a little bit more lesser and relaxed, and as a consequence is, and maybe this is not relevant to me, you're asking, that's why I'm saying the consequences of this more widely meditation in the night. I’ve actually mentally, spontaneously started evaluating what is valuable and what is not valuable. And I can see that, that lot other things which are not valuable. But I think this, this, uh, discrimination, this analysis is going for many people's mind, not everybody, but this and this pandemic has caused them to think I'm using life. You know, definitely use the meditation is probably a little bit more.

Researcher ([29:21](https://www.temi.com/editor/t/KcgZ_2A4vCMklGY9M1NBRv3G9oOVdiURt90xz6t2qCjPFBO_lmn0ap1j0zkUcnYwrL5z7jiw4LyWn8JliEppkS2ad_E?loadFrom=SharedLink&ts=1761.41)): Definitely. And now difficult question. How do you think this pandemic is going to end? How do you think this pandemic would end?

Participant: I, I, I mean, in terms of, uh, would end, I think it's going to be a disaster. There's nothing positive in this except to find the qualities of life. Yeah. That's coming out. But in terms of economics and politics, food, availability pf food, it is going to be a huge challenge. And that's one of my worries, you know?

Researcher: So how long, like, do you have any time period in your mind and can see this coming to an end?

Participant: I think we are talking about years not months to come back to normality. And part of the reason is I think that economics, economics of it all has been, not quite.. For example, I was talking to my colleagues in Spain the other day, and she was saying, you know, 40% of Spain's working population is going to be unemployed, which is, you're not even doing the 2008 crash, economical crash. They had the total unemployment population of about 20%. 40% is a hundred percent increase on that. And the reason for that in Spain is because in Spain, the industry is mostly tourism and hospitality, but I can see that happening here as well in England, a lot of people in the retail industry, a lot of people in the hospitality industry, local restaurants, coffee shop, are going to be. But I also fear one more thing, especially for our Asian or, uh, people who depend on specific type of food. If you look at Indian, Indian vegetables, overnight, there is sharp increase in price, what we were buying four pounds a kilo has become 10 pounds a kilo.

Researcher: Why do you think is that price please? Who has done it?

Participant: But part of it is just opportunities. Opportunities. They've explained that it's is lack of lack of availability. I don't think so. No. I have not found the government saying that we are going to increase the duty or import tax to anyone. Can you imagine something that was costing four pounds, a kilo is now 10 pounds a kilo or more. Cost of living is going to be substantially high.

Researcher: Right now we are at the end of the section one. And we'll finish with the last question by asking you that, how do you think we can prevent pandemic like this in future?

Participant ([32:35](https://www.temi.com/editor/t/KcgZ_2A4vCMklGY9M1NBRv3G9oOVdiURt90xz6t2qCjPFBO_lmn0ap1j0zkUcnYwrL5z7jiw4LyWn8JliEppkS2ad_E?loadFrom=SharedLink&ts=1955.34)): Yeah, well, sadly, um, we don't listen to ourselves very often. Some of the things that has come to me through WhatsApp and through news media is some historical recordings of presidents, prime ministers, scientists, et cetera, or have actually said, we need to be prepared for this kind of things some years ago, but nobody actually took any heed of it. Uh, I remember a clip seeing president Obama saying that the next war is not a weapon war, it's going to be germ war and we need to be, I mean, he actually said in that clip that we need to be prepared with medicine, the hospital, the medical staff etc., You know, but the budget wasn't allocated. So I think we need to take seriously and foresee the future, uh, scientifically, uh, realizing that political warfare and political matters are not going to be fought with guns and ammunition. It's probably something like this, you know?

Researcher: So would you think that these pandemic will give us some lesson to take and help us to prevent any future event like that?

Participant: Well, I think it has woken the world, the political leaders for the fact that they should now prepare for a different type of challenges. I mean, maybe this is a war by China to set the world economy back, you know. Because there was a pretext to this. It wasn't that the trade war, in which China was going to sort of teach a lesson to few people, some lessons in some ways. It’s a very black and white opinion, but there are, there are reasons to think like that. So I think it has woken the world. It has woken the order of, to the fact that there are different types of challenges in terms of political rivals, you know, but it's also maybe give an opportunity to other people to come out, come out as leaders. Unknown people.

**Part II**

Researcher: Okay. So we have finished the first part of our interview, which is interesting. So we have done half and I would move on to the part two, which is basically based on the South Asian community and your perception of how it has affected them. So the first question I would ask is what do you think, um, that, what are the major health concerns in the South Asian community, which might have affected them more or less with the corona virus?

Participant ([35:38](https://www.temi.com/editor/t/KcgZ_2A4vCMklGY9M1NBRv3G9oOVdiURt90xz6t2qCjPFBO_lmn0ap1j0zkUcnYwrL5z7jiw4LyWn8JliEppkS2ad_E?loadFrom=SharedLink&ts=2138.55)):I think, are you talking about South Asians community in UK or globally?

Researcher is the focus, but you can also talk about the global perspective.

Participant I think maybe lifestyle, due to lifestyle and underlying health conditions, the lack of immunity, that would be one. For example, many South Asian communities are obese people, compared to a general public, you know, then you've got the underlying conditions like diabetes or blood pressure or whatever. Yeah. Be, uh, uh, Oh yeah. And also things like vitamin D deficiencies.

Researcher: Right. So do you think vit D deficiencies are more in the Asian people?

Participant :It's a known fact, isn't it? That the vitamin D deficiency is quite prominent and pronounced in Indian subcontinent.

Researcher: Do you think it has any, any further implication because UK gets less Sun for the matter?

Participant ([36:56](https://www.temi.com/editor/t/KcgZ_2A4vCMklGY9M1NBRv3G9oOVdiURt90xz6t2qCjPFBO_lmn0ap1j0zkUcnYwrL5z7jiw4LyWn8JliEppkS2ad_E?loadFrom=SharedLink&ts=2216.15)):Possibly. Possibly. I am not in a position to make a comment. But what I do know is that I comparatively, we suffer more and that's the data that's coming out and I can understand because we've got certain disadvantages in terms of our, immune system because of our underlying conditions, like overcoming the vit D deficiency. Maybe, our lifestyle, in terms of the food we eat, etc., but I am not sure about it. We need to accept, but I'm not sure about that. But also I think people like me, I've been here for 51 years. So I think our food habits are much better than many people, who've just come from India, maybe five years ago. We don't, we hardly eat fried stuff or things like that.

Researcher ([37:43](https://www.temi.com/editor/t/KcgZ_2A4vCMklGY9M1NBRv3G9oOVdiURt90xz6t2qCjPFBO_lmn0ap1j0zkUcnYwrL5z7jiw4LyWn8JliEppkS2ad_E?loadFrom=SharedLink&ts=2263.46)): Right. So how do you think they are different? Like with any other people who has recently come?

Participant : Well, I think our, our food, I am not the person, but our generation's food is much more healthier. Food is much more healthier than people who recently arrived from India, for example. Yeah. Maybe the cooking methodology is better as well. The methodology that the new methodology is cooking in less oil or steam food or whatever, you know.

Researcher: Right, right. And, um, how do you think your community, the, like the South Asians has been specifically affected by the virus?

Participant: I think to some extent there has been dis-integration of community, partly because of closure of the most of the community, they approach the community centres, you know, I see, for example, temples or community, they have a model of having all these premises, like the Mistris, Patels, Lohanas you know, but all of those places have closed down. Yeah.

Researcher: Has it affected you personally? Like you, uh, have been going to any community.

Participant: No, but I think those, I mean, of course I am maintaining the social distancing and that, but cases like this, closed community centres, they play a big role in scaffolding intelligent information, particularly for the elderly population, who don't have a good grasp of, uh, English, you know, who watch media, but may not digest them totally. So the leaders from their class has a role to play, but that has been cut off because the community centres are closed now.

Researcher: Mm. And how would you say it has affected South Asian people, professionally, anything different that you see than the general British population?

Participant: No, I think we're all in the same boat. And I don't think for the Asian population as such is singled out for have been effected more in terms of profession. Yes. In terms of health, because of underlying conditions, maybe some, but not professionally. I think, that all my son, myself, my daughter we all are all work from home basis, as my neighbours are, another people who are patients, you know, we are, we are treated in the same manner.

Researcher: How do you think your family has been affected by the virus? Putting everything in together?

Participant: Well, I think some of it is about taking responsibility. For example, my daughter has put a block to me going out altogether. So simple things like that. You've got diabetes, so you're not allowed to go out. Anything you need, you tell me and I'll go and do the shopping. So in that sense, things have changed. Um, nothing has been different. You see, we have a son who is in London, he would come to us every month or so, but it's worked out better that now we have a video call every day, before we didn't.

Researcher ([41:34](https://www.temi.com/editor/t/KcgZ_2A4vCMklGY9M1NBRv3G9oOVdiURt90xz6t2qCjPFBO_lmn0ap1j0zkUcnYwrL5z7jiw4LyWn8JliEppkS2ad_E?loadFrom=SharedLink&ts=2494.01)):That's something positive, you may keep it up!

Participant ([41:37](https://www.temi.com/editor/t/KcgZ_2A4vCMklGY9M1NBRv3G9oOVdiURt90xz6t2qCjPFBO_lmn0ap1j0zkUcnYwrL5z7jiw4LyWn8JliEppkS2ad_E?loadFrom=SharedLink&ts=2497.69)):He would call twice as a week, or three times a week, every other day. But now he's calling, uh, via video call every day. And the nice thing is that he calls in the evening when it's cooking and doing things like that. So we can see that he's actually living a normal life as opposed to a very stressed professional life. You know?

Researcher ([42:00](https://www.temi.com/editor/t/KcgZ_2A4vCMklGY9M1NBRv3G9oOVdiURt90xz6t2qCjPFBO_lmn0ap1j0zkUcnYwrL5z7jiw4LyWn8JliEppkS2ad_E?loadFrom=SharedLink&ts=2520.31)):So that's something very positive.

Participant : No, absolutely. I appreciate it. That, and I'm sure that is the case with many people.

Researcher :Yeah. And, uh, like the government measures, talking about the government measures, like social distancing about the message, what they are communicating about, work from home, et cetera. How do you think the South Asian community is coping with that?

Participant: Generally? I think very well. There are, there's always a few people. We go for a walk every day because the area I live in, it's not predominantly South Asians, but there are sizable numbers. They are not sort of distancing themselves. They're not thinking that when you're, when you're sort of crossing paths, instead of moving sideways- they're not doing that kind of thing, but they are still wearing masks. They're only coming out in couples instead of 10 people at a time, except for that you know. To be fair, I think they're following, Uh, the government's guidelines fairly, fairly accurately. When I go to the shops also, which, kind of, we do normally, but the other day I had to, I had an occasion to drive my wife to the shop, and when I stopped the car, but I looked out, I was watching people going into the shop and they were actually taking elderly people. They were making the effort, which normally they wouldn't, they would just walk into the shop, making effort to check whether there were many people in the shop or not some of them wearing masks as well. And they weren't gathering and chatting or anything. They were going in the shop, doing the shopping and going back to home you know.

Researcher: Hmm. So do you think that the South Asian people are confronting the crisis with any other different attitude, belief or anything which is different from the white people? White, British?

Participant: No, I don't think so. No.

Researcher: The same. You see the response.

Participant: Yeah, generally. I mean, as I said, you'll get some idiots, you know what? They are too knowledgeable for their own good or they are filthy rich people or whatever, they are in the business of their own world, but excluding that if you talk about the masses, no.

Researcher: Mm, no. You see the same pattern within that.

Participant ([44:21](https://www.temi.com/editor/t/KcgZ_2A4vCMklGY9M1NBRv3G9oOVdiURt90xz6t2qCjPFBO_lmn0ap1j0zkUcnYwrL5z7jiw4LyWn8JliEppkS2ad_E?loadFrom=SharedLink&ts=2661.88)):Yeah. I Mean, it's the same awareness. Just a personal example, nothing. Just to give you an idea. My, my brother's father in law passed away about three weeks ago. He was in a home now here's mother-in-law was in a, in a house and father-in-law's was 85, and she was about the she's about 80, but she was aware and having lived in UK for many years by watching the news, the reason what's happening and whatever. And she didn't tell even her own children to come in during this mourning period, except for her son. So what I'm saying is that even that generation, we may not realize, but they are aware of. I'm saying is that you don't,.. that generation, we may not realize, but they are aware and they're taking that on precautions.

Researcher (46:00) Changing your attitude and changing your lifestyle to suit that.

Participant ([46:08](https://www.temi.com/editor/t/Qxe1yjOtWj4mwEjv3chjjwiyV6DtJjKGjeSmYG0OHmk4UaMfVnPgipRbdjGKmUXZiFy3l2dP_VY0GvQyP1TFCq5WG18?loadFrom=SharedLink&ts=68.34)): Being aware, being conscientious because of the fact that you should be doing. But you're not like, for example, if, if somebody goes, Oh, you must have come in because of this, you know, they may not understand the finer complications, but they do understand enough to take precautions.

Researcher: And how would you think the South Asian people have access to the healthcare facility and how easily or they have any difficulty to access them?

Participant: Well, I, I think that the health care access system is guided by the government in any case. So I know a lot of doctors and doctor friends, and I know from them, that they are doing what everybody else is doing. So I don't personally see, uh, any difference for the South Asian negative or positive, why will it be? For example, I have to order my medicine every month but I don’t do it now because I think there's a new rule which came out about two months ago, I do online prescription. And I'm assuming everybody else has the same level of access and I did not have a problem. They were ready, within three days, they've had it ready.

Researcher: That's something positive about how the system is working.

Participant: Things like, for example, the other day, I had a slight blood pressure issue. Notwithstanding that once a year I do my blood pressure measurement. The other day they fixed it. They said, if you have a machine at home, please check, and put it online, I haven't done it yet, but there's an online, uh, reporting system where I can check my blood pressure and give the results to them. I would think they're sending out the same information to everybody else whether they are South Asians or not, you know.

Researcher: How about the people who cannot access the online system?

Participant: I think they have provisions for them, knowing that they can't. They made me have people calling them who speak the language. I have a sending visit the community nurses. I don't want, I'm just guessing nurses or, or pulling them over to the, on, in single physical bases because we know a friend who works in a surgery and she'll think that we are not seeing any patients. Everything is on telephone on video. Well, we do need to see people. We invite them, we take them to the back door. We meet them there that they've got their process in place, you know. I am sure that’s not going not be any different to anybody else. I must make a comment to this effect. And maybe it's not relevant to you in this country on the whole in England. I think we're very fortunate, but I don't think we treat people differently. I'm not saying it's a hundred percent full proof. I'm sure there is inequality, racism, whether that's a colour bar or gender racism or a relative issue, I'm sure it's there somewhere. But when it comes to government type of things, state, I think on the whole it's fairly even and equal. But the private sector may be a different story.

Researcher: How do you think the community trusts the government that they are making the right choice for them during this crisis?

Participant ([49:45](https://www.temi.com/editor/t/Qxe1yjOtWj4mwEjv3chjjwiyV6DtJjKGjeSmYG0OHmk4UaMfVnPgipRbdjGKmUXZiFy3l2dP_VY0GvQyP1TFCq5WG18?loadFrom=SharedLink&ts=285.07)):I think there's a lot of, lot of trusts. Yeah. There is a lot of trust and people believe in the government, you would get the little bit of dissent entryway, you know, but there is trust. I don't have much respect for the journalists because I think they make their income out of being vocal and sensational. So challenging. It's good to challenge the government, but challenging them on the meeting, it is not the way to do it.

Researcher ([50:18](https://www.temi.com/editor/t/Qxe1yjOtWj4mwEjv3chjjwiyV6DtJjKGjeSmYG0OHmk4UaMfVnPgipRbdjGKmUXZiFy3l2dP_VY0GvQyP1TFCq5WG18?loadFrom=SharedLink&ts=318.5)):And how would you think, um, the messages would have been delivered in any better way to, to reach the South Asian people?

Participant: I think they have. I'm not sure again, but I think because... Some authorities have actually put up things, uh, in minority languages as well. They are trying their best. Yeah.

Researcher: So the language can help, translating.

Participant: Yeah. But again, it depends which generation you're talking about. My generation don't really need it because I, I, there are many people in my generation, who can't even read their own language. Right. So they are better informed in English.

Researcher: So what would you think that people who cannot talk or understand English, how the information is reaching them? If they are not translated?

Participant: Well in a, in a demographic, uh, Lester, when I lived, there has been some efforts made to reach out to those people by producing material in their own languages, people who speak, you know, for example, if you ring the doctor or the hospital, not the hospital, but the doctor then generally, as far as someone who speaks your language is available. The outreach is acceptable, you know?

Researcher: Right. So, um, we are nearly at the end. And the last question I would ask you to reflect on, is there any particular aspect you see of the South Asian community, which has helped to survive this crisis?

Participant: I think in some areas, but certainly there's been, and it's not been talked about my there's been the neighbourly support type of things, which we've been very good at. There's been areas where people have actually run out in front of you to the community in terms of food or welfare support or something like that. I don't mean money. I mean, people brought into them except for you though. Of course. I think we need to realize that a lot of medical stuff, frontline staff, South Asians, you know, nurses, doctors, care people. Yeah. Because they've been aware, important part of this whole process.

Researcher: What would you see like the religion spirituality or anything in particular may matter to the South Asian.

Participant: In terms of local level, there's not been an opportunity to disseminate that because the temples are closed, you know. The temples, mosques, gurdwaras. In terms of other sources, like tv channels- a wide range Indian tv channels are available. But I think there's a lot of a thing. There's an attitude going around that nature is aligning itself. You know, a lot of things that we should start, I think people are being educated and made aware of how we should be appreciating nature, how we should be taking care of nature. Pollution. You know for the wider things like pollute. Should be taking care of nature a bit, you know.

Researcher (55:00): This brings us to the end of the interview. Thank you for your participation.
