# Supplementary material for: Mitigating the identity and health threat of COVID-19: Perspectives of middle-class South Asians living in the UK
Source: J Health Psychol. 2021 Jun 22;27(9):2147–60. doi: 10.1177/13591053211027626 (PMC9353968; doi:10.1177/13591053211027626)
Supplement: sj-docx-9-hpq-10.1177_13591053211027626 – for Mitigating the identity and health threat of COVID-19: Perspectives of middle-class South Asians living in the UK [file sj-docx-9-hpq-10.1177_13591053211027626.docx]

**UK6-groupa-LMI-may20**

56 minutes

Transcription by: Sharmistha Chaudhuri

**Part-1**

Researcher :Thank you again for consenting to participate in this study.

Participant: No problem.

Researcher : I will start with the question like what do you think is happening to the world?

Participant: Well, at the moment, because of this Coronavirus going on in the world, it’s in a way when you look at the news it is a bit stressing at times. Everybody is suffering from this virus and people; some people are in lockdown and others have just uplifted the lockdown. All the time in the news it’s all about this virus all the time. Some people die. Some people are getting better and others are losing their lives. It’s worrying at the moment.

Researcher: Correct. What comes to your mind when you think of Coronavirus?

Participant: It’s a virus which started in China. When I first saw it on the news, you take it very seriously because it’s happening miles and miles and miles away from you. And then, gradually they starting telling us that it’s gone to Italy and then from Italy it comes to UK when it came to UK, 01.59. That is when you realise that something is going on in the world and at that time my son was in Kerala, in India. I am hearing in the news that in Kerala, the flights has been stopped, you know, we were 02.17 getting worried. And then, yeah, and then every day you hear that so many people are dying in the UK and that’s when you realise that something terrible is happening in the world.

Researcher: How do you think Coronavirus has affected the people in general?

Participant: I think it’s affected mentally and physically. But physically those who have contracted. Mentally those who are, you know, knowing they don’t they hear from the news and then families and so I think it’s affected both ways. Mentally and physically and some people it’s mentally. Even that thing I think with young children mostly that’s happening and they are away from school and they ask questions and see so much on the news. They hear from parents. No matter what age group and everybody knows what’s happening.

Researcher: Apart from the mental and the physical effect do you see any other effect to the people in general?

Participant: Mentally, yeah. Mentally it’s affecting lots of people and because of the lockdown they can’t see anybody. They can talk on the phone, but physically they can’t come to see their parents. Again, I heard it on the news that [Unclear] 04.08 happening quite a lot in the population. These people who have got the virus and they are in the first stages and some are coming back home, but they are getting better, but still mentally it’s still in their mind. That’s going to take some time for people to be cured and become a normal person again how they were before the virus, even though they got all away, they got better from this virus and still mentally, I don’t think they have.

Researcher: Do you think this is any different from any other country or this is specifically UK with the mental thing?

Participant: I think it has been getting there all over the world. It’s to do with the mind and no matter where you are, it doesn't matter what country you are, it’s to do with the mind. The location is not important.

Researcher: Absolutely. If you just think of like, I know you mentioned about the news, how have you learned about the Coronavirus like which other sources you refer to?

Participant: In the news or I think news feed. I watch BBC News quite a lot. I heard it on BBC. And then when I first heard it obviously I didn’t take it very seriously as it happened in China. Like I say, when it got here then I started watching the news all the time. Then after a while I thought no, I can’t be doing this watching the news all the time because it’s confuses me so much. You are hearing so much and it’s very confusing.

Researcher: Too much information.

Participant: Even now, switch on the television and lots of information. I thought, I don’t need to know that.

Researcher: Any other sources like the social media because a lot of the news are generalised through the social media like WhatsApp, Facebook?

06.45

Participant: When it was in China, I don’t think. I think when it came to this country and started in Italy I think then I started getting messages from WhatsApp. I am on Facebook, but you know, I am not very much on Facebook. WhatsApp, yeah.

Researcher: Do you read them or did that feed to your information?

Participant: I used to be on WhatsApp. All day people were sending messages and some were some made sense and others just didn’t make sense. Lot of them didn’t make sense, you know. 07.38.

Researcher: Can you tell us like with all the information going about, what do you know about the Coronavirus?

Participant: Personally, I know. 07.53. It’s very harmful. It’s nothing like viruses have been in the past. This is very unusual virus. Find out what exactly it is. This virus is basically, you know, killing people, you can’t see the virus. It is basically killing people. Very dangerous. Very dangerous virus.

Researcher: How does it spread? Do you know?

Participant: It spreads like if somebody has had the virus and like sneezes and you are near, you know, within two metres, you can easily catch or by touching the metal services or if you touched surfaces and the virus is still live and it’s been in the news that the virus can leave up—they said five days and then they said seven days and it’s like nine days for don’t touch things. If somebody is coughing, sneezing and that’s how it goes spreading very fast and especially in London maybe a lot bigger. We are away from London, but my son is in London. I don’t worry. It’s safe where he is. He doesn't go out much. He is just staying indoors and working from home.

Researcher :In your opinion, how did this virus appear?

Participant: Again, it said in the news I think wet market in China and that is where it came from. Also they are buying things, strange things and rest of the world.

Researcher: Animals. Can you elaborate on that like how it could have come from the animals?

Participant: Bats. In the news they said bats especially it could have come from the bats and then one probably, you know, something called bats or whatever things. Messages, I had a message saying, this Chinese having bats and that’s how it started. I don’t know whether that’s the truth or not.

Researcher: Any other theory you have heard of?

Participant: No.

Researcher: No. That’s okay and the news that it has appeared from the wet market.

Participant: Yeah and they were showing how the animals were caged.

Researcher: How do you think it appeared in the UK?

Participant: I think it came from Italy. It was February and February month is a skiing month you know. 11.54. I think some students went on a ski trip and brought it back home. Somewhere on the skiing, trip there were people from China and they brought the virus and then it spread from there. That is what I have heard anyway.

Researcher: Now something to reflect like since the beginning of the pandemic has your perception about the Coronavirus changed?

Participant: Yes, it’s changed. Totally- like it used to be, and it changed, it looks like now that even when it came over if it’s going to be over, we don’t know that. Life is going to be very very different, very different. Not a normal life. By that time, I may not be in this world. 13.12.

Researcher: Absolutely. I agree with you. In the beginning, you didn’t think about it when it was beginning like when it appeared in the news.

Participant: No. Because, I mean, because of the viruses, 13.29 that’s come and go. It didn’t spread this virus was spreading. When we started watching the news and the people are dying and so we thought okay, it’s in their country people are dying. Never imagined that it’s going to go everywhere in the other countries.

Researcher: When you say that life would be different, what do you mean by that?

Participant: Different meaning. You know, before we used to be in a big gathering like wedding and any event. We have big weddings of 14.25 thousands of people.

Researcher: A friend of mine that people raised from other people. Like you move away, and keep a distance. You know, that's very much going to save you first, for a while.

Participant: Right. And what about like any other habits, like your daily hygiene or your other daily activities? How do you think it can affect?

Researcher: Because most of us are staying indoors. We don't go out much. I mean, my shopping, I used to do them, but up till now my daughter was doing my shopping. But I've just started doing that. Even then I, go for once a week. Things like the wash, I keep washing the stuffs after shopping. That's what has changed now. It's not like before- you do the shopping, it is painful, it is empty, you put everything away, so that’s not so good. Yeah, in the house, I mean, you know, every now and then I am wiping the surfaces- when we come back, we wash our hands, spray over things- so you know, a lot of things has changed.

Researcher (17:00): How do you think this pandemic is different from any others in the past? Though you have mentioned like other virus has come and gone, but anything else you can add to that?

Participant: The last, I think the last one was Ebola. We were hearing in the news that people were dying. But this virus, it's different because it has spread to the whole world. But Ebola was not spread up- it was in one place. So that's the difference.

Researcher: And, and what is the impact it had as such do you think is different from the other pandemics- with, the people affecting their livelihood?

Participant: Yeah. This virus has a, you know, a bigger impact because the country has locked down. So that's the biggest impact on the countries. So again, this applies to the whole world because everybody had to shut down their own country. It's bigger, bigger, bigger impact on people's physical and mental state.

Researcher: And what would you think about your government's response to the pandemic?

Participant: because I mean, I stopped watching the news. From the start, they were very slow to take right action, you know? They should have stopped people dying. It should have been about the numbers, and when people started to die you lock down then. Maybe you could have saved people. More people. And they were bit slow to take action.

Researcher: And what was the information about corona virus that most surprised you?

Participant: That it was killing people.

Researcher: Right. And how, how was it any different from the earlier ones?

Participant: Because this virus is affecting the lungs! Those who are dying, it is very difficult for them to breath. And also I heard in the news that more men are dying than women, I don't know why more men men are dying than women. Black and ethnic minorities are dying more than white. Ii do not know why that is!

Researcher: And when you discuss about coronavirus with other people like with friends, relatives, what do you mostly talk about?

Participant ([21:04](https://www.temi.com/editor/t/uBuozhz9XFHkK0Ku2erdgiYQr0aQWvh8GpLKe1DvASq05sy-pn0IyEtm0sBf5QZH6mSZdFHmiGYZD8dyOHN5xO0IReI?loadFrom=SharedLink&ts=364.9)): when it started, and I was getting these messages on the WhatsApp and I have a bhabi (brother's wife is called bhabi in Hindi), my brother's wife, but I knew they had no restrictions put in. Then I showed her a message I received, it was the, I think it was the doctor saying that, you know, how to keep yourself safe because you know, she, she doesn't speak much English. I showed her the video, you know, and I said, look, you should not go around. You must not do these, do that! And that may make you aware what was happening...sorry this is I got as per the question!

Researcher: I know, these are open ended questions so that you can have rather more interpretation and from as many people we can. This is such like, if I can, if I can elaborate, like if you're speaking to your son or daughter, like, as you said, your bhabi, what do you talk about these days?

Participant: Yeah. I think we don't talk much now! In the beginning, you know, every time I my son calling me, I start talking about the virus. Now, he made me aware this month, ma, can we talk about something else?

Researcher: I think that's a better idea. Yes, yes, definitely!

Participant: Yeah, then we stopped talking about it, but when he calls, we talk my friends. Earlier, we would talk about the virus, the precaution, to do this thing and that; then it got so boring. So then yeah. Then we stopped talking about the virus.

Researcher: So why do you think your son requested not to talk about the virus?

Participant: Because it's the depression, and also, you know, I was telling my son, and he said, mum, I do read the news. So he gets the information from the news. He goes, you don’t have to repeat them to me. I think we need to be more on the positive side. I think we should talk about other things. Not to talk about the one thing which would bring depression. It can affect the mind- now as he says, I mean, now he doesn't talk much about it.

Researcher: And how, how your own personal life has been affected by these pandemic?

Participant: My personal life has not been affected much because I was, I was at home. Last two years back, I lost my job. I was made redundant. So, so, and I'm home now. I mean, I got a work in a shop in January and that's where I was going. Few weeks back, they said they don't need me anymore- obviously the shop closed, and then they don't need the staffs.

Researcher: So if these pandemic had not been there that you had been working?

Participant: Yes, oh yeah. It was for some time only. I mean, I only just started in January, middle of January, and then in March they said we don't need you anymore. Therefore, I am home. Personally, it has not affected me much. I am just living my daily life, in a routine.

Researcher: And do you see any positive side of it on your life?

Participant: At the moment..Um well the answer is that it hasn't changed for me much. Unless I go out. When I am in the house and you know, it's like, I'm living a routine life. Wake up in the morning, have breakfast, then do the cleaning and cooking, this hasn't changed in my life. But when I go out, we'll shop a couple of times. But it's not a normal month. Wearing masks, wearing gloves, doing the grocery shops, and then the cleaning up..that way, when I'm out, then yes. I can see the changes itself. But when I am in the house it is the normal thing.

Researcher: And can you tell us a bit about how your daily life is, you've already spoken about your daily life, how your life goes about?

Participant: Just normal, you know, waking up, breakfasting, cleaning, watching bit of tv, and then do the cooking, and we have lounge and then we go out for a walk, you know. I'm not going to the gym anymore, since March it has closed down, when we had the lock down. That has affected me, I try to do my exercise at home, but we are hoping things get better soon.

Researcher: So you still go out for a walk?

Participant: Yeah. Once a week. Once I think.

Researcher: How do you think this pandemic is going to end?

Participant: Oh! at the moment, I think even the scientists don't know how it will end. We are talking about vaccine, but it's a thing, it's yet to believe it, you know!

Researcher (28:38):And this is the last question of the section one which is, how do you think we might be able to prevent such pandemic in future?

Participant: I said, no, I have no idea.

Researcher: Do you think we can take any lesson from this one?

Participant: But do they? Has it not that happened before?

Researcher: That's a very good question! And do we learn?

Participant: Oh, I don't think we learn. We haven't learned things from the past! So I don't know whether we will learn from this! When, you know, everything will be back to normal again, you know, human mind is very quick, to forget easily. So you know, in the news, sometime ago, a lot of politicians are upset- elections are bad during the pandemic. I remember..amazing, President Obama. It was about like five years ago. And in his interview, he also said that we need to be prepared for these kinds of events which happened, but have we learnt from that? What people say? You all meant to keep up this pace. No, I don't think so!

Researcher: So you say, the humans, the society, will remain the same after the pandemic is over?

Participant: No. I think peoples' mentality will change for better I think. People would be more more affection to other people. So that way it is going to change and...at the moment, you know I don't like people complaining about this lockdown. This lockdown has a very positive side- people looking at their family, having more time to spend with the family, like, you know. Most men, men are working more, more hours, you know, they get more time to spent with the wife, the children, with the partners. And yet some people are not happy so, well, I don't know what, you know, what type of happiness they looking for.

Part II

Researcher: Okay. So we have ended the first part of the interview. So I have a few more questions and will start the section two, in which our focus will be on the South Asian community. So I will start by asking you, what do you think are some of the health concerns for the South Asian community during the pandemic and why.

Participant (32:00):The health or to do with virus?

Researcher: How they may be more or less at risk to the virus or what is their health condition, which may be related to having the virus, compared to white British.

Participant: Okay. I think the most important thing is the diet. Yeah. I saw most of them suffer from diabetes, high blood pressure. Lack of exercise. I used to go I see lots of Asian 'kakas' (uncles), elderly, you know. What they do is, I mean, they go to the gym... They just sit in the jacuzzi ...being lazy, in the gym. They just sit there; they just watch other people (laughs).

Researcher: That's a separate thing altogether, but yes!

Participant: I think, lack of exercise, diet, lot of eating, many factors!

Researcher: So how we can be related to the coronavirus? Their health?

Participant: They're they're more... let's just say risk vulnerable. More at risk. But I, I don't know. So thinking about this, I have a doctor friend, I was asking- she was telling me, most of the people dying are black. She was saying, I could not figure this out ,why black people are dying? No idea, but why so many blacks dying? So more than whites!

Researcher: Did you also hear that South Asian people are dying more than the white people?

Participant: Yes. On the news, I think. Um it was about this young man. His father died and my uncle died, but they've not been out on the street that they've saved indoors, that they have wine with in the side with the [inaudible]. So he might've brought the food and they said so most things I'm finding that she's more accurate.

Researcher: And how old do you think the Southeast Asian community has been specifically affected by this coronavirus?

Participant: No, I think it is it factored in health issues.

Researcher: You can think about their lifestyle. You can think about their livelihood here.

Participant (37:00) Oh, that's right. Yeah. You know, my understanding is there shouldn't be any difference between other people. Yes. Everybody here is same.

Researcher: Correct. And though you have mentioned some, but I would repeat the question again. How has your family been affected by the coronavirus?

Participant: In a way that I think, me and my husband are ok with this. There is not much difference than before. But with my kids with my son, you know, he very workaholic also. So he I think it's kind of affected them, more than us. My daughter, she, she can't work from home, so, that way, it has affected her. She could not go to the class and she was just happy for a couple of weeks to have her classroom closed. But my son things to the type of person he is, he is so used to be working with people.

Researcher: So it's basically that the restriction of the lock down has affected them mostly.

Participant: Yeah. Restriction maybe. Yeah.

Researcher: Do you think your daily...your has not...which you have mentioned, but do you think family-wise taking your entire family, that daily routine has any significant changes?

Participant: No. I said, it’s even better because, we can talk to our son every day on Facetime, we've been saving that!

Researcher: I would say definitely better.

Participant: Better than before, when he would ring us. Before he was like, you know, when he got a chance, like, hi mom, hi dad. Now. Yeah. Spending more time on the phone, you know.. Yeah.

Researcher: So how, how would you think these entire South Asian community and the British community, how it has affected, how can you compare, or can you compare any difference of the effect between the communities?

Participant: No, I cannot compare!

Researcher: It's more or less you see that as same as globally affecting everybody, right?

Participant (40:00): Yeah. It says, you know, the government, so as they say, there are black family, white family..! They do not have to say that!

Researcher: Correct!

Participant: I don't know, maybe our Asian people, because we are more like family people, we tend to be spending more time with the family, but maybe that's one way of affecting the South Asian.

Researcher: I think that's a very good point actually. Yeah.

Participant: But the white people, they mostly live with themselves. They are not out and open. But we are. Yeah. Yeah. Very much people, people's person. So that's me. Yeah. Maybe kind of..

Researcher: And if you look at the lot of different measures, like lock down, working from home or social distancing, restrictions on travel, et cetera, how do you think the South Asian community is coping with these changes?

Participant: Some are very ignorant, some are not following the rules. So I would say 10% are following the rules and others are not following the rules. They are ignorant, honestly.

Researcher: Why do you think this difference could be?

Participant: Lack of understanding 'who cares?' that of attitude.

Researcher: So, so you look at, there may be a difference in the attitude?

Participant: Yeah. Yeah. Definitely, it's the attitude. That this is not going to happen to me! Yeah.

Researcher: Denial? So the consciousness is more needed you think?

Participant: Definitely! Yeah, right? Yes. If you look on the street, in the wider population and everywhere!

Researcher: And looking at the health facility, how do you think this, the South Asian community are more are at ease or have more difficulty or can easily access the healthcare facilities?

Participant: It shouldn't be any different between white and the Asians.

Researcher (43:00):Like approaching the services or contacting any healthcare workers?

Participant: Good. That's yeah. You know when you have to order repeat prescriptions, you can order online. I know some Asian population, and they find it difficult, as they can’t log-on, they can't be in computers, sometimes they can't find the correct options, But they can always use a form from the surgery. And, mind also there, there are some Asian people going into surgery: one of my friend is working in a surgery, and they they, don't open the door. So there's a button to ring them from outside. Um, y have to make an appointment to come in. So that way they are more ignorant, about what they should do and what they can't do.

Researcher: Why do you think, where this ignorance is coming from, and why it is different from white people?

Participant: Please don't think me stupid- but they are so used to, used to go to the surgery. They say hello to the doctor, and then when this virus thing was announced, they were .. They were told to stop doing that. But honestly, you can't!

Researcher: And how would you think the South Asian community trust the government that they have taken the right decision?

Participant: Well, first of all, they haven't taken the right decision, so the trust is not there! (Laughs). So I think because of the language issues, you know, they cannot express what they feel, what they want to say. So they are just following the crowd.

Researcher: So language is the main barrier you think!

Participant: Yes, language is the main barrier. And I suppose either way, because in English, maybe English news, I am sure they are watching any other channels, whichever language, may be in Hindi. And I'm sure they would get the core information from that channel, you know? So I think they would be much, much aware of it.

Researcher: Yeah. And how would you think any, this messaging service to the community could have been improved by the government?

Participant: In what way?

Researcher (46:49): So that they are more aware, so that they have more understanding do reach the community. Do you think anything could have been done to reach the information about the virus, about what they should be doing, to the community level?

Participant: Yeah. I don't know how the mandirs, the gurudwaras, the mosques... I don't know, how they relayed the messages to the, to the public. So, that works one way of relaying the message to the public, and whether they did that, I am not sure, whether they did that or not. Um I think the elderly generation, probably got the messages from their children, most of them live with the family, you know. Probably the children within the family are relying messages to the elderly people: what they can do and what they can't do. They are not to go to the shop and the children do the shopping. I know my, one of my cousin has a relative, she lives on her own, she is in her 80s. Her children told her she go cannot go around, she cannot go to the grocery shop- they arranged shopping to be dropped off. Yeah. So I think that, you know, children are messaging the parents. Yes.

Researcher: Because you mentioned about this mandir, gurudwara, mosque: but they may have just closed down by the time, when they were hit by the pandemic.

Participant: I think. Yes. But then they must have, they must have information- why they were closing down. They wrote them down. I know some of the mandir, which closed down, one week before the lockdown, because they had to protect the elderly population. On Mondays and Wednesdays, there was a play group, mostly, with people which was quite regular, with young people, and they were worried about the elder generation. And I think therefore they shut down before the lockdown. They must've been informed about the lockdown- that this is happening, to make them aware.

Researcher: How do you think this messaging services could be better in any way?

Participant: Messaging for everyone?

Researcher: For the general community as a whole.

Participant: Yeah. so messaging, you know, what you see in the news. We see, everywhere, in the news, in the radio! For the elderly, I think they just watch the news on the television, and listen to the radio. You know, not many people have a mobile phone, it is mainly for the children. The newspapers? I dont know!

Researcher (51:00): As you said, technology may be a challenge to these generations.

Participant: Yeah, mine too. My age group! Well, my, friends, in their seventies, they say, they struggle! They struggle a lot.

Researcher: We are coming to the final question now, something to think about what do you think has helped you and the South Asian community as a whole to deal with this crisis? Any particular thing you can think of that they rely on to come out of the crisis?

Participant: Coming out of the crisis is very very far-fetched. Coming out the crisis means, you have to start enjoying your life, people need to go back to work, but be safe at the same time, I think everybody's waiting for that announcement. Coming out of the crisis, I think is the only way out. Can't see any other way round. Um and then we will have lots and lots of people will be losing their jobs. The crisis we fear, will be there for some time.

Researcher: So how would you cope with the crisis if things has happened for real? So now people have lost jobs. People has death; been locked down. So any particular things, you can see that people rely on, for example, like religion or anything else you can see as a positive, which can give them strength.

Participant: Yeah. So I, as an elderly generation probably, when the lock down is over, we will start visiting the temples, that is their way of dealing with it. Young people will go back to work. You know, the people in the middle...I don't know. Personally for me, I will go back to swimming and gym! That is my normal life. I would then know life has come back to the normal. I think it is more on individual person. For my husband, it is when he is able to travel. But then again, I'm not, I'm not so sure that how safe it would be, so that would be worrying for me about, about struggling.. here are lots and lots of issue. Uh, nice when this big issue is over, there will be little little things we are not thinking about.

Researcher: So, yeah, we have come to the end of it. And thank you so much again. So do you think you missed out anything or do you want to add anything?

Participant: Not at the moment, but maybe the night, when I'm lying down, and think about it- that I should have said this way or I should have said that way.

Researcher (55:11):Yes, for the next time, it might help if you remember how your representation has changed, how your understanding has changed.

Participant: We talk about that. Yeah.

Researcher: Thank you for your participation!
